# Accelerating the end-to-end production of cyclic phosphate monomers with modular flow chemistry†

**DOI:** 10.1039/d2sc02891c

**Published:** 2022-07-30

**Authors:** Romain Morodo, Raphaël Riva, Nynke M. S. van den Akker, Daniel G. M. Molin, Christine Jérôme, Jean-Christophe M. Monbaliu

**Affiliations:** Center for Integrated Technology and Organic Synthesis, MolSys Research Unit, University of Liège B-4000 Liège Sart Tilman Belgium jc.monbaliu@uliege.be; Center for Education and Research on Macromolecules, Cesam Research Unit, University of Liège B-4000 Liège Sart Tilman Belgium; Department of Physiology, Cardiovascular Research Institute Maastricht (CARIM), Maastricht University 6200 MD Maastricht The Netherlands

## Abstract

The biocompatibility, tunable degradability and broad functionalities of polyphosphoesters and their potential for biomedical applications have stimulated a renewed interest from Chemistry, Medicinal Chemistry and Polymer Sciences. Commercial applications of polyphosphoesters as biomaterials are still hampered because of the time and resource-intensive sourcing of their corresponding monomers, in addition to the corrosive and sensitive nature of their intermediates and by-products. Here, we present a groundbreaking challenge for sourcing the corresponding cyclic phosphate monomers by a different approach. This approach relies on the use of continuous flow technologies to intensify the end-to-end preparation of cyclic phosphate monomers with a semi-continuous modular flow platform. The applied flow technology mitigates both safety and instability issues related to the more classical production of cyclic phosphate monomers. The first flow module allows safe synthesis of a library of cyclic chlorophosphite building blocks and features in-line ^31^P NMR real-time monitoring. After optimization on the microfluidic scale, this first module is successfully transposed toward mesofluidic scale with a daily throughput of 1.88 kg. Downstream of the first module, a second module is present, allowing the quantitative conversion of cyclic chlorophosphites with molecular oxygen toward chlorophosphate derivatives within seconds. The two modules are concatenable with a downstream semi-batch quench of intermediate chlorophosphate with alcohols, hence affording the corresponding cyclic phosphate monomers. Such a continuous flow setup provides considerable unprecedented advantages to safely and efficiently synthesize a library of versatile high value-added cyclic phosphate monomers at large scale. These freshly produced monomers can be successfully (co)polymerized, using either batch or flow protocols, into well-defined polyphosphoesters with assessed thermal properties and cytotoxicity.

## Abstract 2

A semi-continuous flow platform allows the safer end-to-end production of cyclic phosphate monomers providing an easier access to polyphosphoesters and their applications.

## Graphical abstract

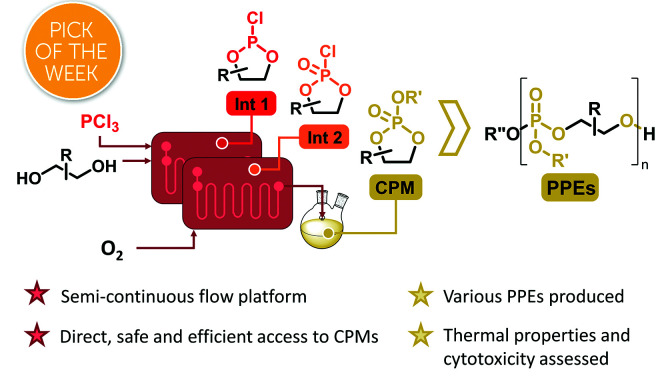

## Introduction

With major roles as building blocks of the genetic information through deoxy- and ribonucleic acids and as cell energy currency in metabolic processes through adenosine triphosphate, polyphosphoester (PPE) structures are ubiquitous in Biology.^1^ Contrastingly to polyesters (*i.e.* based on carboxylic acid esters), synthetic PPEs have only found scarce industrial applications such as flame retardants.^2–4^ However, PPEs have recently gained a renewed interest from the academic community in light of their potential biomedical applications^5^ such as drug delivery systems^6–12^ and tissue engineering.^13–17^

Synthetic PPEs are especially appealing for their tunable (bio)degradability and biocompatibility properties.^2,18^ A convenient synthetic method to obtain PPEs involves the ring opening polymerization (ROP) of cyclic phosphate monomers (CPMs), which enables the preparation of high molecular weight materials with narrow polydispersities.^19^ Among the various CPMs, 5-membered ring monomers are currently more popular compared to their 6-membered ring counterparts. The ring strain of the 5-membered CPMs makes them more reactive during polymerization, yet also more sensitive to hydrolysis, and prone to efficient organocatalyzed ROP.^2^ In sharp contrast to classical aliphatic polyesters, the physicochemical properties of PPEs, such as hydrophobicity,^20,21^ can conveniently be adjusted through straightforward modifications of the pendant side-chain on the pentavalent phosphorous during the upstream monomer preparation. Moreover, introduction of a suitable functional pendant side-chain also enables post-polymerization modifications^22,23^ and introduction of bioactive molecules,^24,25^ which further expands the scope of applications of PPEs.

**Fig. 1:**
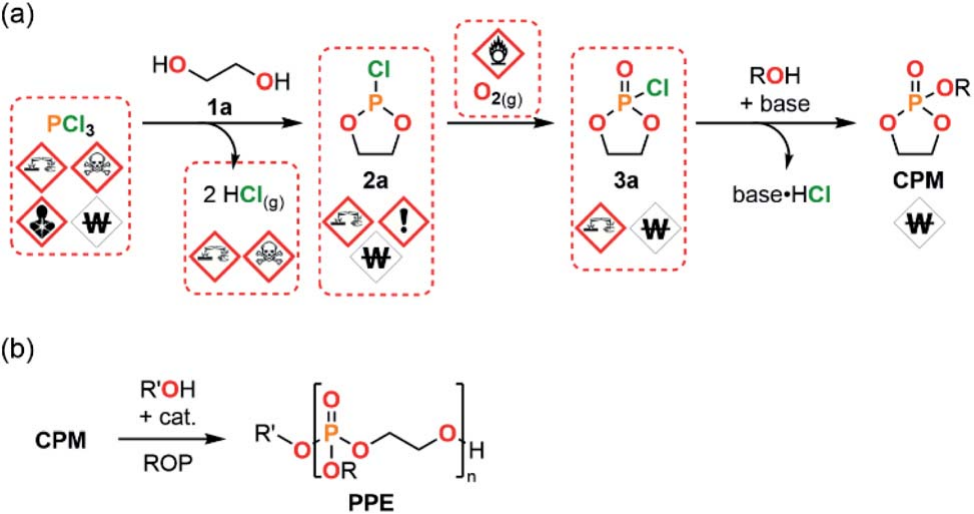
(a) 3-step synthetic pathway commonly used for the preparation of CPMs involving hazardous and/or sensitive reactants, intermediates and by-products. (b) Ring-opening polymerization strategy used for the production of PPEs starting from CPMs.

One of the most significant challenges met during the production of PPEs using ROP strategies actually relates to the sourcing of CPMs (Fig. 1). The preparation of such monomers is usually performed by a resource- and time-intensive 3-step procedure. The preparation of CPMs starts from PCl_3_ and a suitable diol (1a) to yield a cyclic chlorophosphite adduct 2a with the concomitant release of 2 equiv. of gaseous HCl. Next, the chlorophosphite intermediate is further oxidized into its cyclic chlorophosphate counter-part 3a. The CPM is obtained after a final functionalization using an adapted alcohol.^18,26^ This process involves the use of hazardous PCl_3_ as starting material, corrosive and sensitive intermediates (chlorophosphi(*a*)tes 2a and 3a) and produces large amounts of HCl as a by-product. These circumstances lead to significant safety concerns upon scalability trials.

To address these issues that challenge upscaling a modular semi-continuous flow platform dedicated to the production of CPMs has been developed. The platform allows to produce CPMs with unprecedented versatility, efficiency and scalability starting from bulk chemicals. The modular flow platform features the concatenation (*i.e.*, the connection within a same uninterrupted system) of 3 chemical steps and mitigates the inherent safety hazards related to production of large amounts of by-product HCl, as well as to handling of corrosive intermediates and reactants. Moreover, this approach also significantly reduces the overall process time for production of chlorophosphi(*a*)te intermediates in a continuous fashion (batch: 5 h to 4 d; this flow protocol: <2 min). This study also demonstrates that model CPMs are successfully polymerized through either batch or continuous flow protocols. The absence of cytotoxicity of selected PPE samples is also assessed, as well as their thermal properties. This contribution unlocks the production of CPMs and consequent PPEs, and thus opens new avenues to access novel biomaterials and large-scale production of industrially relevant PPEs by enabling their safer and efficient end-to-end production.

## Results and discussion

### Continuous flow preparation of cyclic chlorophosphites and analogs

The preparation of cyclic chlorophosphites in batch typically relies on the method of Lucas *et al.*,^27^ which involves the careful addition of a diol to a solution containing PCl_3_ with the subsequent release of HCl (2 equiv.). Even with appropriate care during the addition process, reckless release of HCl gas raises significant safety concerns upon scale-up. Taking these hazards into account prompted us to adapt the synthesis of chlorophosphites under continuous flow conditions for enabling a smoother and more controllable generation of HCl gas, as well as a fast and efficient production of cyclic chlorophosphites and analogs.

The first exploratory optimizations involved the preparation of model compound 2a through the addition of neat ethylene glycol (1a) to a concentrated solution of PCl_3_ in CH_2_Cl_2_ at room temperature. The outlet of the reactor was directly connected to a closed surge with a flow of an inert gas to continuously flush gaseous HCl out of the reactor effluent toward an alkaline aqueous trap. Starting from a 1 M PCl_3_ solution, reducing the residence time to 1 min allowed complete conversion of PCl_3_ (Table 1, entries 1–4). Next, to maximize the output, the concentration of the PCl_3_ solution was increased (entries 5 and 6) with an appropriate adjustment of the counter-pressure to avoid HCl degassing inside the coil reactor. A starting 3 M solution (entry 5) maintained complete conversion and excellent selectivity, whilst affording a 76% isolated yield after fractional distillation under reduced pressure. A very high hourly productivity of 14.8 g was obtained using a 1 mL internal volume coil reactor, despite its minimal footprint. Further increasing the concentration of the starting solution (entry 6) led to a biphasic regime and to the appearance of major side-products; thus, the concentration was set to 3 M for the PCl_3_ solution as a reference for the following experiments. A variety of solvents were assessed for the production of 2a, yet leading to similar (entries 7, 9 and 10) or lower (entry 8) yields. 2-Methyltetrahydrofuran (MeTHF) emerged as a more sustainable alternative to CH_2_Cl_2_, as it is biobased and more favorable than CH_2_Cl_2_ according to the CHEM21 selection guide.^28^ MeTHF also supersedes THF in terms of health and environmental scoring. In-line ^31^P NMR was conveniently implemented downstream the reactor for real-time reaction monitoring, hence accelerating the optimization process as well as improving process safety (see ESI, Section 2.5.1†). Implementation of in-line monitoring indeed contributes to ensure that hazardous PCl_3_ is fully converted and that no major impurities are present, while avoiding time-consuming high-field NMR analysis that could lead to a degradation of the crude sample containing a sensitive chlorophosphite. Chlorophosphite 2a was detected as a sole peak (∼168 ppm) during in-line monitoring while the absence of PCl_3_ (∼219 ppm) was confirmed. No other peaks were observed by ^31^P NMR.

**Table 1:** Optimization of the continuous flow preparation of cyclic chlorophosphite 2a^a^

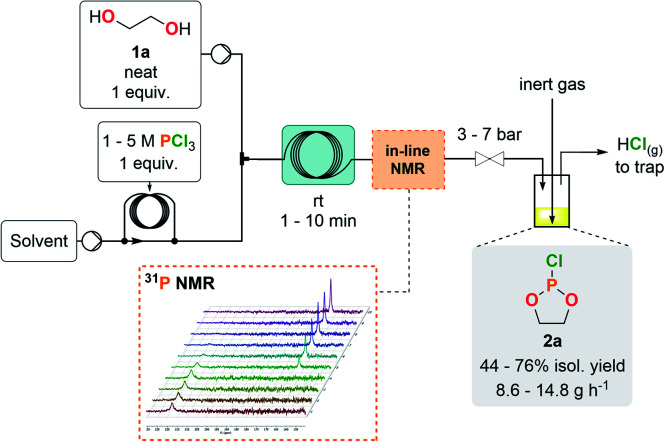							
Entry	Res. time [min]	Solvent	PCl_3_ conc. [mol L^−1^]	*P* [bar]	Conv. PCl_3_^b^ [%]	Isol. yield [%]	Prod. [g h^−1^]
1	10	CH_2_Cl_2_	1	3	>99	n.d.	n.d.
2	5	CH_2_Cl_2_	1	3	>99	n.d.	n.d.
3	2.5	CH_2_Cl_2_	1	3	>99	n.d.	n.d.
4	1	CH_2_Cl_2_	1	3	>99	n.d.	n.d.
5	1	CH_2_Cl_2_	3	5	>99	76	14.8
6^c^	1	CH_2_Cl_2_	5	7	>99	n.d.	n.d.
7	1	MTBE	3	5	>99	62	12.2
8	1	MeCN	3	5	>99	44	8.6
9	1	THF	3	5	>99	72	14.0
10	1	MeTHF	3	5	>99	74	14.4

a General conditions: room temperature, continuous flow coil reactor of 1 mL of internal volume.

b Determined by ^31^P NMR.

c Two phases present in the effluent of the reactor, impurities detected by ^31^P NMR. MTBE = methyl *tert*-butyl ether, MeCN = acetonitrile, THF = tetrahydrofuran, MeTHF = 2-methyltetrahydrofuran.

**Fig. 2:**
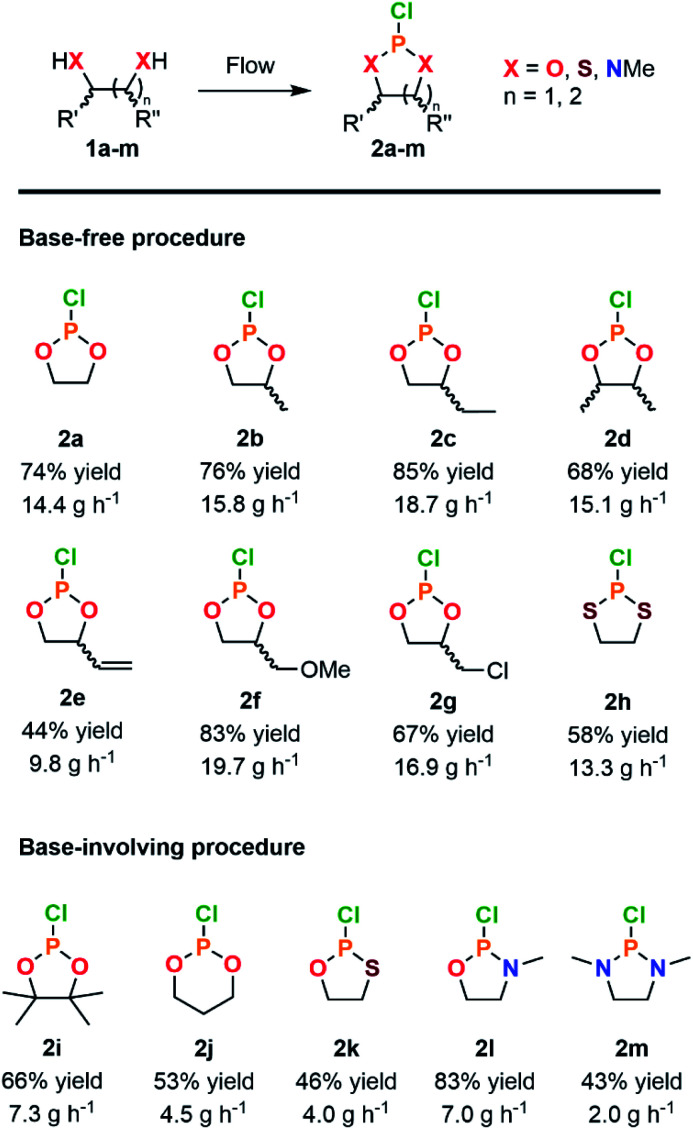
Scope of cyclic adducts 2a–m obtained from the continuous flow reaction of PCl_3_ with various diols and analogs through a base-free (see Table 1, entry 10) or a base-involving (see ESI, Section 2.5.2†) procedure.

With a highly productive and compact continuous flow system that mitigates the hazardous preparation of 2a in hand, the scope of the procedure was next extended to a variety of cyclic chlorophosphites and analogs 2. Starting from various 1,2-diol and derivatives 1 (including potentially biobased derivatives 1a–g, j, l, m)^29–33^ allowed to rapidly generate a library of chlorophosphites bearing a range of backbone modulations (Fig. 2). Good to high isolated yields were obtained for most examples, except for 2e for which the final vacuum distillation led to significant decomposition (see ESI, Section 2.4.1†). Switching from a 1,2-diol to a 1,2-dithiol (1h) accessed dithiaphospholane derivative 2h in 58% of isolated yield, which was reported for the functionalization of nucleosides^34,35^ and the preparation of phospholipid analogs.^36^

With diverse applications as a phosphitylation agent for the preparation of glycosyl donors^37^ or the derivatization of lignin samples for characterization by ^31^P NMR,^38–40^ the preparation of 2i was next envisioned. Exploring the reaction with hindered 1,2-diol such as pinacol (1i) led to a major loss of selectivity and precluded the isolation of the product. According to the prior Art,^41^ highly sterically congested pinacol-like diols are associated with a much slower formation rate toward the corresponding cyclic chlorophosphites, hence allowing for competitive formation of a biphosphite derivative. The latter, in presence of HCl, underwent dealkylation eventually leading to the formation of 2 equivalents of cyclic H-phosphonate. Attempts to avoid formation of such side-products relied on addition of a base to the reaction mixture; however, in order to avoid extensive precipitation of hydrochlorides upon continuous flow operation, organic bases known to form ionic liquids under protonation, such as 1-methylimidazole, were privileged.^42^ Minor adaptations from the original continuous flow system were implemented with the selection of acetonitrile as a solvent to allow solubilization of the ionic liquid (see ESI, Section 2.5.2†). Chlorophosphite 2i was isolated in 66% yield using the adapted system accordingly, leading to a hourly productivity of 7.3 g. With such an adaptation, preparation of more demanding building blocks 2j–m was foreseen (Fig. 2). The six-membered ring dioxaphosphinane 2j was successfully produced in the presence of 1-methylimidazole, leading to a 53% isolated yield. This derivative is used in the preparation of various six-membered CPMs.^2,18^ Oxathiaphospholane 2k and oxazaphospholidine 2i were produced in average to high yields, thus even further broadening the scope. Finally, the synthesis of the diazaphospholidine derivative 2m was developed but required additional adaptations to avoid clogging of the continuous flow system. Switching to 1,8-diazabicyclo[5.4.0]undec-7-ene (DBU) as a base and lowering the concentration of the starting feed solutions prevented precipitation of solid residues, therefore enabling the isolation of 2m in a moderate yield (43%). This continuous flow protocol offers a compact solution for on-demand production of versatile cyclic P(iii) adducts while enabling a gram-scale production in minutes and mitigating the safety issues related with synthesis of such derivatives.

### Scalability of the continuous flow preparation of cyclic chlorophosphite 2a

The optimized procedure for the generation of cyclic chlorophosphites in a microfluidic environment bears significant potential for further scalability trials.^43,44^ To this end, the preparation of 2a was transposed in a commercial glass mesofluidic continuous flow reactor (see ESI, Section 2.4.3†). The reactor setup consisted in two glass fluidic modules (FMs), fluidically connected in series (5.4 mL total internal volume). The production of 2a was conveniently handled with the controlled release of the HCl gas by-product through a dome-type back-pressure regulator (4 bar) downstream the reactor. The effluent was collected in a closed vessel under a continuous flow of argon to flush out HCl toward an alkaline aqueous trap. The mesofluidic scale was associated with an isolated yield of 74% and a daily production of 1.88 kg under safe conditions (STY = 348 kg per L per day).

### Cyclic phosphate monomers (CPMs) production in a semi-continuous flow platform

The concatenation of the following steps toward functionalized CPMs was envisioned. The oxidation of cyclic chlorophosphites is typically carried out with a stream of gaseous molecular oxygen which is bubbled in an organic solution containing the chlorophosphite. The reaction time needed for this batch procedure typically varies between 8 h to 4 days.^8,26,45^ Continuous flow oxidation of the model compound 2a was optimized using molecular oxygen. The lab-scale continuous flow setup included the upstream generation of 2a, which was further reacted with oxygen in a second PFA coil reactor (see ESI, Section 2.5.3†). Quick analysis of the effluent by a benchtop ^31^P NMR allowed to qualitatively control the conversion and selectivity of the reaction. A significant improvement of the selectivity was observed upon using a coil reactor with a smaller internal diameter and an arrowhead-type micromixer system. The mixing efficiency appeared as a much powerful leverage than residence time for the oxidation step, indeed, the slug flow regime of the gas–liquid system combined with a medium counter-pressure (15 bar), a smaller internal diameter of the coil reactor and a specific arrowhead micromixer (see Table S1 in the ESI†) leads to an improved interfacial area, mixing and solubility of oxygen within the liquid phase and, thus, to high mass transfer and improved performances. Quantitative conversion with 59% of selectivity toward 3a was obtained under 65 °C within 21 s of residence time in the presence of 4 equiv. of molecular oxygen. Despite these very promising results, chlorophosphate 3a rapidly degraded after collection.

**Fig. 3:**
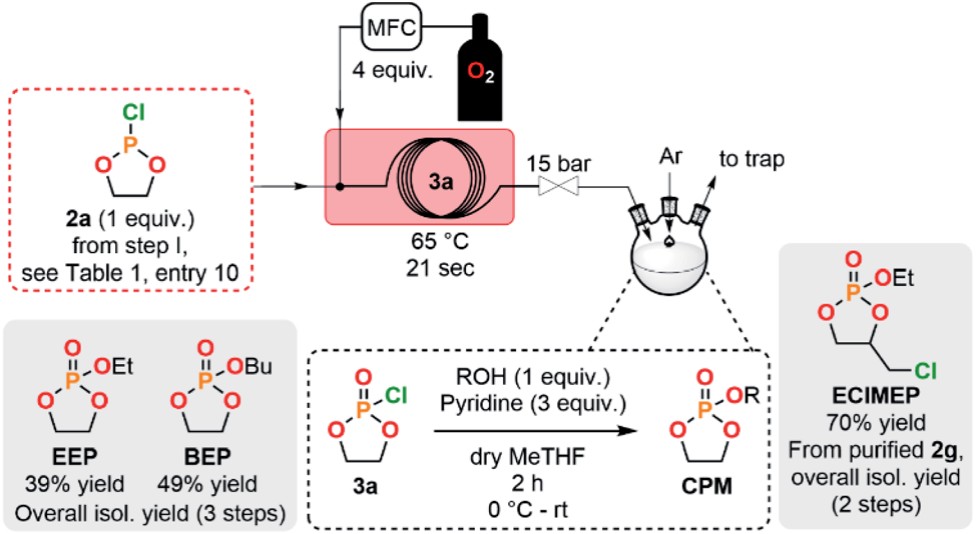
Concatenated semi-continuous flow process for the production of cyclic phosphate monomers (see ESI, Fig. S2 and S3† for the detailed setups).

It was therefore decided to directly implement the last condensation reaction to access CPMs by directly collecting the reactor effluent in a surge containing an alcohol and a base. Such a fully concatenated protocol would not only enable the neutralization of the remaining HCl gas, but also the reaction of the intermediate chlorophosphate to form the desired CPM directly from PCl_3_ and a diol. It was indeed foreseen that avoiding the handling of intermediate chlorophosphi(*a*)tes 2 and 3 would significantly increase process safety and efficiency. Using the optimized conditions for the preparation of 2a and 3a (see Table 1, entry 10 and ESI, Table S3, entry 8†), a semi-continuous flow system was developed (Fig. 3). The upstream generation of 2a allowed its direct and quick conversion to 3a and a final functionalization toward a CPM in the semi-batch reactor using an adapted alcohol in presence of pyridine. This flexible process allowed generation of various CPMs from bulk chemicals by conveniently switching the alcohol present in the collection surge. Two model monomers EEP and BEP were isolated in less than a day starting from PCl_3_ and 1a using this procedure and required only one final purification step. Moreover, the overall isolated yields obtained (39% and 49% for EEP and BEP, respectively) over the 3 chemical steps outperform the typical output achieved in batch (17–21%).^8,46^ The concatenation under continuous flow indeed contributes to reducing the number of purifications required and has thus a positive impact on the overall yield. Minor traces of a cyclic *H*-phosphonate were noticed in samples of EEP and BEP (see ESI, Section 2.5.4†) potentially highlighting a partial hydrolysis of 2a in the system.^47^ Starting directly from purified 2a led to a high purity monomer free of *H*-phosphonate impurities through a 2-step semi-continuous flow procedure (see ESI, Section 2.5.5†).

A novel EClMEP monomer bearing an ethoxy pendant group, additionally to a chloromethyl functionality linked to the cyclic backbone, was successfully isolated using an adapted procedure (see Fig. 3 and ESI, Section 2.4.5†). The 2-step process started from purified 2g, which allowed to avoid side-products from the synthesis of the chlorophosphite and, subsequently, to obtain high purity EClMEP in 70% of isolated yield over 2 chemical steps. A batch procedure for the preparation of the monomer EClMEP was additionally developed (see ESI, Sections 2.5.6–2.5.8†).

### Batch and flow (co)polymerization of cyclic phosphate monomers (CPMs)

The development of a semi-continuous flow system for the convenient and safer preparation of CPMs paves the way toward a better accessibility to PPEs as source for potential flame-retardants or biomedical applications at lab- and industrial-scale. To demonstrate the potential of this methodology, EEP was first selected as a reference monomer to assess its polymerizability. EEP ROP was first carried out in a batch reactor using DBU and a thiourea derivative (TU) as a well-established organocatalytic system^48^ in the presence of tetraethylene glycol (TEG) as an initiator (Table 2, entries 1–3). The EEP monomer produced through the semi-continuous flow platform was successfully polymerized accordingly (entry 1) with a good control of the macromolecular parameters. A well-defined polymer TEG-PEEP homopolymer with a narrow dispersity (*Đ* = 1.14) and an average molar mass of 13 600 g mol^−1^ close to the targeted value (12 000 g mol^−1^) was obtained. The small molar mass difference was attributed to unavoidable transesterification side-reactions at high monomer conversion.^48^

**Table 2:** Batch and continuous flow polymerization trials of EEP^a^

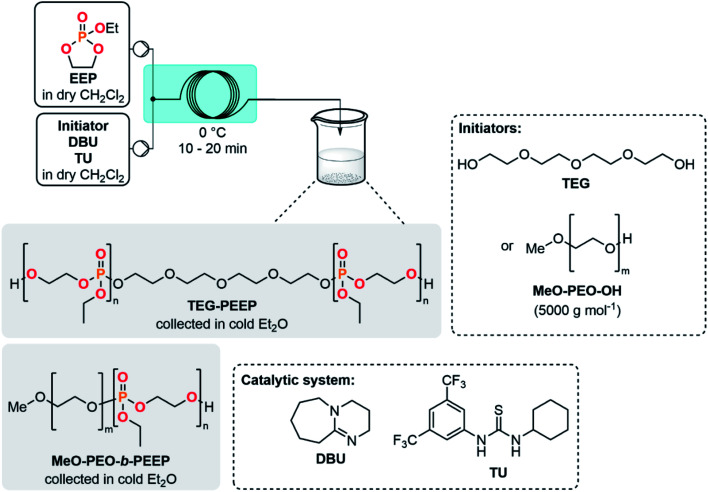										
Entry	Reactor	Initiator	EEP equiv.	DBU equiv.	TU equiv.	Reaction time^b^ [min]	*M*_n_ NMR^c^ [g mol^−1^]	*M*_n_ SEC^d^ [g mol^−1^]	*Đ* (SEC)^e^	*M* ^theo^_n_ f [g mol^−1^]
1	Batch	TEG	80	15.8	4.6	30	13 600	3300	1.14	12 000
2	Flow	TEG	80	15.8	4.6	10	5300	1700	1.06	12 000
3	Flow	TEG	80	15.8	4.6	20	9400	2100	1.11	12 000
4	Flow	MeO-PEO-OH	13	5	4.6	20	7000	8300	1.05	7000

a General conditions: temperature = 0 °C, atmospheric pressure, 1 equiv. of initiator, for the batch experiment 6.58 mmol of EEP used, for the flow experiments: conc._EEP_ = 1.10 (entries 2 and 3) or 1.65 (entry 4) mol L^−1^.

b Defined as residence time for continuous flow experiments.

c Average molar mass of the (co)polymer determined by ^1^H NMR as follow: *M*_n_ = DP_EEP_ × 152 + 194.23 for TEG-PEEP and *M*_n_ = DP_EEP_ × 152 + 5000 for MeO-PEO-*b*-PEEP.

d Determined by SEC according to a polystyrene calibration.

e Molecular weight distribution (PDI) determined by SEC.

f Targeted molar mass of the (co)polymer after complete monomer conversion: *M*_n_ = *n*_EEP_/*n*_initiator_ × 152 + 194.23 for TEG-PEEP and *M*_n_ = *n*_EEP_/*n*_initiator_ × 152 + 5000 for MeO-PEO-*b*-PEEP.

Polymerization reactions can also benefit from continuous flow systems, leading to lower dispersity and improved reproducibility by taking advantage of better thermal management and mass transfer.^49^ The homopolymerization of EEP was further explored under microfluidic conditions (entries 2 and 3) with inspiration from the study of Junkers *et al.*^23^ The outlet of the reactor was directly collected in cold Et_2_O, triggering precipitation of PPE. A residence time of 10 min at 0 °C (entry 2) led to a molar mass of 5300 g mol^−1^ and a low dispersity (*Đ* = 1.06). Interestingly, increasing the residence time to 20 min (entry 3) led to an increase of molar mass (*M*_n_ NMR = 9400 g mol^−1^) while keeping a low polydispersity index (*Đ* = 1.11). The molecular weight distributions were monomodal for TEG-PEEP homopolymers obtained under flow conditions, whereas a shoulder toward high molecular weight was observed for the TEG-PEEP sample obtained under batch conditions (see ESI, Sections 2.8.1–2.8.3†).

Design of novel block or graft copolymers based on the association of poly(ethylene oxide) (PEO) and PPEs have already demonstrated their potential for applications such as drug delivery systems^9,24,25,50–52^ and hydrogels.^53,54^ High reproducibility of continuous flow procedures is particularly appealing for preparation of such biomaterials to face demanding regulatory policies required for potential commercialization phases.

Using a PEO-based macroinitiator (MeO-PEO-OH, 5000 g mol^−1^) in conjunction with a DBU/TU catalytic system and based on the batch study of Jérôme *et al.*,^20^ the polymerization of EEP was carried out toward the formation of a MeO-PEO-*b*-PEEP diblock copolymer under continuous flow conditions (entry 4). Performing the reaction at 0 °C within a residence time of 20 min led to the targeted *M*_n_ of 7000 g mol^−1^ toward a well-defined block copolymer (*Đ* = 1.05). Cytotoxicity assays of MeO-PEO-*b*-PEEP on bovine fibroblast cells and human umbilical vein endothelial cells revealed that no cytotoxic effects were observed for a concentration of up to 0.1 mg mL^−1^ of copolymer (see ESI, Section 2.5.12†). These results are in agreement with data collected in a previous study regarding PEO–PPE diblock copolymers,^55^ which paves the way for a potential biomedical application.

Following the encouraging results collected for the polymerization of EEP, the polymerization of EClMEP (obtained from 2g, see Fig. 3) was investigated for the production of novel PPEs. Indeed, the presence of a chloromethyl functionality on the polymer backbone will first modulate the physicochemical properties of the corresponding poly(EClMEP) and additionally enable further post-polymerization derivatization as similarly described in literature for poly(ε-caprolactone).^56,57^ Moreover, the introduction of a chloromethyl moiety directly linked to a carbon of the polymer main-chain could render a more stable functionality after derivatization over elimination by hydrolysis compared to a more conventional functionalization through the pendant side-chain linked to the phosphorus atom which can be degraded under basic or acidic conditions.^58^ For this purpose, homopolymerization of EClMEP monomer was carried out in a batch process with similar conditions used for EEP. Unfortunately, these conditions are not adapted for the polymerization of EClMEP and did not lead to the formation of the expected homopolymer. This might be explained by a higher steric hindrance compared to the EEP monomer but also by the potential formation of a secondary alcohol at the ω chain-end after the initiation step, which could be insufficiently reactive to polymerize further with a second EClMEP. Nevertheless, random copolymerization of EClMEP with EEP using benzyl alcohol (BzOH) as an initiator was successful when the molar ratio of EClMEP in the co-monomer feed was lower or equal to 0.50 (Table 3, entries 1 and 2). BzO-PEEP-*co*-PEClMEP copolymers of two different compositions and molar mass were prepared. Monomodal molecular weight distribution with low dispersity were obtained for both copolymers.

**Table 3:** Batch (co)polymerization trials toward BzO-PEEP-*co*-PEClMEP and BzO-PEEP^a^

Entry	Target polymer	Molar fraction of EClMEP in the comonomer feed	Molar fraction of EClMEP^b^	*M*_n_ NMR^c^ [g mol^−1^]	*M*_n_ SEC^d^ [g mol^−1^]	*Đ* e (SEC)	*T*_g_ f [°C]
1	BzO-PEEP-*co*-PEClMEP	0.50	0.50	11 300	4000	1.15	−69.7
2	BzO-PEEP-*co*-PEClMEP	0.25	0.31	4700	2300	1.08	−61.5
3	BzO-PEEP	0	0	3300	2600	1.05	−71.9

a General conditions: initiator = benzyl alcohol (BzOH, 0.1 mmol), *n*_EEP_ + *n*_EClMEP_ = 17.5 mmol, *n*_DBU_ = 6.5 mmol, *n*_TU_ = 2.3 mmol, temperature = 0 °C, reaction time = 30 min.

b Molar fraction of EClMEP subunits in the copolymer determined by ^1^H NMR.

c Average molar mass of the (co)polymer determined by ^1^H NMR as follow: *M*_n_ = DP_EEP_ × 152 + DP_EClMEP_ × 201 + 108.

d Determined by SEC according to a polystyrene calibration.

e Molecular weight distribution (PDI) determined by SEC.

f Glass transition temperature determined by DSC.

Regarding the effects of the chloromethyl functionality on the physicochemical properties of both BzO-PEEP-*co*-PEClMEP copolymers as compared to a BzO-PEEP homopolymer reference (entry 3), a limited influence on the value of the glass transition temperature (*T*_g_) of the copolymers was observed. Both copolymers are amorphous with a *T*_g_ varying between −70 and −60 °C. Concerning the thermal stability, measured by TGA (see ESI, Sections 2.8.5–2.8.7†), the presence of the chloromethyl moiety modifies the degradation profile of BzO-PEEP-*co*-PEClMEP copolymers with an increase of the percentage of combustion residues at 600 °C compared to BzO-PEEP. These results pave the way for potential applications of these new copolymers as flame-retardant materials which are currently investigated in a follow-up study.

## Conclusions

A semi-continuous flow modular platform is described and allows convenient and safer preparation of CPMs directly from bulk chemicals. The first module was dedicated to the preparation of cyclic chlorophosphite building blocks with high productivities despite a small footprint system. This procedure allowed to efficiently mitigate the safety concerns inherently associated with the preparation of CPMs, while benefiting from real-time ^31^P NMR monitoring. The process was further expanded to a large scope of cyclic chlorophosphites and (*O*,*S*)-, (*O*,*N*)-, (*N*,*N*)- and (*S*,*S*)-analogs. The preparation of a model cyclic chlorophosphite was transposed to pilot-scale with a mesofluidic reactor upon minor parameters adjustments leading to a daily productivity of ∼2 kg. Next, the concatenation of the first module was implemented with (a) a downstream continuous flow oxidation using molecular oxygen and (b) a subsequent final functionalization using an alcohol in a semi-batch reactor. The oxidation step toward a cyclic chlorophosphate was intensified by taking advantage of a high mixing efficiency of the continuous flow system leading to a residence time of 21 s to achieve a quantitative conversion of the chlorophosphite. Finally, both cyclization and oxidation modules were telescoped with a downstream semi-batch reactor to conveniently perform the final functionalization toward CPMs using an adapted alcohol. The fully integrated semi-continuous flow platform allowed the production of CPMs while mitigating significant safety hazards. Next, a model CPM, EEP, obtained from the semi-continuous flow platform was successfully (co)polymerized by batch and continuous flow procedures leading to well-defined and biocompatible PPEs. Finally, EClMEP, a novel CPM bearing a chloromethyl functionality was successfully copolymerized leading to new PPEs with promising thermal properties. With the development of a semi-continuous flow platform for the efficient end-to-end production of CPMs, we forecast an accelerated and easier access to PPEs and their industrial applications by enabling a safer, more convenient and scalable preparation of the source monomers.

## Data availability

All experimental supporting data are provided in the ESI.†

## Author contributions

RM planned, executed, optimized the experiments in batch and flow for the preparation of CPMs and wrote the manuscript. RM and RR performed the polymerization experiments in batch and flow. RR executed the assessment of thermal properties of PPEs. NMSVDA and DGMM are responsible for the biocompatibility studies and related cytotoxicity assays and provided feedback to the manuscript. CJ advised and proofread the manuscript. JCMM supervised the project, advised, and corrected the manuscript.

## Conflicts of interest

There are no conflicts to declare.

## Supplementary Material

SC-013-D2SC02891C-s001
