# Supplementary material for: Accelerating the end-to-end production of cyclic phosphate monomers with modular flow chemistry
Source: Chem Sci. 2022 Jul 30;13(36):10699–706. doi: 10.1039/d2sc02891c (PMC9491087; doi:10.1039/d2sc02891c)
Supplement: SC-013-D2SC02891C-s001 [file SC-013-D2SC02891C-s001.pdf]

Electronic Supplementary Material (ESI) for Chemical Science.
This journal is © The Royal Society of Chemistry 2022

Electronic Supplementary Information for:

Accelerating the End-to-end Production of Cyclic Phosphate Monomers with Modular Flow Chemistry

Romain Morodo,[a] Raphaël Riva,[b] Nynke M. S. van den Akker,[c] Daniel G. M. Molin,[c] Christine Jérôme[b] and Jean-Christophe M. Monbaliu[a,]*

[a] Center for Integrated Technology and Organic Synthesis, MolSys Research Unit, University of Liège, B-4000 Liège (Sart Tilman), Belgium

[b] Center for Education and Research on Macromolecules, Cesam Research Unit, University of Liège, B-4000 Liège (Sart Tilman), Belgium

[c] Department of Physiology, Cardiovascular Research Institute Maastricht (CARIM), Maastricht University, 6200 MD Maastricht, The Netherlands

Table of Contents

Continuous flow setups 4
1.1 Microfluidic setups and parts 4
1.1.1 Pumps 4
1.1.2 Mass flow controller 4
1.1.3 PFA tubing and coils 4
1.1.4 Connectors, ferrules and mixers 4
1.1.5 Check-valves 4
1.1.6 Back-pressure regulators 4
1.1.7 Thermoregulatory devices 4
1.2 Mesofluidic scale setup 4
1.2.1 Pumps 4
1.2.2 Mesofluidic reactor 5
1.2.3 Thermoregulatory devices 5
1.2.4 Back-pressure regulators 5
1.3 Part numbers & vendors 5
1.4 Detailed continuous flow setups 7
1.4.1 Continuous flow setup for the preparation of cyclic chlorophosphites and derivatives **2a-m** 7
1.4.2 (Semi-)continuous flow setup for the preparation of cyclic chlorophosphate **3a** and EEP and BEP monomers 8
1.4.3 Semi-continuous flow setup for the preparation of 4-(chloromethyl)-2-ethoxy-1,3,2-dioxaphospholane 2-oxide (**EClMEP**) 9
1.4.4 Synthesis of 2-chloro-1,3,2-dioxaphospholane (**2a**, mesofluidic scale) 10
1.4.5 Continuous flow polymerization toward **TEG-PEEP** and **MeO-PEO-*b*-PEEP** 11
2. Additional experimental details 12
2.1 General information 12
2.2 Chemicals 13
2.3 Experimental setup 14
2.3.1 Microfluidic setups 14
2.3.2 Mesofluidic setup 15
2.4 Typical runs 15
2.4.1 General continuous flow protocol for the preparation of cyclic chlorophosphites and derivatives **2a-h** (base-free procedures) 15
2.4.2 General continuous flow protocol for the preparation of cyclic chlorophosphites and derivatives **2i-2m** (base-involving procedures) 16
2.4.3 Continuous flow preparation of 2-chloro-1,3,2-dioxaphospholane **2a** (mesofluidic scale) 16

2.4.4 Concatenated semi-continuous flow preparation of cyclic phosphate monomers **EEP** and **BEP** (3-step process) 17
2.4.5 Semi-continuous flow preparation of 4-(chloromethyl)-2-ethoxy-1,3,2-dioxaphospholane 2-oxide (**EClMEP**, 2-step process). 17
2.4.6 Continuous flow homopolymerization toward **TEG-PEEP** 18
2.4.7 Continuous flow copolymerization toward **MeO-PEO-PEEP** with **MeO-PEO-OH** as a macroinitiator. 18
2.5 Additional experimental data 19
2.5.1 In-line monitoring of the continuous flow preparation of 2-chloro-1,3,2-dioxaphospholane (**2a**) by ^{31}P NMR 19
2.5.2 Base-involving procedure for the continuous flow synthesis of **2i-m** 20
2.5.3 Optimization of the continuous flow synthesis of 2-chloro-1,3,2-dioxaphospholane 2-oxide (**3a**) 20
2.5.4 Identification of a minor *H*-phosphonate impurity in cyclic phosphate monomer samples ...22
2.5.5 Semi-continuous flow synthesis (2-step) of 2-ethoxy-1,3,2-dioxaphospholane 2-oxide (**EEP**) and 2-butoxy-1,3,2-dioxaphospholane 2-oxide (**BEP**) 22
2.5.6 Batch procedure for the synthesis of 2-chloro-4-(chloromethyl)-1,3,2-dioxaphospholane (**2g**) 23
2.5.7 Batch procedure for the synthesis of 2-chloro-4-(chloromethyl)-1,3,2-dioxaphospholane 2-oxide (**3g**) 23
2.5.8 Batch procedure for the synthesis of 4-(chloromethyl)-2-ethoxy-1,3,2-dioxaphospholane 2-oxide (**EClMEP**) 24
2.5.9 Batch homopolymerization of **EEP** toward **TEG-PEEP** 24
2.5.10 Batch homopolymerization of **EEP** toward **BzO-PEEP** 24
2.5.11 Batch copolymerization of **EEP** with **EClMEP** toward **PEEP-*co*-PEClMEP** 25
2.5.12 Cytotoxicity assays 25
2.6 Characterization of compounds 27
2.7 Copies of NMR and IR spectra 31
2.8 Characterization of polymeric materials 69
2.8.1 **TEG-PEEP** (batch process) 69
2.8.2 **TEG-PEEP** (flow process, 10 min of residence time) 71
2.8.3 **TEG-PEEP** (flow process, 20 min of residence time) 74
2.8.4 **MeO-PEO-*b*-PEEP** (flow process) 76
2.8.5 **BzO-PEEP** (batch process) 79
2.8.6 **PEEP-*co*-PEClMEP** (**EEP/EClMEP**, 75:25) 81
2.8.7 **PEEP-*co*-PEClMEP** (**EEP/EClMEP**, 50:50) 84
3 References 87

Continuous flow setups

1.1 Microfluidic setups and parts

All microfluidic setups were assembled with commercially available parts.

1.1.1 Pumps

Chemyx Fusion 6000 High Force syringe pumps equipped with stainless steel syringes (6 or 20 mL) with Dupont Kalrez Spectrum AS-568 O-rings (0.549 x 0.103”) or Braun Luer Lock plastic syringe (5 mL, for polymerization experiments) were utilized to handle the liquid feeds.

1.1.2 Mass flow controller

A Bronkhorst® EL-FLOW® Prestige mass flow controller was utilized to handle the oxygen feed.

1.1.3 PFA tubing and coils

PFA coil reactors and collection lines were constructed from PFA tubing (high purity PFA; 1.58 mm outer diameter, 750 µm internal diameter or 3.18 mm outer diameter, 0.76 mm wall thickness).

1.1.4 Connectors, ferrules and mixers

PEEK connectors, ferrules, static mixers and unions were purchased from IDEX/Upchurch (details in Table S1).

1.1.5 Check-valves

The check-valves inserted between the pumps and the reactors were purchased from IDEX/Upchurch Scientific (PEEK check-valve holder).

1.1.6 Back-pressure regulators

Dome-type BPRs were purchased from Zaiput Flow Technologies (BPR-10). The dome-type BPR was connected to a compressed gas cylinder (nitrogen) to set the working pressure.

1.1.7 Thermoregulatory devices

PFA coils reactors were thermoregulated in oil or water baths (Heidolph MR Hei-Tec equipped with Pt-1000 temperature sensors).

1.2 Mesofluidic scale setup

1.2.1 Pumps

Asia syringe pumps (Syrris) equipped with Asia Red Syringes (2.5 mL / 5 mL) or Chemyx Fusion 6000 High Force syringe pumps equipped with stainless steel syringes (6 or 20 mL) with Dupont Kalrez Spectrum AS-568 O-rings (0.549 x 0.103”) were utilized to handle the liquid feeds.

1.2.2 Mesofluidic reactor

The mesofluidic reactor was manufactured by Corning SAS (Corning® Advanced-Flow™ LF/G1 skid Reactor) and equipped with 2 fluidic modules connected in series (Lab Reactor glass fluidic modules: 2.7 mL internal volume). See section 1.4.4 for detailed configuration.

1.2.3 Thermoregulatory devices

The reactor was maintained at reaction temperature with a Huber Ministat thermostat (THERM 180 thermofluid).

1.2.4 Back-pressure regulators

A dome-type BPR from Zaiput Flow Technologies (BPR-10) connected to a compressed gas cylinder (nitrogen) was utilized to set the working pressure.

1.3 Part numbers & vendors

Standard fluidic elements and connectors were purchased from IDEX/Upchurch Scientific, Valco Instruments Co. Inc, Swagelok and Zaiput Flow Technologies (Table S1).

Table S1. Connectors, ferrules and unions.

Item	Details	Vendor	Reference
Connectors	One-Piece Fingertight, PEEK, 10-32 Coned, for 1/16” OD	IDEX/ Upchurch Scientific	F-120X
	Super Flangeless Nuts, natural PEEK 1/4-28 thread for 1/16” OD tubing	IDEX/ Upchurch Scientific	P-255X
	Super Flangeless Ferrule Tefzel (ETFE) and SS ring 1/4-28 thread for 1/16” OD tubing	IDEX/ Upchurch Scientific	P-259X
Unions	Natural polypropylene standard low pressure union 1/4-28	IDEX/ Upchurch Scientific	P-620
	Union Assembly, PEEK, 0.02" through hole, for 1/16" OD"	IDEX/ Upchurch Scientific	P-702
Mixers	T-mixer, natural PEEK 1/4-28 thread for 1/16" o.d. tubing, 0.02" through hole	IDEX/ Upchurch Scientific	P-712
	High Pressure Static Mixing Tee (arrowhead), PEEK, 10-32 Coned thread for 1/16" o.d. tubing, 0.02" through hole,	IDEX/ Upchurch Scientific	U-466

	embedded with a UHMWPE Frit		
Check-valve	Check-valve inline cartridge 1.5 psi	IDEX/ Upchurch Scientific	CV-3001
Dome-type BPR	Dome-type BPR, metal-free, with adjustable set point	Zaiput Flow Techn.	BPR-10
Tubing	High-purity PFA tubing, 1.58 mm outer diameter, 750 µm internal diameter	VICI (Valco Ins. Co. Inc.)	JR-T-4002-M25
	High-purity 1/8” and 1/4” PFA tubing, including appropriate PFA connections	Swagelok	PFA-T2-030-100 PFA-T4-047-100

1.4 Detailed continuous flow setups

1.4.1 Continuous flow setup for the preparation of cyclic chlorophosphites and derivatives **2a-m**

See manuscript for experimental details (Table 1, entry 10) for derivatives **2a-h** and section 2.5.2 for derivatives **2i-m**.

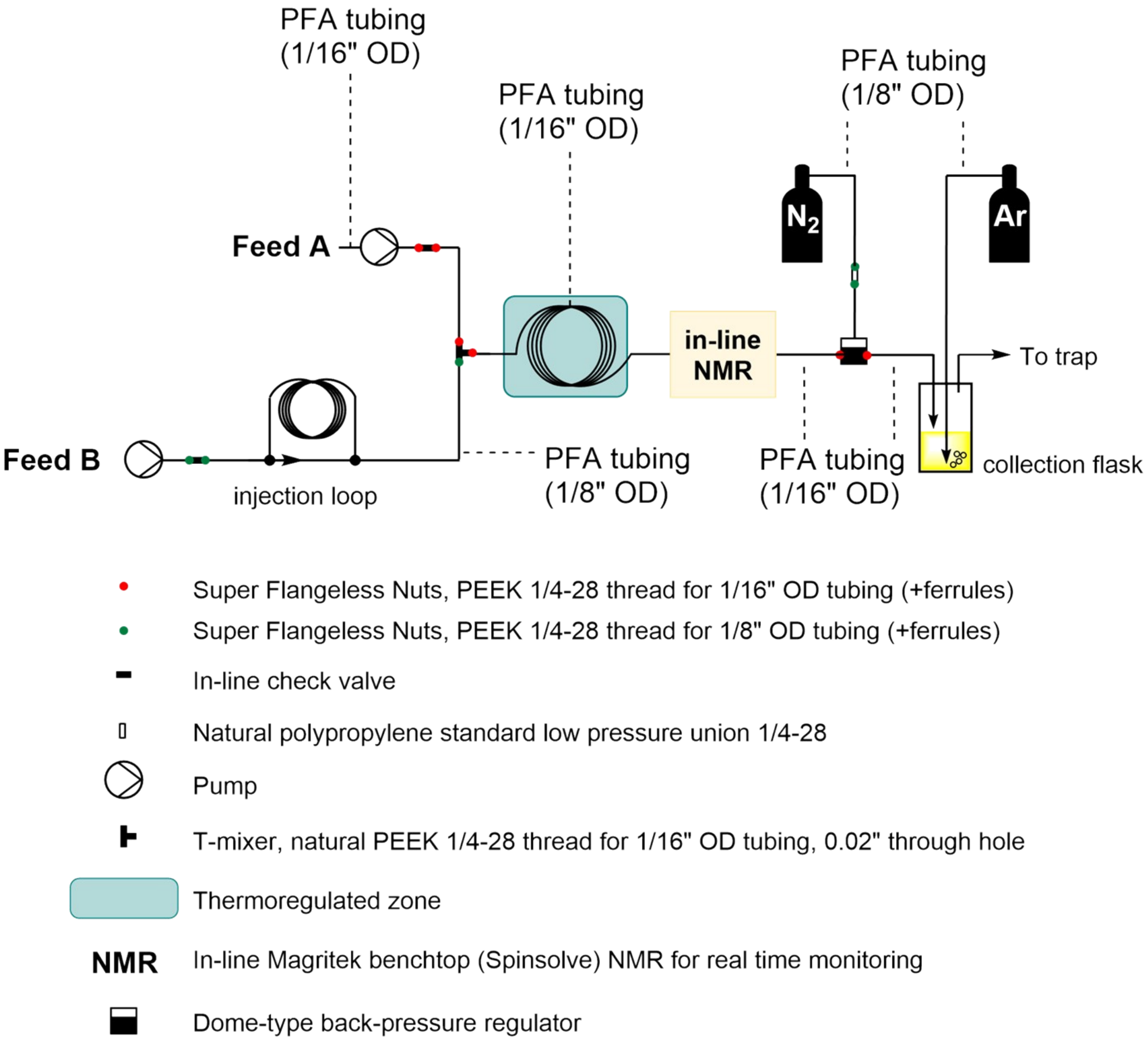

Figure S1. Detailed setup for the continuous flow preparation of **2a-m** adducts.

1.4.2 (Semi-)continuous flow setup for the preparation of cyclic chlorophosphate **3a** and **EEP** and **BEP** monomers

See section 2.4.4 for experimental details for **EEP** and **BEP** monomers and section 2.5.3 for derivative **3a**.

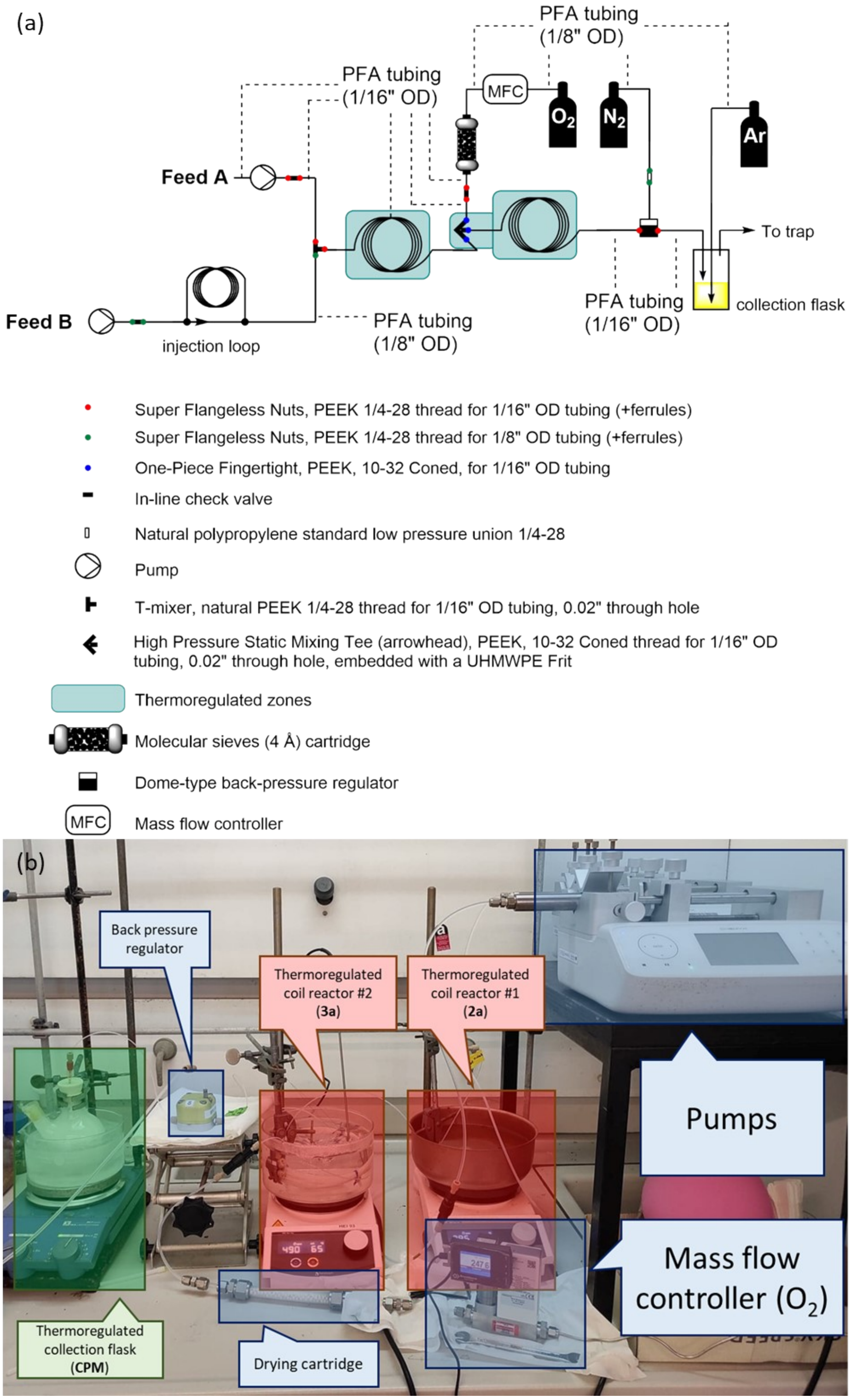

Figure S2. (a) Detailed setup for the (semi-)continuous flow synthesis of **3a** and **EEP** and **BEP** monomers and (b) labeled picture of the continuous flow system.

1.4.3 Semi-continuous flow setup for the preparation of 4-(chloromethyl)-2-ethoxy-1,3,2-dioxaphospholane 2-oxide (**EClMEP**)

See section 2.4.5 for experimental details.

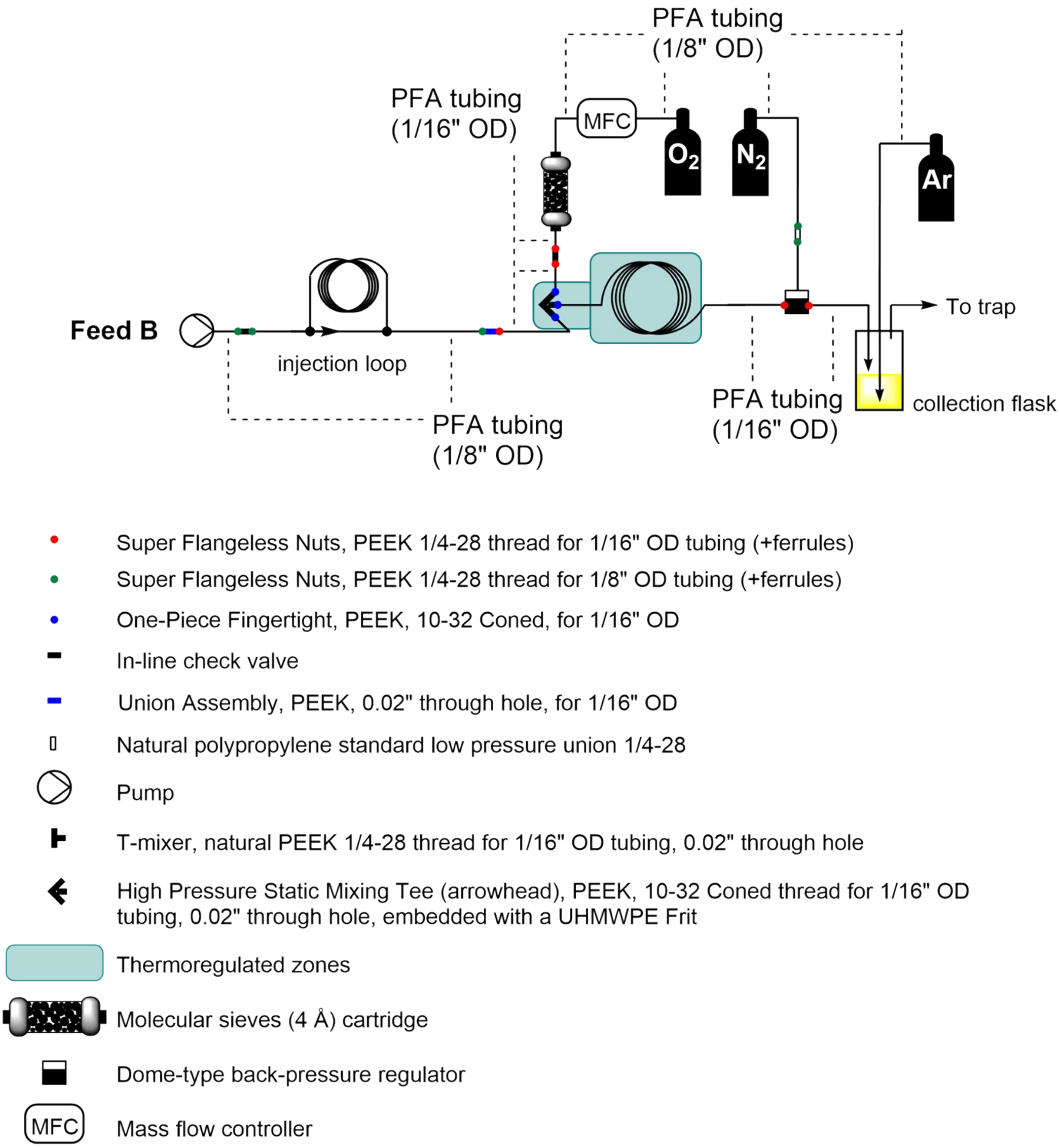

Figure S3. Detailed setup for the semi-continuous flow synthesis of 4-(chloromethyl)-2-ethoxy-1,3,2-dioxaphospholane 2-oxide (**EClMEP**).

1.4.4 Synthesis of 2-chloro-1,3,2-dioxaphospholane (**2a**, mesofluidic scale)

See section 2.4.3 for experimental details.

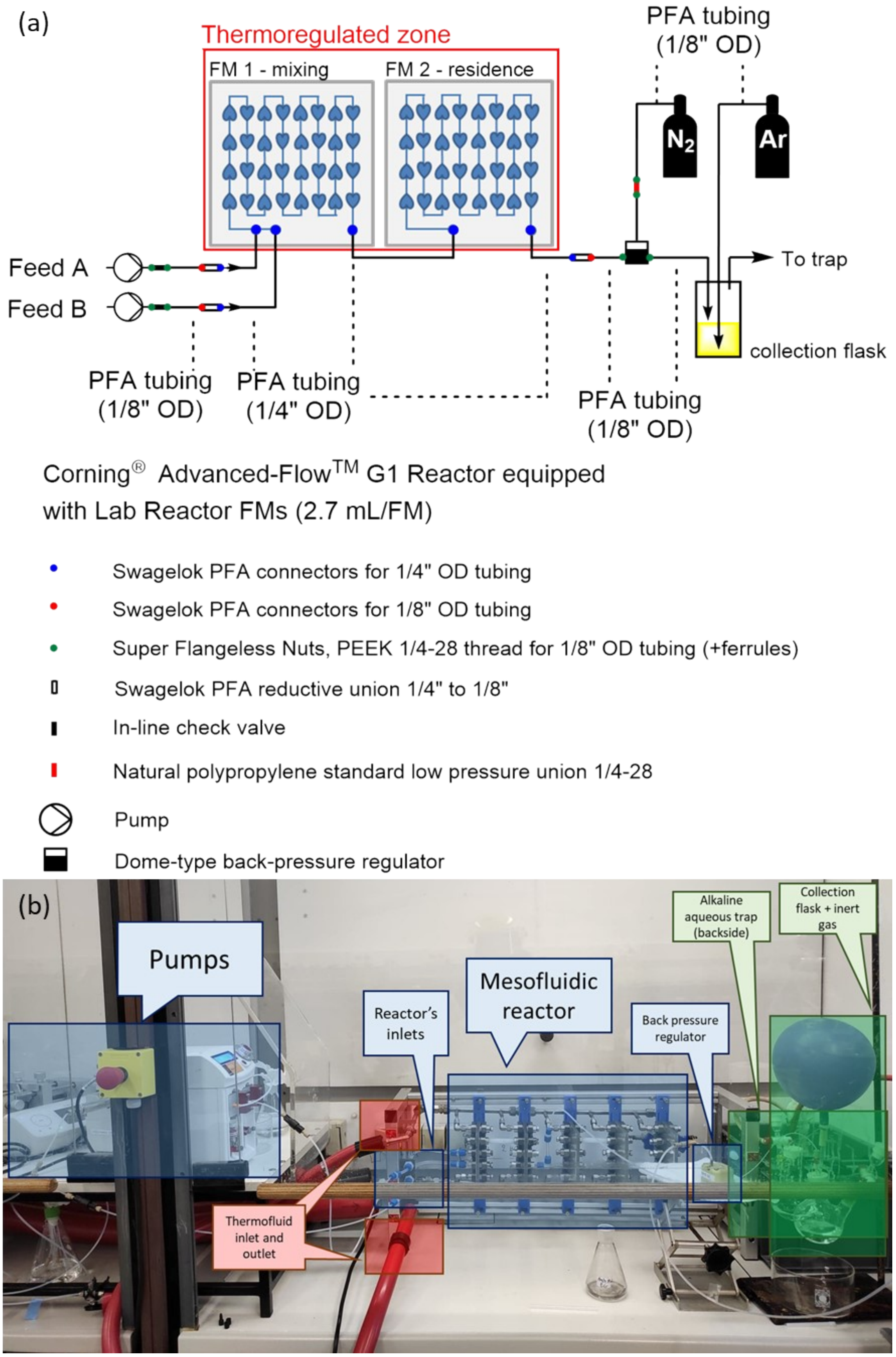

Figure S4. (a) Detailed setup for the mesofluidic scale preparation of 2-chloro-1,3,2-dioxaphospholane (**2a**) and (b) labeled picture of the continuous flow system.

1.4.5 Continuous flow polymerization toward **TEG-PEEP** and **MeO-PEO-*b*-PEEP**

See section 2.4.6 and 2.4.7 for experimental details.

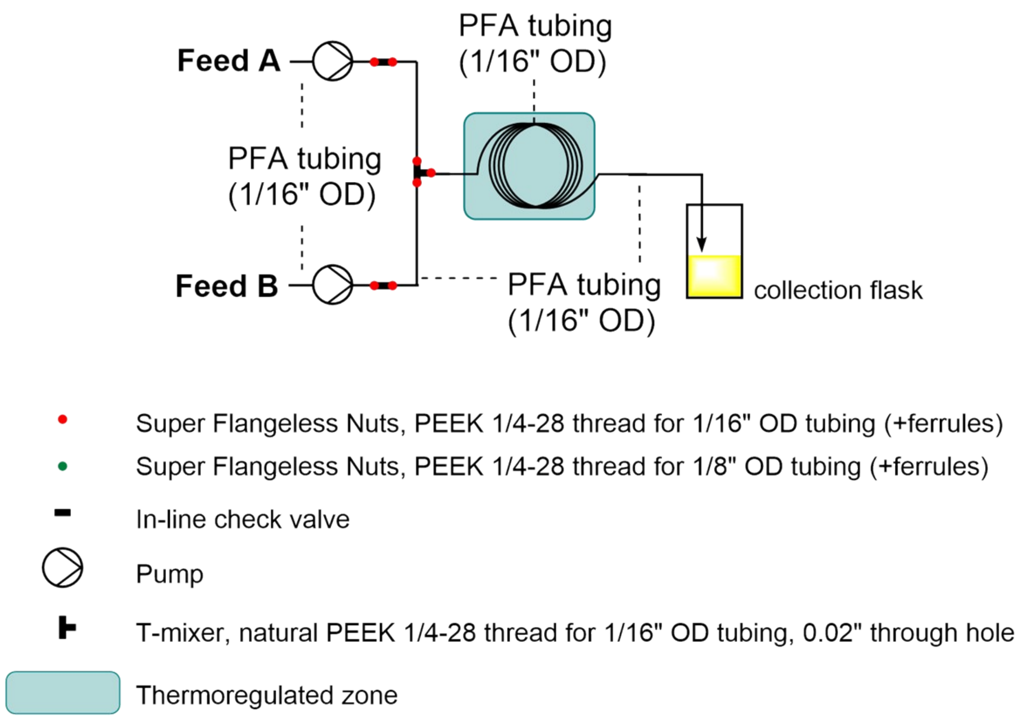

Figure S5. Detailed setup for the continuous flow preparation of **TEG-PEEP** and **MeO-PEO-*b*-PEEP**.

2. Additional experimental details

2.1 General information

Structural identity was confirmed by ^1H, ^{13}C and ^{31}P NMR spectroscopy (400 MHz Bruker Avance spectrometer) in $CDCl_3$ (see sections 2.6-2.8). The chemical shifts are reported in ppm relative to TMS as an internal standard or to the solvent residual peak for ^1H and ^{13}C NMR. The size exclusion chromatography (SEC) was carried out in DMF (flow rate = 1 mL min^{-1}) at 40 °C using a Water 600 auto sampler liquid chromatograph equipped with a differential refractometer index detector. Waters gel 5 µm (105, 104, 500, and 100 Å) columns were calibrated with polystyrene standards for **PEEP** homopolymers and **PEEP-*co*-PEClMEP** copolymers and in THF at 45°C with a throughput of 1 mL min^{-1} on a Viscotek 305 TDA liquid chromatograph equipped with two PSS SDV linear M columns calibrated with polystyrene standards for **MEO-PEO-*b*-PEEP** block copolymers. Thermogravimetric analysis (TGA) was performed using a TGA 2 (Mettler Toledo) equipment. A sample of 5 mg of polymer was transferred in a pan and a heating ramp of 10°C min^{-1} was then applied from 25 °C to 600 °C under nitrogen atmosphere to record the weight lost. Differential scanning calorimetry (DSC) was performed using a DSC 250 (TA Instruments) equipped with a RSC 90 cooling system and calibrated with indium. An amount of around 5 mg of the (co)polymer was transferred in an aluminum capsule. Two consecutive heating ramps (10°C min^{-1}) were applied to the sample between - 80°C and 100 °C. The glass transition temperature (T_g), the melting temperature (T_m) and the melting enthalpy (ΔH_m) were recorded during the second heating ramp. Solvents (dichloromethane, methyl *tert*-butylether, acetonitrile, tetrahydrofuran, 2-methyltetrahydrofuran) were dried over molecular sieves prior to use. Ethylene glycol, propylene glycol, 1,2-butanediol, 2,3-butanediol, but-3-ene-1,2-diol, 3-methoxy-1,2-propanediol, 1,2-ethanedithiol, pinacol, 1,3-propanediol, 2-mercaptoethanol, 2-(methylamino-)ethanol, N,N′-dimethylethylenediamine, PCl_3, 1-methylimidazole, tetraethylene glycol, diethyl ether and poly(ethylene oxide monomethylether) (M_W = 5 Kg mol^{-1}), were obtained from commercial sources and used as received (see section 2.2). 3-Chloropropane-1,2-diol, ethanol (absolute), *n*-butanol, pyridine, triethylamine, benzyl alcohol and 1,8-diazabicyclo[5.4.0]undec-7-ene (DBU) were obtained from commercial sources (see section 2.2) and purified by distillation and dried prior to use. 1-[3,5-bis(trifluoromethyl)phenyl]-3-cyclohexylthiourea was synthetized according to a method described previously.[1] Oxygen (Alphagaz 1) was obtained from a commercial source (Air Liquide) and used as received.

2.2 Chemicals

Chemicals, purity, CAS numbers and suppliers are provided in Table S2.

Table S2. Solvents, chemicals and suppliers.

Solvents	Purity (%)	CAS Number	Supplier
THF	99.8	109-99-9	Fisher
MeTHF	≥99.5	96-47-9	Sigma-Aldrich
MTBE	≥99	1634-04-4	VWR
Acetonitrile	≥99.9	75-05-8	Fisher
Dichloromethane	≥99.8	75-09-2	Fisher
Diethyl ether	Tech.	60-29-7	VWR
DMF	≥99.9	68-12-2	VWR
**Chemicals**	**Purity (%)**	**CAS number**	**Supplier**
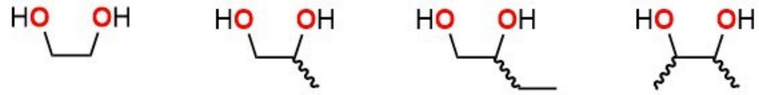 **1a** CAS 107-21-1; **1b** CAS 57-55-6; **1c** CAS 584-03-2; **1d** CAS 513-85-9			
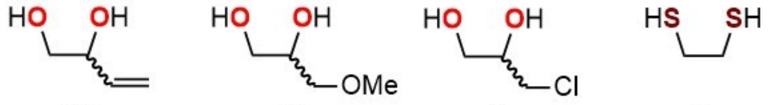 **1e** CAS 497-06-3; **1f** CAS 623-39-2; **1g** CAS 96-24-2; **1h** 540-63-6			
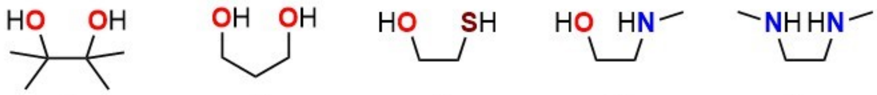 **1i** CAS 76-09-5; **1j** CAS 505-63-2; **1k** CAS 60-24-2; **1l** CAS 109-83-1; **1m** CAS 110-70-3			
Ethanol (absolute)	>99	64-17-5	VWR
n-Butanol	99.5	71-36-3	Biosolve
Ethylene glycol	99.8	107-21-1	Acros Organics
Propylene glycol	≥99	57-55-6	TCI
1,2-Butanediol	>98	584-03-2	TCI
2,3-Butanediol	>97	513-85-9	TCI

3,4-Dihydroxy-1-butene	≥99	497-06-3	Sigma-Aldrich
3-Methoxy-1,2-propanediol	>98	623-39-2	TCI
(±)-3-Chloro-1,2-propanediol	>98	96-24-2	TCI
1,3-Propanediol	99	505-63-2	Alfa Aesar
Pinacol	>98	76-09-5	TCI
2-Mercaptoethanol	99	60-24-2	Acros Organics
1,2-Ethanedithiol	≥98	540-63-6	Sigma Aldrich
2-(Methylamino)ethanol	>99	109-83-1	TCI
N,N′-Dimethylethylenediamine	>97	110-70-3	TCI
Phosphorus trichloride	99	7719-12-2	Acros Organics
1-Methylimidazole	99	616-47-7	abcr
1,8-Diazabicyclo[5.4.0]undec-7-ene (DBU)	>98	6674-22-2	TCI
Triethylamine	≥99	121-44-8	VWR
Pyridine	>99	110-86-1	VWR
Tetraethylene glycol	99	112-607	Aldrich
Poly(ethylene oxide monomethylether) (5 kg mol^{-1})		9004-74-4	Aldrich
Benzyl alcohol	>99	100-51-6	TCI

2.3 **Experimental setup**

2.3.1 Microfluidic setups

Microfluidic reactors consisted of modular continuous flow assemblies constructed from PFA tubing (1.58 mm outer diameter, 0.750 mm internal diameter) equipped with PEEK/ETFE connectors and ferrules (IDEX/ Upchurch Scientific). Feed and collection lines consisted of PFA or PEEK tubing (1.58 mm outer diameter, 0.750 mm internal diameter) equipped with PEEK/ETFE connectors and ferrules (IDEX/Upchurch Scientific). Liquid feeds were handled with high force Chemyx Fusion 6000 syringe pumps equipped with SS syringes and DuPont Kalrez O-rings. Gas feeds were handled with a Bronkhorst EL-FLOW Prestige mass flow controller. The coil reactors were thermoregulated with a Heidolph MR Hei-Tec equipped with a Pt-1000 temperature sensor. Downstream pressure was regulated with back pressure regulators from Zaiput Flow Technologies (BPR-10) or from IDEX/Upchurch Scientific.

2.3.2 Mesofluidic setup

Mesofluidic experiments were carried out in a Corning® Advanced-Flow™ G1 Reactor embedded with 2 Lab Reactor glass fluidic modules (5.4 mL total internal volume). Feed and collection lines consisted of PFA tubing (1/8″ or 1/4″ o.d.) equipped with PFA Swagelok connectors and ferrules. Liquid feeds were handled with Asia syringe pumps (Syrris) equipped with Asia Red Syringes (2.5 mL / 5 mL) or Chemyx Fusion 6000 High Force syringe pumps equipped with SS syringes and DuPont Kalrez O-rings. The reactor was maintained at reaction temperature with a Huber Ministat thermostat (THERM 180 thermofluid). Downstream pressure was regulated with a back pressure regulator from Zaiput Flow Technologies (BPR-10).

2.4 **Typical runs**

2.4.1 General continuous flow protocol for the preparation of cyclic chlorophosphites and derivatives **2a-h** (base-free procedures)

Caution! *Purification by distillation under reduced pressure of cyclic chlorophosphites and derivatives* ***2a-m*** *must be handled with appropriate care to avoid uncontrolled decomposition reactions (in particular for derivative* ***2e****). Avoiding overheating is strongly advised during the distillation process, color change of the crude mixture from yellow to orange/red indicates significant risks of uncontrolled decomposition.*

See also section 1.4.1

The pump used to deliver neat diol or dithiol **1a-h** (1 equiv.) and PCl_3 (3 M in anhydrous 2-methyltetrahydrofuran, 1 equiv., loaded in a PFA injection loop) were set according to each target as following: 143.2 µL min^{-1} for **1a** and 856.8 µL min^{-1} for the PCl_3 solution toward **2a**; 180.6 µL min^{-1} for **1b** and 819.4 µL min^{-1} for the PCl_3 solution toward **2b**; 212.4 µL min^{-1} for **1c** and 787.6 µL min^{-1} for the PCl_3 solution toward **2c**; 205.5 µL min^{-1} for **1d** and 794.5 µL min^{-1} for the PCl_3 solution toward **2d**; 201.6 µL min^{-1} for **1e** and 798.4 µL min^{-1} for the PCl_3 solution toward **2e**; 222.3 µL min^{-1} for **1f** and 777.7 µL min^{-1} for the PCl_3 solution toward **2f**; 200.8 µL min^{-1} for **1g** and 799.2 µL min^{-1} for the PCl_3 solution toward **2g**; 201.5 µL min^{-1} for **1h** and 798.5 µL min^{-1} for the PCl_3 solution toward **2h**. Both streams were mixed through a PEEK T-mixer and the resulting mixture was reacted in a PFA capillary coil (1 mL internal volume, 1 min residence time) at room temperature under 5 bar of counterpressure. The reactor effluent was collected in a closed vessel under an inert atmosphere (Ar) and connected to an alkaline aqueous trap. In-line analysis was optionally performed with a benchtop NMR (17.4 MHz Spinsolve™ ^{31}P NMR spectrometer from Magritek® equipped with a flow-through cell). The reactor effluent was concentrated under reduced pressure and purified by a vacuum distillation. Isolated yields: starting from ethylene glycol **1a** (**2a**, 5.8 g, 24 min of collection time, 74% yield), propylene glycol **1b** (**2b**, 8.9 g, 34 min of collection time, 76% yield), 1,2-butanediol **1c** (**2c**, 10.9 g, 35 min of collection time, 85% yield), 2,3-butanediol **1d** (**2d**, 8.6 g, 34 min of collection time, 68% yield), but-3-ene-1,2-diol **1e** (**2e**, 4.8 g, 29.5 min of collection time, 44% yield), 3-methoxy-1,2-propanediol **1f** (**2f**, 11.2 g, 34 min of collection time, 83% yield), 3-chloropropane-1,2-diol **1g** (**2g**, 10.2 g, 36 min of collection time, 67% yield) and 1,2-ethanedithiol **1h** (**2h**, 8.0 g, 36 min of collection time, 58% yield).

2.4.2 General continuous flow protocol for the preparation of cyclic chlorophosphites and derivatives **2i**-**2m** (base-involving procedures)

Caution! *Purification by distillation under reduced pressure of cyclic chlorophosphites and derivatives* ***2a-m*** *must be handled with appropriate care to avoid uncontrolled decomposition reactions (in particular for derivative* ***2e****). Avoiding overheating is strongly advised during the distillation process, color change of the crude mixture from yellow to orange/red indicates significant risks of uncontrolled decomposition.*

See also section 1.4.1 and 2.5.2

The pump used to deliver diol or derivative **1i**-**1m** (2 M when employing **1i**-**1l** or 1 M when employing **1m** in anhydrous acetonitrile, 1 equiv.) and an organic base (2 equiv. of 1-methylimidazole when employing **1i**-**1k**, 2.2 equiv. when employing **1l** and 2 equiv. of 1,8-diazabicyclo[5.4.0]undec-7-ene (DBU) when employing **1m**) was set to 500 µL min^{-1}. The pump used to deliver PCl_3 (2 M when preparing **2i**-**2l** or 1 M when preparing **2m** in anhydrous acetonitrile, 1 equiv., loaded in a PFA injection loop) was set to 500 µL min^{-1}. Both streams were mixed through a PEEK T-mixer and the resulting mixture was reacted in a PFA capillary coil (1 mL internal volume, 1 min residence time) at room temperature under 5 bar of counterpressure, when preparing **2k** the T-mixer and the coil reactor were immersed in an ultrasound bath. The reactor effluent was collected in a closed vessel under inert atmosphere (Ar) and connected to an alkaline aqueous trap. In-line analysis was optionally performed with a benchtop NMR (17.4 MHz Spinsolve™ ^{31}P NMR spectrometer from Magritek® equipped with a flow-through cell). The reactor effluent was concentrated under reduced pressure and purified by a fractional vacuum distillation. Isolated yields: starting from pinacol **1i** (**2i**, 5.8 g, 48 min of collection time, 66% yield), 1,3-propanediol **1j** (**2j**, 3.7 g, 49 min of collection time, 53% yield), 2-mercaptoethanol **1k** (**2k**, 1.8 g, 26.5 min of collection time, 46% yield), 2-(methylamino)ethanol **1l** (**2l**, 5.9 g, 51 min of collection, 83% yield) and N,N'-dimethylethylenediamine **1m** (**2m**, 1.6 g, 49 min of collection, 43% yield).

2.4.3 Continuous flow preparation of 2-chloro-1,3,2-dioxaphospholane **2a** (mesofluidic scale)

See also section 1.4.4

The two pumps used to deliver neat ethylene glycol (**1a**) were set to 386.6 µL min^{-1} (773.2 µL min^{-1} of total **1a** flow rate). The pump used to deliver the 3 M PCl_3 solution in 2-methyltetrahydrofuran was set to 4.63 mL min^{-1}. Both streams were mixed and reacted in a Corning® Advanced-Flow™ G1 Reactor equipped with two Lab Reactor fluidic modules (5.4 mL of internal volume) operated at 23 °C under 4 bar of counterpressure with an associated residence time of 1 min. The reactor effluent was collected in a closed vessel under an inert atmosphere (Ar) and connected to an alkaline aqueous trap placed in an ice/water bath. The reactor effluent was concentrated under reduced pressure and purified by a vacuum fractional distillation. 2-Chloro-1,3,2-dioxaphospholane (**2a**) was isolated as a colorless liquid in 74% yield with a daily productivity value of 1.88 kg.

2.4.4 Concatenated semi-continuous flow preparation of cyclic phosphate monomers **EEP** and **BEP** (3-step process)

See also section 1.4.2 and Figure 3 in the manuscript.

The pump used to deliver neat ethylene glycol (**1a**, 1 equiv.) was set to 143.2 µL min^{-1} of flow rate. The pump used to deliver PCl_3 (3 M in anhydrous 2-methyltetrahydrofuran, 1 equiv., loaded in a PFA injection loop) was set to 856.8 µL min^{-1}. Both streams were mixed through a PEEK T-mixer and the resulting mixture was reacted in a PFA capillary coil (1 mL internal volume, 1 min residence time) at room temperature. The reactor effluent was subsequently mixed with oxygen (247.4 mL_n min^{-1}, 4 equiv.) through an arrowhead-type mixer embedded with a UHMWPE frit and reacted in a PFA capillary coil (7 mL of internal volume, 21 s of estimated residence time) at 65 °C under 15 bar of counterpressure. The reactor effluent was collected in a closed vessel under stirring containing 1 equiv. of an appropriate anhydrous alcohol (77.1 mmol of ethanol for **EEP** or *n*-butanol for **BEP**, for 30 min of total collection time) and 3 equiv. of anhydrous pyridine (231.3 mmol for 30 min of total collection time) in 2-methyltetrahydrofuran (60 mL for 30 min of total collection time) at 0 °C under an inert atmosphere (Ar) and connected to an alkaline aqueous trap. After collection the mixture was allowed to react at room temperature for two additional hours and was subsequently filtered, concentrated under reduced pressure and purified by a vacuum fractional distillation. **EEP** (4.41 g, 39% yield) and **BEP** (6.79 g, 49% yield) were isolated as colorless liquids.

2.4.5 Semi-continuous flow preparation of 4-(chloromethyl)-2-ethoxy-1,3,2-dioxaphospholane 2-oxide (**EClMEP**, 2-step process).

See also section 1.4.3 and Figure S6.

The pump used to deliver **2g** (2.4 M in anhydrous 2-methyltetrahydrofuran, 1 equiv., loaded in a PFA injection loop) was set to 1 mL min^{-1}. The mass flow controller used to deliver oxygen (4.2 equiv.) was set to 247.4 mL_n min^{-1}. The liquid and the gas streams were both mixed through an arrowhead-type mixer embedded with a UHMWPE frit and reacted in a PFA capillary coil (7 mL of internal volume, 21 s of residence time) at 65 °C under 15 bar of counterpressure. The reactor effluent was collected in a closed vessel under stirring containing 1 equiv. of ethanol (79.1 mmol for 30 min of total collection time) and 1 equiv. of pyridine (79.1 mmol for 30 min of total collection time) in 2-methyltetrahydrofuran (60 mL for 30 min of total collection time) at 0 °C under an inert atmosphere (Ar) and connected to an alkaline aqueous trap. After collection the mixture was allowed to react at room temperature for two additional hours and was subsequently filtered, concentrated under reduced pressure and purified by a vacuum fractional distillation affording **EClMEP** as a colorless liquid (10.12 g, 70% yield).

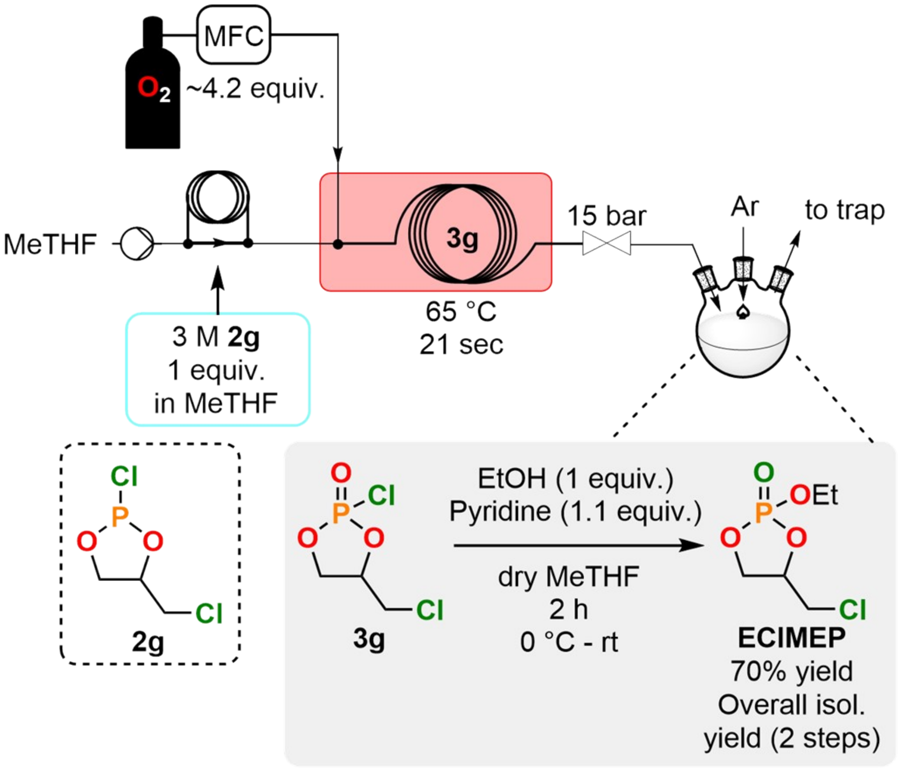

Figure S6. Two-step semi-continuous flow synthesis of monomer **EClMEP**.

2.4.6 Continuous flow homopolymerization toward **TEG-PEEP**

See also section 1.4.5 and Table 2 in the manuscript.

The pump used to deliver **EEP** (1.10 M, 80 equiv.) in anhydrous CH_2Cl_2 was set to 26.8 µL min^{-1} (10 min of res. time) or to 13.4 µL min^{-1} (20 min of res. time). The pump used to deliver a solution containing tetraethylene glycol (15.8 mM, 1 equiv.), 1,8-diazabicyclo[5.4.0]undec-7-ene (DBU, 0.25 M, 15.8 equiv.) and 1-[3,5-bis(trifluoromethyl)phenyl]-3-cyclohexylthiourea (77.4 mM, 4.6 equiv.) in anhydrous CH_2Cl_2 was set to 23.2 µL min^{-1} (10 min of res. time) or to 11.6 µL min^{-1} (20 min of res. time). Both streams were mixed through a PEEK T-mixer and the resulting mixture was reacted in a PFA capillary coil (0.5 mL of internal volume) at 0 °C under atmospheric pressure. The reactor effluent was directly collected in a cold diethyl ether solution to trigger the precipitation of the polymer. The ether solution was left at -20 °C overnight and the polymer was retrieved after decantation and dried under vacuum. For 10 min res. time: M_n (^{1}H NMR) = 2,700 g mol^{-1}, M_w/M_n (SEC) = 1.06. For 20 min res. time: M_n (^{1}H NMR) = 4,700 g mol^{-1}, M_w/M_n (SEC) = 1.11.

2.4.7 Continuous flow copolymerization toward **MeO-PEO-PEEP** with **MeO-PEO-OH** as a macroinitiator.

See also section 1.4.5 and Table 2 in the manuscript.

The pump used to deliver **EEP** (1.65 M, 13 equiv.) in anhydrous CH_2Cl_2 was set to 7.8 µL min^{-1}. The pump used to deliver a solution containing **MeO-PEO-OH** (59.1 mM, 1 equiv.), 1,8-diazabicyclo[5.4.0]undec-7-ene (DBU, 0.30 M, 5 equiv.) and 1-[3,5-

bis(trifluoromethyl)phenyl]-3-cyclohexylthiourea (0.14 M, 2.4 equiv.) in anhydrous CH_2Cl_2 was set to 17.2 µL min^{-1}. Both streams were mixed through a PEEK T-mixer and the resulting mixture was reacted in a PFA capillary coil (0.5 mL of internal volume, 20 min of res. time) at 0 °C under ambient pressure. The reactor effluent was directly collected in cold diethyl ether solution to trigger the precipitation of the polymer. The ether solution was left at -20 °C overnight and the polymer was retrieved after filtration and dried under vacuum. The catalysts were removed by solubilization of the copolymer in methanol followed by a dialysis against methanol (MWCO = 1 KDa) overnight. The purified copolymer was recovered by evaporation of the solvent under vacuum. For 20 min of residence time: M_n (^{1}H NMR) = 7,000 g mol^{-1}, M_w/M_n (SEC) = 1.05. Dialysis was performed prior to cytotoxicity assays (see section 2.5.12).

2.5 **Additional experimental data**

2.5.1 In-line monitoring of the continuous flow preparation of 2-chloro-1,3,2-dioxaphospholane (**2a**) by ^{31}P NMR

A Spinsolve™ ^{31}P NMR (17.4 MHz) spectrometer from Magritek® equipped with a flow-through cell was used for the real-time monitoring of the continuous flow synthesis of **2a** (see also Table 1 in the manuscript). Prior to the reaction, a shim was performed on the solvent (2-methyltetrahydrofuran). A ^{31}P NMR spectrum was recorded after each loop which consisted of 2 scans (4 s of repetition time, 90° of pulse angle) of a 285 ppm bandwidth centered at 90 ppm. During the monitoring, the PCl_3 solution was firstly injected prior to the ethylene glycol which triggered the progressive appearance of **2a** and the consumption of PCl_3 (Figure S7).

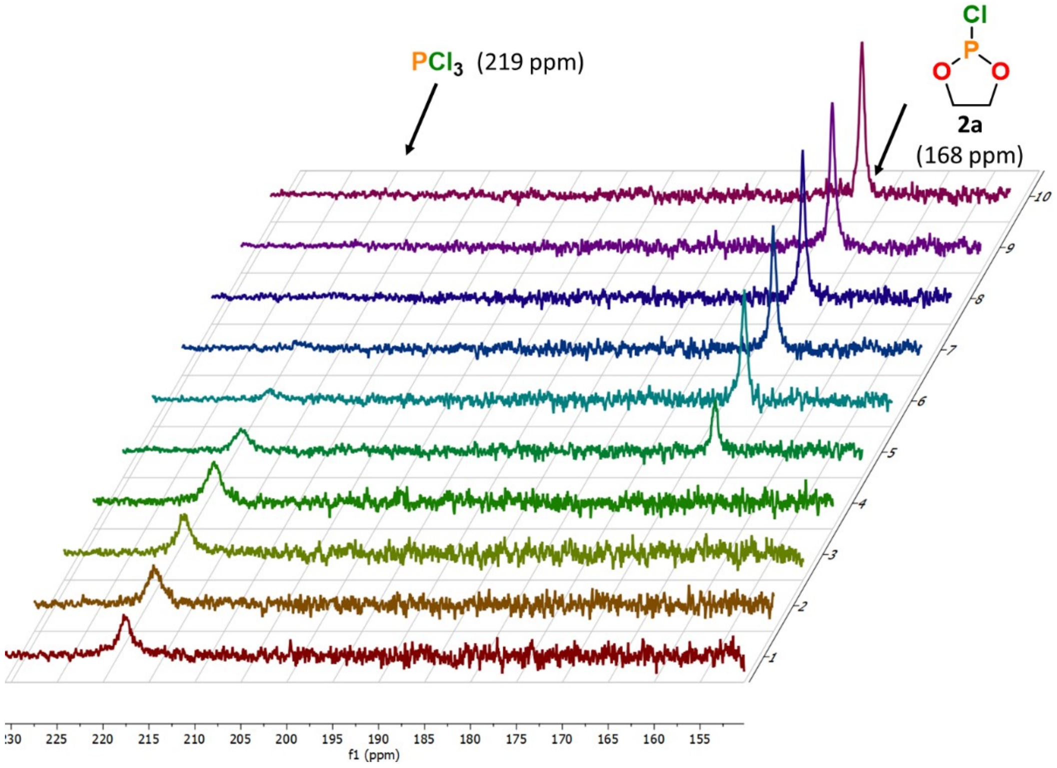

Figure S7. Stacked ^{31}P NMR spectra obtained during the inline monitoring of the continuous flow synthesis of **2a**.

2.5.2 Base-involving procedure for the continuous flow synthesis of **2i-m**

Starting diol, thio alcohol, amino alcohol and diamine derivatives **1i-m** were mixed with an appropriate base in dry acetonitrile prior to injection (Figure S8). The solution of PCl_3 diluted in dry acetonitrile was loaded in an injection loop prior to injection. Both streams were mixed through a T-mixer and reacted in a coil reactor under 5 bar of counterpressure at room temperature, in the case of **2k** the T-mixer and the coil reactor were immersed in an ultrasound bath. The reactor effluent was collected at steady state in a closed vessel under inert atmosphere (argon) that was connected to an alkaline aqueous trap. The reactor effluent was concentrated under reduced pressure and purified by a fractional vacuum distillation affording **2i-m** in 43 – 83% yield with an associated daily productivity of 48 – 175 g (see also section 2.4.2).

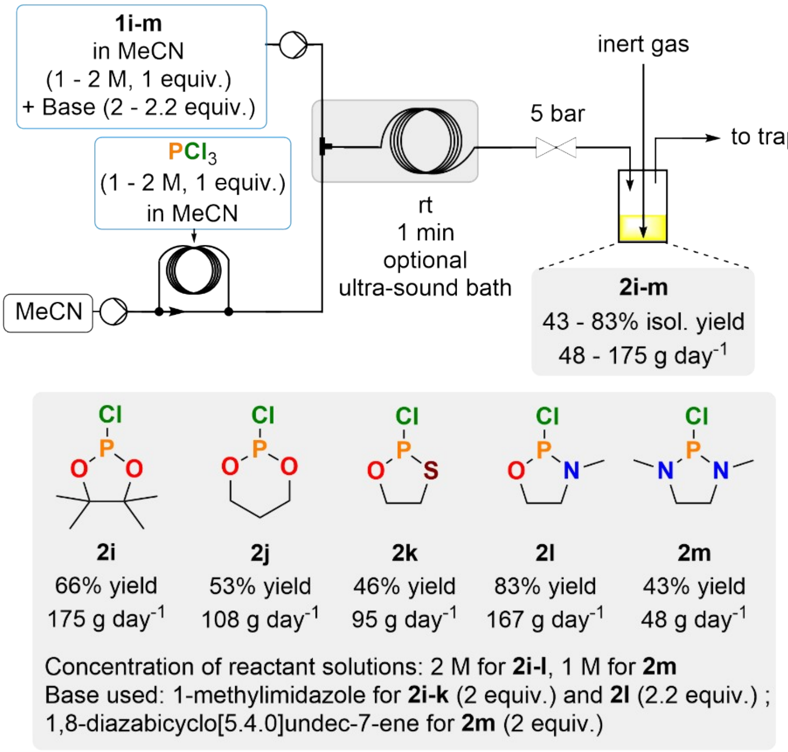

Figure S8. Base-involving continuous flow process used for the preparation of **2i-m** adducts

2.5.3 Optimization of the continuous flow synthesis of 2-chloro-1,3,2-dioxaphospholane 2-oxide (**3a**)

A concatenated system with the upstream preparation of **2a** and its subsequent oxidation toward **3a** was developed (Table S3). Samples of the effluent were collected at steady state and immediately analyzed by a benchtop ^{31}P NMR spectrometer for a qualitative measurement of the conversion of **2a** and selectivity toward **3a**. Preliminary experiments were performed using 1/8” OD tubing for the oxidation reactor (MFR#2, V_{int} = 18 mL) with an upstream T-mixer. Poor conversion values were obtained for temperatures ranging from room temperature to 45 °C (Table S3, entries 1-3); increasing the temperature to 50 °C led to a quantitative conversion of **2a** but the selectivity toward **3a** dropped substantially (18%, entry 4). To circumvent selectivity issues related to the oxidation of **2a** the design of the coil reactor was adapted to allow a maximization of the mixing efficiency rather than a longer residence

time. Switching to 1/16” OD tubing for the oxidation reactor (MFR#2, V_{int} = 7 mL) and using an arrowhead-type mixer embedded with a UHMWPE frit allowed to significantly increase the selectivity of the reaction even at higher temperatures (entries 5-9) while reducing the residence time. Working at 65°C with 4 equivalents of molecular oxygen allowed to obtain **3a** in 59% of selectivity with a quantitative conversion of **2a** with an associated residence time of 21 s (entry 8). A quick degradation of **3a** samples were noticed after collection potentially due to the presence of HCl by-product generated during the upstream synthesis of **2a**. The telescoped condensation of **3a** with various alcohol derivatives was next envisioned (see manuscript).

Table S3. Process optimization for the telescoped continuous flow synthesis of 2-chloro-1,3,2-dioxaphospholane 2-oxide (**3a**).[a]

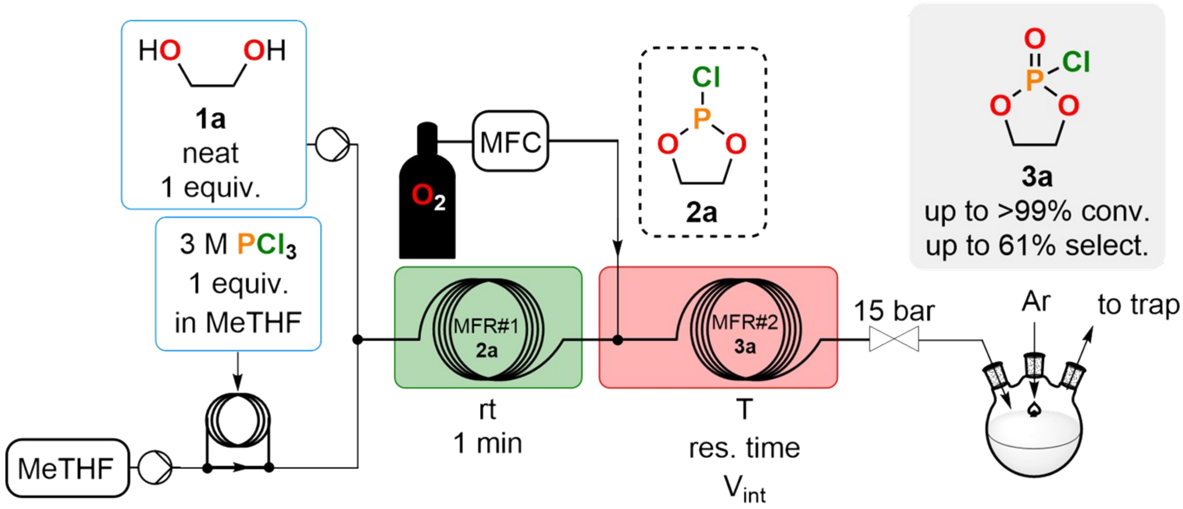

Entry	Temperature of MFR#2 (°C)	O_2 flow rate (mL_n min^{-1}) [O_2 equiv.]	Estimated res. time in MFR#2	Conversion of **2a** (%)[b]	Selectivity toward **3a** (%)[b]
1/8” OD tubing used for MFR#2 (V_{int} = 18 mL) + T-mixer					
1	Room temp.	123.7 [2.0]	1 min 55 s	6	12
2	40	100 [1.6]	2 min 11 s	25	57
3	45	100 [1.6]	2 min 9 s	26	50
4	50	100 [1.6]	2 min 7 s	>99	18
1/16” OD tubing used for MFR#2 (V_{int} = 7 mL) + arrowhead mixer					
5	40	123.7 [2.0]	51 s	13	61
6	40	247.4 [4.0]	22 s	22	55
7	60	247.4 [4.0]	21 s	90	55
8	65	247.4 [4.0]	21 s	>99	59
9	70	247.4 [4.0]	20 s	>99	41

[a]Typical conditions: P = 15 bar; neat **1a** flow rate = 143.2 µL min^{-1}, 2-methyltetrahydrofuran (MeTHF) flow rate = 856.8 µL min^{-1}, V_{int} of MFR#1 = 1 mL. [b]Conversion and selectivity values were determined by ^{31}P NMR (Magritek Spinsolve 43 MHz NMR spectrometer).

2.5.4 Identification of a minor *H*-phosphonate impurity in cyclic phosphate monomer samples

The telescoped semi-continuous flow procedure for the preparation of **EEP** and **BEP** monomers (see Figure 3 in the manuscript) led to the presence of a minor impurity in purified samples (detected by ^{31}P NMR). Comparison between ^{1}H-decoupled and coupled ^{31}P NMR spectra (Figure S9) highlighted a typical $J^{1}_{P\text{-}H}$ coupling constant of ~700 Hz corresponding to the signal of the impurity (centered at ~23 ppm). The structural identity of the impurity is most likely related to the compound **HP** which could be generated from **2a** in the presence of moisture.[2]

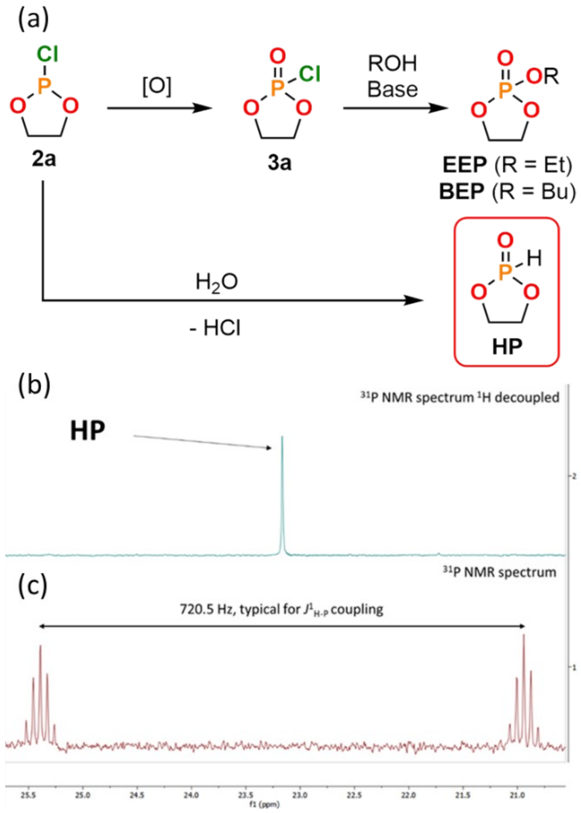

Figure S9. (a) Proposed chemical pathway leading to the presence of an *H*-phosphonate impurity (**HP**) during the production of **EEP** and **BEP** monomers, (b) ^{31}P {^{1}H} NMR spectrum centered on the *H*-phosphonate impurity and (c) ^{31}P NMR spectrum focused on the *H*-phosphonate impurity highlighting a $J^{1}_{P\text{-}H}$ coupling constant.

2.5.5 Semi-continuous flow synthesis (2-step) of 2-ethoxy-1,3,2-dioxaphospholane 2-oxide (**EEP**) and 2-butoxy-1,3,2-dioxaphospholane 2-oxide (**BEP**)

To produce high purity **EEP** and **BEP** monomers, a 2-step semi-continuous flow process starting from purified **2a** was designed (Figure S10). The pump used to deliver **2a** (2.57 M in anhydrous 2-methyltetrahydrofuran, 1 equiv., loaded in a PFA injection loop) was set to 1 mL min^{-1}. The stream was mixed with oxygen (247.4 mL_n min^{-1}, 4 equiv.) through an arrowhead-type mixer embedded with a UHMWPE frit and reacted in a PFA capillary coil (7 mL of internal volume, 21 s of residence time) at 65 °C under 15 bar of counterpressure. The reactor effluent was collected in a closed vessel under stirring containing 1 equiv. of an appropriate anhydrous

alcohol (77.1 mmol of ethanol for **EEP** or of *n*-butanol for **BEP** for 30 min of total collection time) and 1.1 equiv. of anhydrous pyridine (84.8 mmol for 30 min of total collection time) in 2-methyltetrahydrofuran (60 mL for 30 min of total collection time) at 0 °C under an inert atmosphere (Ar) and connected to an alkaline aqueous trap. After collection the mixture was allowed to react at room temperature for two additional hours and was subsequently filtered, concentrated under reduced pressure and purified by a vacuum fractional distillation. **EEP** (4.73 g, 40% overall yield) and **BEP** (5.55 g, 40% overall yield) were isolated as colorless liquids.

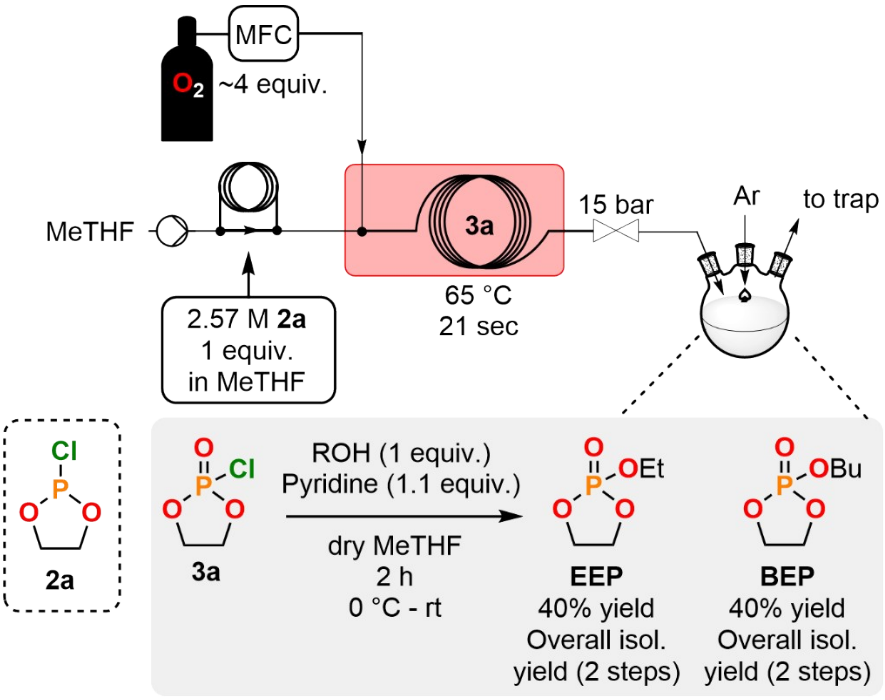

Figure S10. Two-step semi-continuous flow synthesis of monomer **EEP** and **BEP** from purified **2a**.

2.5.6 Batch procedure for the synthesis of 2-chloro-4-(chloromethyl)-1,3,2-dioxaphospholane (2g)

The general procedure was adapted from the literature.[3] A magnetic stir bar, dried dichloromethane (75 mL) and PCl_3 (43.6 mL, 0.5 mol, 1 equiv.) were added into a flame-dried three-necked round-bottom flask equipped with a dropping funnel containing neat (±)-3-chloro-1,2-propanediol (**1g**, 41.9 mL, 0.5 mol, 1 equiv.) and a reflux condenser connected to an alkaline aqueous trap. The diol was carefully added dropwise to the stirred solution at room temperature while a stream of nitrogen was passed through to expel the hydrogen chloride by-product toward the trap. Two hours after the end of the addition, the solvent was removed under reduced pressure and the resulting crude material was purified by a fractional distillation under vacuum, affording 2-chloro-4-(chloromethyl)-1,3,2-dioxaphospholane as a fuming colorless liquid (**2g**, 69.3 g, 79% yield).

2.5.7 Batch procedure for the synthesis of 2-chloro-4-(chloromethyl)-1,3,2-dioxaphospholane 2-oxide (3g)

The general procedure was adapted from the literature.[3] A magnetic stir bar, dry toluene (250 mL) and 2-chloro-4-(chloromethyl)-1,3,2-dioxaphospholane (**2g**, 20.0 g, 114.0 mmol) were added into a flame-dried three-necked round-bottom flask equipped with a reflux condenser. Oxygen was bubbled through the stirred solution overnight at room temperature. The solvent was removed under reduced pressure and the resulting crude material was purified by a fractional distillation under high vacuum, affording 2-chloro-4-(chloromethyl)-1,3,2-dioxaphospholane 2-oxide as a colorless liquid (**3g**, 12.4 g, 57% yield).

2.5.8 Batch procedure for the synthesis of 4-(chloromethyl)-2-ethoxy-1,3,2-dioxaphospholane 2-oxide (**EClMEP**)

The general procedure was adapted from the literature.[4] A magnetic stir bar, dry THF (50 mL), ethanol (3.4 mL, 58.5 mmol, 1 equiv.) and triethylamine (9.0 mL, 64.4 mmol, 1.1 equiv.) were added into a flame-dried three-necked round-bottom flask equipped with a dropping funnel containing dry THF (50 mL) and 2-chloro-4-(chloromethyl)-1,3,2-dioxaphospholane 2-oxide (**3g**, 11.2 g, 58.5 mmol, 1 equiv.). Under an argon atmosphere, the solution containing **3g** was added dropwise to the stirred ethanol solution at - 5 °C generating triethylammonium chloride salt. The solution was stirred overnight and was allowed to warm to room temperature. The hydrochloride salt was filtered off and the filtrate was concentrated under reduced pressure. The resulting crude material was purified by a fractional distillation under high vacuum, affording 4-(chloromethyl)-2-ethoxy-1,3,2-dioxaphospholane 2-oxide as a colorless liquid (**EClMEP**, 8.0 g, 68% yield).

2.5.9 Batch homopolymerization of **EEP** toward **TEG-PEEP**

1-1-[3,5-Bis(trifluoromethyl)phenyl]-3-cyclohexyl-2-thiourea (TU, 140 mg, 0.4 mmol) and tetraethylene glycol (TEG, 16 mg, 0.08 mmol) were transferred into a flame-dried one-necked round-bottom flask containing a magnetic stir bar and dried by three azeotropic distillations with anhydrous toluene. **EEP** monomer (1 g, 6.6 mmol) was then added in the round-bottom flask and the mixture was put under vacuum during 15 min. After the transfer of 10 ml of anhydrous CH_2Cl_2, the mixture was cooled down to 0 °C, and 1,8-diazabicyclo[5.4.0]undec-7-ene (DBU, 0.2 ml, 1.3 mmol) was finally introduced under a N_2 atmosphere with a syringe equipped with a stainless-steel capillary. The reaction medium was stirred at 0 °C for 30 min. The resulting copolymer was precipitated in cold diethyl ether, recovered by decantation and dried under vacuum before characterization by NMR and SEC analyses.

2.5.10 Batch homopolymerization of **EEP** toward **BzO-PEEP**

1-1-[3,5-Bis(trifluoromethyl)phenyl]-3-cyclohexyl-2-thiourea (TU, 93 mg, 0.25 mmol) was transferred into a flame-dried one-necked round-bottom flask containing a magnetic stir bar and dried by three azeotropic distillations with anhydrous toluene. **EEP** monomer (2.7 g, 17.5 mmol) was then added in the round-bottom flask and the mixture was put under vacuum during 15 min. After the transfer of 10 ml of anhydrous CH_2Cl_2, 2 ml (0.1 mmol) of a benzyl alcohol stock solution (prepared by adding 6.2 mmol of benzyl alcohol in 100 mL of anhydrous CH_2Cl_2) was added under a N_2 atmosphere. The mixture was cooled down to 0 °C, and 1,8-diazabicyclo[5.4.0]undec-7-ene (DBU, 0.1 ml, 0.65 mmol) was finally introduced under a N_2 atmosphere using a syringe equipped with a stainless-steel capillary. The reaction medium

was stirred at 0 °C for 30 min. the copolymer was precipitated in cold diethyl ether. After decantation, the recovered copolymer was dissolved in methanol and dialyzed against methanol (MWCO = 1 kDa) overnight in order to remove impurities. After evaporation of methanol under vacuum, the copolymer was collected and characterized by NMR and SEC analyses.

2.5.11 Batch copolymerization of **EEP** with **EClMEP** toward **PEEP-*co*-PEClMEP**

1-1-[3,5-Bis(trifluoromethyl)phenyl]-3-cyclohexyl-2-thiourea (TU, 93 mg, 0.25 mmol) was transferred into a flame-dried one-necked round-bottom flask containing a magnetic stir bar and dried by three azeotropic distillations with anhydrous toluene. **EEP** monomer (1.33 g, 8.75 mmol for the 50:50 copolymer and 2 g, 13.125 mmol for the 75:25 copolymer) and **EClMEP** (1.63 g, 8.75 mmol for the 50:50 copolymer and 0.81 g, 4.375 mmol for the 75:25 copolymer) were then added in the round-bottom flask and the mixture was put under vacuum during 15 min. After the transfer of 10 ml of anhydrous CH_2Cl_2, 2 ml (0.1 mmol) of benzyl alcohol stock solution (prepared by adding 6.2 mmol of benzyl alcohol in 100 ml of anhydrous CH_2Cl_2) was added under a N_2 atmosphere. The mixture was cooled down to 0 °C, and 1,8-diazabicyclo[5.4.0]undec-7-ene (DBU, 0.1 ml, 0.65 mmol) was finally introduced under a N_2 atmosphere using a syringe equipped with a stainless-steel capillary. The reaction medium was stirred at 0 °C for 30 min. The copolymer was precipitated in cold diethyl ether. After decantation, the recovered copolymer was dissolved in methanol and dialyzed against methanol (MWCO = 1 kDa) overnight in order to remove impurities. After evaporation of methanol under vacuum, the copolymer was collected and characterized by NMR and SEC analyses.

2.5.12 Cytotoxicity assays

Cytotoxicity assays are executed in line with ISO 10993-5 on Biomedical evaluation of medical devices. Primary Bovine fibroblast (Bfb) cells and primary Human umbilical vein endothelial cells (HUVEC) were seeded in Falcon 96-well black flat bottom plates (Corning) at a concentration of 5,000 cells per well and were cultured in DMEM advanced (Thermo Fisher) with 5% FBS (Thermo Fisher) and 1% Penicillin-Streptomycin-Amphotericin B (Lonza) or in EBM™-2 Basal Medium with addition of EGM™-2 MV SingleQuots™ kit (Lonza) respectively. Cells were incubated for 72 hours to reach 80% confluency, after which different concentrations of **MeO-PEO-*b*-PEEP** suspensions were added (n=3 per condition). Suspensions at a concentration of 40 mg/mL were made by dissolving **MeO-PEO-*b*-PEEP** in the corresponding cell culture medium without FBS and growth factors, but with antibiotics. These suspensions had been incubated for 24 hours at 37˚C, 5% CO_2 prior to addition to the cells. Plates were incubated with different **MeO-PEO-*b*-PEEP** concentrations for 24 hours at 37˚C and 5% CO_2. Next, cytotoxicity was determined by live/dead staining. In short, cells were incubated with Hoechst 33342 (final concentration 3.25 µM, Thermo Fisher) and Ethd-1 (final concentration 650 nM, Sigma-Aldrich) and imaged with a BD Pathway 855 high content analyzer. Images were analyzed with MetaXpress (Molecular Devices) software. Viable cell number per well was calculated by subtracting the dead cells (Ethd-1 positive nuclei) from total cell number count (Hoechst positive nuclei) per well. The average amount of viable cells in the culture control wells (wells that underwent the same treatment as the conditions but

without **MeO-PEO-*b*-PEEP**) was set to 100% and the percentage of viable cells per experimental condition was determined relative to this (Figures S11 and S12). Conditions with percentages viable cells < 70% are considered to be cytotoxic.

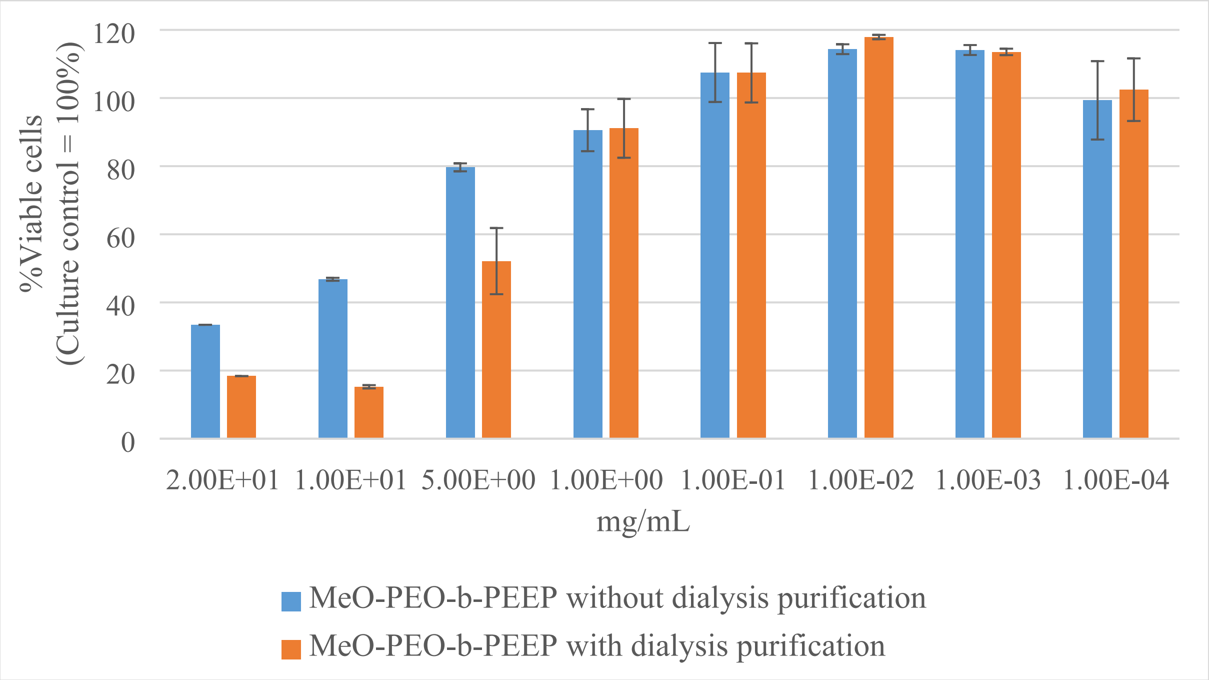

Figure S11. Cytotoxicity assays performed on **MeO-PEO-*b*-PEEP** using bovine fibroblast cells.

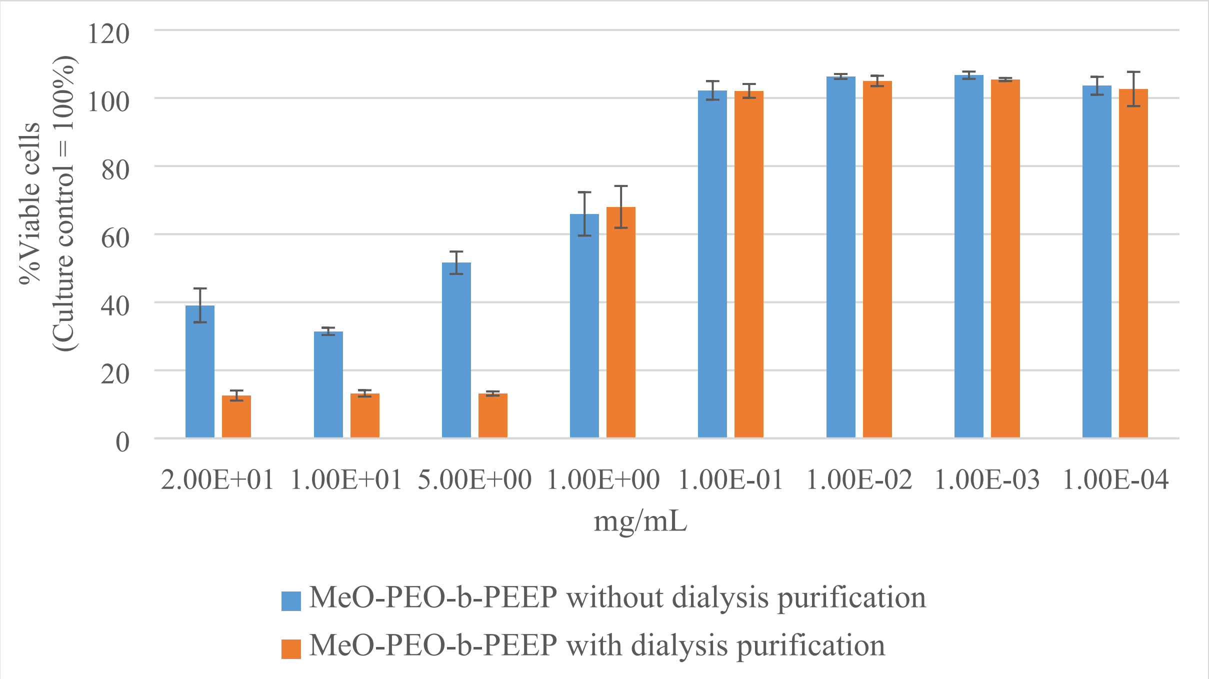

Figure S12. Cytotoxicity assays performed on **MeO-PEO-*b*-PEEP** using HUVEC cells.

2.6 **Characterization of compounds**

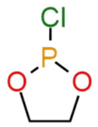

$C_2H_4ClO_2P$
MW 126,48

2-Chloro-1,3,2-dioxaphospholane. ^{1}H NMR (CDCl$_3$, 400 MHz): δ = 4.51 – 4.32 (m, 2H), 4.30 – 4.10 (m, 2H) ppm. **^{13}C NMR (CDCl$_3$, 100.6 MHz):** δ = 65.2 (d, J = 8.8 Hz) ppm. **^{31}P NMR (CDCl$_3$, 162 MHz):** δ = 167.6 ppm. The NMR data match those reported in the literature.[3]

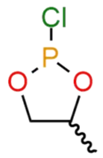

$C_3H_6ClO_2P$
MW 140,51

2-Chloro-4-methyl-1,3,2-dioxaphospholane (mixture of stereoisomers, M = major, m = minor). ^{1}H NMR (CDCl$_3$, 400 MHz, integration values: 1H$_M$ = ~1, 1H$_m$ = ~0.55): δ = 5.00 – 4.69 (m, 2H$_m$), 4.59 – 4.34 (m, 2H$_M$), 4.16 – 3.90 (m, 1H$_m$), 3.82 – 3.62 (m, 1H$_M$), 1.63 – 1.46 (m, 3H$_m$), 1.46 – 1.30 (m, 3H$_M$) ppm. **^{13}C NMR (CDCl$_3$, 100.6 MHz):** δ = 77.0 (d, J = 9.0 Hz, minor), 74.8 (d, J = 8.4 Hz, major), 71.3 (d, J = 7.6 Hz, major), 71.2 (d, J = 8.1 Hz, minor), 19.8 (s, minor), 19.4 (d, J = 3.7 Hz, major) ppm. **^{31}P NMR (CDCl$_3$, 17.4 MHz):** δ = 171.5 (minor), 170.5 (major) ppm. The NMR data match those reported in the literature.[2]

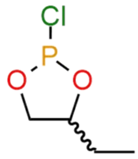

$C_4H_8ClO_2P$
MW 154,53

2-Chloro-4-ethyl-1,3,2-dioxaphospholane (mixture of stereoisomers, M = major, m = minor). ^{1}H NMR (CDCl$_3$, 400 MHz, integration values: 1H$_M$ = ~1, 1H$_m$ = ~0.5): δ = 4.65 (p, J = 6.7 Hz, 1H$_M$), 4.51 – 4.37 (m, 1H$_M$ + 1H$_m$), 4.31 – 4.17 (m, 1H$_m$), 4.04 (t, J = 9.5 Hz, 1H$_m$), 3.86 – 3.73 (m, 1H$_M$), 2.02 – 1.85 (m, 1H$_m$), 1.85 – 1.73 (m, 1H$_m$), 1.73 – 1.59 (m, 2H$_M$), 1.10 – 0.93 (m, 3H$_M$ + 3H$_m$) ppm. **^{13}C NMR (CDCl$_3$, 100.6 MHz):** δ = 82.1 (d, J = 9.5 Hz, minor), 79.5 (d, J = 8.4 Hz, major), 69.9 (d, J = 8.4 Hz, minor), 69.6 (d, J = 7.7 Hz, major), 27.4 (s, minor), 26.80 (d, J = 3.7 Hz, major), 10.3 (s, minor), 9.5 (s, major) ppm. **^{31}P NMR (CDCl$_3$, 17.4 MHz):** δ = 171.5 (minor), 170.3 (major) ppm. **IR (ATR):** 2972, 2941, 2883, 2434, 1463, 1278, 978, 940, 881, 814, 785, 743, 578 cm^{-1}.

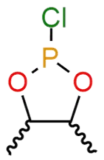

$C_4H_8ClO_2P$
MW 154,53

2-Chloro-4,5-dimethyl-1,3,2-dioxaphospholane (mixture of 3 stereoisomers A – B – C, 18:3.4:1). ^{1}H NMR (CDCl$_3$, 400 MHz): δ_A = 4.87 – 4.72 (m, 2H), 1.30 – 1.23 (m, 6H) ppm. δ_B = 4.47 – 4.36 (m, 1H), 3.99 – 3.86 (m, 1H), 1.54 – 1.47 (m, 6H) ppm. δ_C = 4.70 – 4.61 (m, 2H), 1.46 – 1.41 (m, 6H) ppm. **^{13}C NMR (CDCl$_3$, 100.6 MHz):** δ_A = 76.5 (d, J = 7.3 Hz), 15.6 (d, J = 3.7 Hz), δ_B = 82.6 (d, J = 8.1 Hz), 80.4 (d, J = 7.7 Hz), 19.1 (s), 17.4 (d, J = 5.5 Hz), δ_C = 79.1 (d, J = 9.9 Hz), 16.9 (s) ppm. **^{31}P NMR (CDCl$_3$, 162 MHz):** δ_A = 167.5 ppm. δ_B = 171.1 ppm. δ_C = 172.2 ppm. The NMR data match those reported in the literature.[5,6]

$C_4H_6ClO_2P$
MW 152,52

2-Chloro-4-vinyl-1,3,2-dioxaphospholane (mixture of stereoisomers, M = major, m = minor). ^{1}H NMR ($CDCl_3$, 400 MHz, integration values: $1H_M$ = ~1, $1H_m$ = ~0.7): δ = 6.05 – 5.94 (m, $1H_m$), 5.87 – 5.74 (m, $1H_M$), 5.50 – 5.32 (m, $2H_M + 2H_m$), 5.13 (q, *J* = 7.1 Hz, $1H_M$), 4.78 – 4.65 (m, $1H_m$), 4.58 – 4.42 (m, $1H_M + 1H_m$), 4.11 (t, *J* = 9.6 Hz, $1H_m$), 3.93 – 3.81 (m, $1H_M$) ppm. **^{13}C NMR ($CDCl_3$, 100.6 MHz):** δ = 134.0 (s), 133.2 (d, *J* = 4.3 Hz), 121.3 (s), 120.3 (s), 81.2 (d, *J* = 8.7 Hz), 78.7 (d, *J* = 8.3 Hz), 69.6 (d, *J* = 7.6 Hz), 69.2 (d, *J* = 8.0 Hz) ppm. **^{31}P NMR ($CDCl_3$, 162 MHz):** δ = 171.0 (minor), 170.6 (major) ppm. **IR (ATR):** 3091, 2994, 2961, 2906, 1647, 1467, 1428, 1410, 1285, 1217, 962, 859, 823, 774, 711, 664, 599 cm^{-1}.

$C_4H_8ClO_3P$
MW 170,53

2-Chloro-4-(methoxymethyl)-1,3,2-dioxaphospholane (mixture of stereoisomers, M = major, m = minor). ^{1}H NMR ($CDCl_3$, 400 MHz, integration values: 1 H_M = ~1, 1 H_m = ~0.40): δ = 4.86 – 4.71 (m, $1H_M$), 4.53 – 4.38 (m, $1H_M + 2H_m$), 4.18 – 4.01 (m, $1H_M + 1H_m$), 3.82 – 3.72 (m, $1H_m$), 3.66 – 3.57 (m, $1H_m$), 3.56 – 3.43 (m, $2H_M$), 3.42 – 3.31 (m, $3H_M + 3H_m$) ppm. **^{13}C NMR ($CDCl_3$, 100.6 MHz):** δ = 78.3 (d, *J* = 9.1 Hz, minor), 76.3 (d, *J* = 8.4 Hz, major), 73.5 (s, minor), 72.1 (d, *J* = 3.4 Hz, major), 68.1 (d, *J* = 8.4 Hz, minor), 67.1 (d, *J* = 7.9 Hz, major), 59.6 (s, minor), 59.5 (s, major) ppm. **^{31}P NMR ($CDCl_3$, 162 MHz):** δ = 171.7 (minor), 170.7 (major) ppm. **IR (ATR):** 2986, 2934, 2895, 2821 2450, 1456, 1273, 1198, 1092, 1066, 945, 813, 784, 569 cm^{-1}.

$C_3H_5Cl_2O_2P$
MW 174,95

2-Chloro-4-(chloromethyl)-1,3,2-dioxaphospholane (mixture of stereoisomers, M = major, m = minor). ^{1}H NMR ($CDCl_3$, 400 MHz, integration values: $1H_M$ = ~1, $1H_m$ = ~0.5): δ = 5.00 – 4.88 (m, $1H_M$), 4.67 – 4.49 (m, $1H_M + 2H_m$), 4.35 – 4.14 (m, $1H_M + 1H_m$), 3.93 (dd, *J* = 10.9, 5.3 Hz, $1H_m$), 3.80 – 3.71 (m, $1H_m$), 3.70 – 3.55 (m, $2H_M$) ppm. **^{13}C NMR ($CDCl_3$, 100.6 MHz):** δ = 78.6 (d, *J* = 9.2 Hz, minor), 76.5 (d, *J* = 8.8 Hz, major), 69.7 (d, *J* = 8.4 Hz, minor), 67.9 (d, *J* = 7.7 Hz, major), 44.2 (s, minor), 44.1 (s, major) ppm. **^{31}P NMR ($CDCl_3$, 17.4 MHz):** δ = 172.6 (minor), 171.3 (major) ppm. **IR (ATR):** 2963, 1467, 1445, 1428, 1289, 1229, 975, 897, 876, 850, 772, 739, 652, 602 cm^{-1}.

$C_2H_4ClPS_2$
MW 158,61

2-Chloro-1,3,2-dithiaphospholane. ^{1}H NMR ($CDCl_3$, 400 MHz): δ = 3.77 – 3.64 (m, 2H), 3.63 – 3.51 (m, 2H) ppm. **^{13}C NMR ($CDCl_3$, 100.6 MHz):** δ = 42.8 (d, *J* = 2.6 Hz) ppm. **^{31}P NMR ($CDCl_3$, 162 MHz):** δ = 168.4 ppm. The NMR data match those reported in the literature.[7]

$C_6H_{12}ClO_2P$
MW 182,59

2-Chloro-4,4,5,5-tetramethyl-1,3,2-dioxaphospholane. ^{1}H NMR ($CDCl_3$, 400 MHz): δ = 1.52 (s, 6H), 1.32 (s, 6H) ppm. **^{13}C NMR ($CDCl_3$, 100.6 MHz):** δ = 88.8 (d, J = 8.4 Hz), 25.7 (d, J = 3.5 Hz), 24.7 (d, J = 1.9 Hz) ppm. **^{31}P NMR ($CDCl_3$, 162 MHz):** δ = 175.3 ppm. The NMR data match those reported in the literature.[8]

$C_3H_6ClO_2P$
MW 140,51

2-Chloro-1,3,2-dioxaphosphinane. ^{1}H NMR ($CDCl_3$, 400 MHz): δ = 4.73 – 4.55 (m, 2H), 4.12 – 3.91 (m, 2H), 2.63 – 2.42 (m, 1H), 1.75 – 1.61 (m, 1H) ppm. **^{13}C NMR ($CDCl_3$, 100.6 MHz):** δ = 61.6 (d, J = 2.5 Hz), 28.1 (d, J = 5.2 Hz) ppm. **^{31}P NMR ($CDCl_3$, 162 MHz):** δ = 154.0 ppm. The NMR data match those reported in the literature. [9]

C_2H_4ClOPS
MW 142,54

2-Chloro-1,3,2-oxathiaphospholane. ^{1}H NMR ($CDCl_3$, 400 MHz): δ = 4.77 – 4.67 (m, 1H), 4.62 – 4.50 (m, 1H), 3.43 – 3.33 (m, 1H), 3.22 – 3.12 (m, 1H) ppm. **^{13}C NMR ($CDCl_3$, 100.6 MHz):** δ = 76.7 (d, J = 15.3 Hz), 32.5 (d, J = 2.6 Hz) ppm. **^{31}P NMR ($CDCl_3$, 162 MHz):** δ = 204.7 ppm. The NMR data match those reported in the literature.[7]

C_3H_7ClNOP
MW 139,52

2-Chloro-3-methyl-1,3,2-oxazaphospholidine. ^{1}H NMR ($CDCl_3$, 400 MHz): δ = 4.48 – 4.31 (m, 2H), 3.20 – 3.04 (m, 2H), 2.70 (d, J = 15.4 Hz, 3H) ppm. **^{13}C NMR ($CDCl_3$, 100.6 MHz):** δ = 70.8 (d, J = 9.5 Hz), 48.7 (d, J = 7.3 Hz), 30.8 (d, J = 13.9 Hz) ppm. **^{31}P NMR ($CDCl_3$, 162 MHz):** δ = 169.6 ppm. The NMR data match those reported in the literature.[10]

$C_4H_{10}ClN_2P$
MW 152,56

2-Chloro-1,3-dimethyl-1,3,2-diazaphospholidine. ^{1}H NMR ($CDCl_3$, 400 MHz): δ = 3.22 (d, J = 7.7 Hz, 4H), 2.68 (dd, J = 15.0, 2.3 Hz, 6H) ppm. **^{13}C NMR ($CDCl_3$, 100.6 MHz):** δ = 52.8 (d, J = 11.0 Hz), 33.2 (d, J = 18.7 Hz) ppm. **^{31}P NMR ($CDCl_3$, 162 MHz):** δ = 171.0 ppm. The NMR data match those reported in the literature.[11]

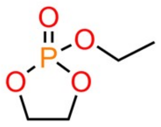

$C_4H_9O_4P$
MW 152,09

2-Ethoxy-1,3,2-dioxaphospholane 2-oxide. ^{1}H NMR ($CDCl_3$, 400 MHz): δ = 4.50 – 4.14 (m, 4H), 4.14 – 3.86 (m, 2H), 1.39 – 1.05 (m, 3H) ppm. **^{13}C NMR ($CDCl_3$, 100.6 MHz):** δ = 66.9 (d, J = 2.6 Hz), 64.7 (d, J = 6.2 Hz), 15.8 (d, J = 6.2 Hz) ppm. **^{31}P NMR ($CDCl_3$, 162 MHz):** δ = 17.2 ppm. The NMR data match those reported in the literature.[12]

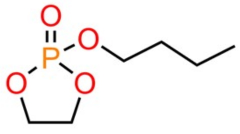

$C_6H_{13}O_4P$
MW 180,14

2-Butoxy-1,3,2-dioxaphospholane 2-oxide. **^{1}H NMR ($CDCl_3$, 400 MHz)**: δ = 4.51 – 4.25 (m, 4H), 4.20 – 4.04 (m, 2H), 1.75 – 1.56 (m, 2H), 1.49 – 1.28 (m, 2H), 0.91 (t, J = 7.3 Hz, 3H) ppm. **^{13}C NMR ($CDCl_3$, 100.6 MHz):** δ = 68.7 (d, J = 6.3 Hz), 65.9 (d, J = 2.4 Hz), 32.2 (d, J = 6.1 Hz), 18.5 (s), 13.4 (s) ppm. **^{31}P NMR ($CDCl_3$, 162 MHz):** δ = 17.6 ppm. The NMR data match those reported in the literature.[13]

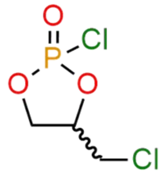

$C_3H_5Cl_2O_3P$
MW 190,95

2-Chloro-4-(chloromethyl)-1,3,2-dioxaphospholane 2-oxide (mixture of stereoisomers, M = major, m = minor). ^{1}H NMR ($CDCl_3$, 400 MHz, integration values: 1 H_M = ~1, 1 H_m = ~0.55): δ = 5.04 – 4.94 (m, 1H_m), 4.94 – 4.84 (m, 1H_M), 4.73 – 4.60 (m, 1H_m), 4.60 – 4.50 (m, 1H_M), 4.49 – 4.38 (m, 1 H_M), 4.38 – 4.29 (m, 1H_m), 3.74 (d, J = 5.3 Hz, 2H_M + 2H_m) ppm. **^{13}C NMR ($CDCl_3$, 100.6 MHz):** δ = 77.6 (d, J = 2.2 Hz, minor), 76.5 (d, J = 2.6 Hz, major), 68.7 (s, minor), 68.4 (s, major), 43.3 (d, J = 6.6 Hz, major), 42.5 (d, J = 6.2 Hz, minor) ppm. **^{31}P NMR ($CDCl_3$, 162 MHz):** δ = 21.3 (major), 21.2 (minor) ppm. **IR (ATR):** 3021, 2973, 2921, 1474, 1429, 1304, 997, 921, 888, 845, 812, 751, 599 cm^{-1}.

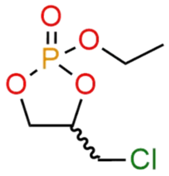

$C_5H_{10}ClO_4P$
MW 200,56

4-(Chloromethyl)-2-ethoxy-1,3,2-dioxaphospholane 2-oxide (mixture of stereoisomers). ^{1}H NMR ($CDCl_3$, 400 MHz): δ = 4.82 – 4.59 (m, 1H), 4.52 – 4.33 (m, 1H), 4.30 – 4.07 (m, 3H), 3.73 – 3.58 (m, 2H), 1.30 (t, J = 7.1 Hz, 3H) ppm. **^{13}C NMR ($CDCl_3$, 100.6 MHz):** δ = 75.8 (d, J = 2.6 Hz, major), 75.7 (d, J = 2.9 Hz, minor), 68.1 (d, J = 1.1 Hz, minor), 68.0 (d, J = 0.7 Hz, major), 65.5 (d, J = 6.2 Hz, minor), 65.3 (d, J = 6.2 Hz, major), 43.2 (d, J = 5.9 Hz, major), 43.1 (d, J = 5.9 Hz, minor), 16.1 (d, J = 5.8 Hz, minor), 16.1 (d, J = 6.2 Hz, major) ppm. **^{31}P NMR ($CDCl_3$, 162 MHz):** δ = 16.3 (minor), 16.2 (major) ppm. **IR (ATR):** 2994, 2918, 1480, 1444, 1432, 1392, 1371, 1283, 1176, 1006, 915, 836, 751, 611 cm^{-1}. **ESI HRMS** *m/z* $C_5H_{11}O_4ClP$ $[M+H]^+$: calcd 201.0078; found 201.0078.

2.7 **Copies of NMR and IR spectra**

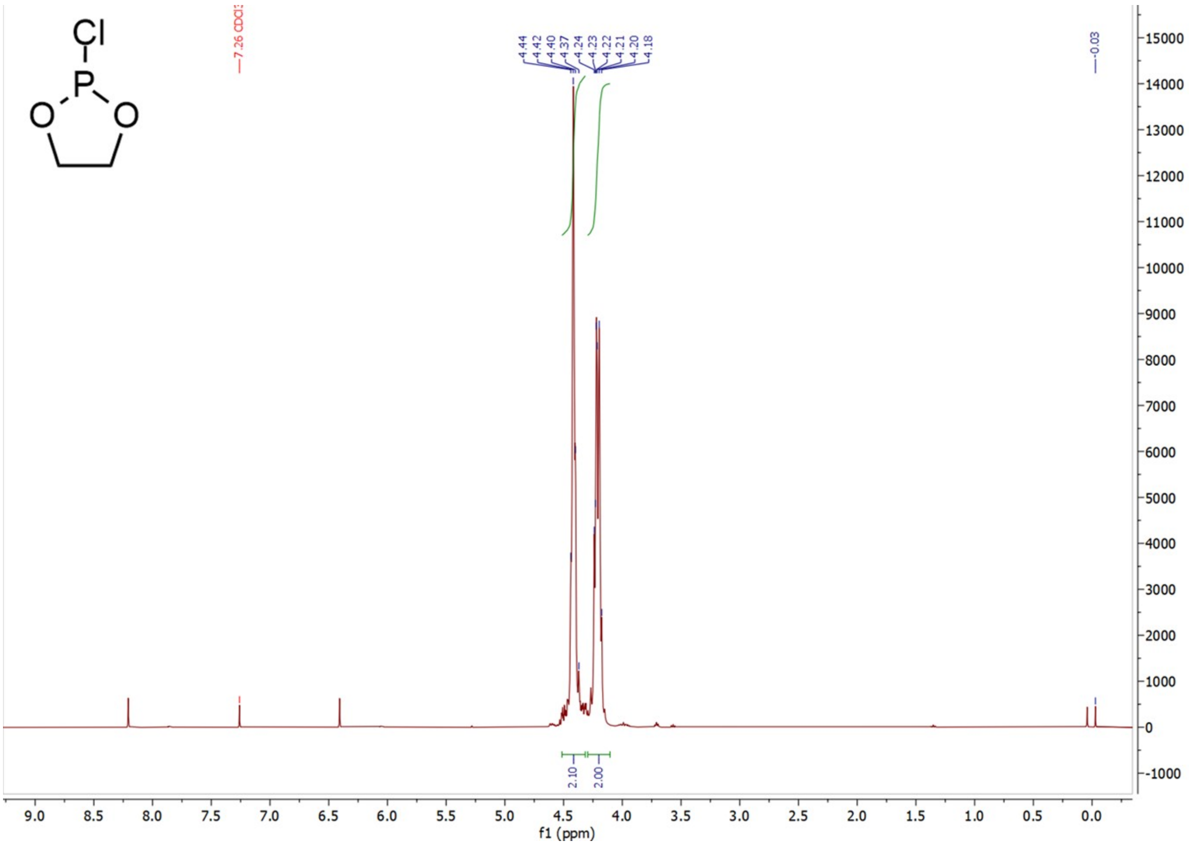

Figure S13. ^{1}H NMR spectrum (400 MHz) of 2-chloro-1,3,2-dioxaphospholane in $CDCl_3$.

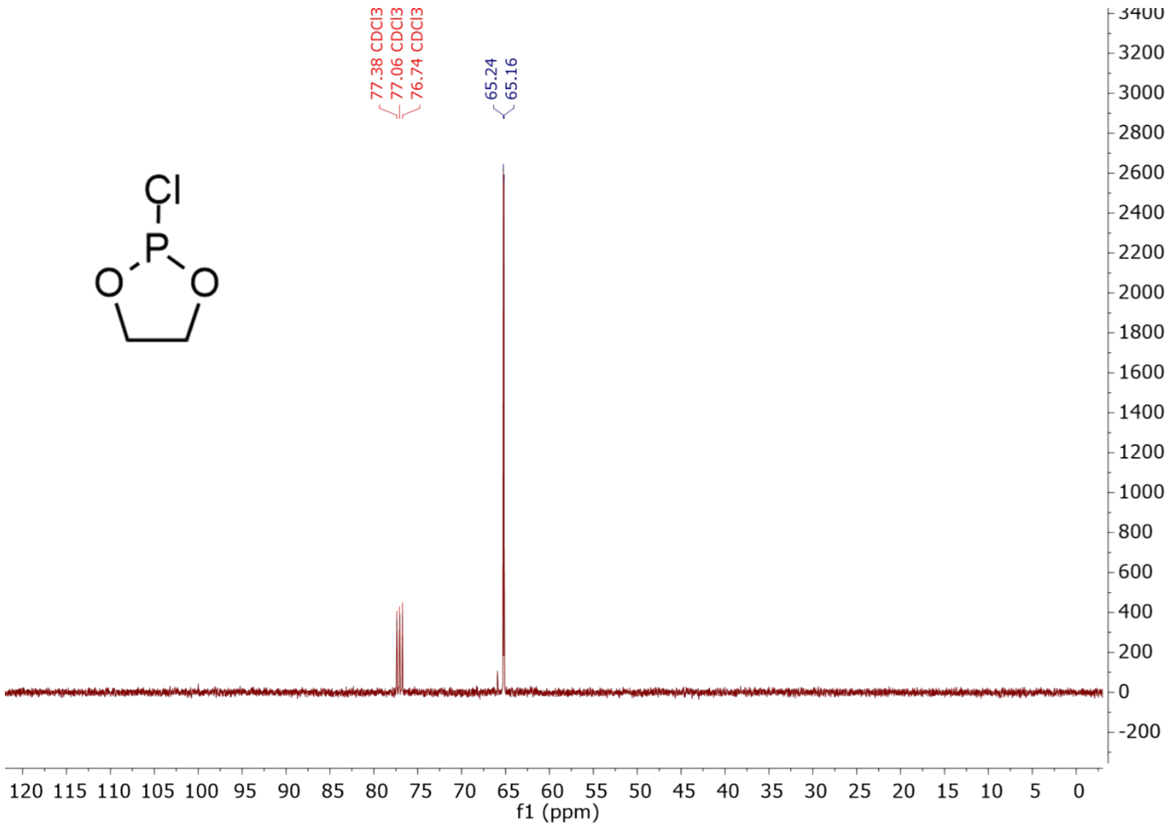

Figure S14. ^{13}C APT NMR spectrum (100.6 MHz) of 2-chloro-1,3,2-dioxaphospholane in CDCl3.

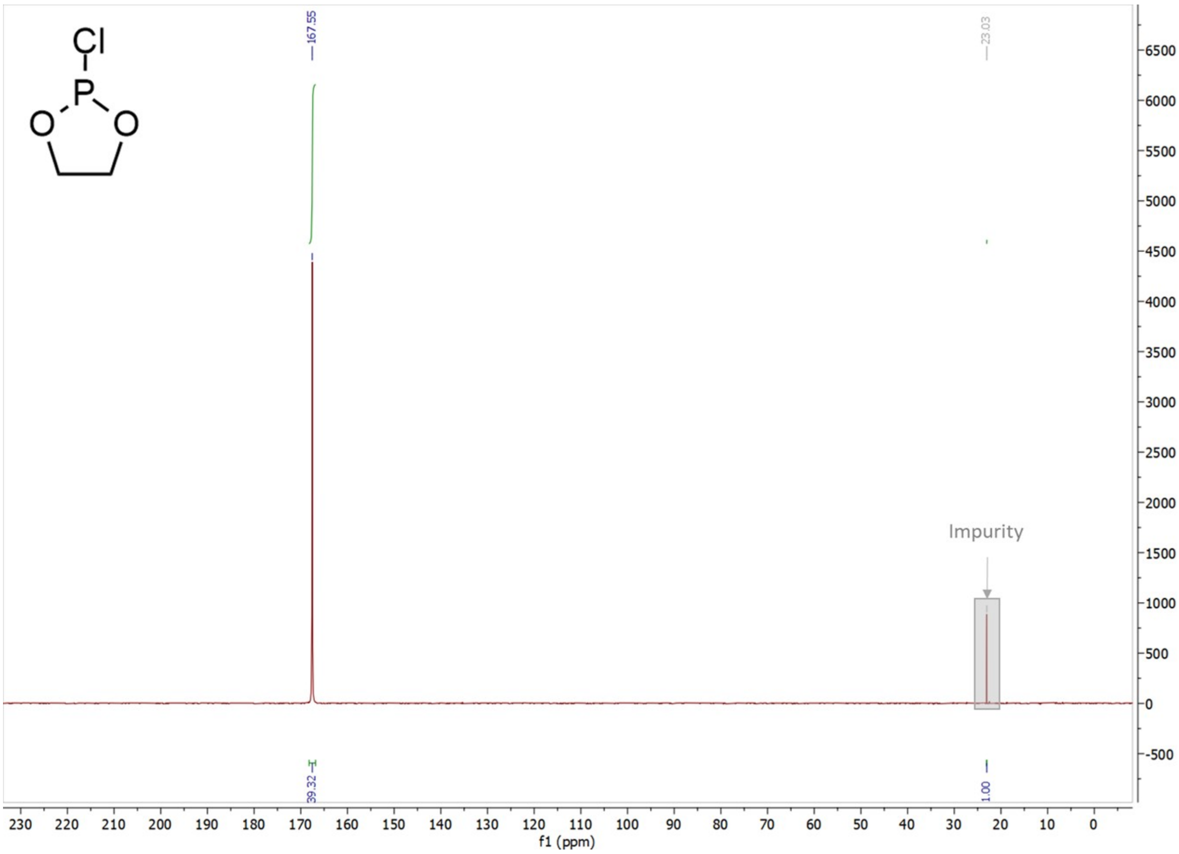

Figure S15. ^{31}P NMR spectrum (162 MHz) of 2-chloro-1,3,2-dioxaphospholane in $CDCl_3$.

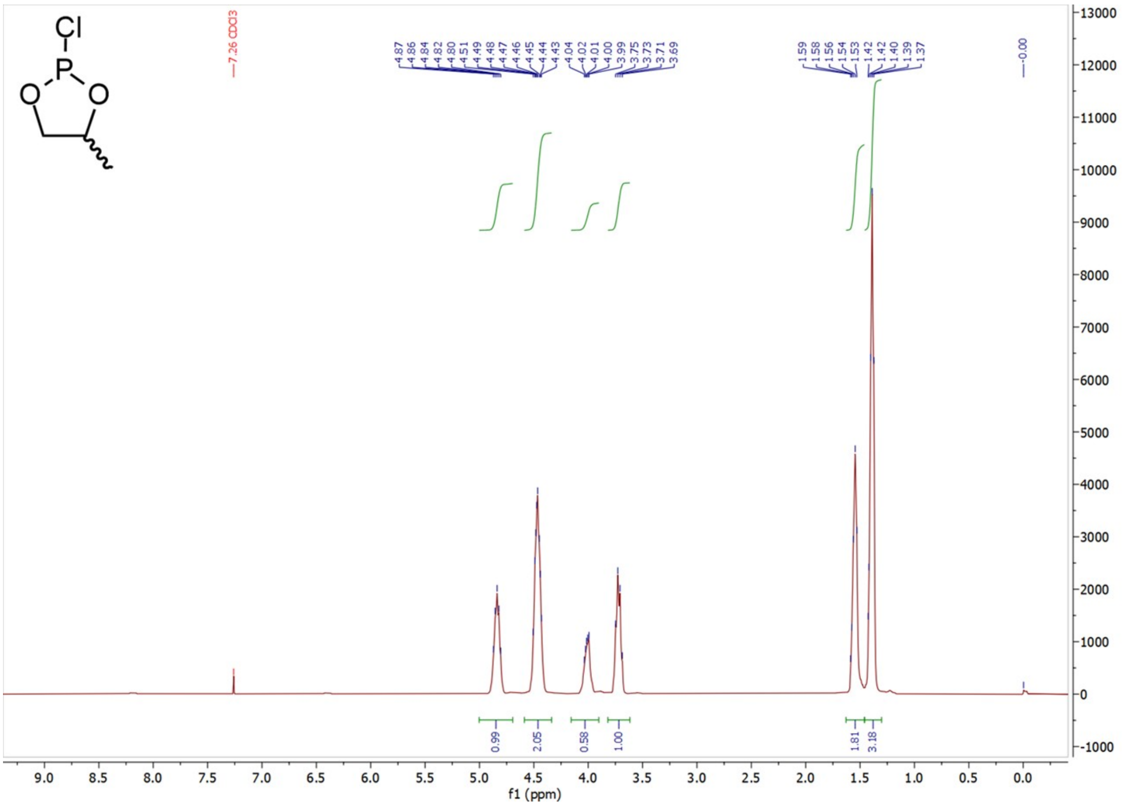

Figure S16. ^{1}H NMR spectrum (400 MHz) of 2-chloro-4-methyl-1,3,2-dioxaphospholane (mixture of stereoisomers) in $CDCl_3$.

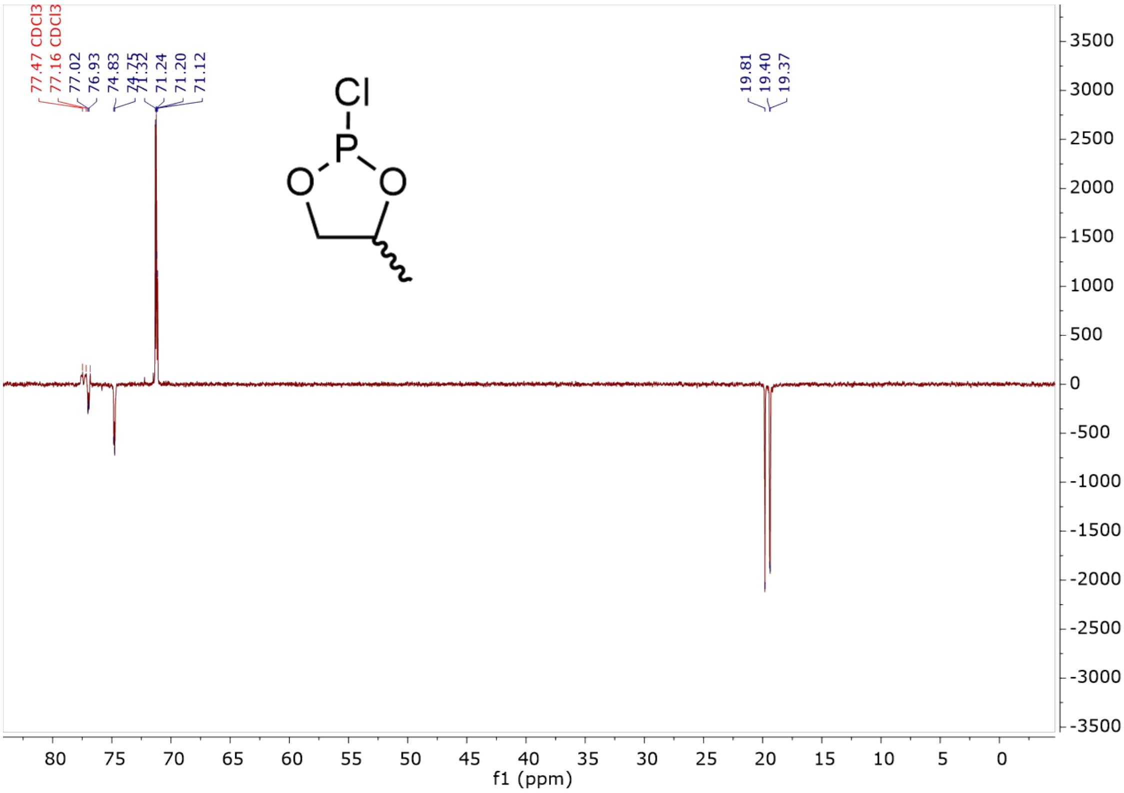

Figure S17. ^{13}C APT NMR spectrum (100.6 MHz) of 2-chloro-4-methyl-1,3,2-dioxaphospholane (mixture of stereoisomers) in $CDCl_3$.

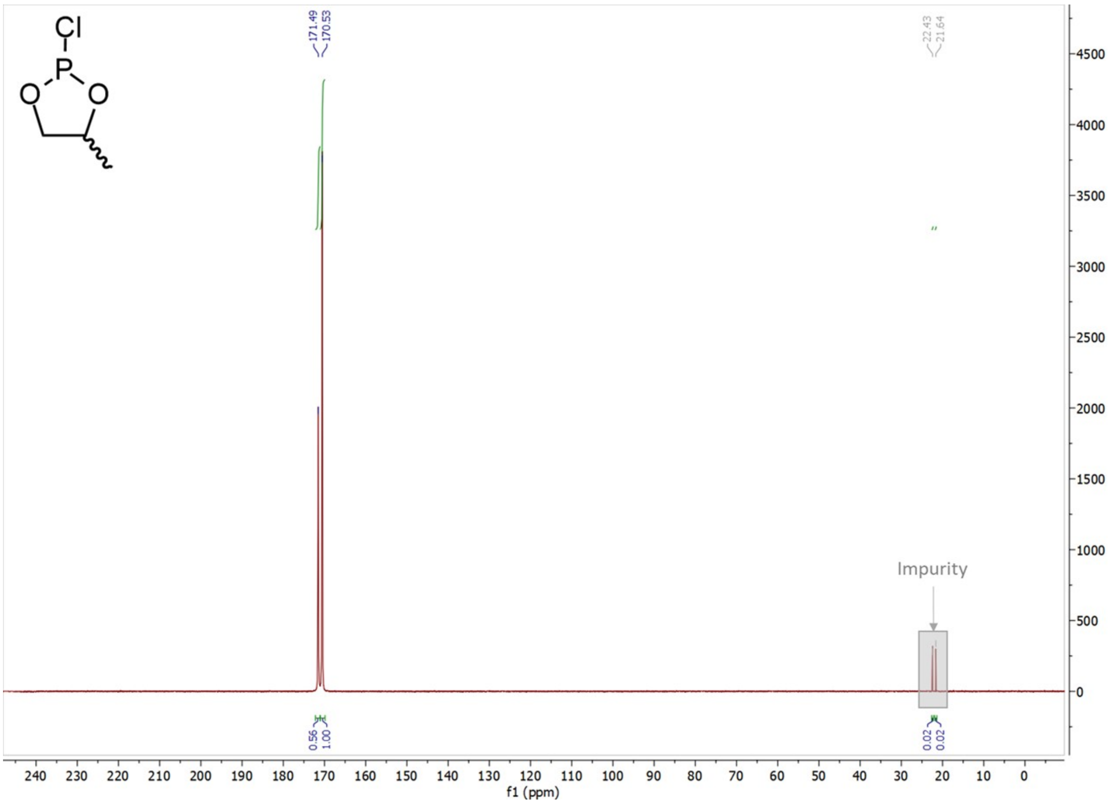

Figure S18. ^{31}P NMR spectrum (162 MHz) of 2-chloro-4-methyl-1,3,2-dioxaphospholane (mixture of stereoisomers) in $CDCl_3$.

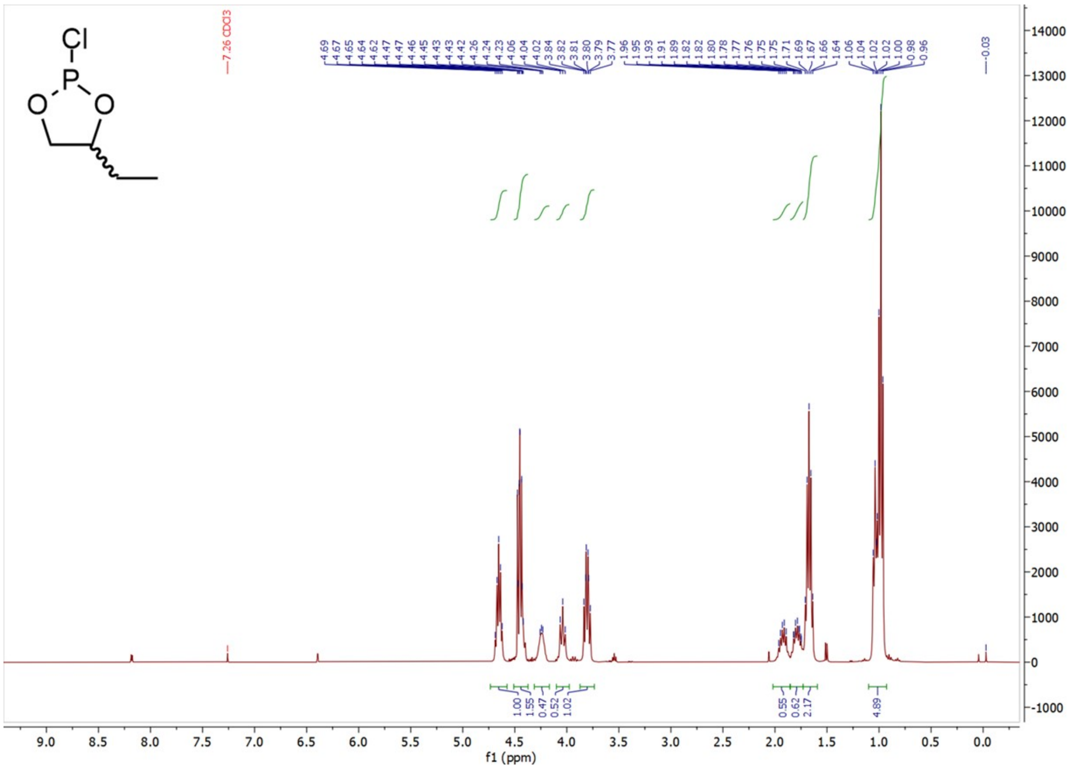

Figure S19. ^{1}H NMR spectrum (400 MHz) of 2-chloro-4-ethyl-1,3,2-dioxaphospholane (mixture of stereoisomers) in $CDCl_3$.

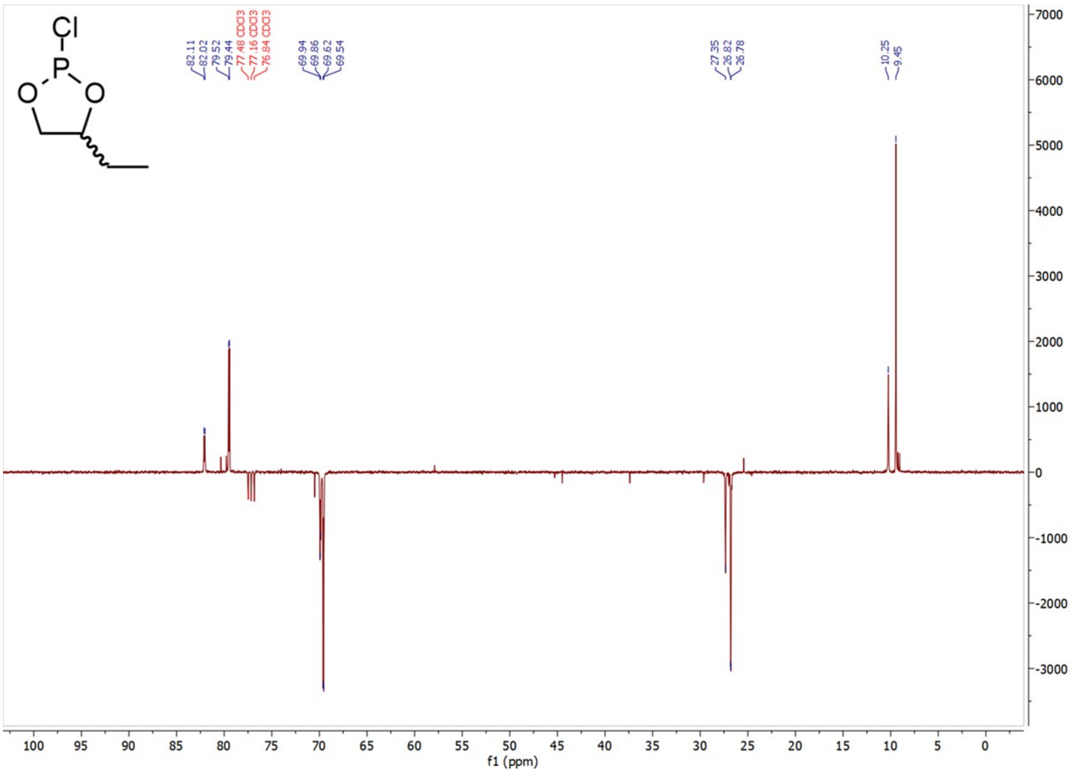

Figure S20. ^{13}C APT NMR spectrum (100.6 MHz) of 2-chloro-4-ethyl-1,3,2-dioxaphospholane (mixture of stereoisomers) in $CDCl_3$.

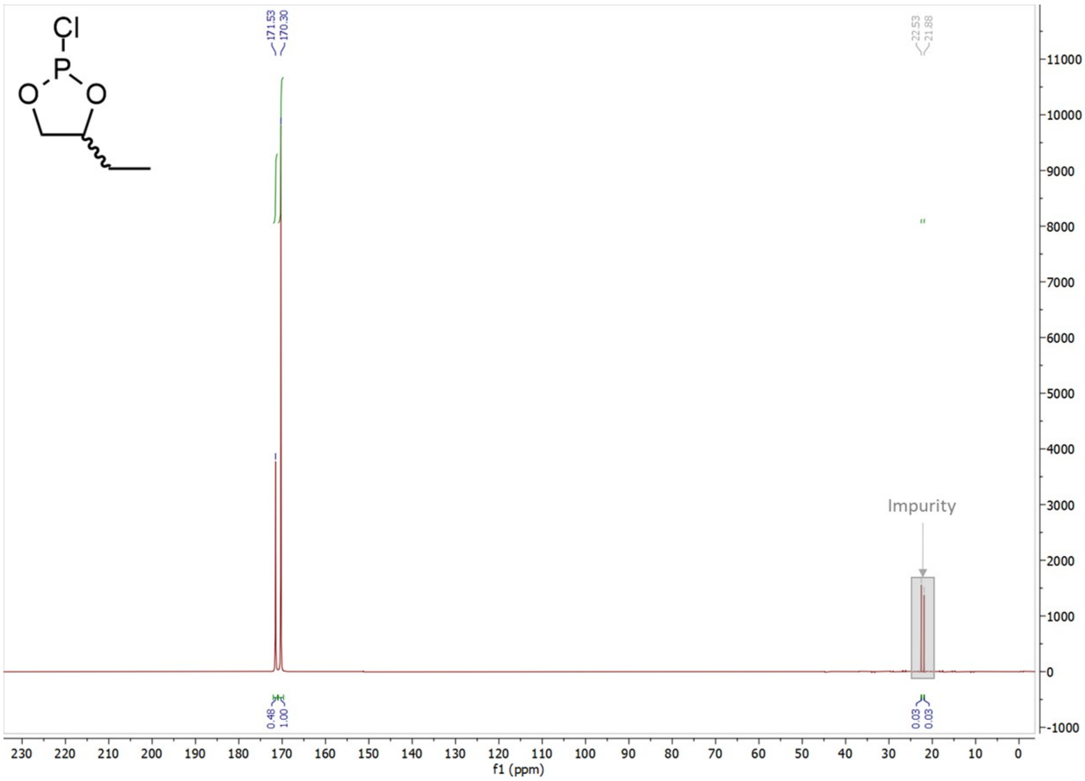

Figure S21. ^{31}P NMR spectrum (162 MHz) of 2-chloro-4-ethyl-1,3,2-dioxaphospholane (mixture of stereoisomers) in $CDCl_3$.

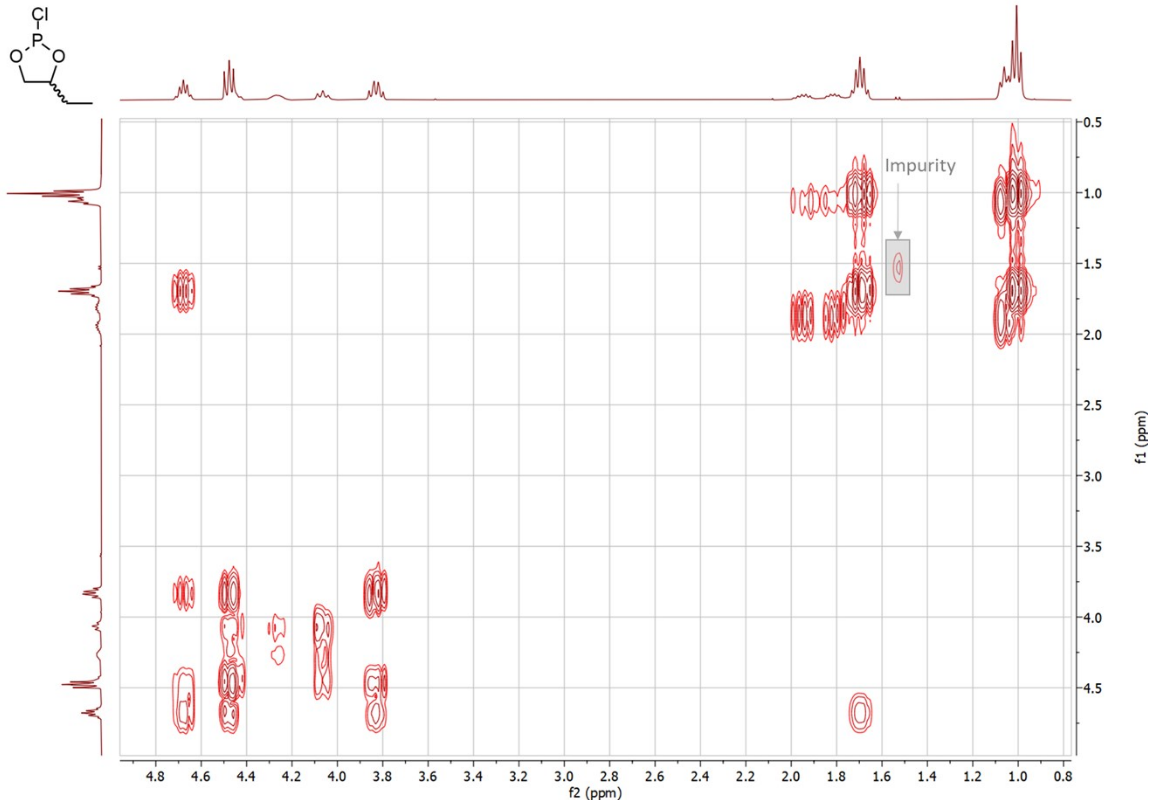

Figure S22. COSY NMR spectrum of 2-chloro-4-ethyl-1,3,2-dioxaphospholane (mixture of stereoisomers) in $CDCl_3$.

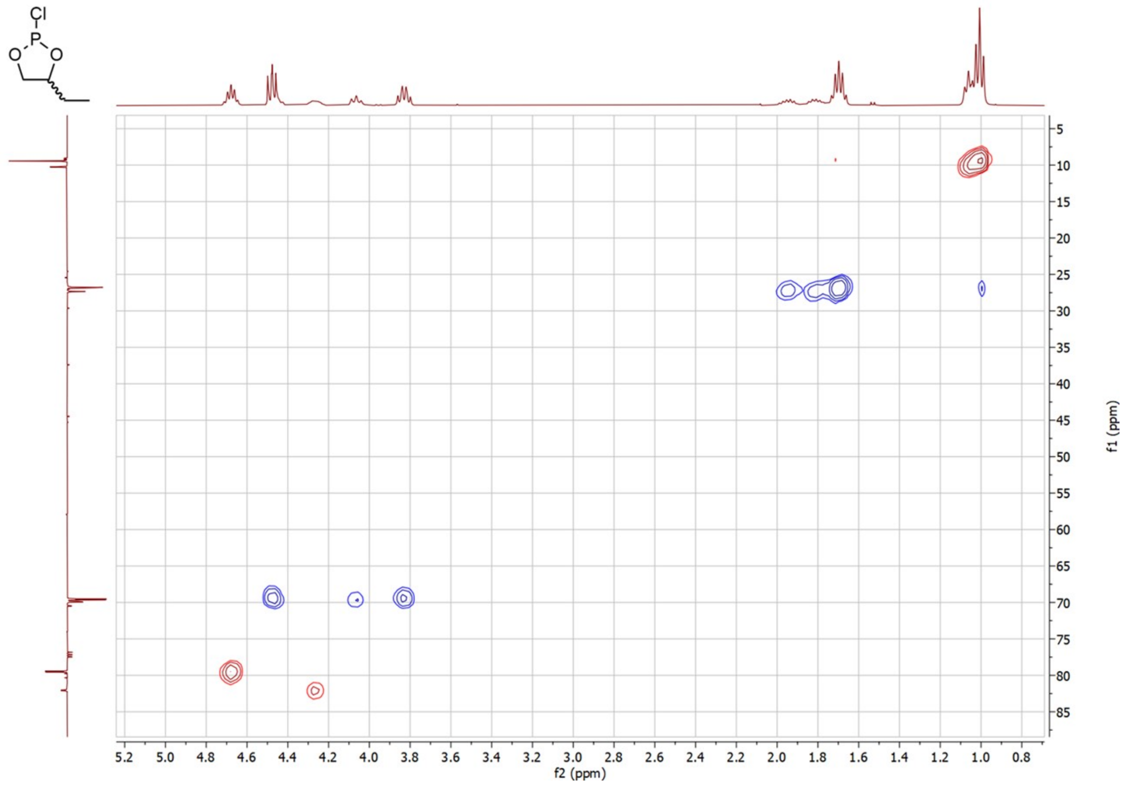

Figure S23. HSQC NMR spectrum of 2-chloro-4-ethyl-1,3,2-dioxaphospholane (mixture of stereoisomers) in $CDCl_3$.

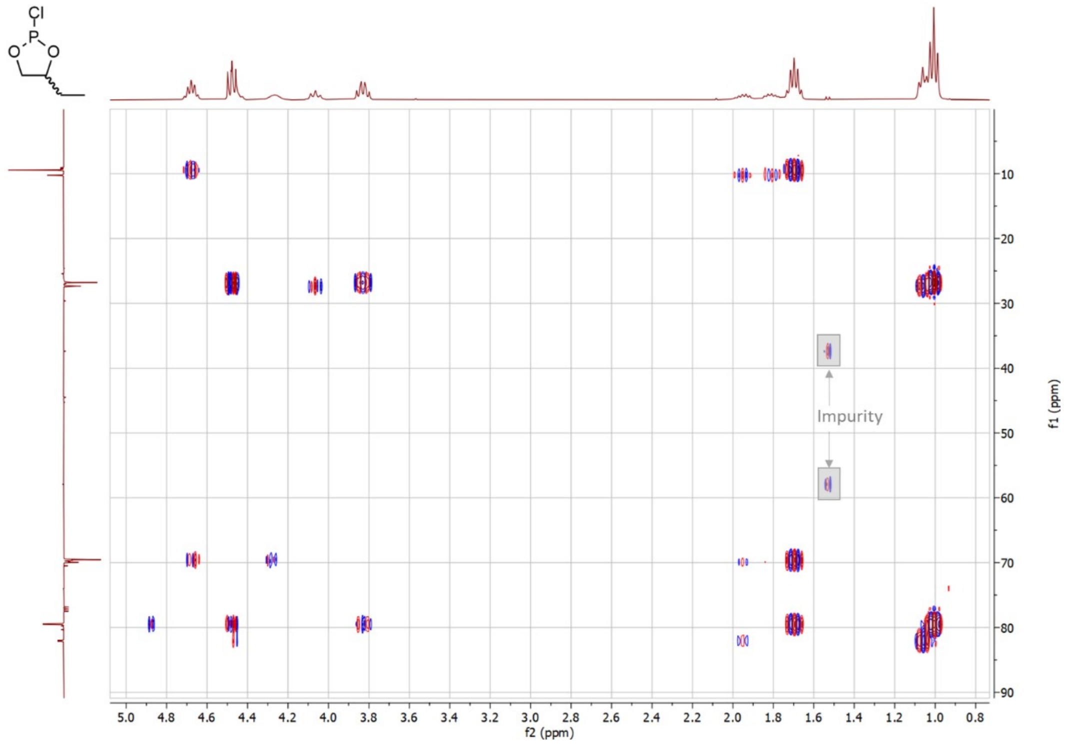

Figure S24. HMBC NMR spectrum of 2-chloro-4-ethyl-1,3,2-dioxaphospholane (mixture of stereoisomers) in $CDCl_3$.

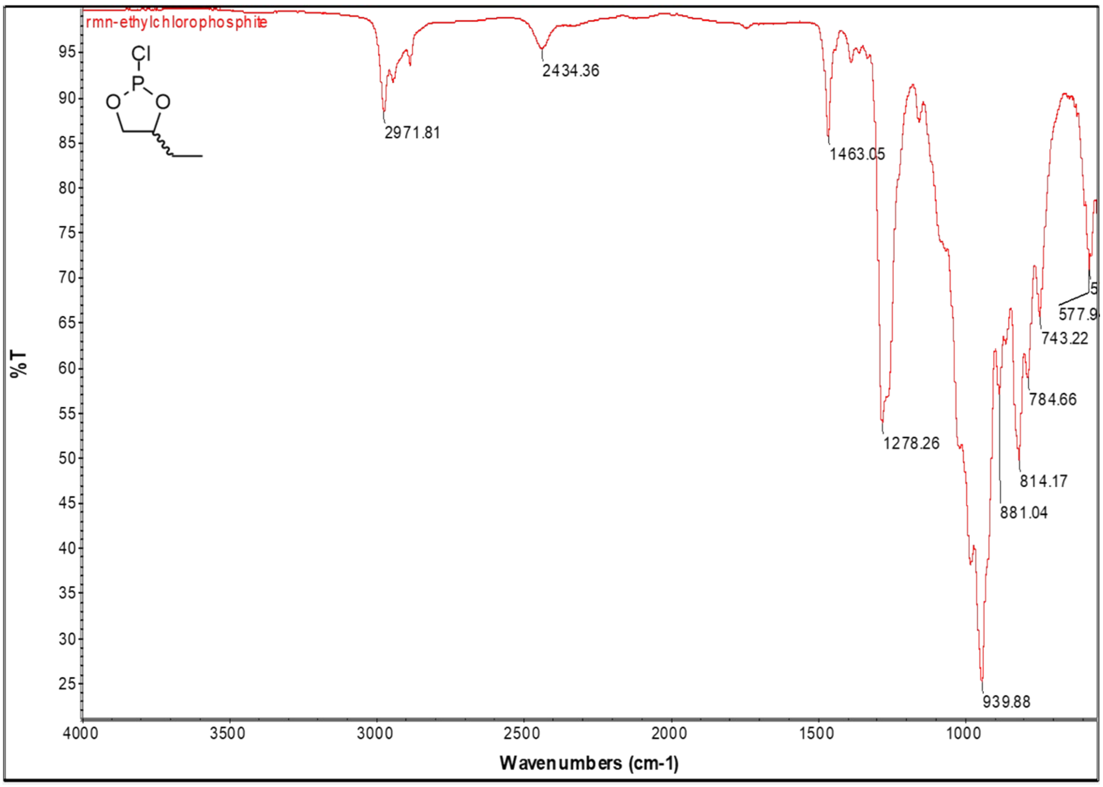

Figure S25. Infrared spectrum (ATR) of 2-chloro-4-ethyl-1,3,2-dioxaphospholane (mixture of stereoisomers).

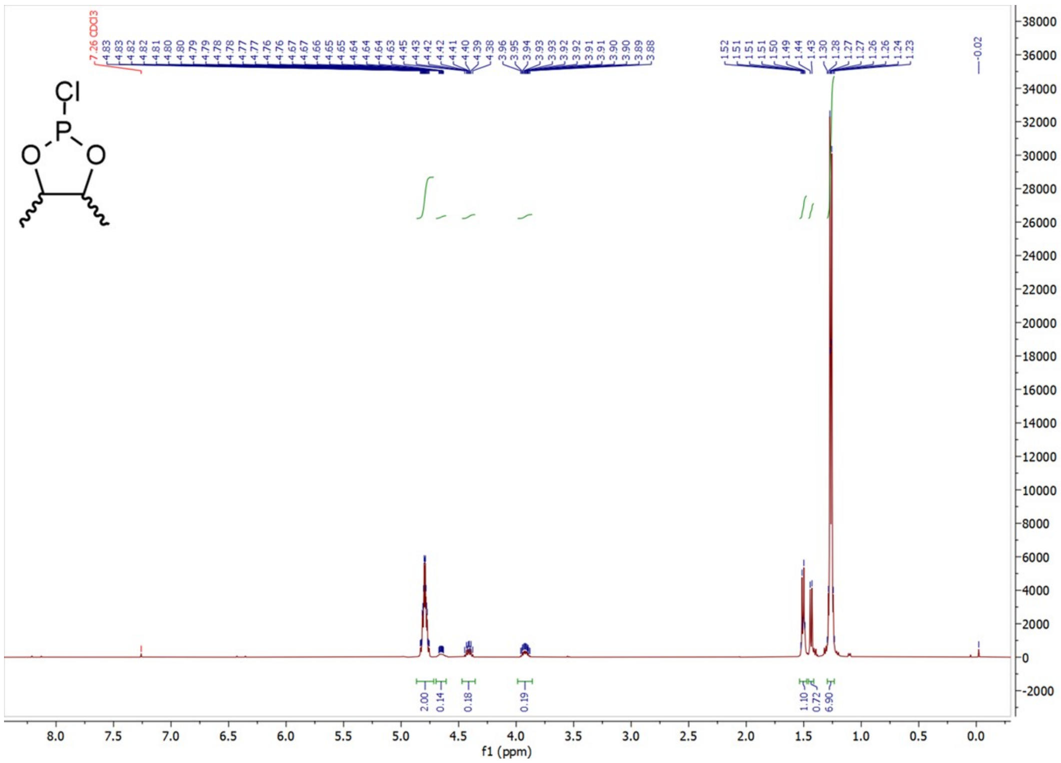

Figure S26. ^{1}H NMR spectrum (400 MHz) of 2-chloro-4,5-dimethyl-1,3,2-dioxaphospholane (mixture of stereoisomers) in $CDCl_3$.

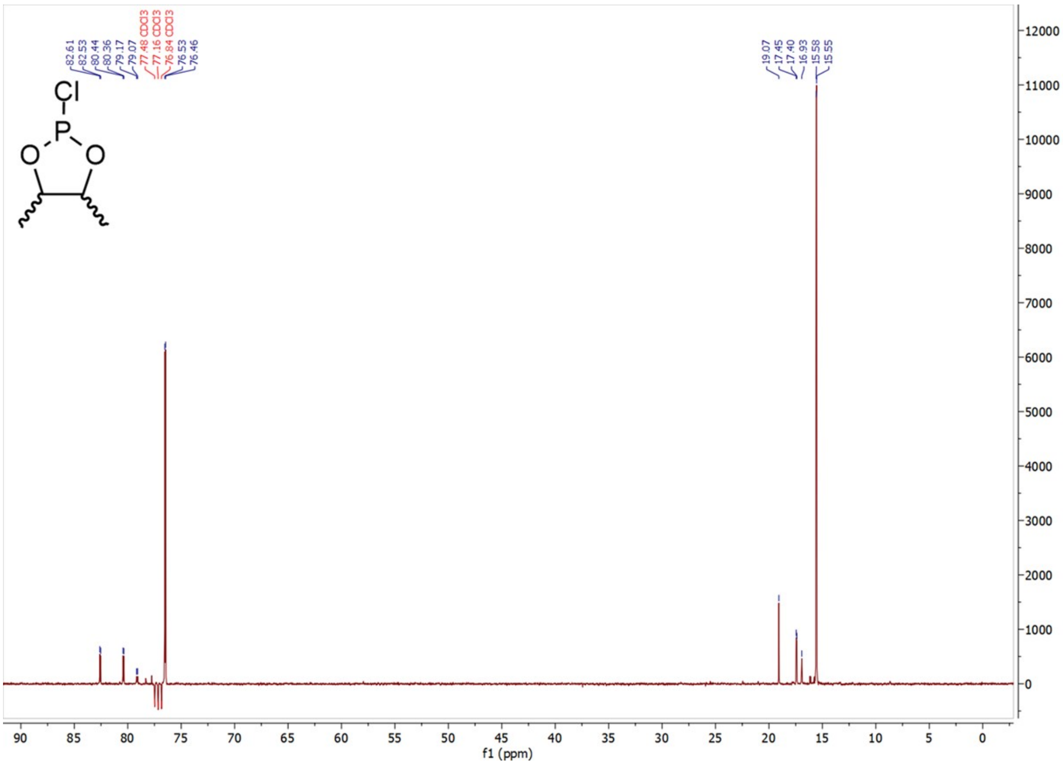

Figure S27. ^{13}C APT NMR spectrum (100.6 MHz) of 2-chloro-4,5-dimethyl-1,3,2-dioxaphospholane (mixture of stereoisomers) in $CDCl_3$.

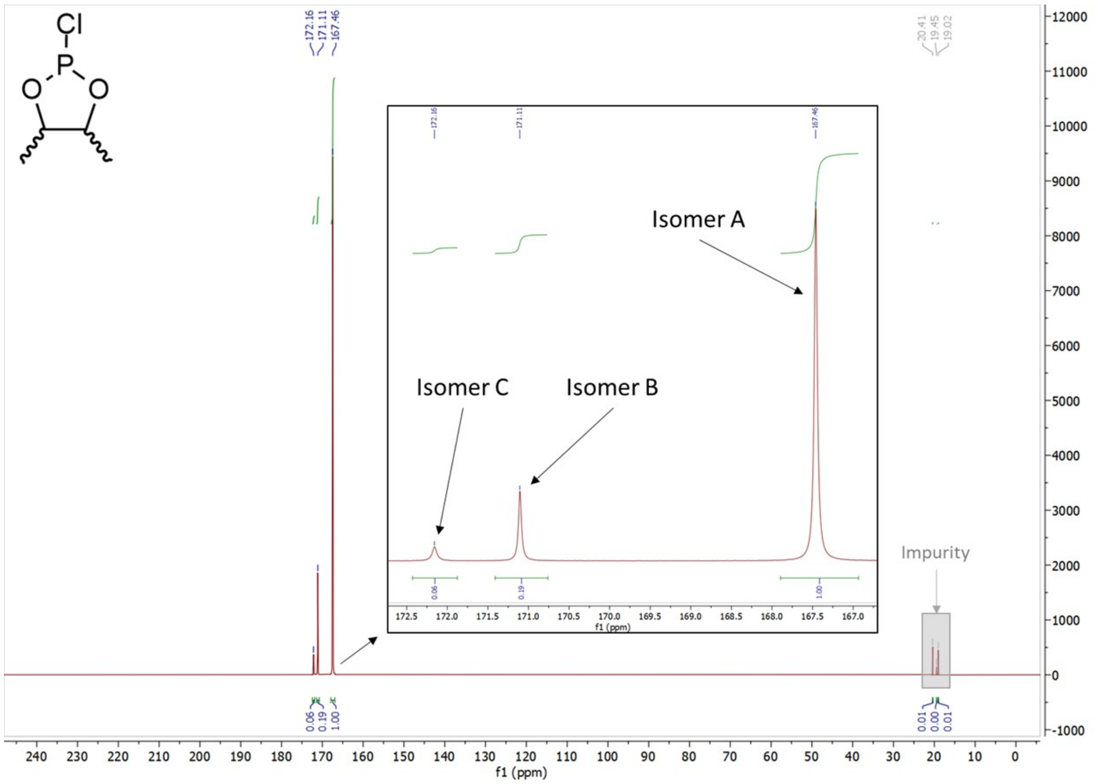

Figure S28. ^{31}P NMR spectrum (162 MHz) of 2-chloro-4,5-dimethyl-1,3,2-dioxaphospholane (mixture of stereoisomers) in $CDCl_3$.

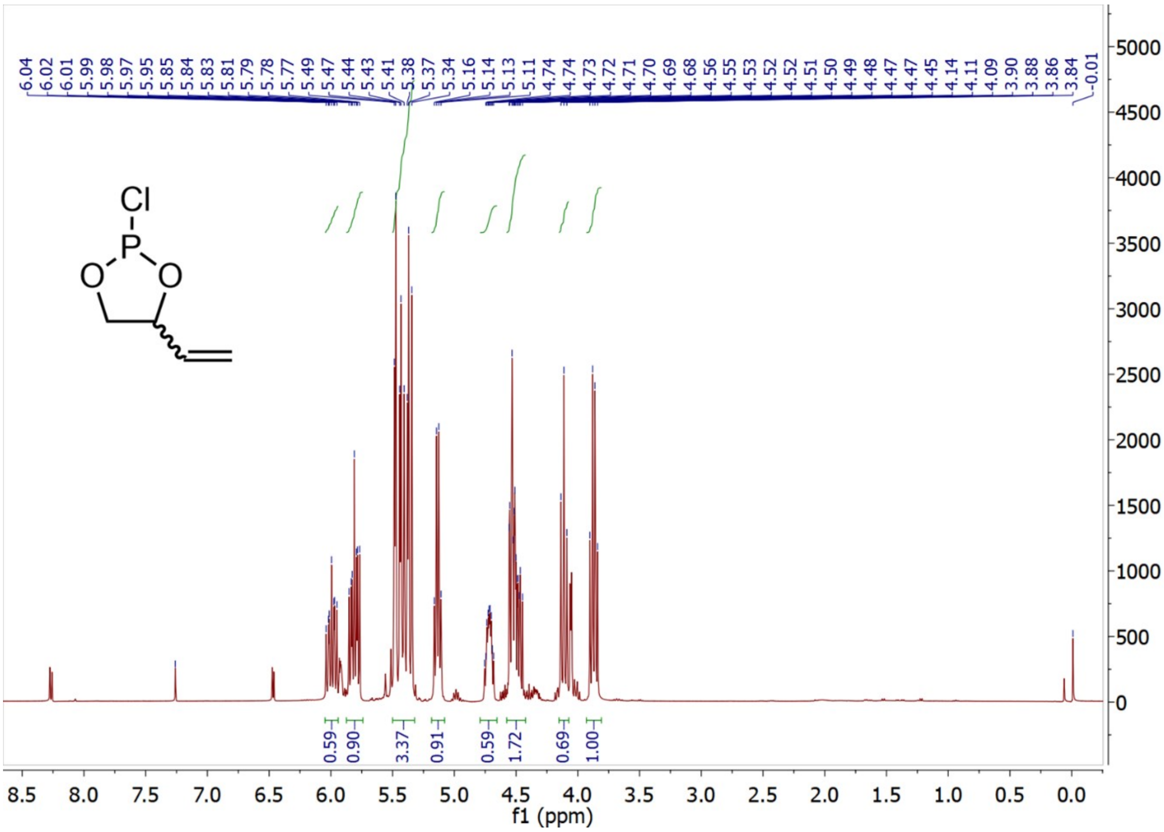

Figure S29. ^{1}H NMR spectrum (400 MHz) of 2-chloro-4-vinyl-1,3,2-dioxaphospholane (mixture of stereoisomers) in $CDCl_3$.

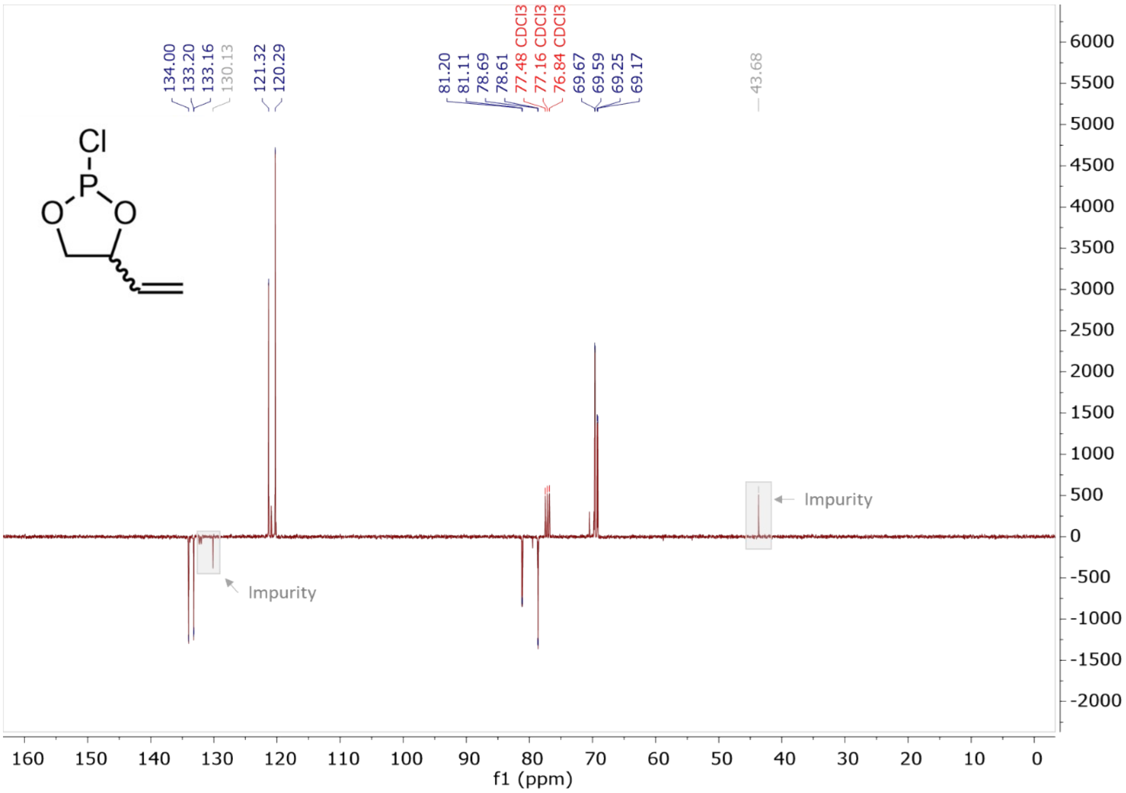

Figure S30. ^{13}C APT NMR spectrum (100.6 MHz) of 2-chloro-4-vinyl-1,3,2-dioxaphospholane (mixture of stereoisomers) in $CDCl_3$.

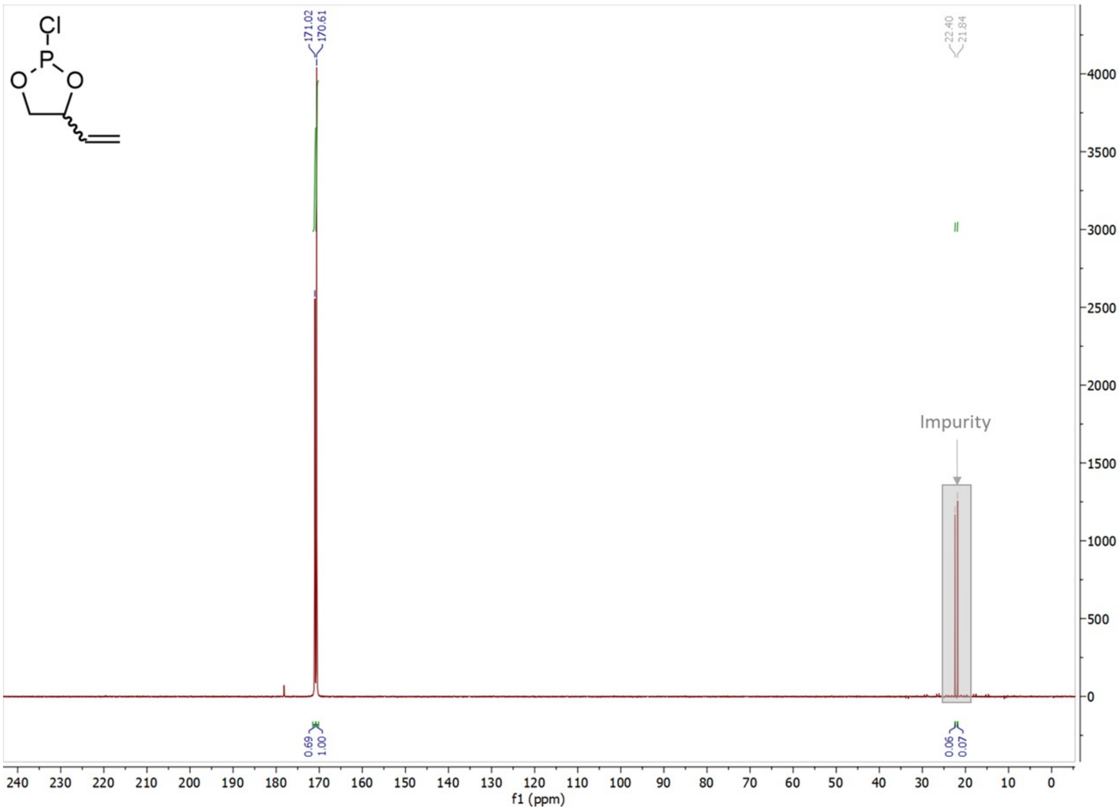

Figure S31. ^{31}P NMR spectrum (162 MHz) of 2-chloro-4-vinyl-1,3,2-dioxaphospholane (mixture of stereoisomers) in $CDCl_3$.

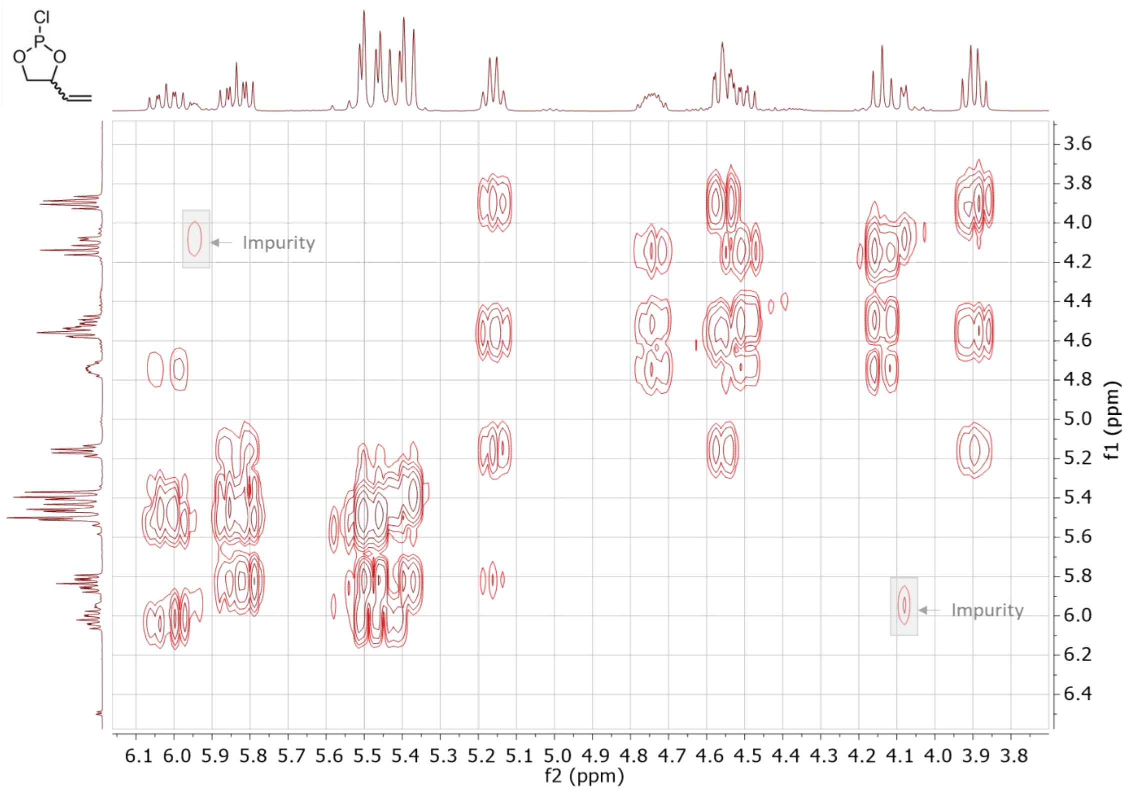

Figure S32. COSY NMR spectrum of 2-chloro-4-vinyl-1,3,2-dioxaphospholane (mixture of stereoisomers) in $CDCl_3$.

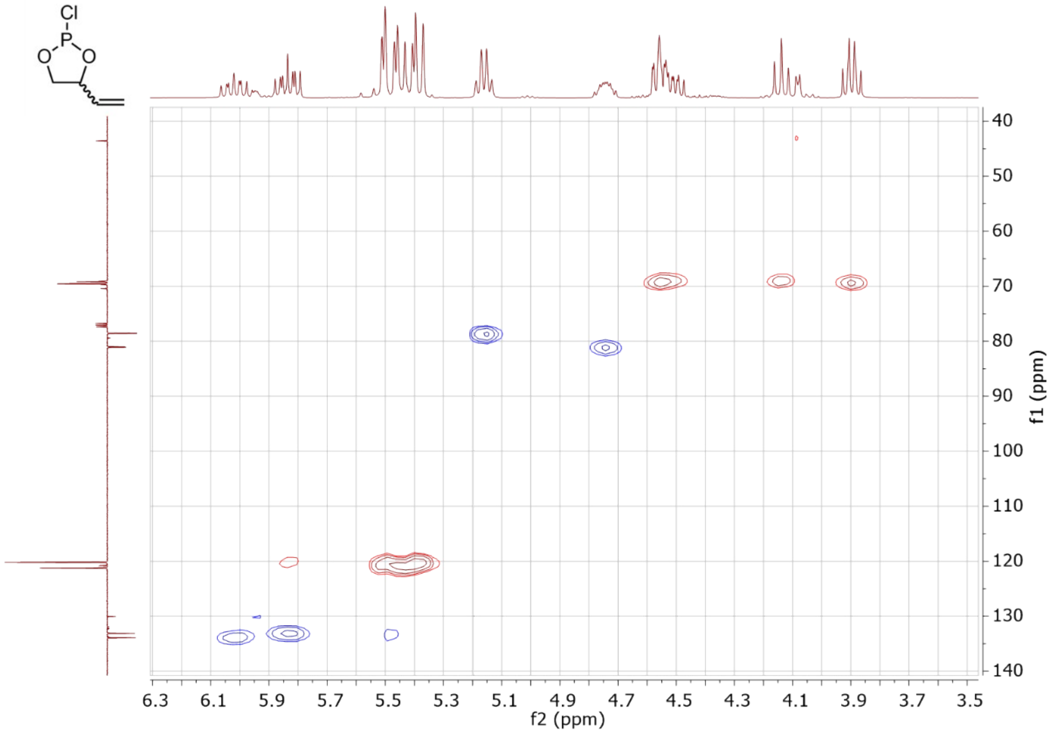

Figure S33. HSQC NMR spectrum of 2-chloro-4-vinyl-1,3,2-dioxaphospholane (mixture of stereoisomers) in $CDCl_3$.

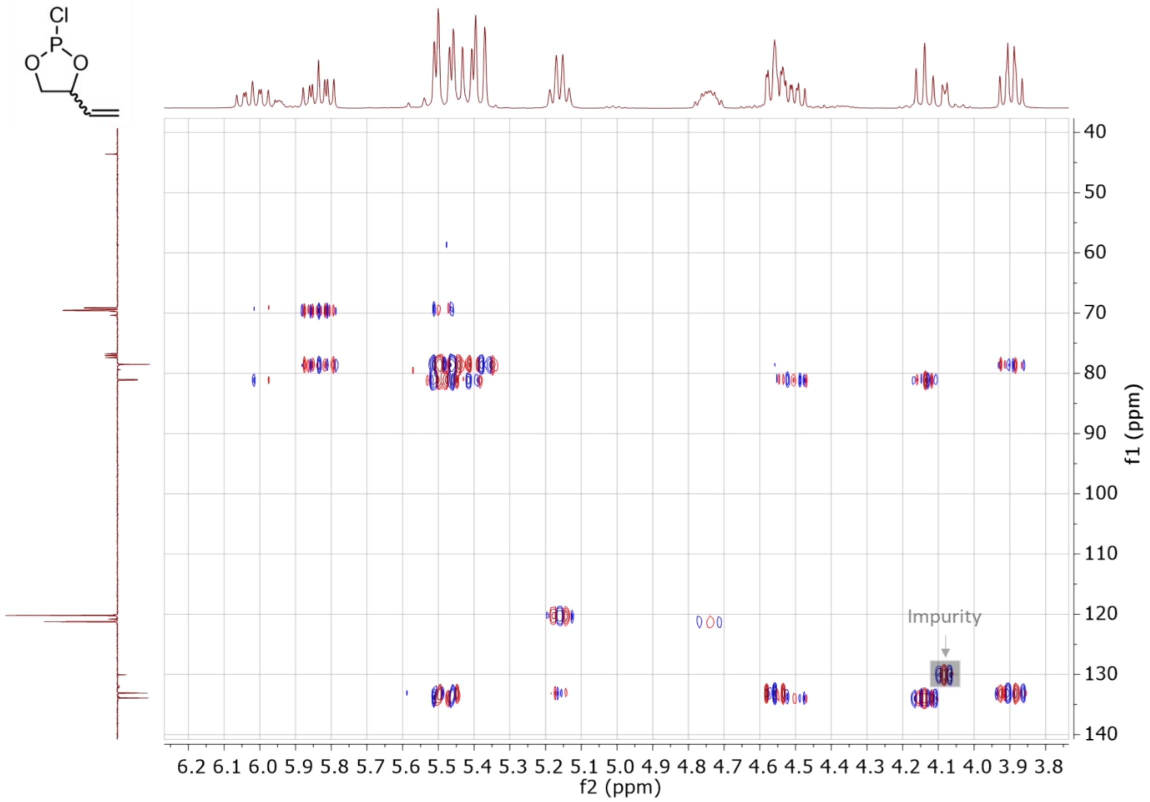

Figure S34. HMBC NMR spectrum of 2-chloro-4-vinyl-1,3,2-dioxaphospholane (mixture of stereoisomers) in $CDCl_3$.

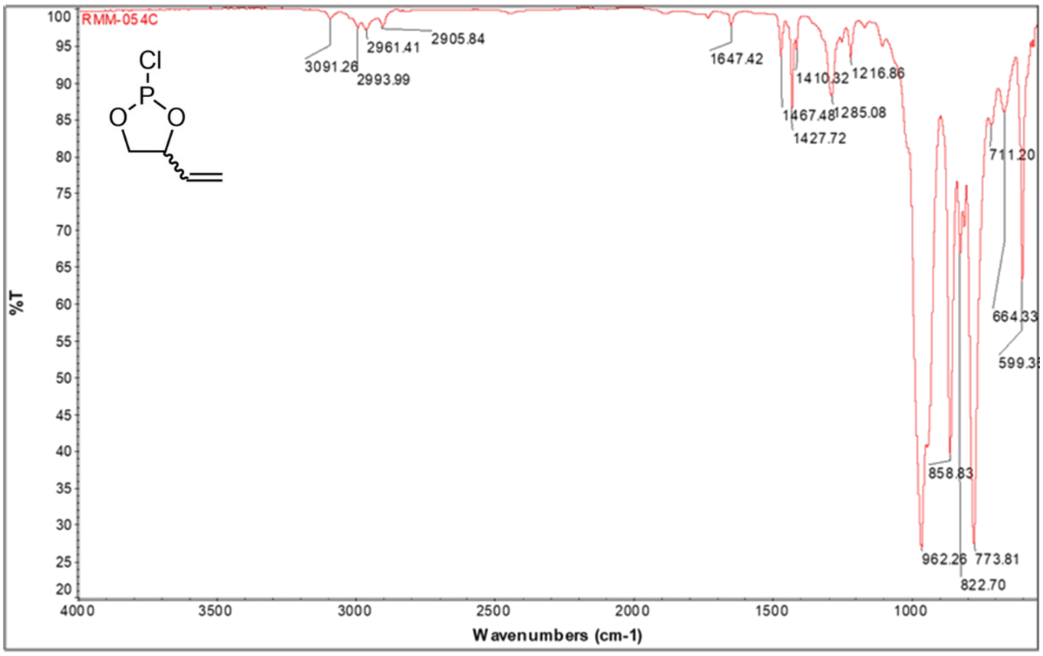

Figure S35. Infrared spectrum (ATR) of 2-chloro-4-vinyl-1,3,2-dioxaphospholane (mixture of stereoisomers).

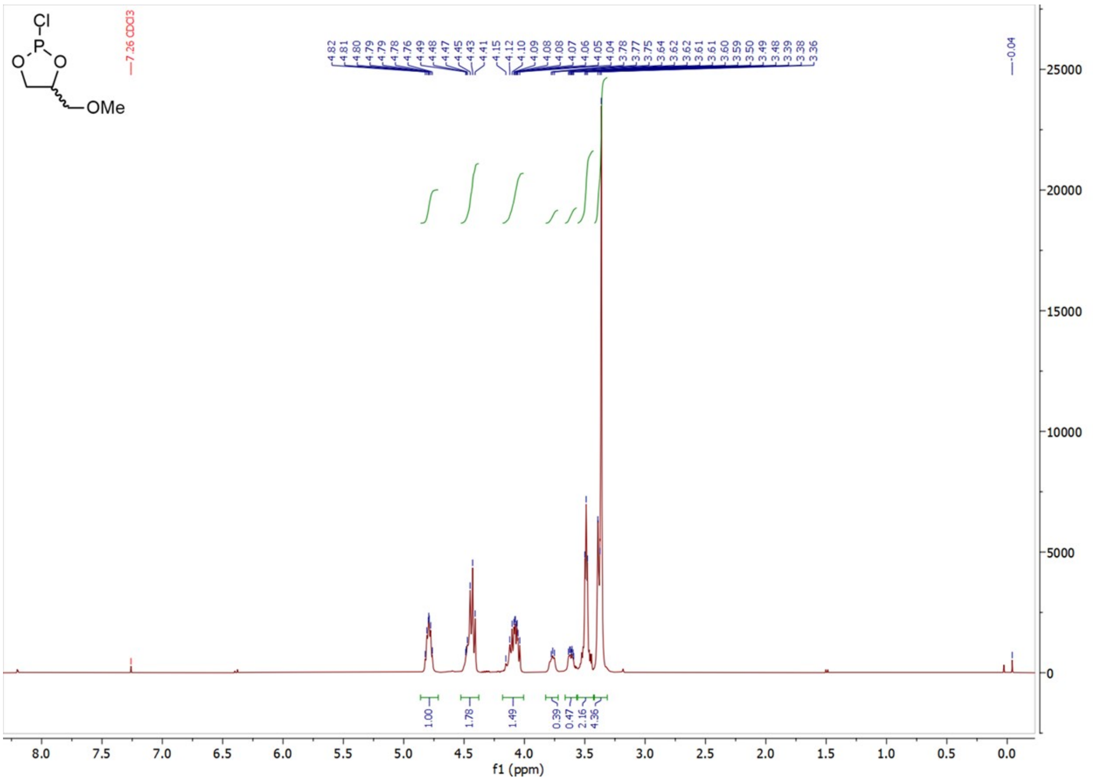

Figure S36. ^{1}H NMR spectrum (400 MHz) of 2-chloro-4-(methoxymethyl)-1,3,2-dioxaphospholane (mixture of stereoisomers) in $CDCl_3$.

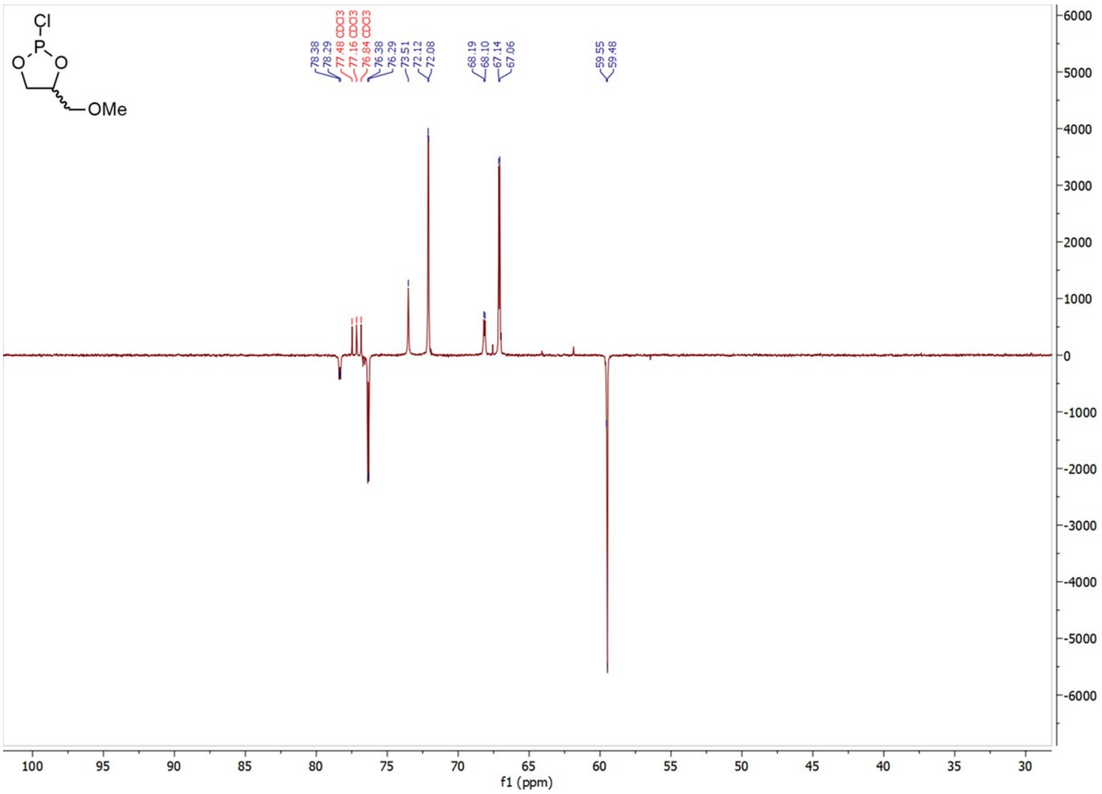

Figure S37. ^{13}C APT NMR spectrum (100.6 MHz) of 2-chloro-4-(methoxymethyl)-1,3,2-dioxaphospholane (mixture of stereoisomers) in $CDCl_3$.

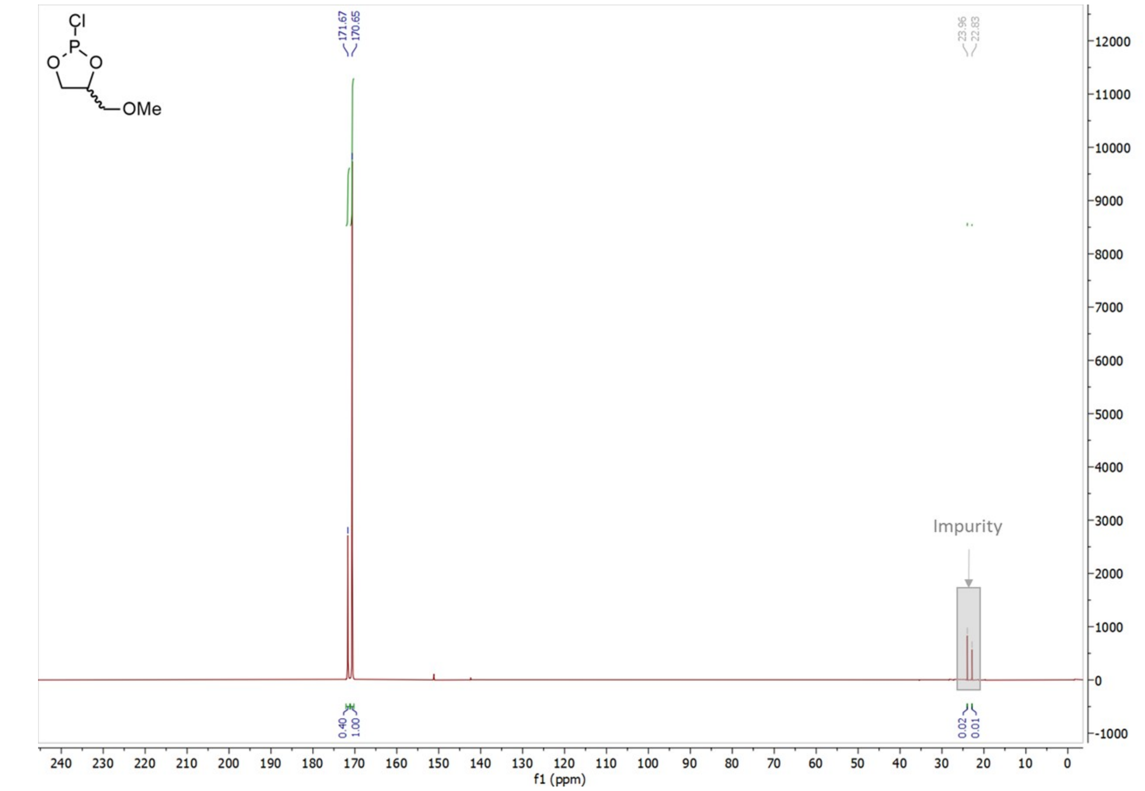

Figure S38. ^{31}P NMR spectrum (162 MHz) of 2-chloro-4-(methoxymethyl)-1,3,2-dioxaphospholane (mixture of stereoisomers) in $CDCl_3$.

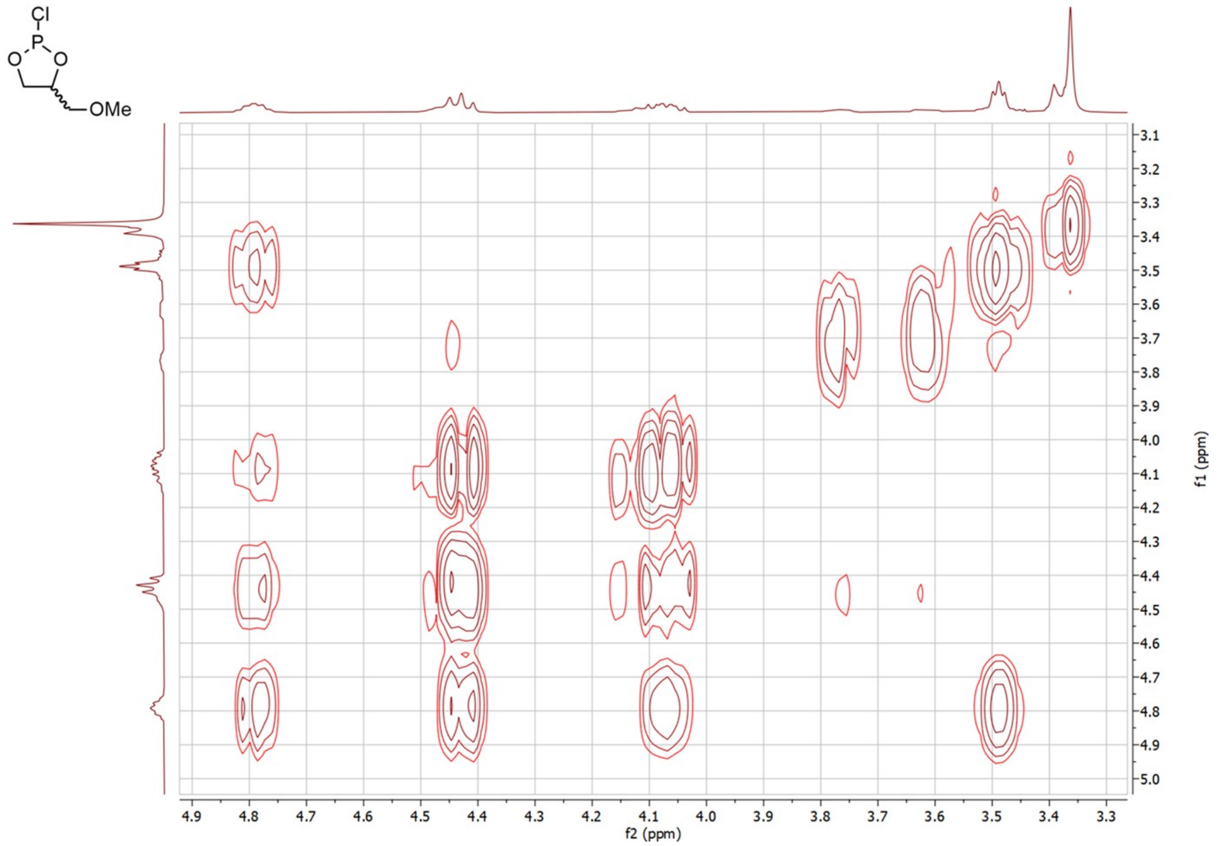

Figure S39. COSY NMR spectrum of 2-chloro-4-(methoxymethyl)-1,3,2-dioxaphospholane (mixture of stereoisomers) in $CDCl_3$.

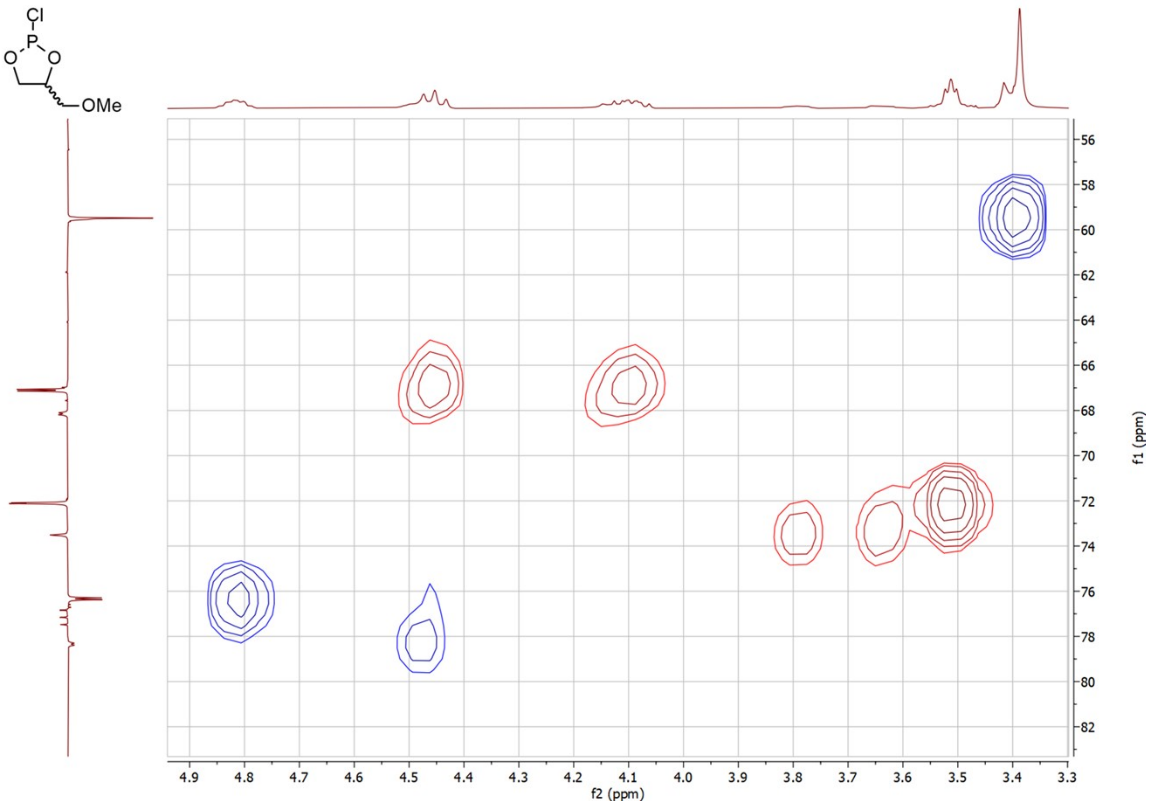

Figure S40. HSQC NMR spectrum of 2-chloro-4-(methoxymethyl)-1,3,2-dioxaphospholane (mixture of stereoisomers) in $CDCl_3$.

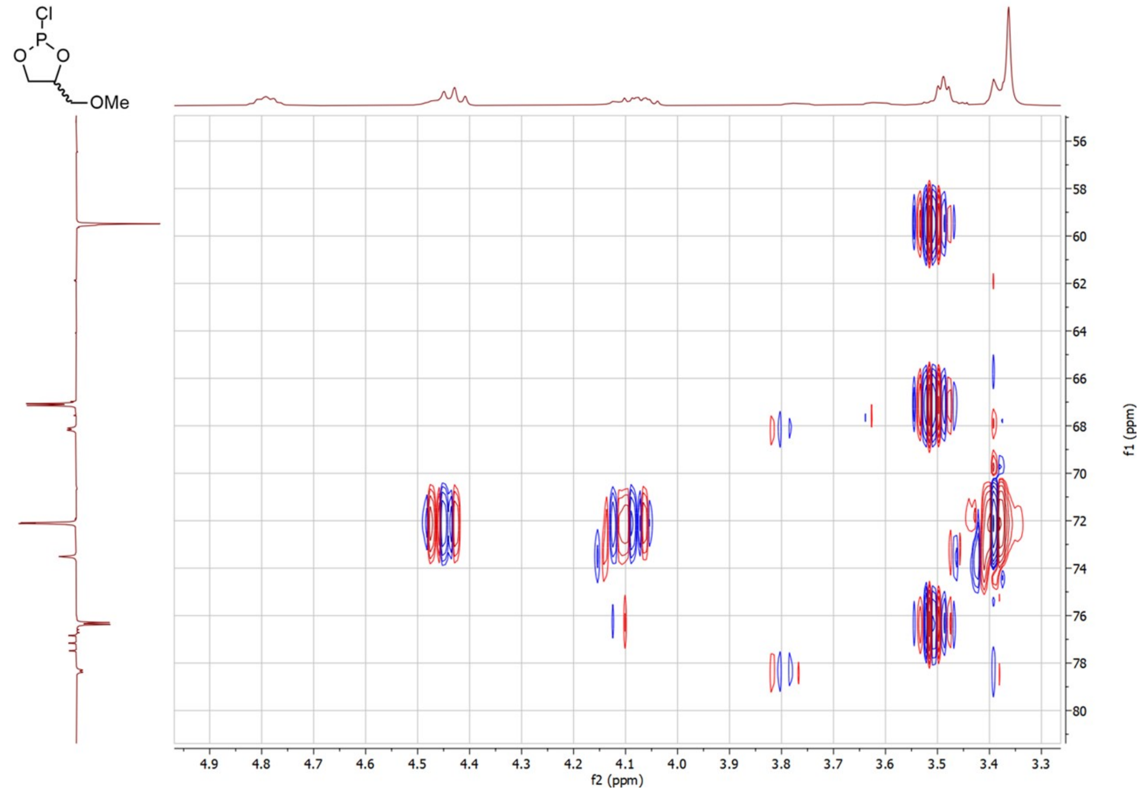

Figure S41. HMBC NMR spectrum of 2-chloro-4-(methoxymethyl)-1,3,2-dioxaphospholane (mixture of stereoisomers) in $CDCl_3$.

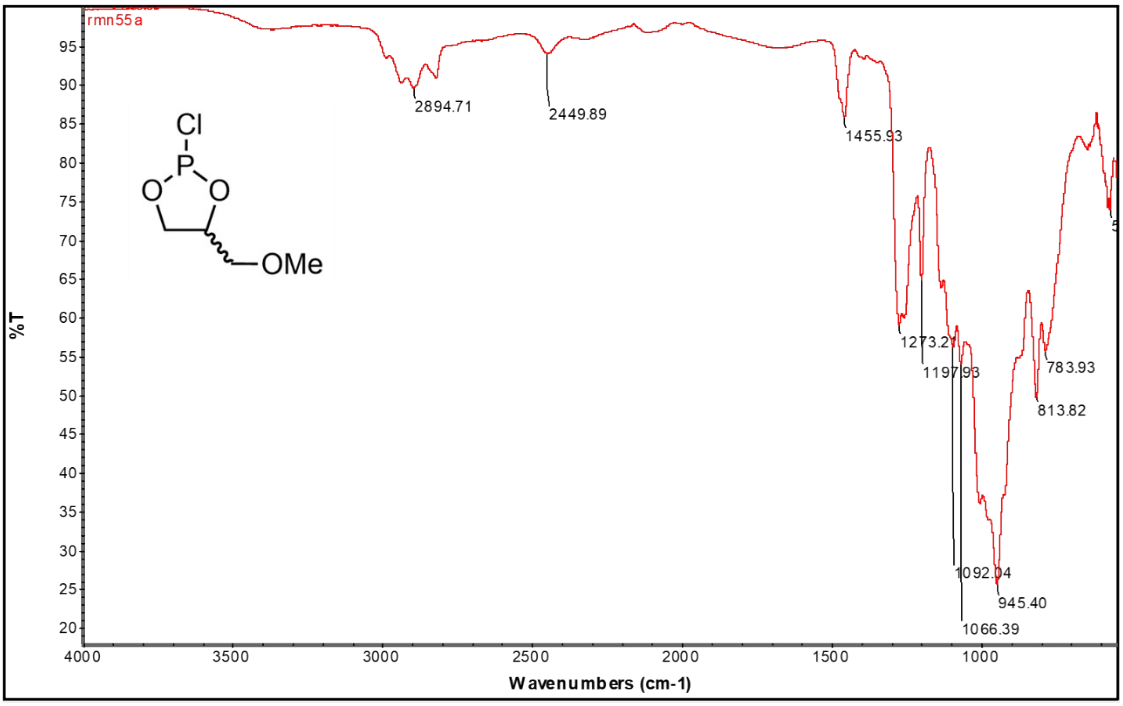

Figure S42. Infrared spectrum (ATR) of 2-chloro-4-(methoxymethyl)-1,3,2-dioxaphospholane (mixture of stereoisomers).

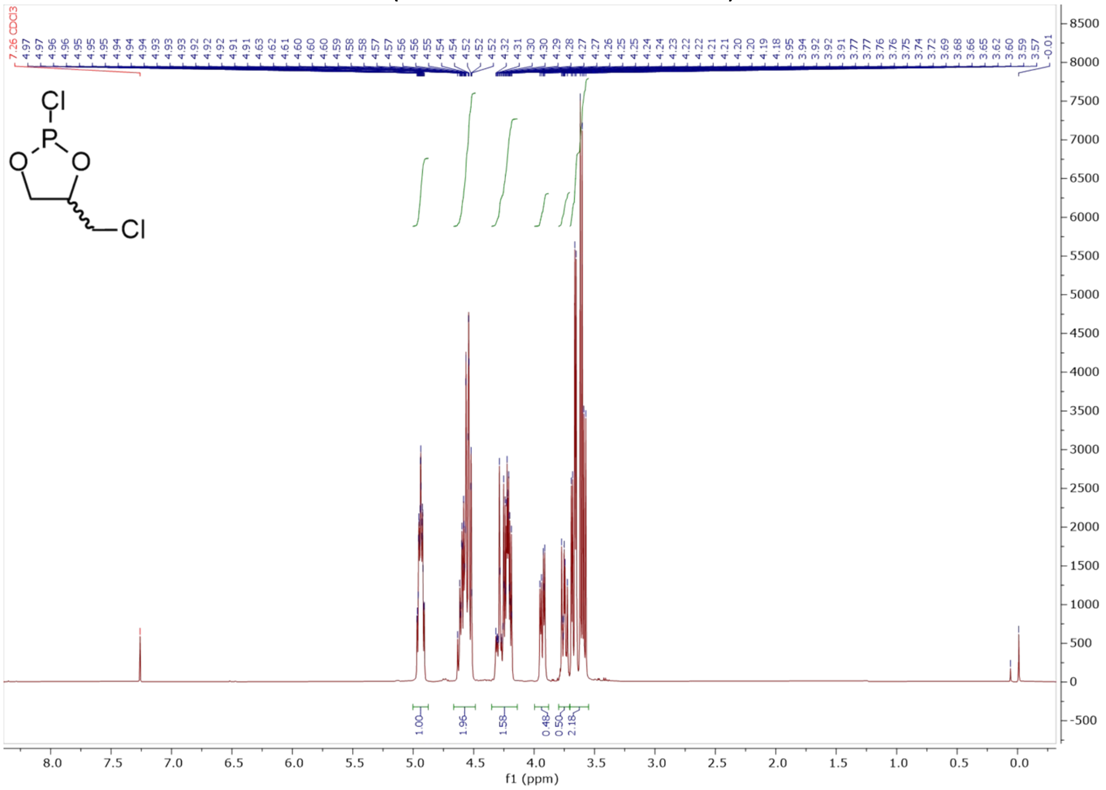

Figure S43. ^{1}H NMR spectrum (400 MHz) of 2-chloro-4-(chloromethyl)-1,3,2-dioxaphospholane (mixture of stereoisomers) in $CDCl_3$.

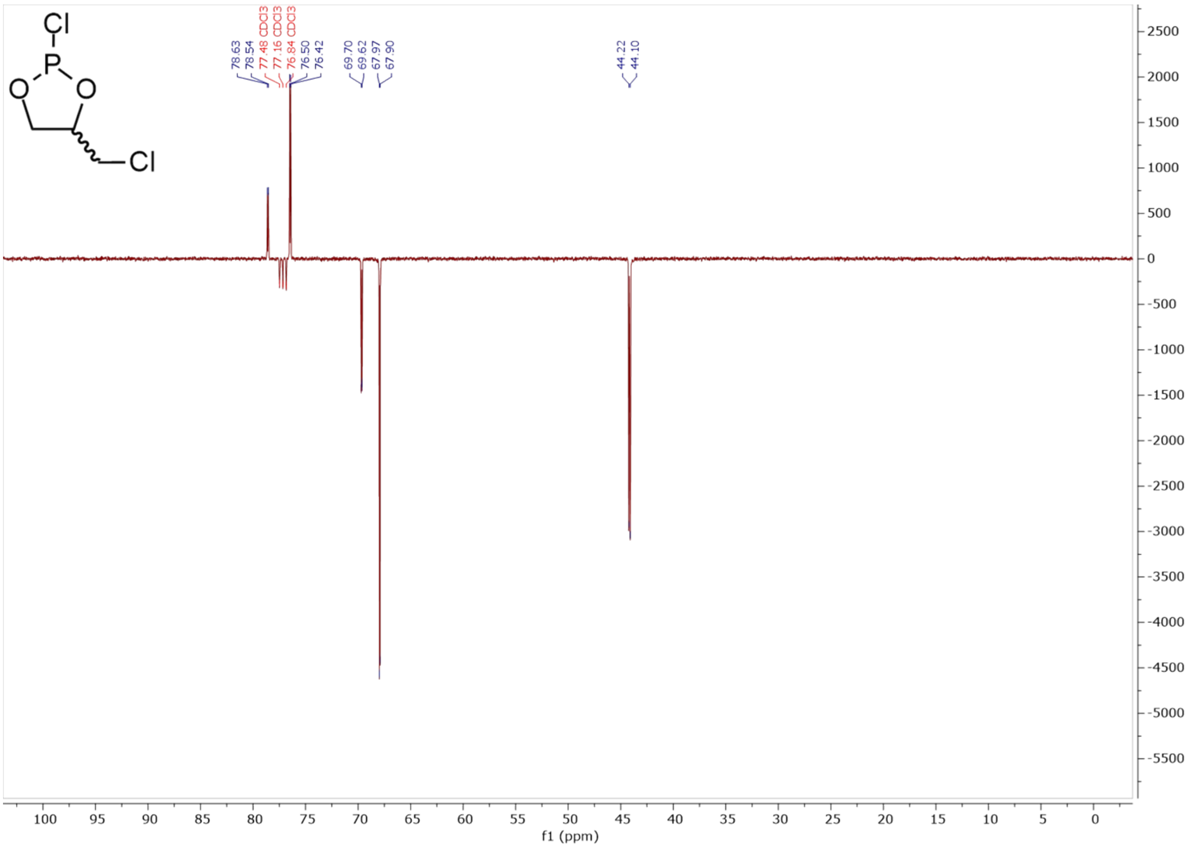

Figure S44. ^{13}C APT NMR spectrum (100.6 MHz) of 2-chloro-4-(chloromethyl)-1,3,2-dioxaphospholane (mixture of stereoisomers) in $CDCl_3$.

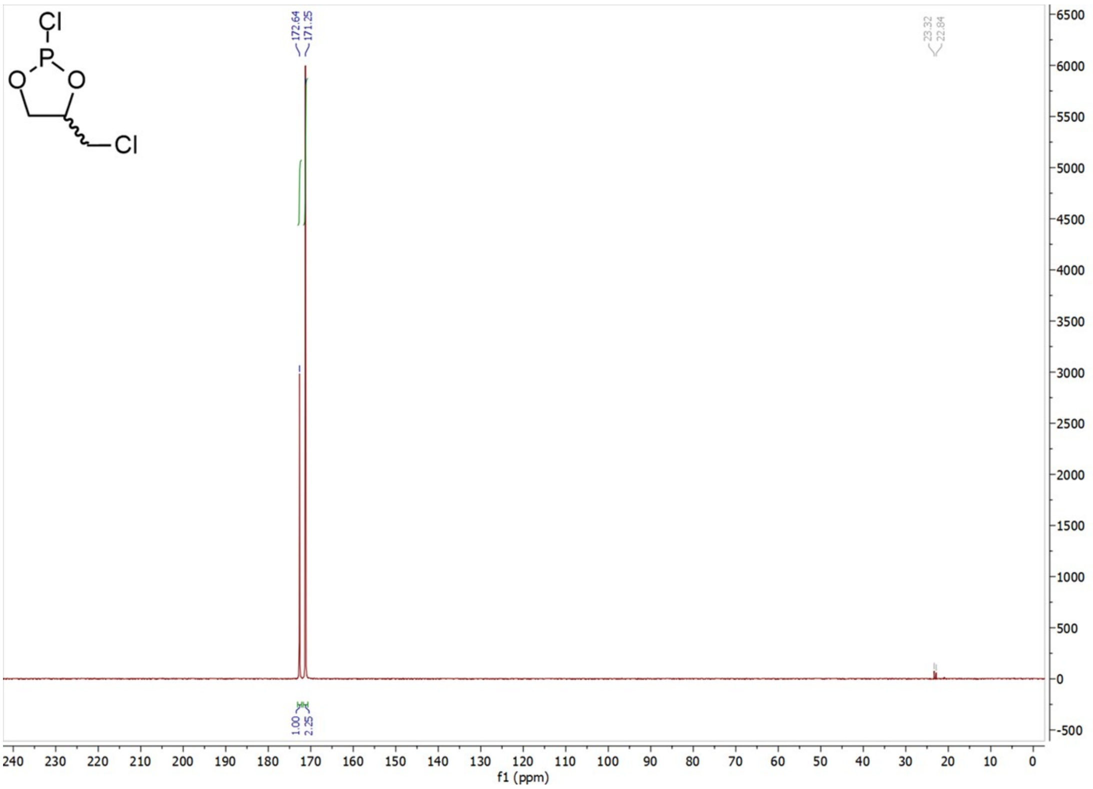

Figure S45. ^{31}P NMR spectrum (162 MHz) of 2-chloro-4-(chloromethyl)-1,3,2-dioxaphospholane (mixture of stereoisomers) in $CDCl_3$.

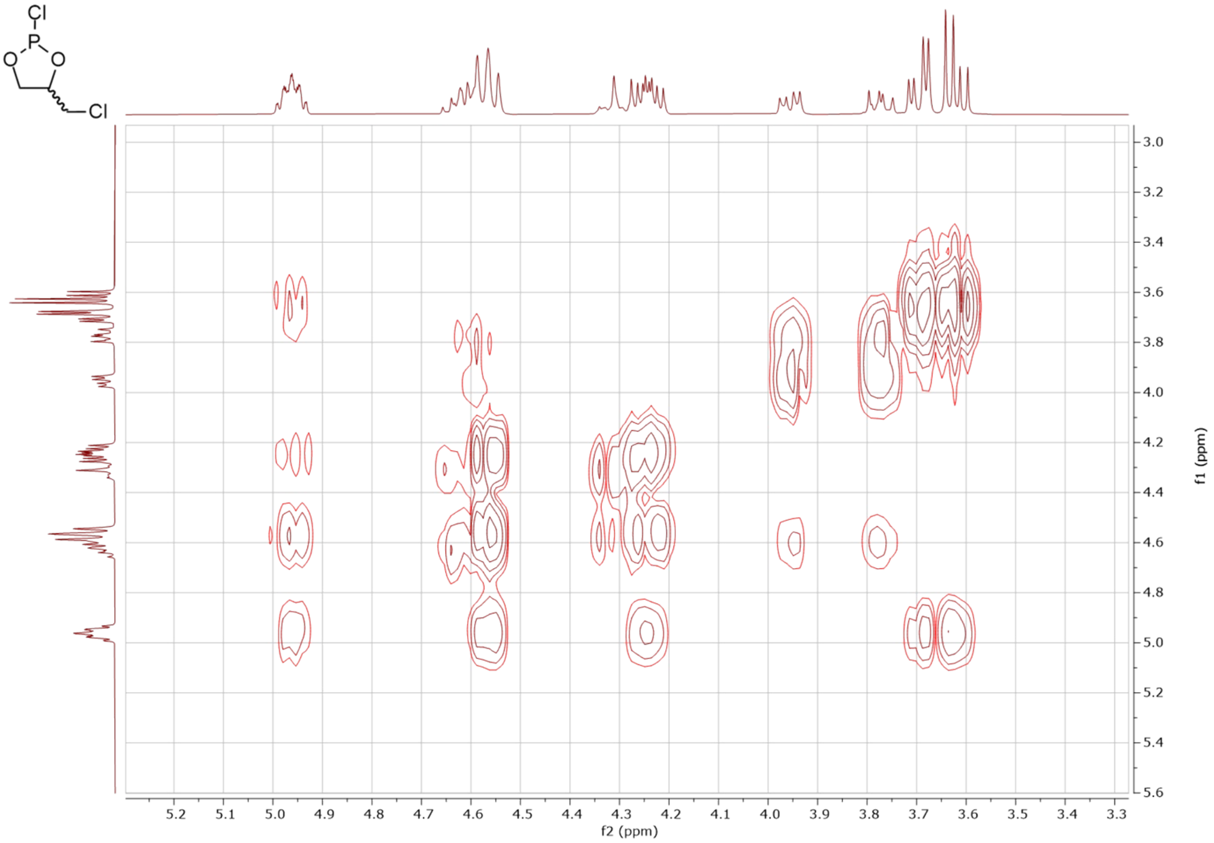

Figure S46. COSY NMR spectrum of 2-chloro-4-(chloromethyl)-1,3,2-dioxaphospholane (mixture of stereoisomers) in $CDCl_3$.

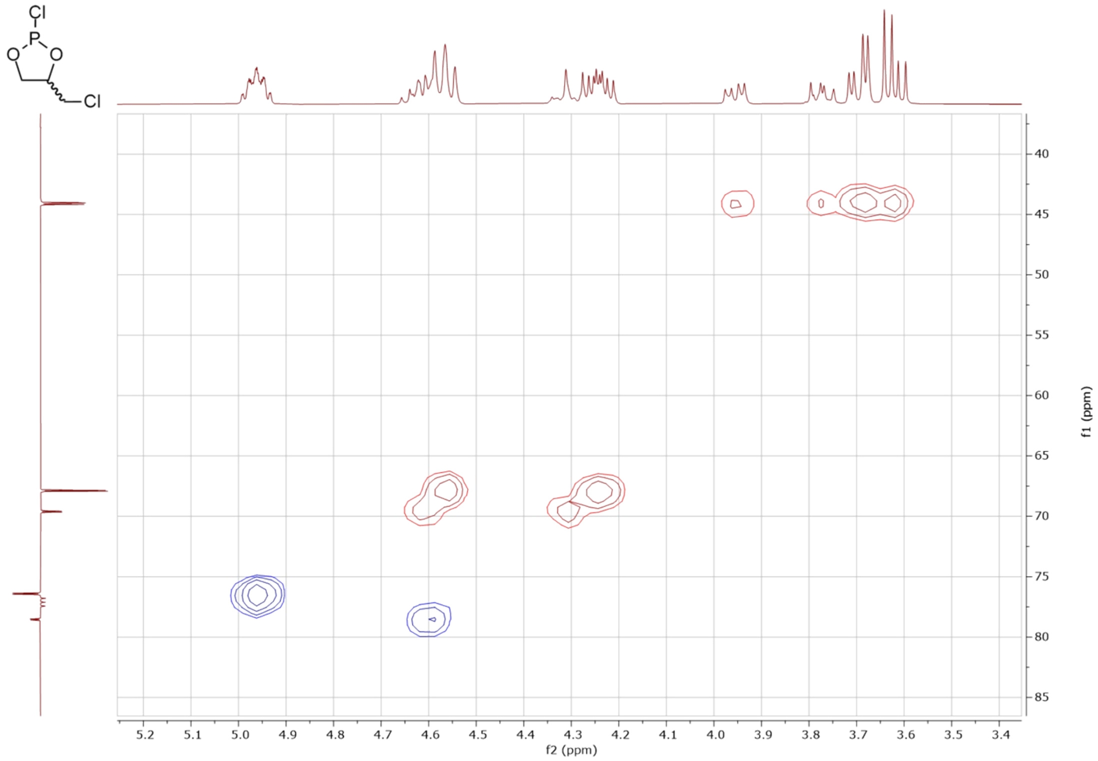

Figure S47. HSQC NMR spectrum of 2-chloro-4-(chloromethyl)-1,3,2-dioxaphospholane (mixture of stereoisomers) in $CDCl_3$.

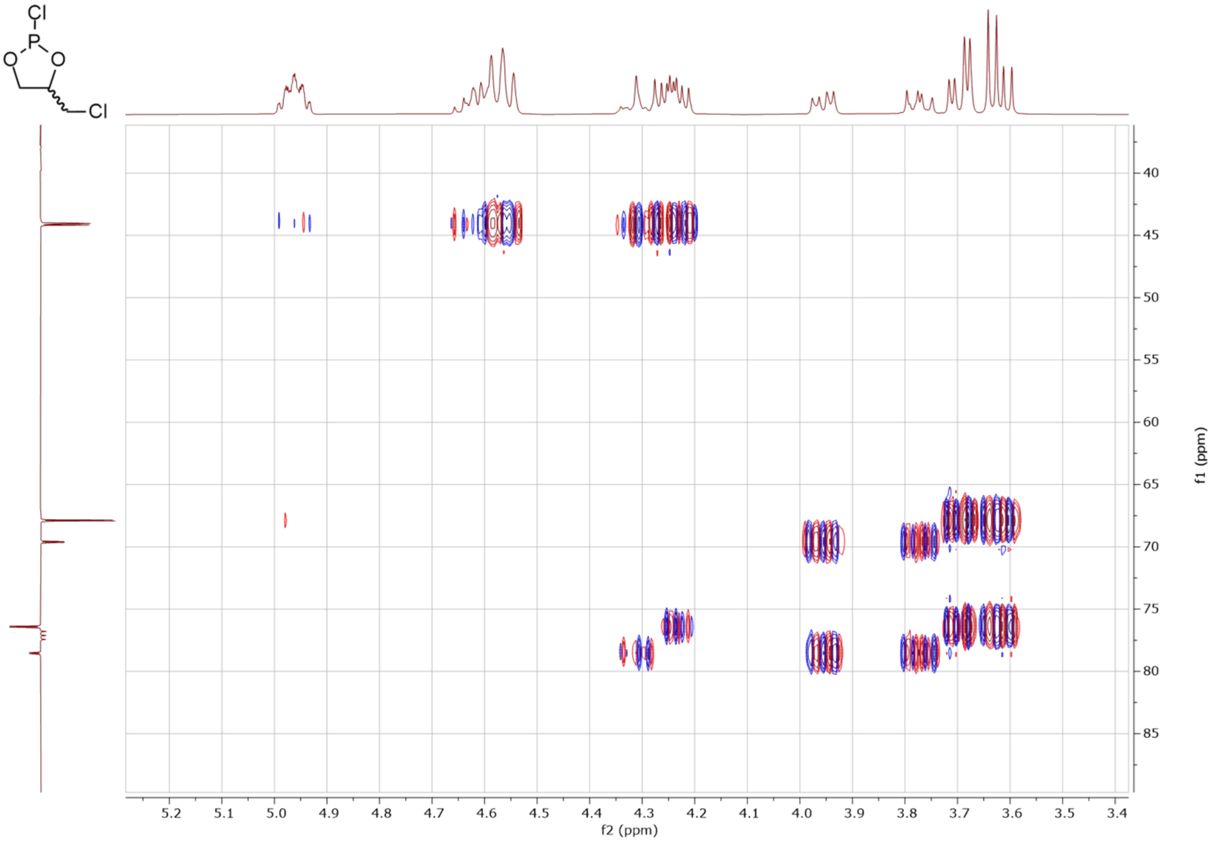

Figure S48. HMBC NMR spectrum of 2-chloro-4-(chloromethyl)-1,3,2-dioxaphospholane (mixture of stereoisomers) in $CDCl_3$.

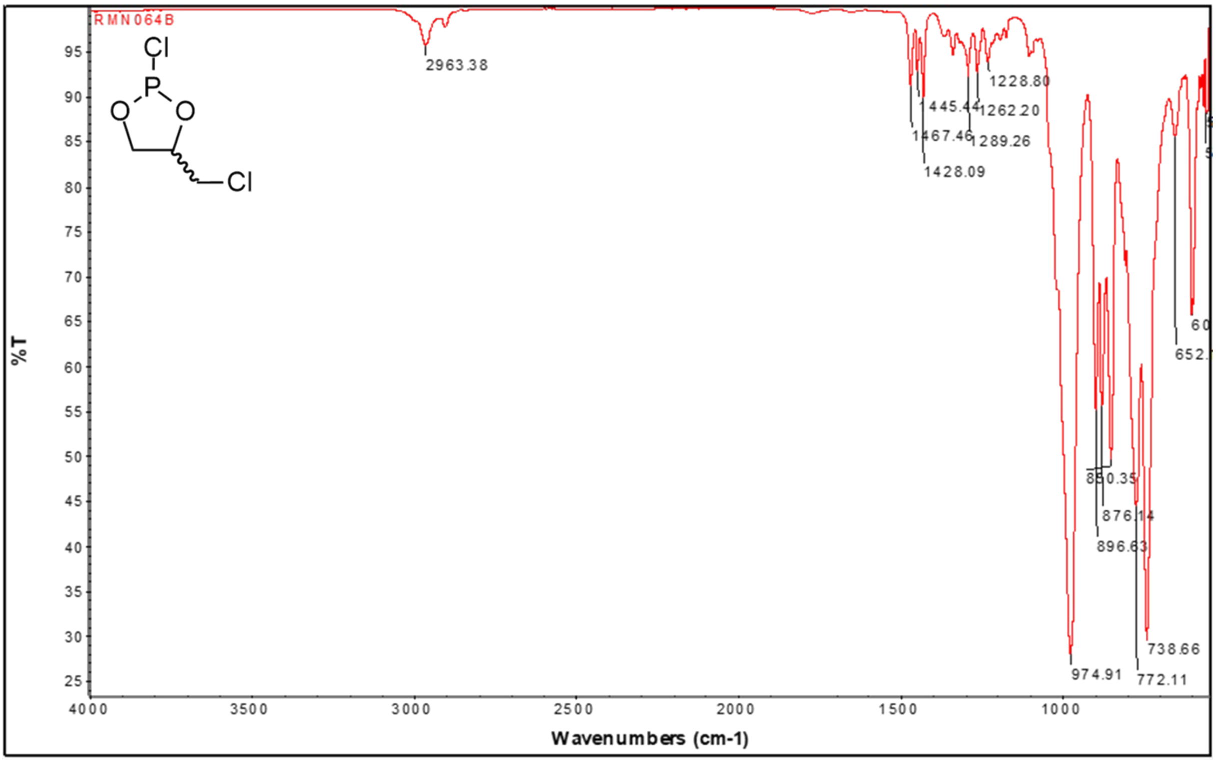

Figure S49. Infrared (ATR) spectrum of 2-chloro-4-(chloromethyl)-1,3,2-dioxaphospholane (mixture of stereoisomers).

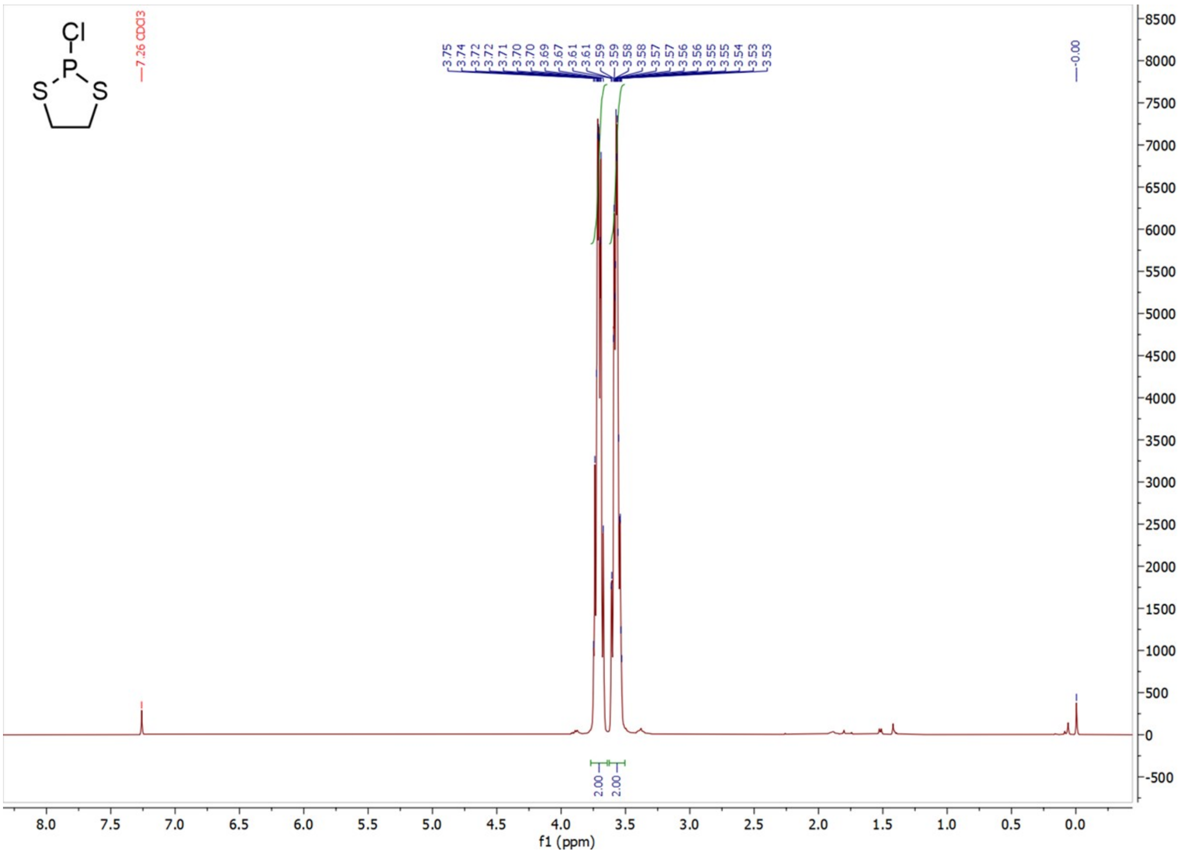

Figure S50. ^{1}H NMR spectrum (400 MHz) of 2-chloro-1,3,2-dithiaphospholane in $CDCl_3$.

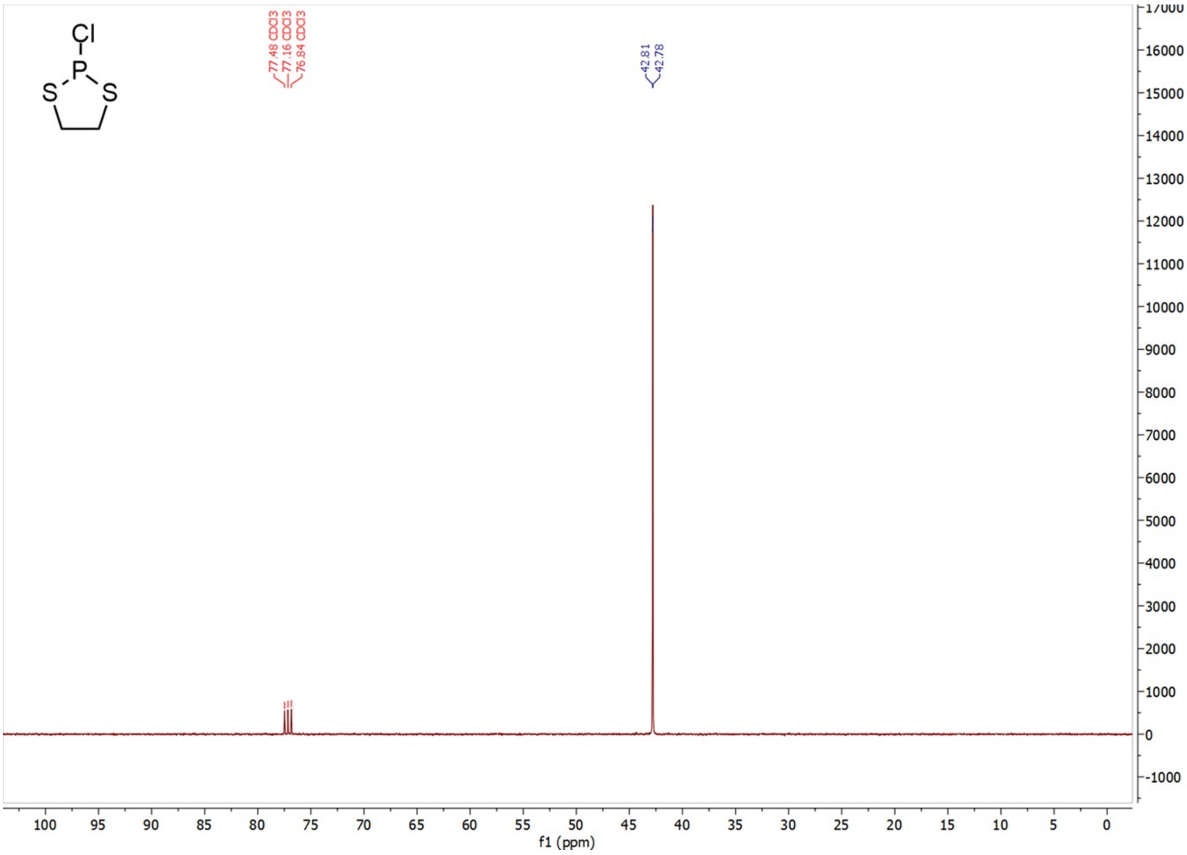

Figure S51. ^{13}C APT NMR spectrum (100.6 MHz) of 2-chloro-1,3,2-dithiaphospholane in $CDCl_3$.

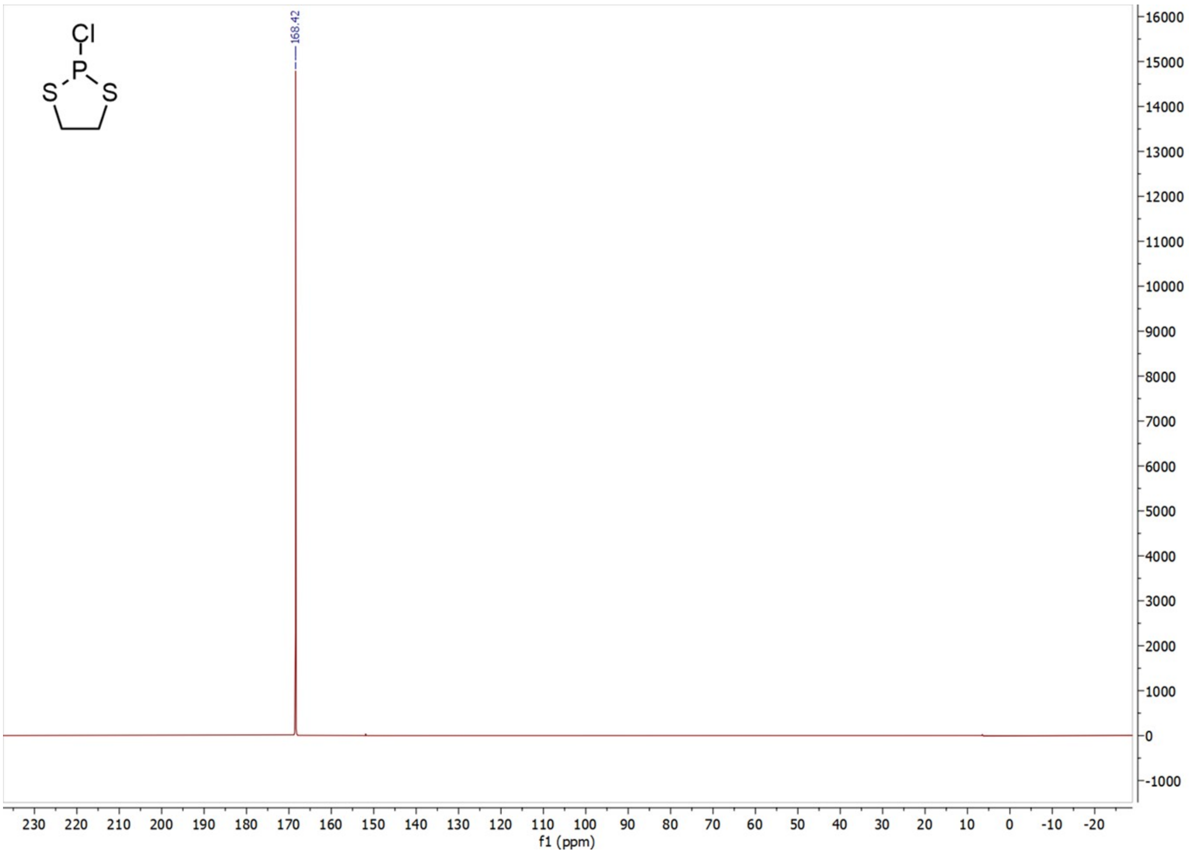

Figure S52. ^{31}P NMR spectrum (162 MHz) of 2-chloro-1,3,2-dithiaphospholane in $CDCl_3$.

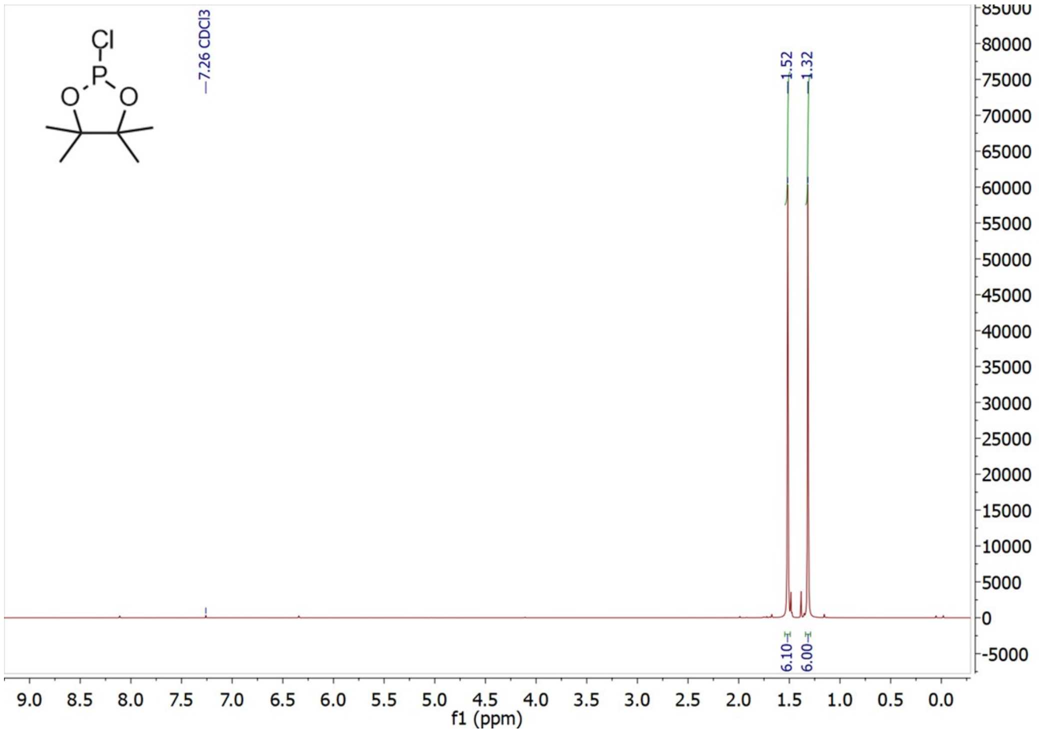

Figure S53. ^{1}H NMR spectrum (400 MHz) of 2-chloro-4,4,5,5-tetramethyl-1,3,2-dioxaphospholane in $CDCl_3$.

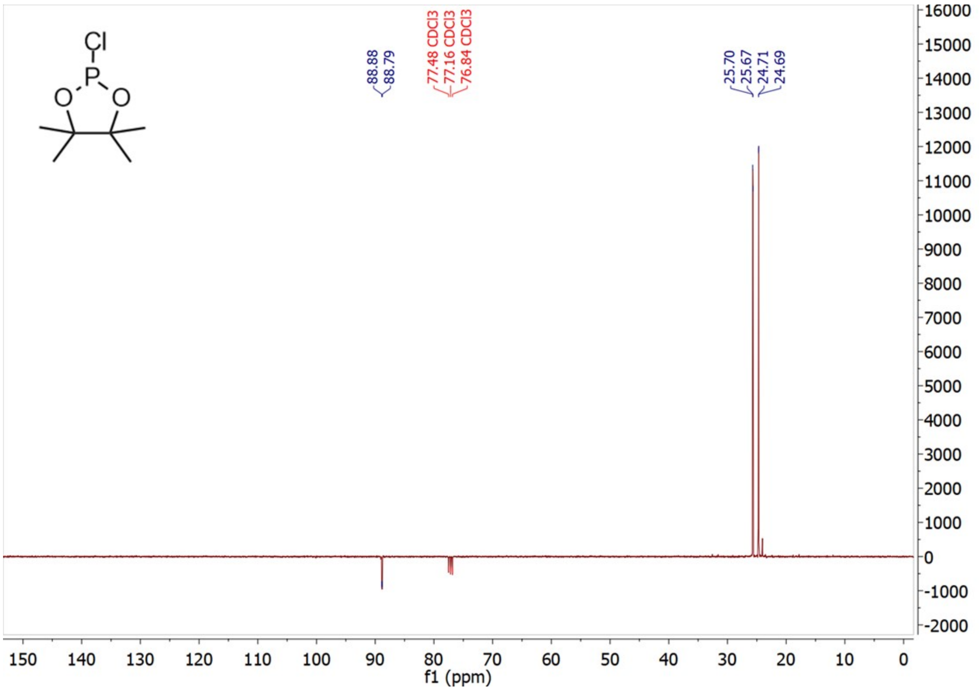

Figure S54. ^{13}C APT NMR spectrum (100.6 MHz) of 2-chloro-4,4,5,5-tetramethyl-1,3,2-dioxaphospholane in $CDCl_3$.

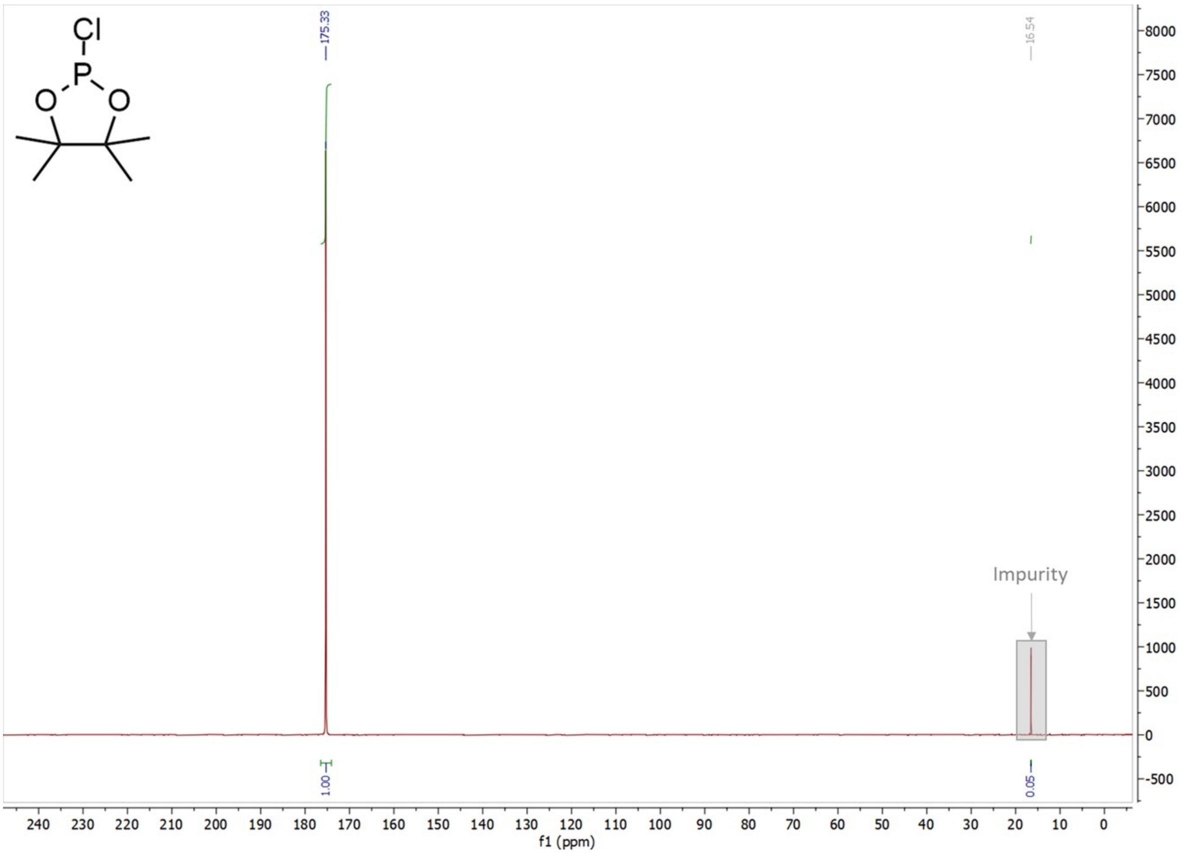

Figure S55. ^{31}P NMR spectrum (162 MHz) of 2-chloro-4,4,5,5-tetramethyl-1,3,2-dioxaphospholane in $CDCl_3$.

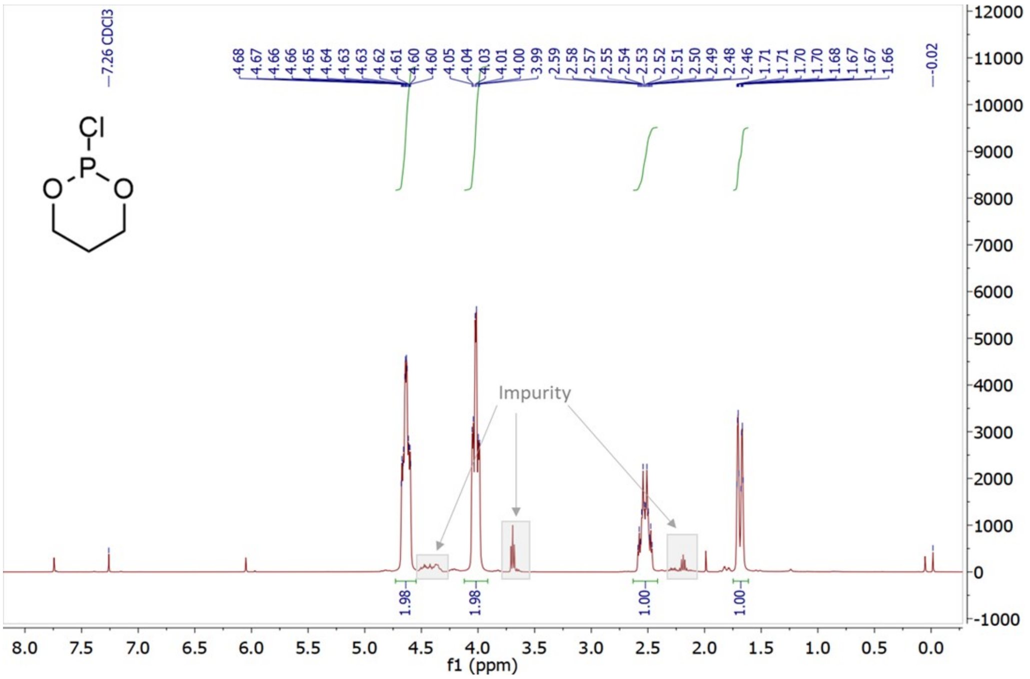

Figure S56. ^{1}H NMR spectrum (400 MHz) of 2-chloro-1,3,2-dioxaphosphinane in $CDCl_3$.

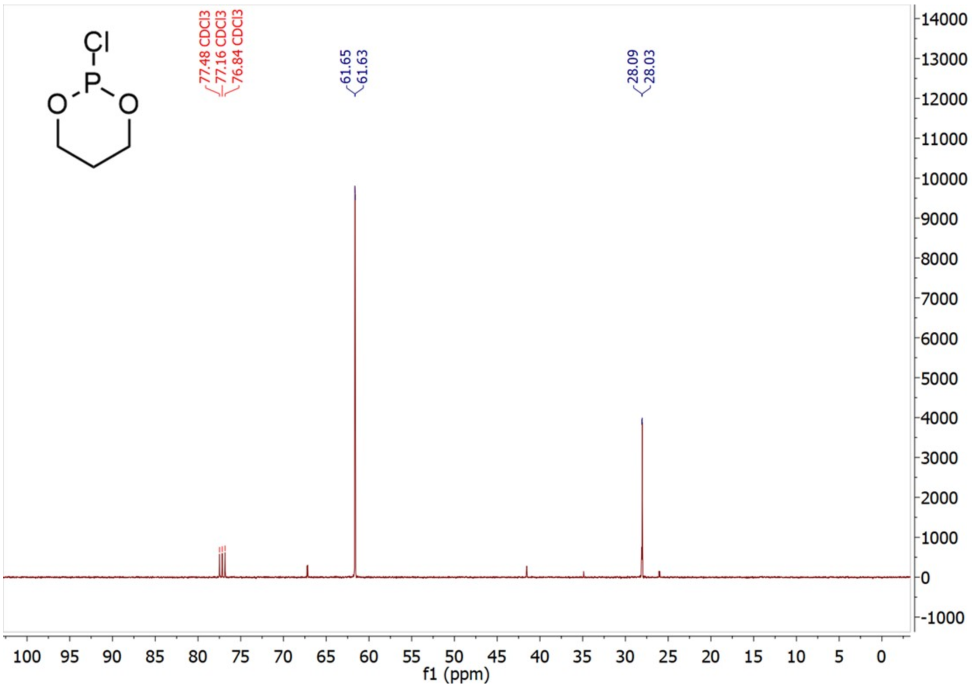

Figure S57. ^{13}C APT NMR spectrum (100.6 MHz) of 2-chloro-1,3,2-dioxaphosphinane in $CDCl_3$.

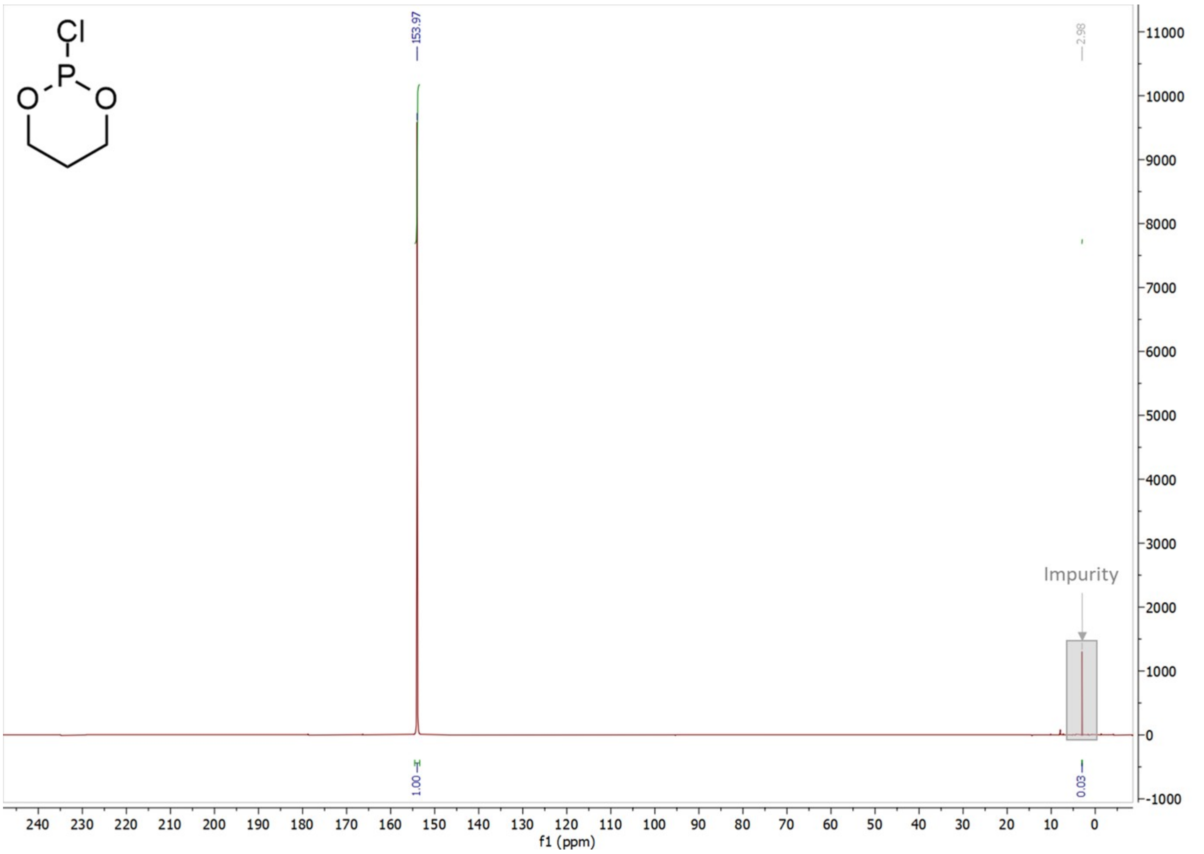

Figure S58. ^{31}P NMR spectrum (162 MHz) of 2-chloro-1,3,2-dioxaphosphinane in $CDCl_3$.

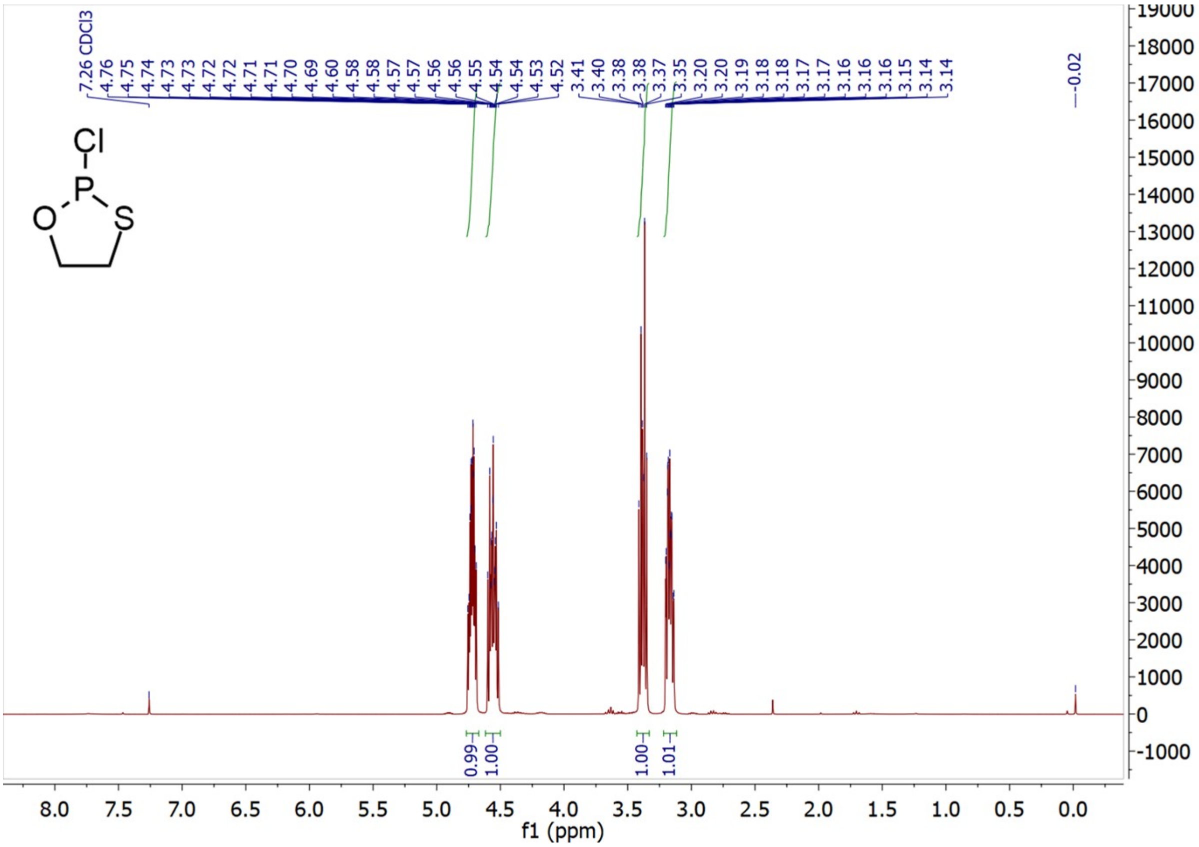

Figure S59. ^{1}H NMR spectrum (400 MHz) of 2-chloro-1,3,2-oxathiaphospholane in $CDCl_3$.

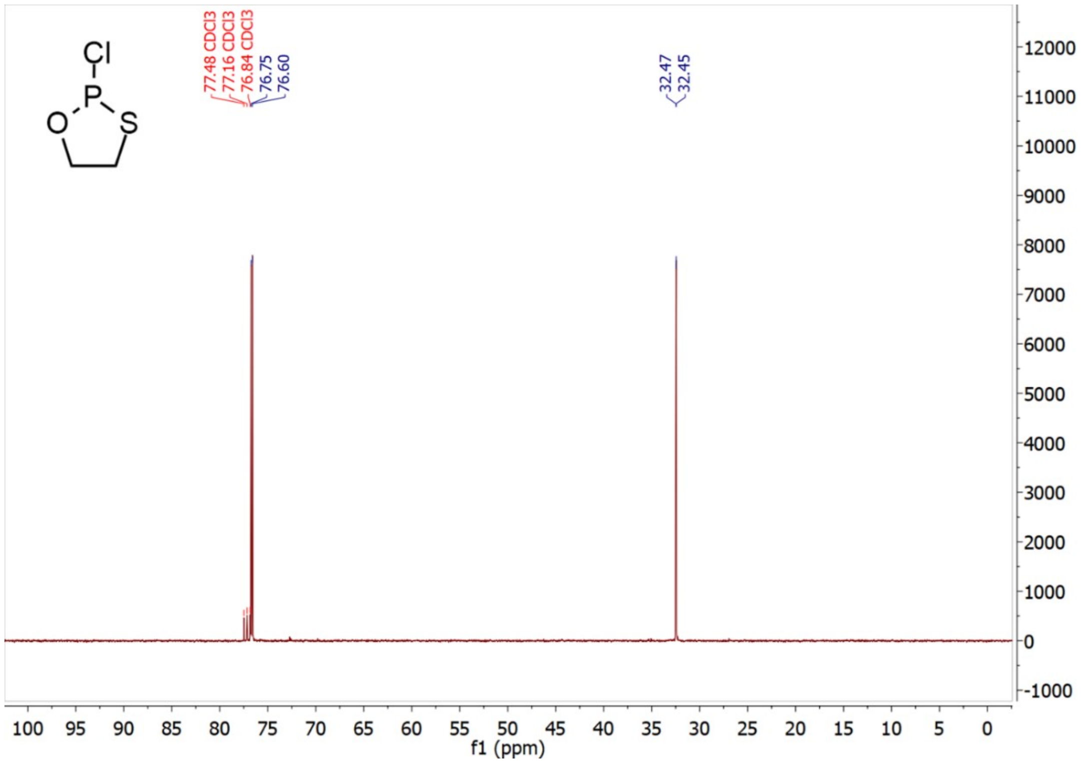

Figure S60. ^{13}C APT NMR spectrum (100.6 MHz) of 2-chloro-1,3,2-oxathiaphospholane in $CDCl_3$.

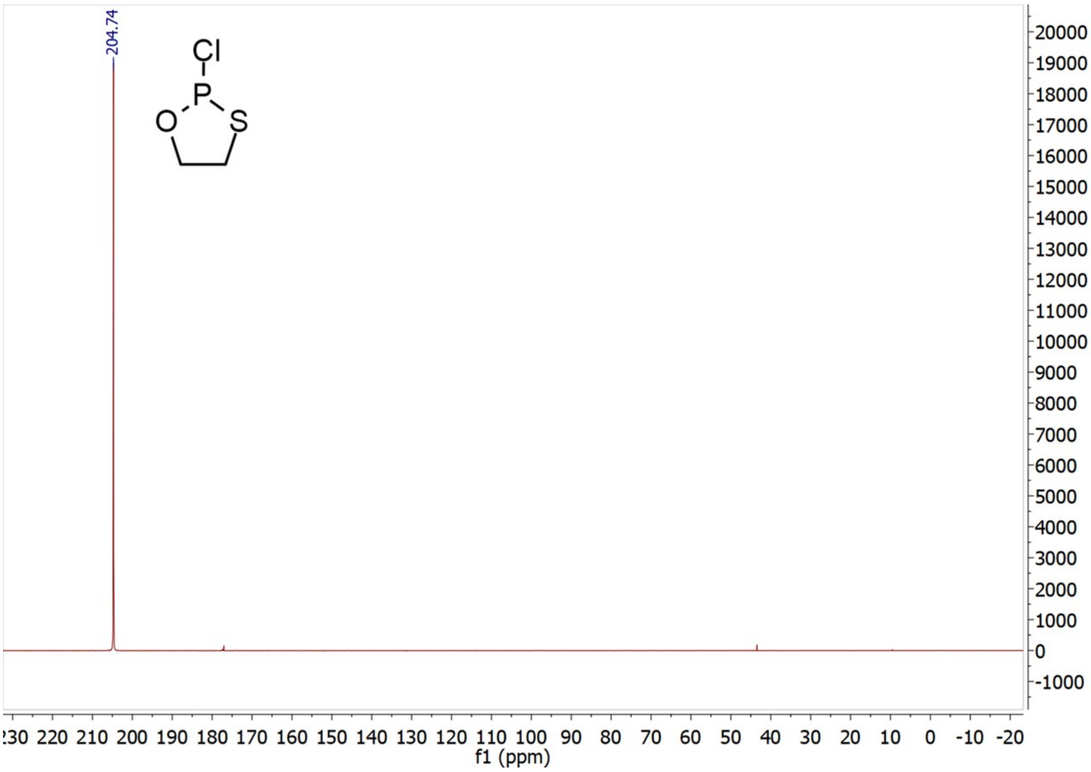

Figure S61. ^{31}P NMR spectrum (162 MHz) of 2-chloro-1,3,2-oxathiaphospholane in $CDCl_3$.

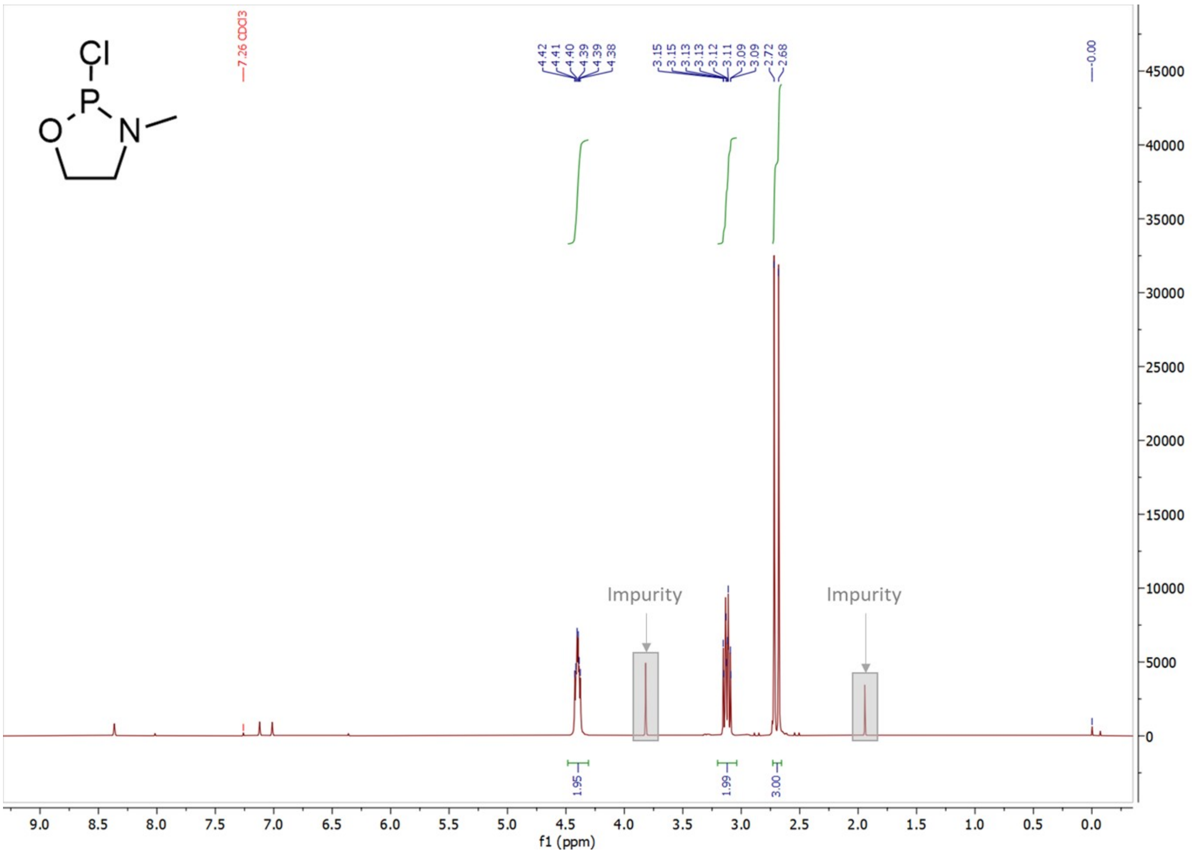

Figure S62. ^{1}H NMR spectrum (400 MHz) of 2-chloro-3-methyl-1,3,2-oxazaphospholidine in $CDCl_3$.

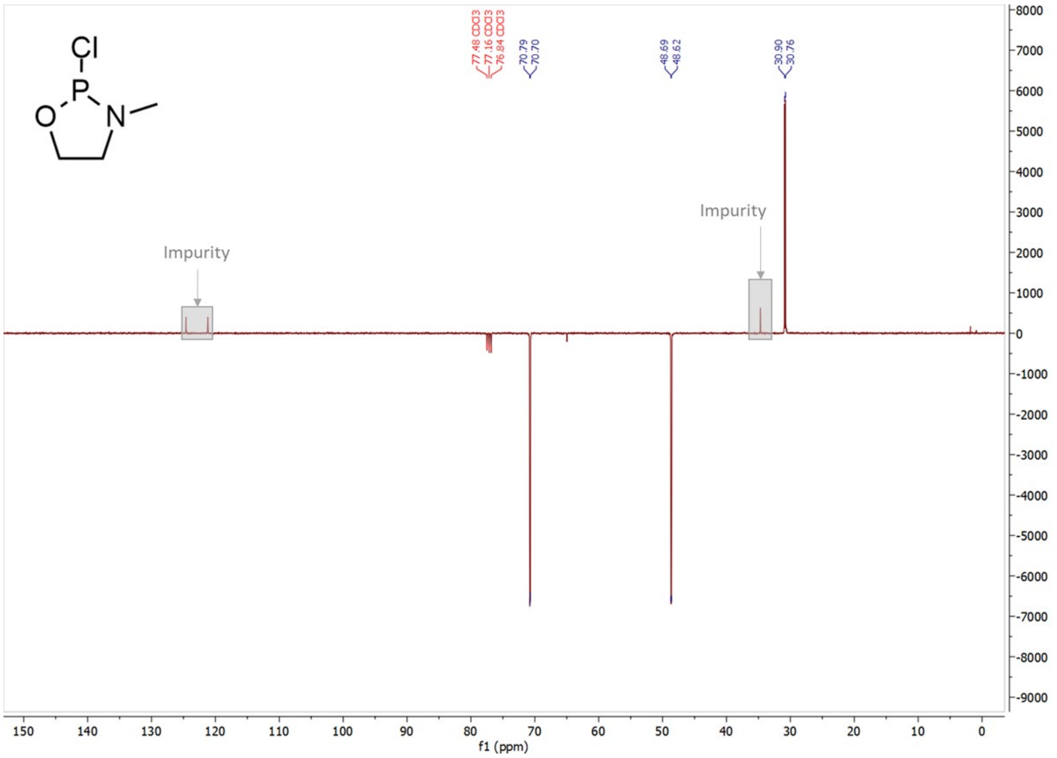

Figure S63. ^{13}C APT NMR spectrum (100.6 MHz) of 2-chloro-3-methyl-1,3,2 oxazaphospholidine in $CDCl_3$.

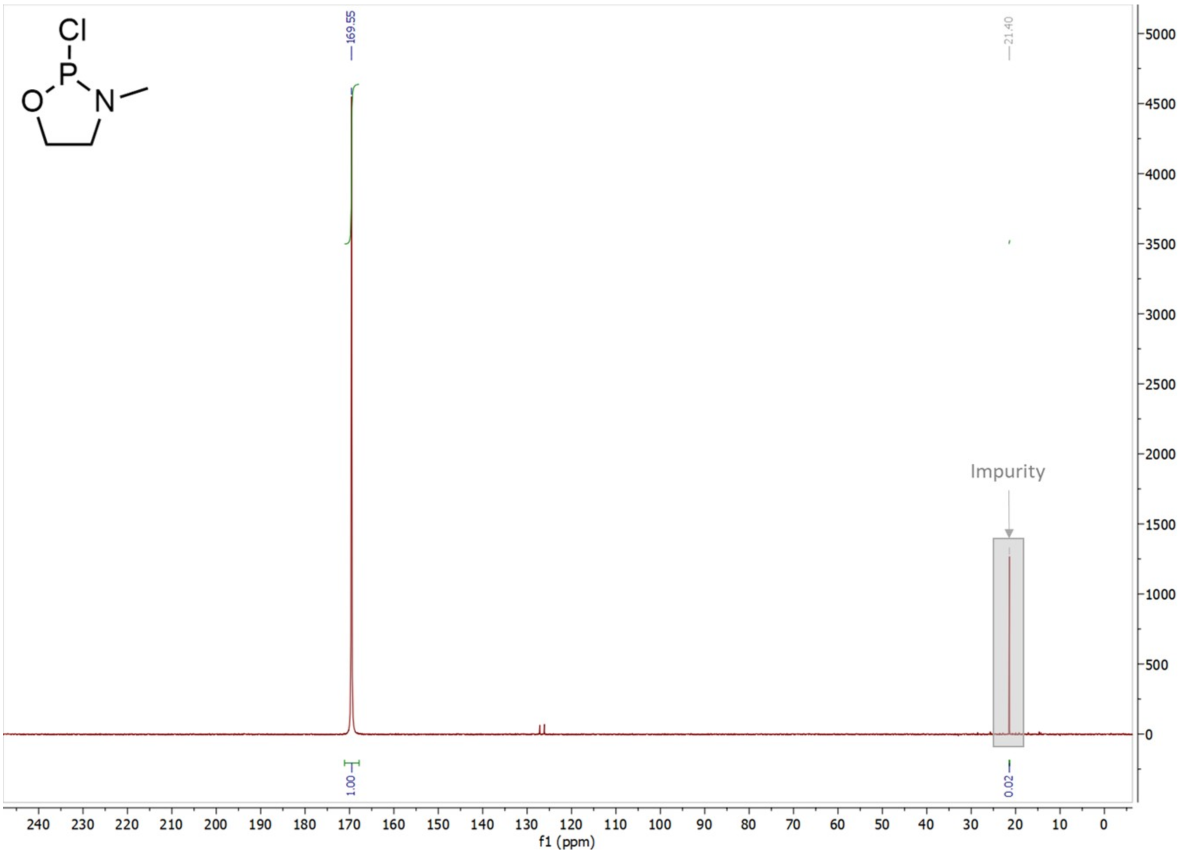

Figure S64. ^{31}P NMR spectrum (162 MHz) of 2-chloro-3-methyl-1,3,2-oxazaphospholidine in $CDCl_3$.

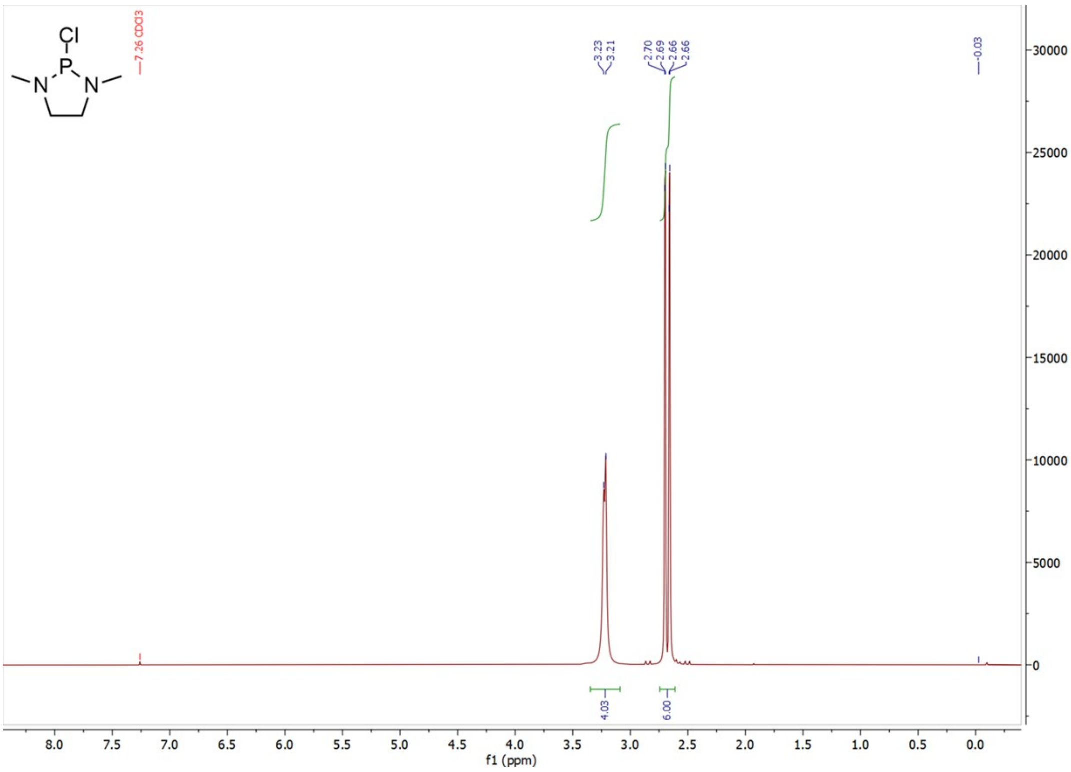

Figure S65. ^{1}H NMR spectrum (400 MHz) of 2-chloro-1,3-dimethyl-1,3,2-diazaphospholidine in $CDCl_3$.

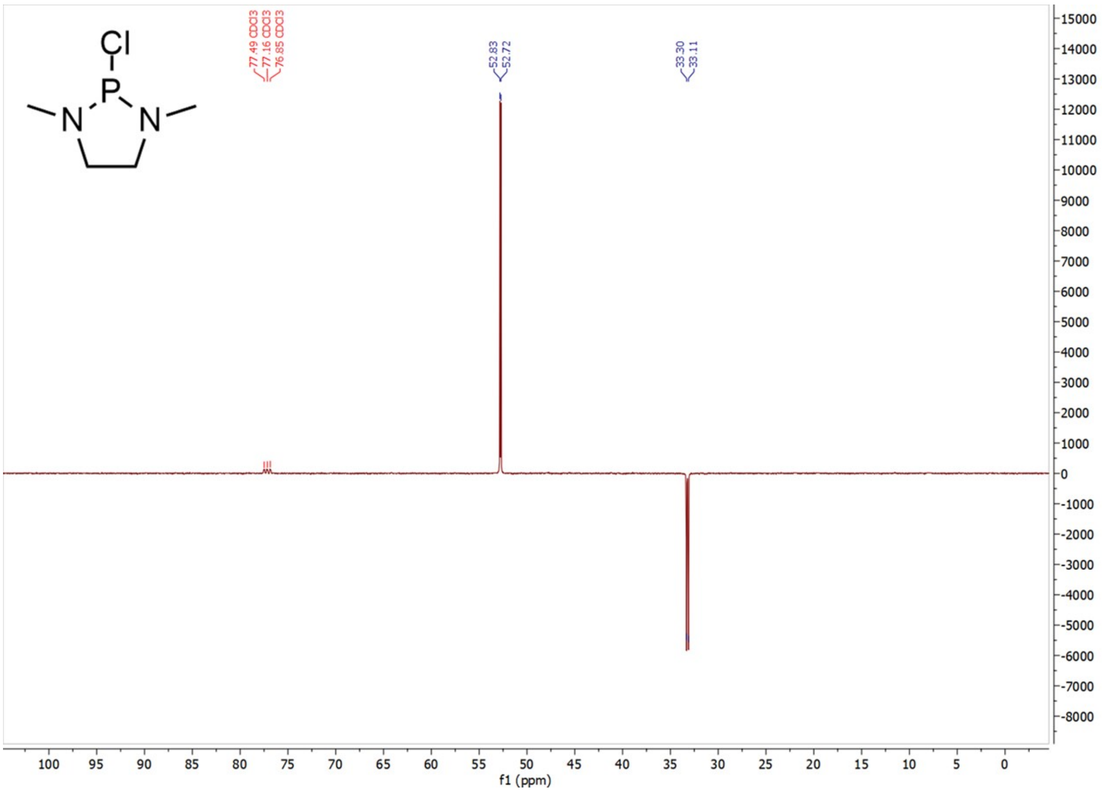

Figure S66. ^{13}C APT NMR spectrum (100.6 MHz) of 2-chloro-1,3-dimethyl-1,3,2-diazaphospholidine in $CDCl_3$

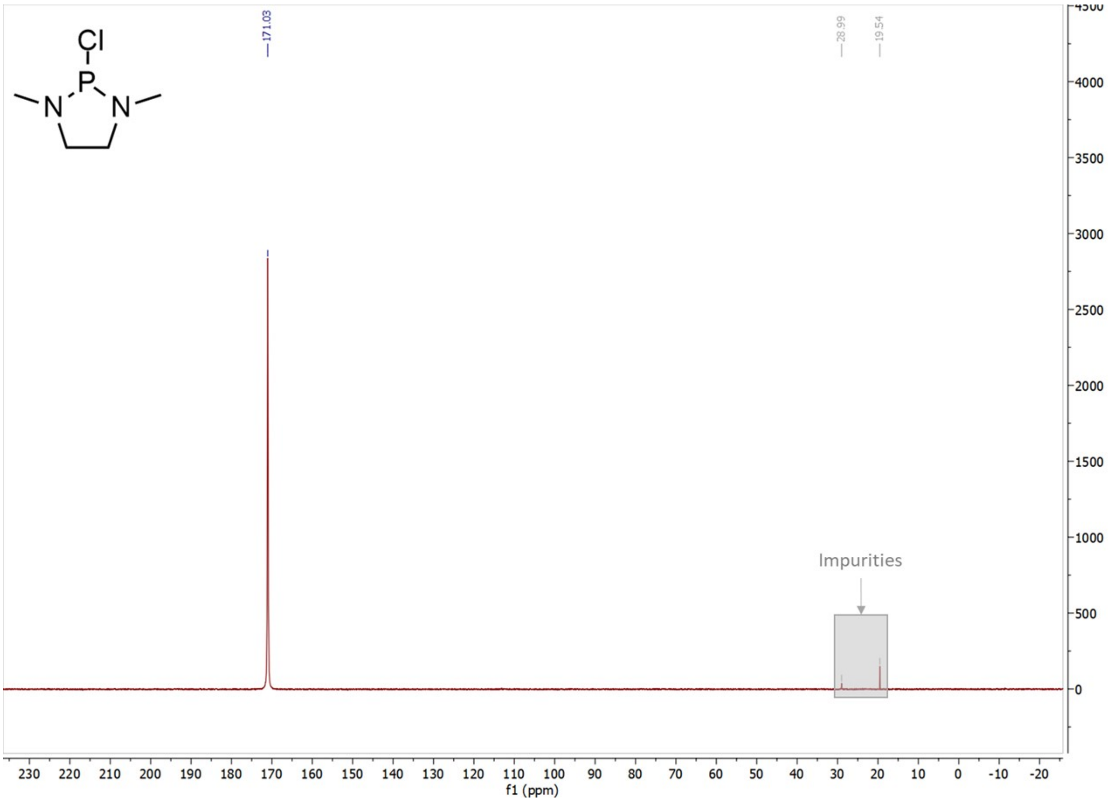

Figure S67. ^{31}P NMR spectrum (162 MHz) of 2-chloro-1,3-dimethyl-1,3,2-diazaphospholidine in $CDCl_3$.

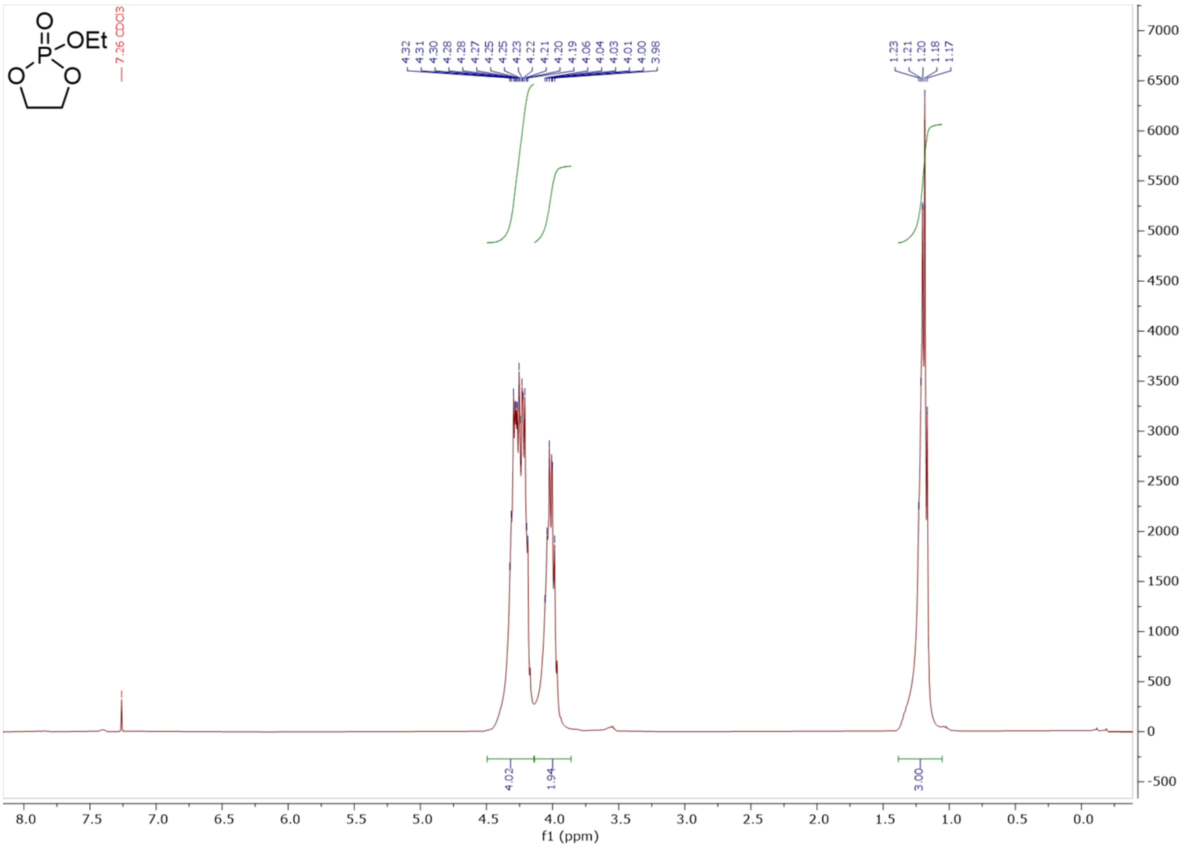

Figure S68. ^{1}H NMR spectrum (400 MHz) of 2-ethoxy-1,3,2-dioxaphospholane 2-oxide in $CDCl_3$.

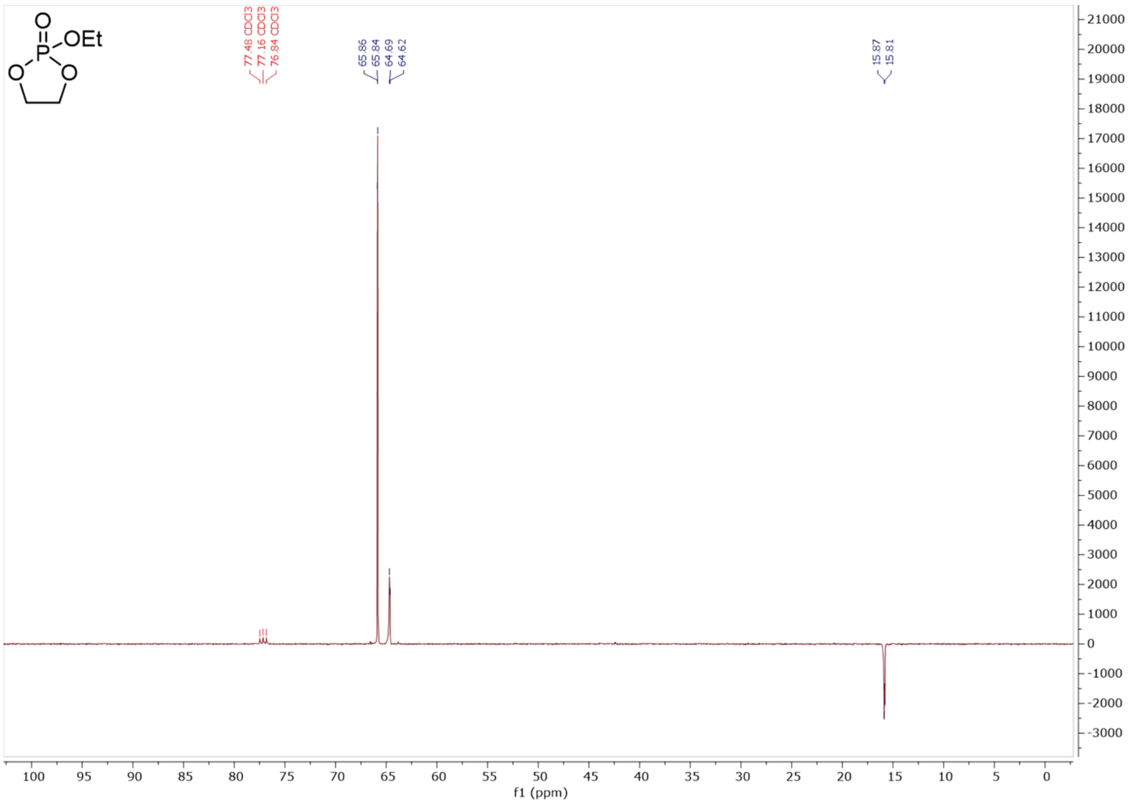

Figure S69. ^{13}C APT NMR spectrum (100.6 MHz) of 2-ethoxy-1,3,2-dioxaphospholane 2-oxide in $CDCl_3$.

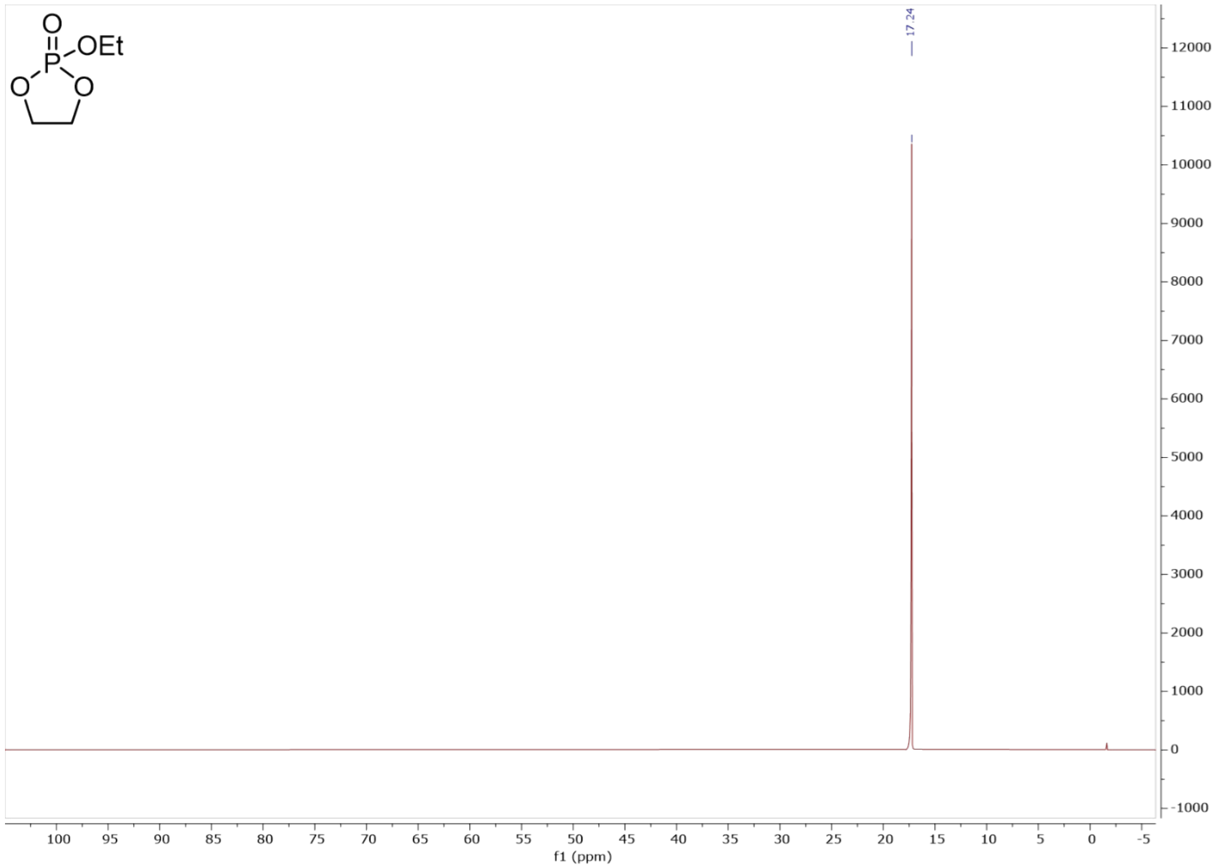

Figure S70. ^{31}P NMR spectrum (162 MHz) of 2-ethoxy-1,3,2-dioxaphospholane 2-oxide in $CDCl_3$.

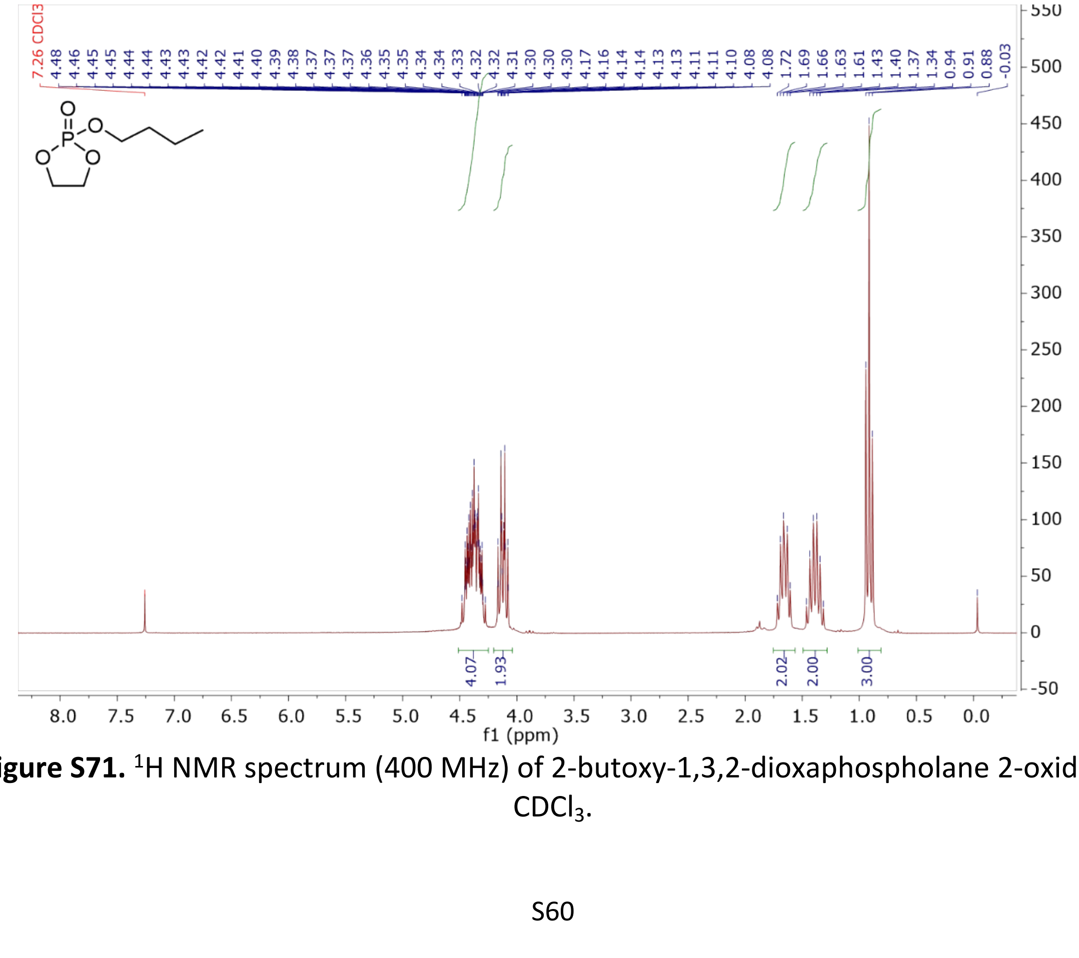

Figure S71. ^{1}H NMR spectrum (400 MHz) of 2-butoxy-1,3,2-dioxaphospholane 2-oxide in $CDCl_3$.

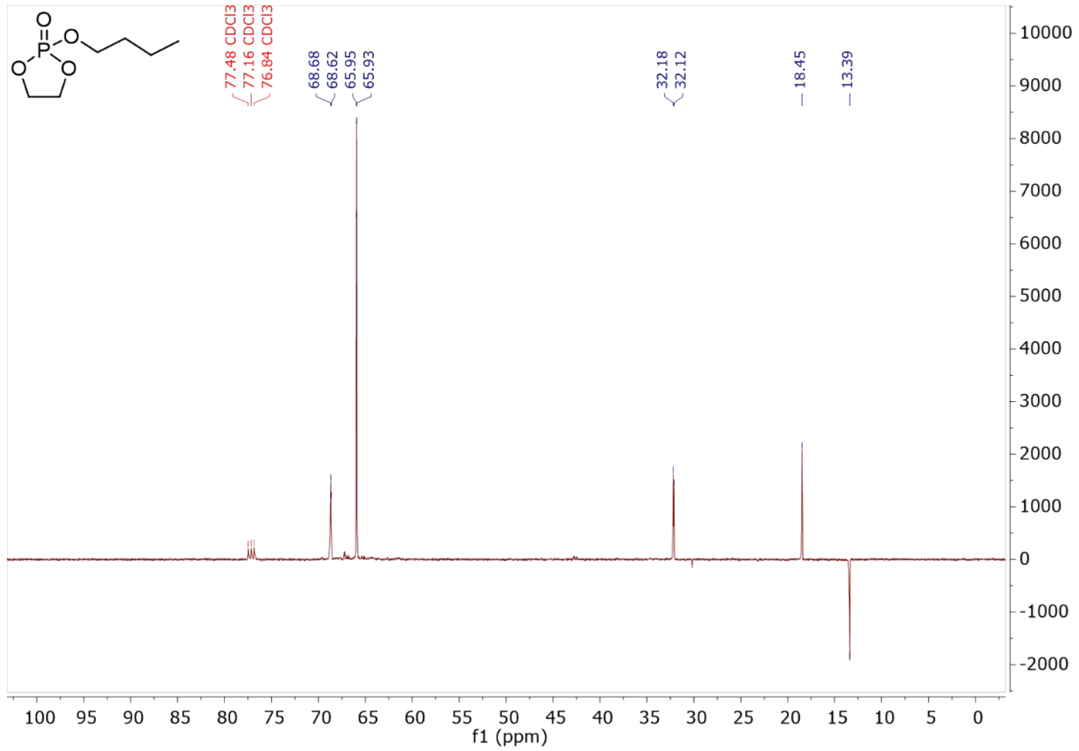

Figure S72. ^{13}C APT NMR spectrum (100.6 MHz) of 2-butoxy-1,3,2-dioxaphospholane 2-oxide in $CDCl_3$.

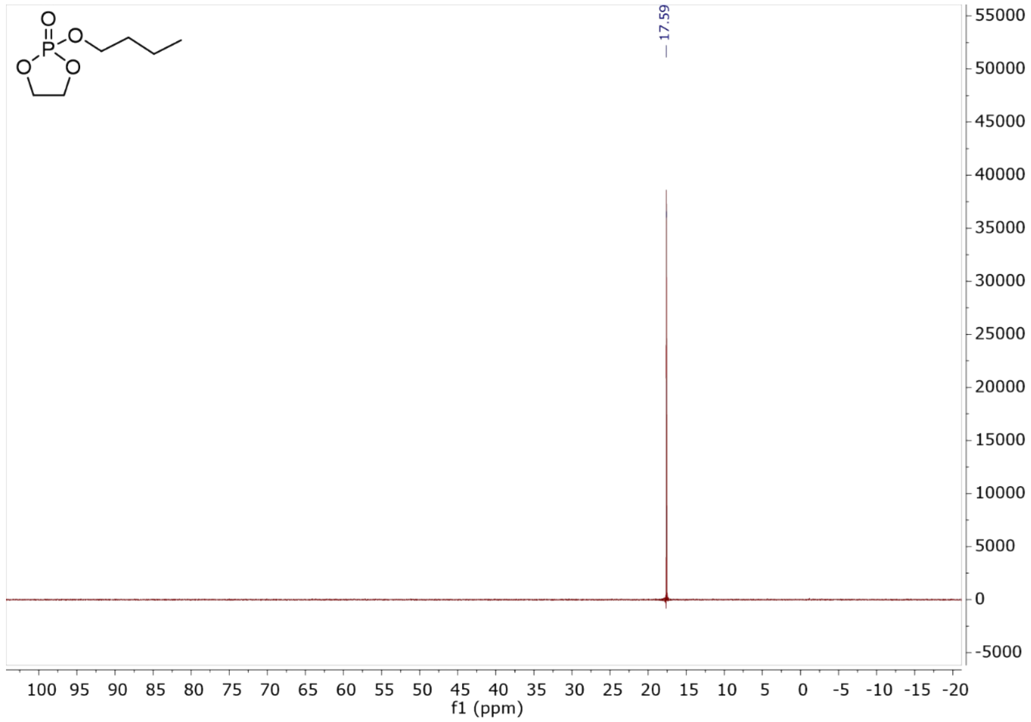

Figure S73. ^{31}P NMR spectrum (162 MHz) of 2-butoxy-1,3,2-dioxaphospholane 2-oxide in $CDCl_3$.

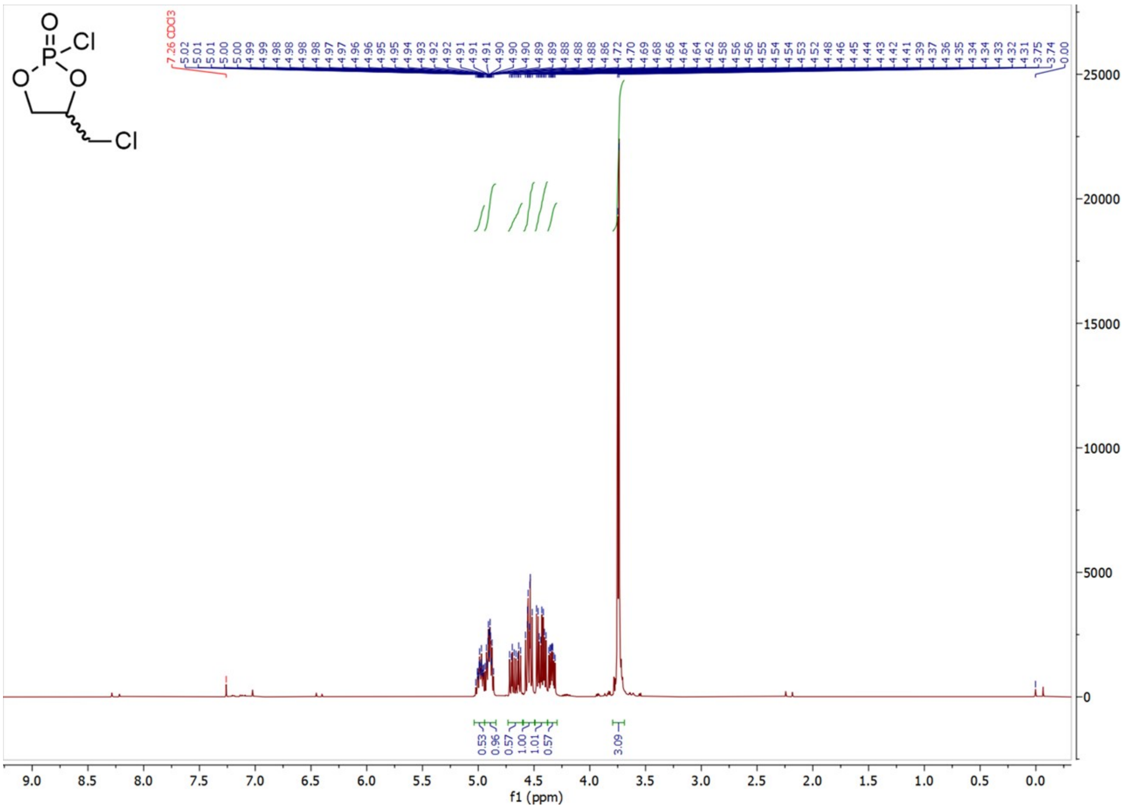

Figure S74. ^{1}H NMR spectrum (400 MHz) of 2-chloro-4-(chloromethyl)-1,3,2-dioxaphospholane 2-oxide in $CDCl_3$ (mixture of stereoisomers).

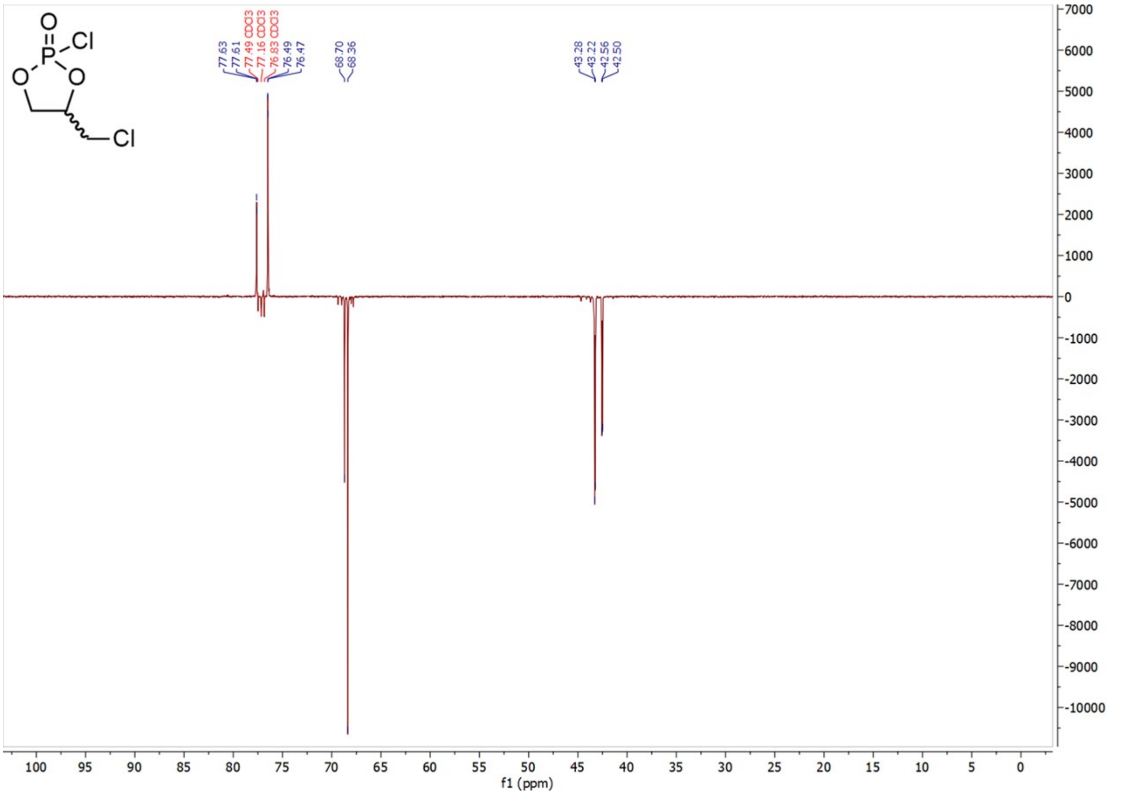

Figure S75. ^{13}C APT NMR spectrum (100.6 MHz) of 2-chloro-4-(chloromethyl)-1,3,2-dioxaphospholane 2-oxide in $CDCl_3$ (mixture of stereoisomers).

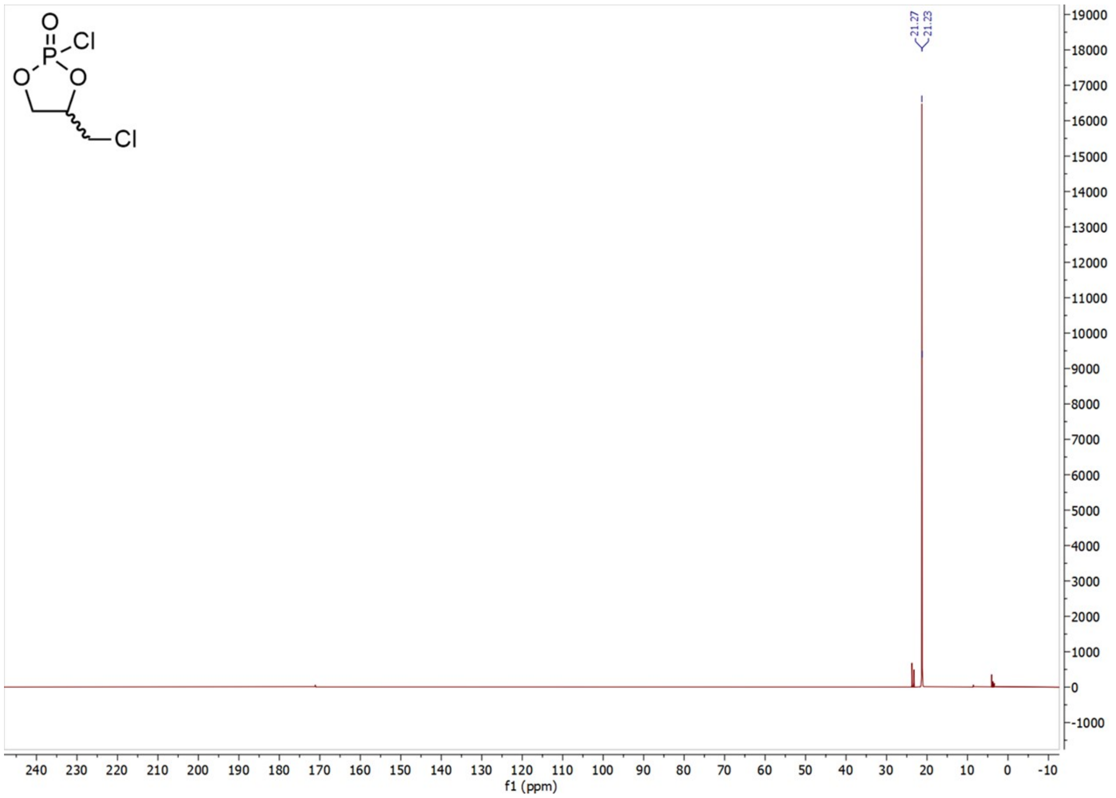

Figure S76. ^{31}P NMR spectrum (162 MHz) of 2-chloro-4-(chloromethyl)-1,3,2-dioxaphospholane 2-oxide in $CDCl_3$ (mixture of stereoisomers).

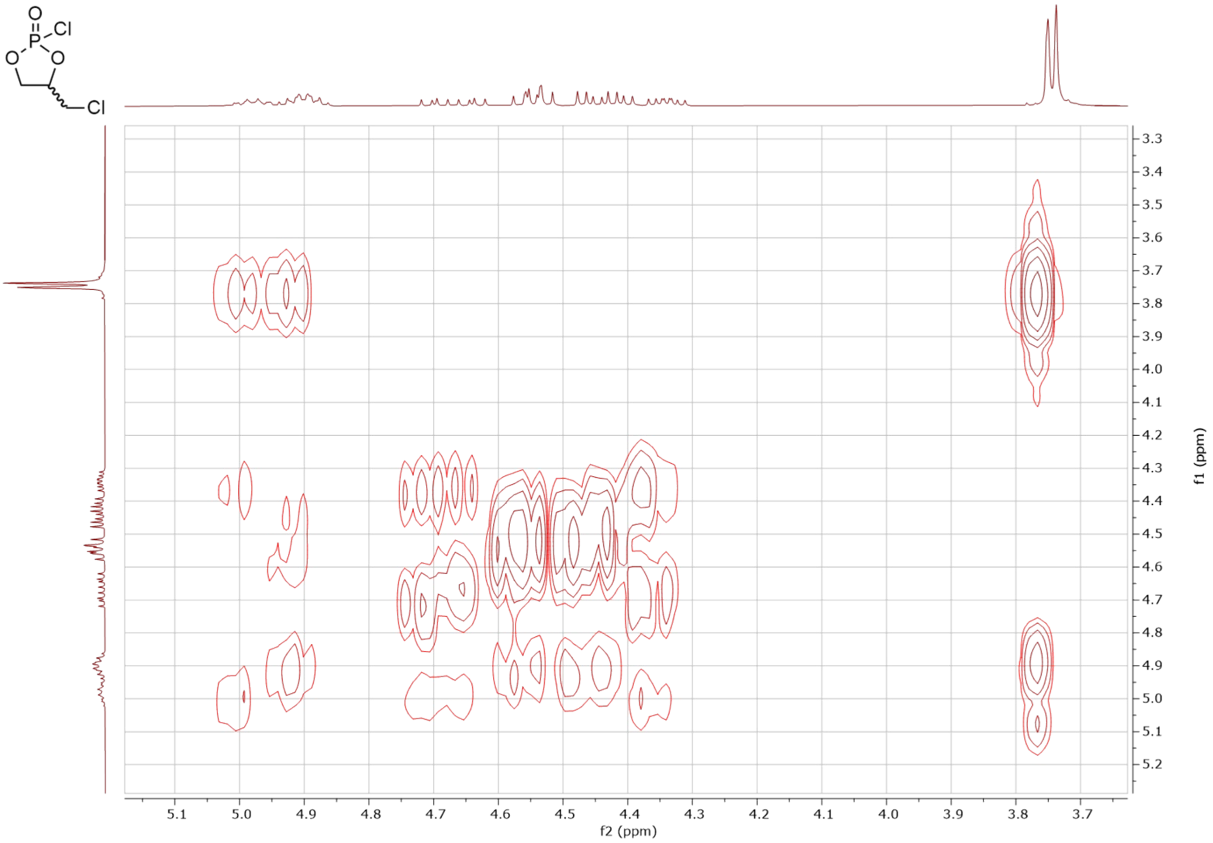

Figure S77. COSY NMR spectrum of 2-chloro-4-(chloromethyl)-1,3,2-dioxaphospholane 2-oxide in $CDCl_3$ (mixture of stereoisomers).

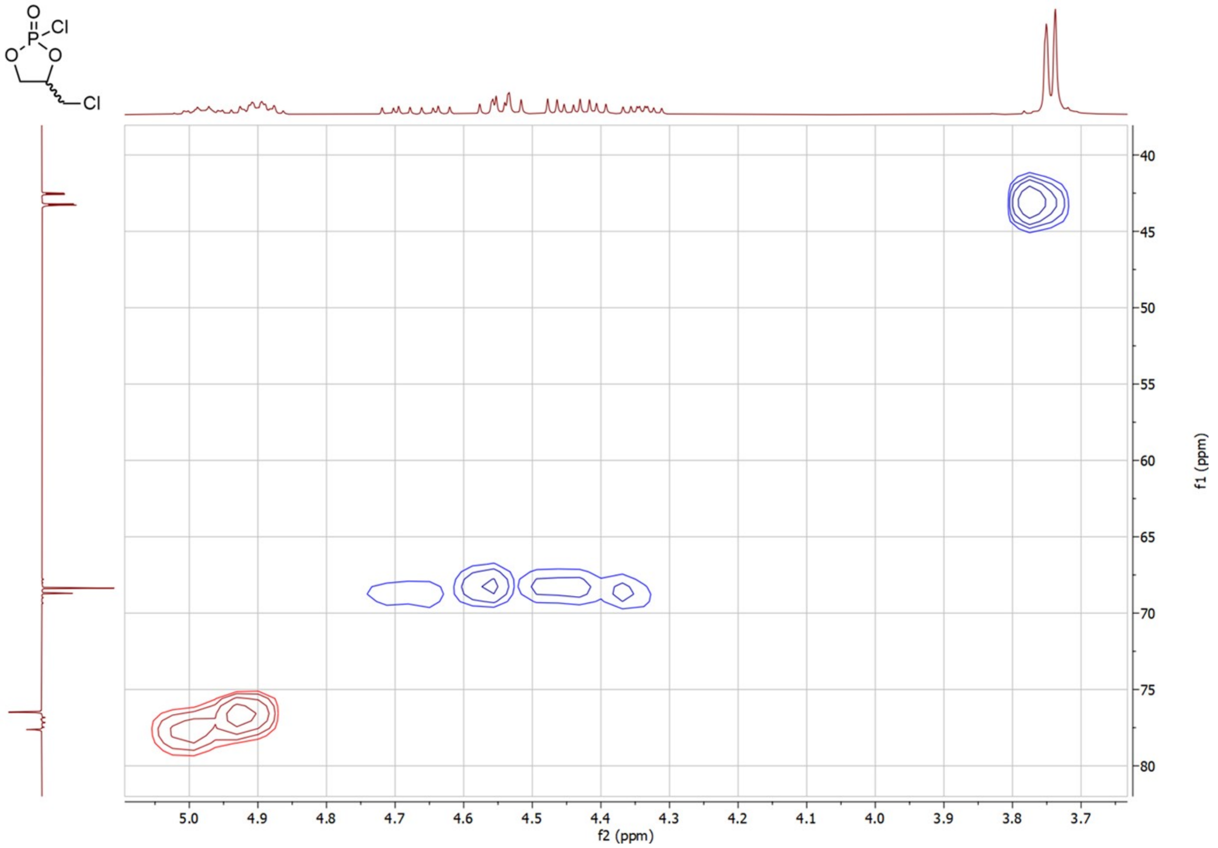

Figure S78. HSQC NMR spectrum of 2-chloro-4-(chloromethyl)-1,3,2-dioxaphospholane 2-oxide in $CDCl_3$ (mixture of stereoisomers).

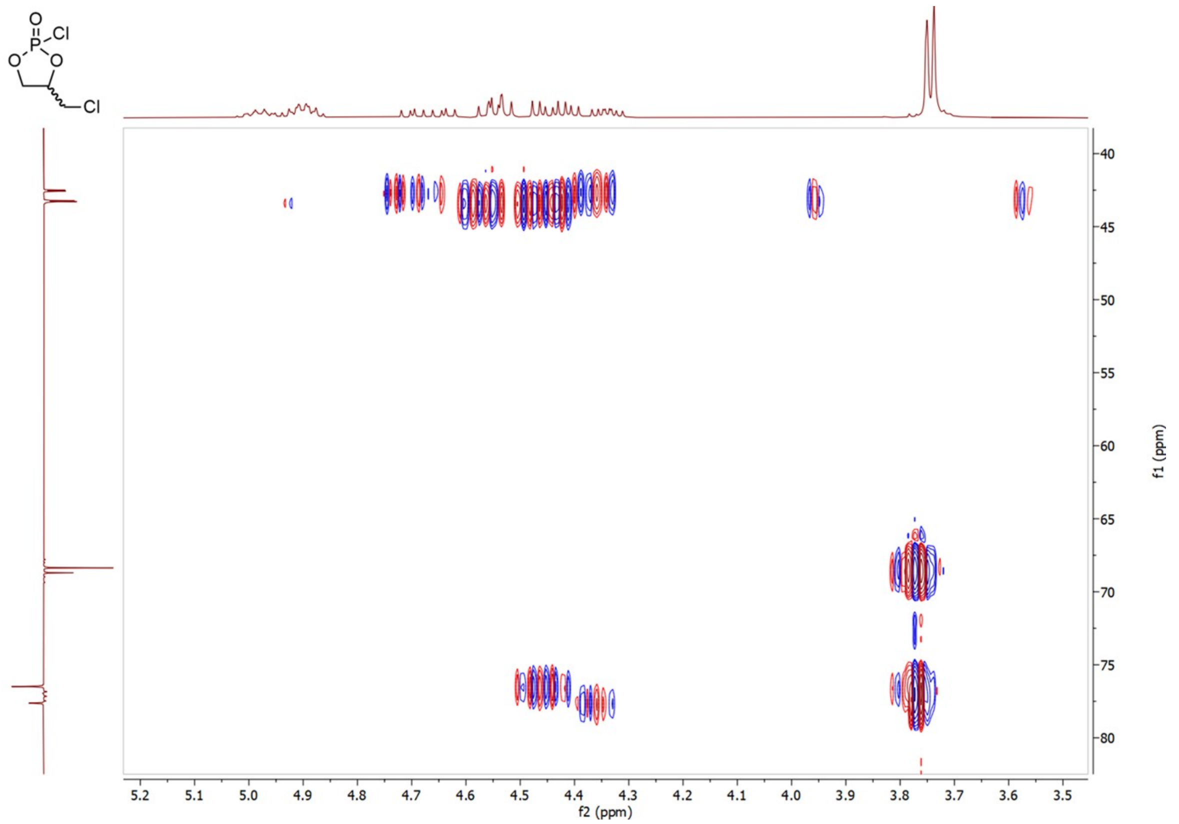

Figure S79. HMBC NMR spectrum of 2-chloro-4-(chloromethyl)-1,3,2-dioxaphospholane 2-oxide in $CDCl_3$ (mixture of stereoisomers).

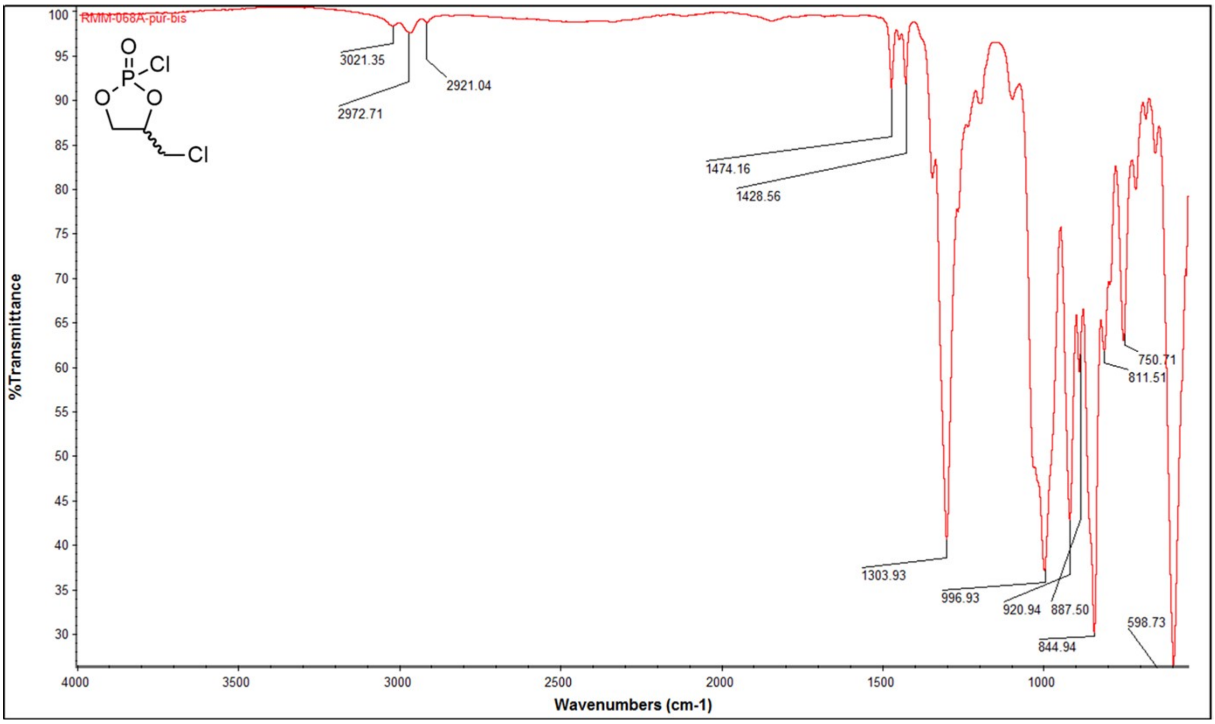

Figure S80. Infrared (ATR) spectrum of 2-chloro-4-(chloromethyl)-1,3,2-dioxaphospholane 2-oxide (mixture of stereoisomers).

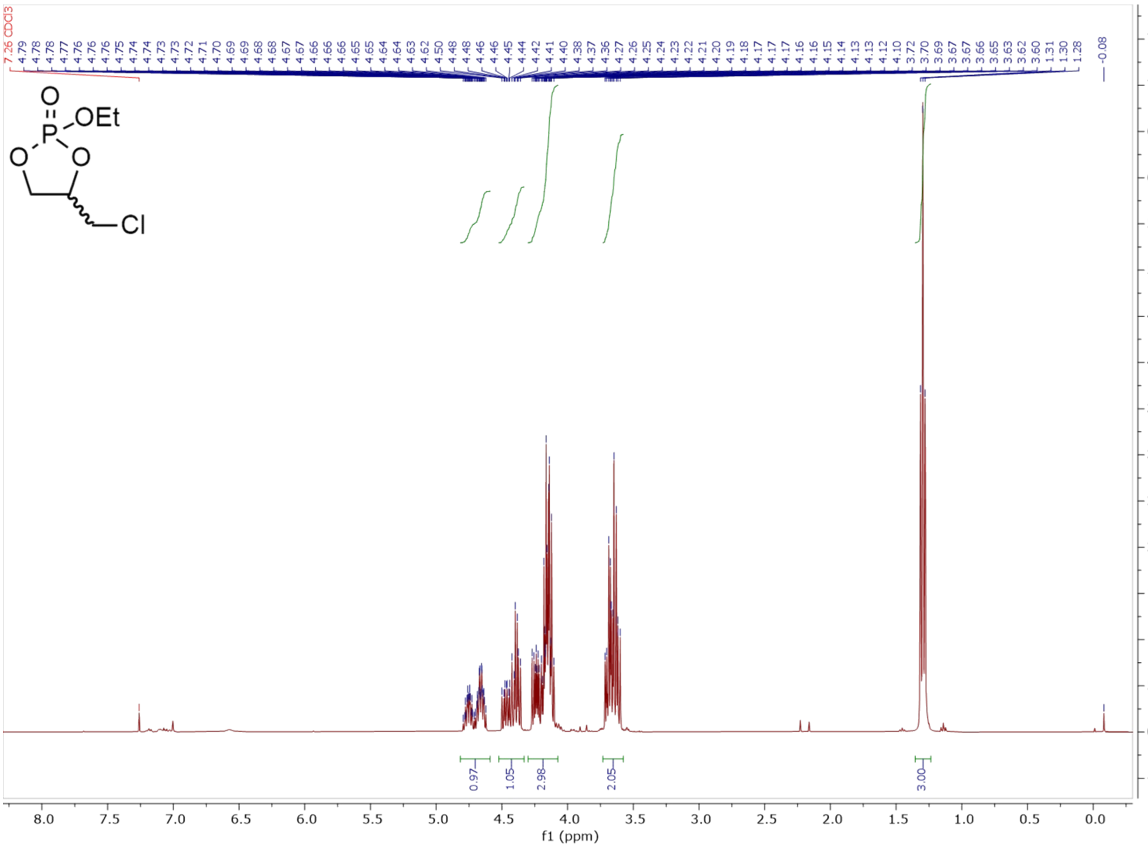

Figure S81. ^{1}H NMR spectrum (400 MHz) of 4-(chloromethyl)-2-ethoxy-1,3,2-dioxaphospholane 2-oxide in $CDCl_3$ (mixture of stereoisomers).

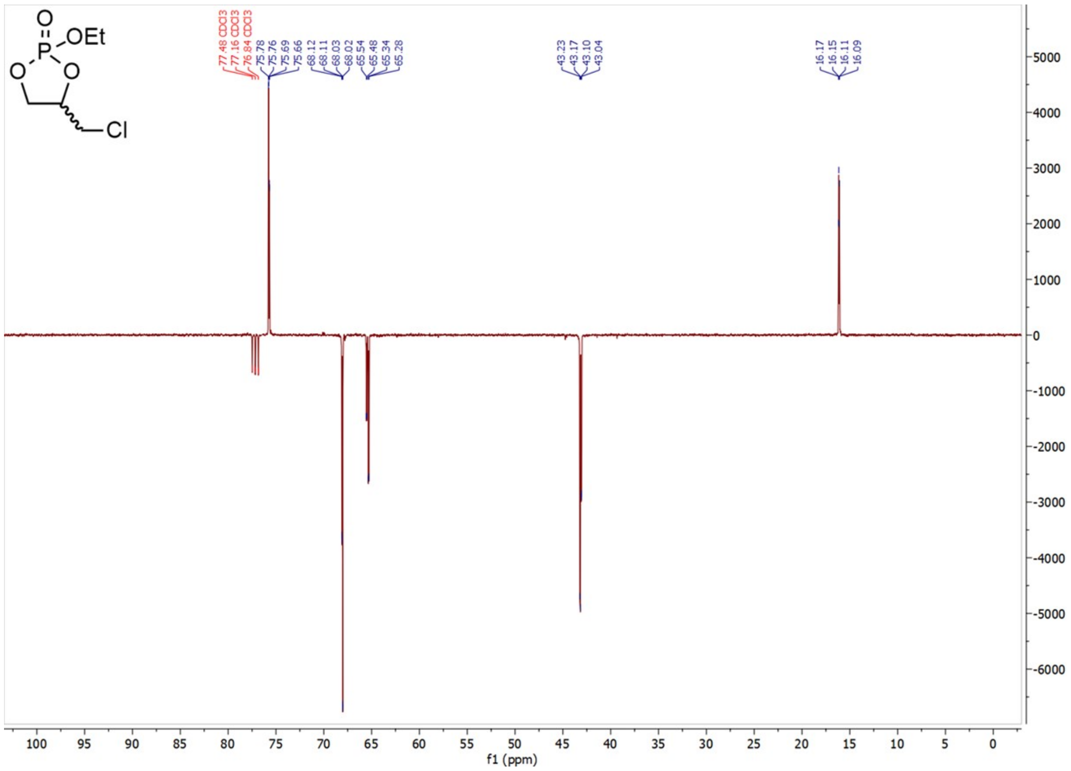

Figure S82. ^{13}C APT NMR spectrum (100.6 MHz) of 4-(chloromethyl)-2-ethoxy-1,3,2-dioxaphospholane 2-oxide in $CDCl_3$ (mixture of stereoisomers).

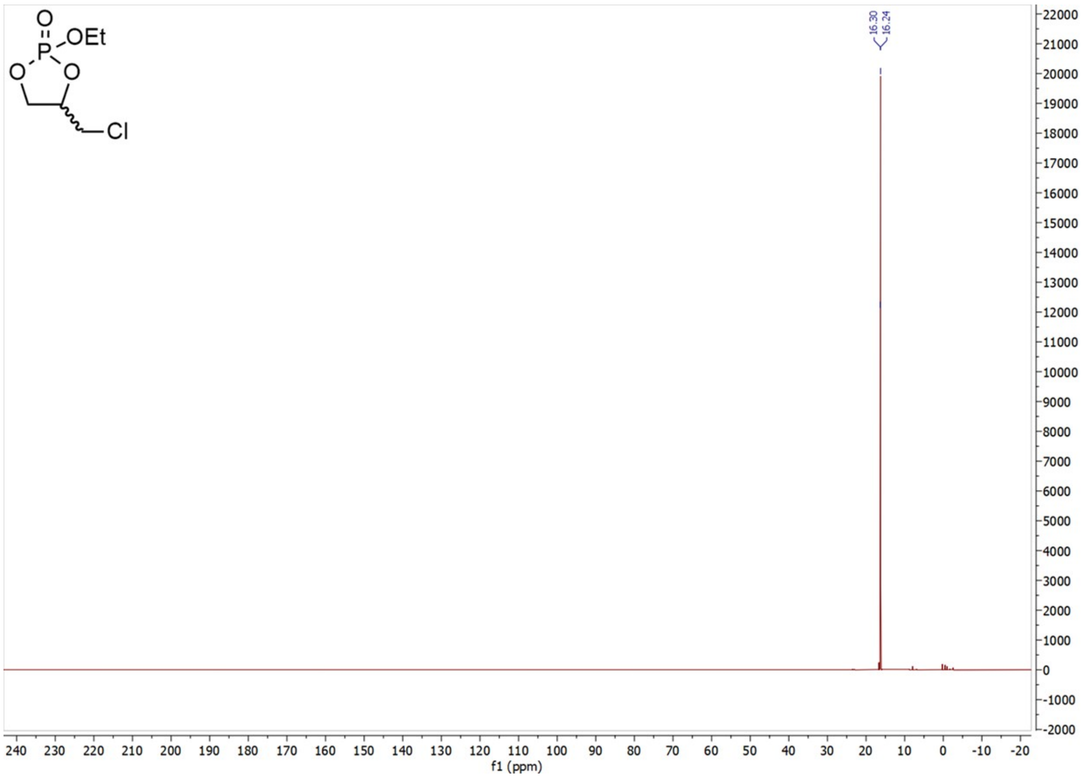

Figure S83. ^{31}P NMR spectrum (162 MHz) of 4-(chloromethyl)-2-ethoxy-1,3,2-dioxaphospholane 2-oxide in $CDCl_3$ (mixture of stereoisomers).

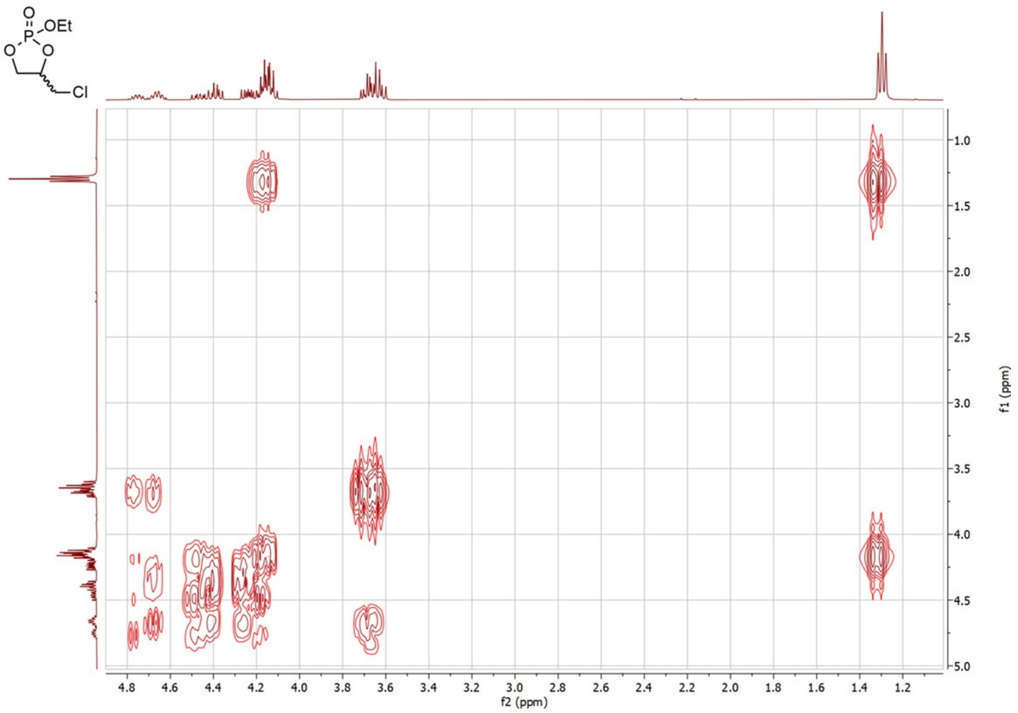

Figure S84. COSY NMR spectrum of 4-(chloromethyl)-2-ethoxy-1,3,2-dioxaphospholane 2-oxide in $CDCl_3$ (mixture of stereoisomers).

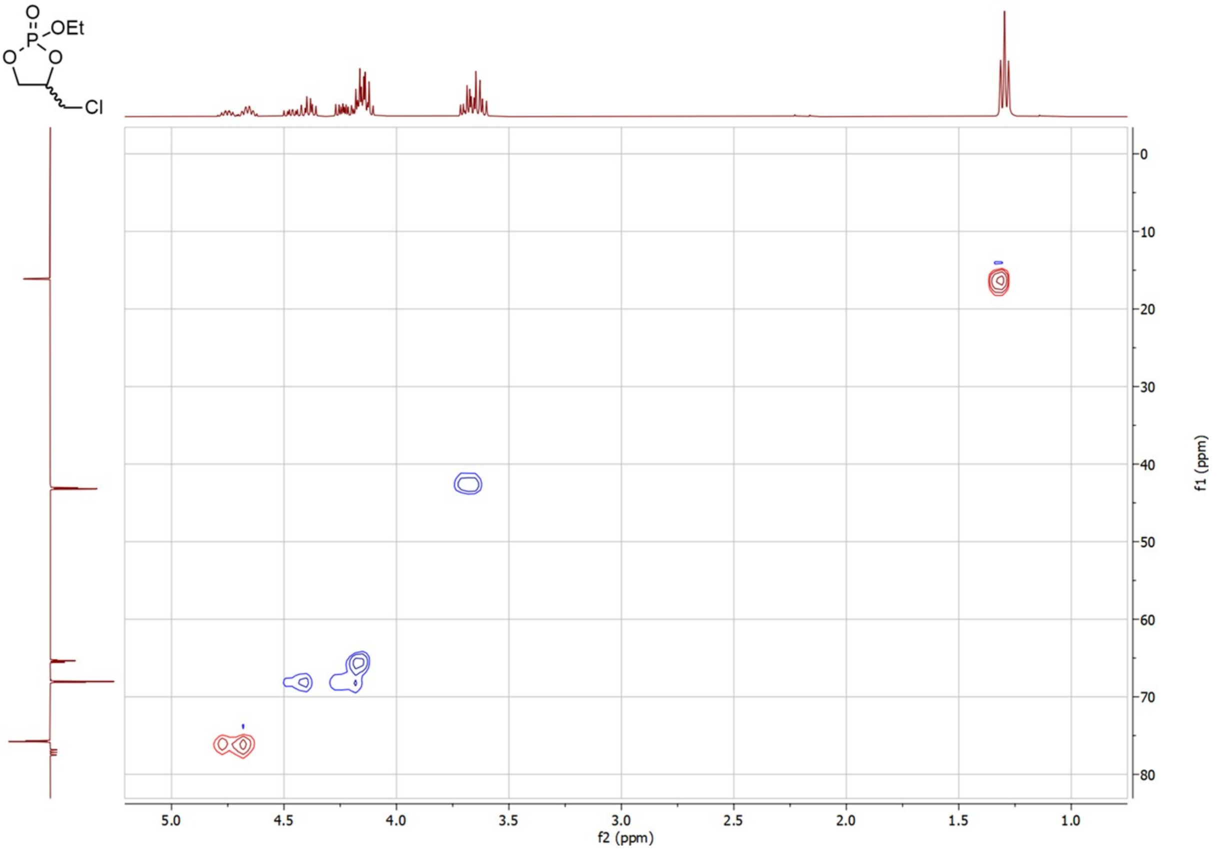

Figure S85. HSQC NMR spectrum of 4-(chloromethyl)-2-ethoxy-1,3,2-dioxaphospholane 2-oxide in $CDCl_3$ (mixture of stereoisomers).

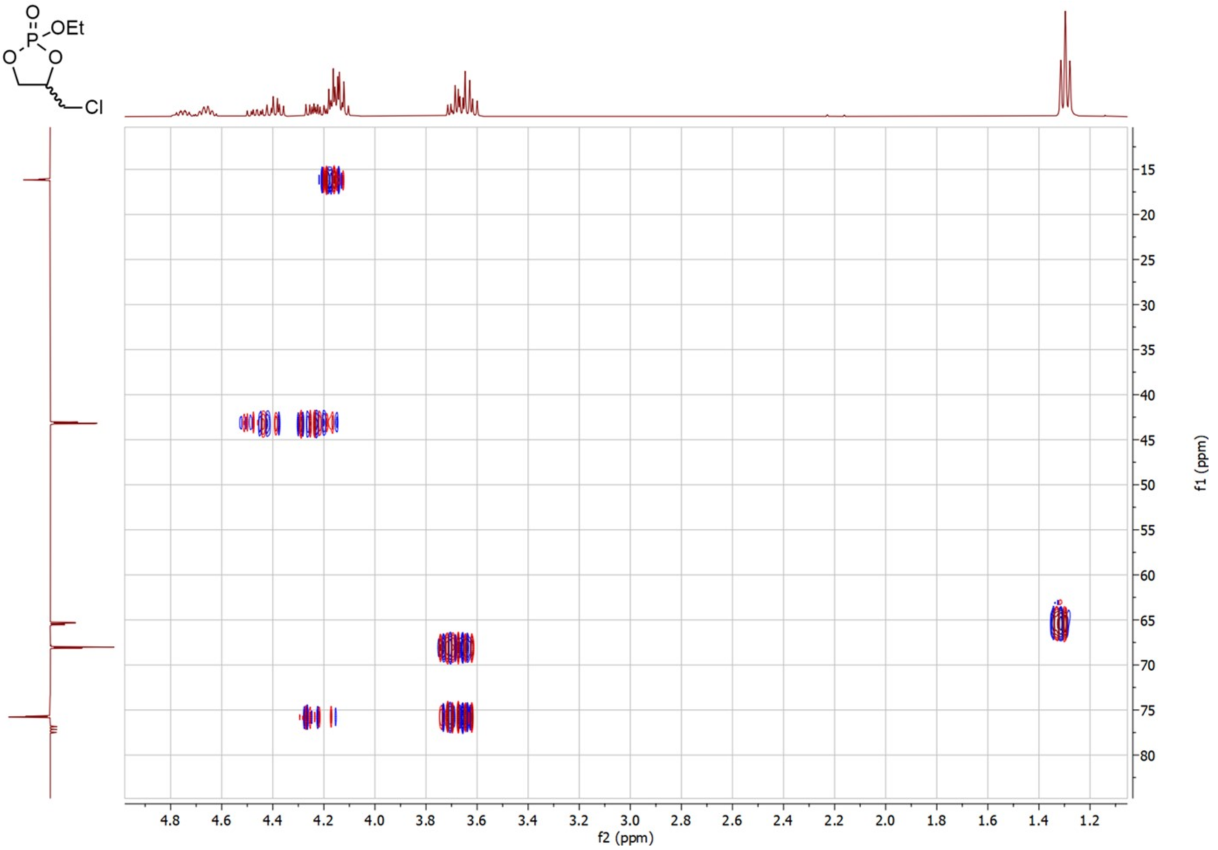

Figure S86. HMBC NMR spectrum of 4-(chloromethyl)-2-ethoxy-1,3,2-dioxaphospholane 2-oxide in $CDCl_3$ (mixture of stereoisomers).

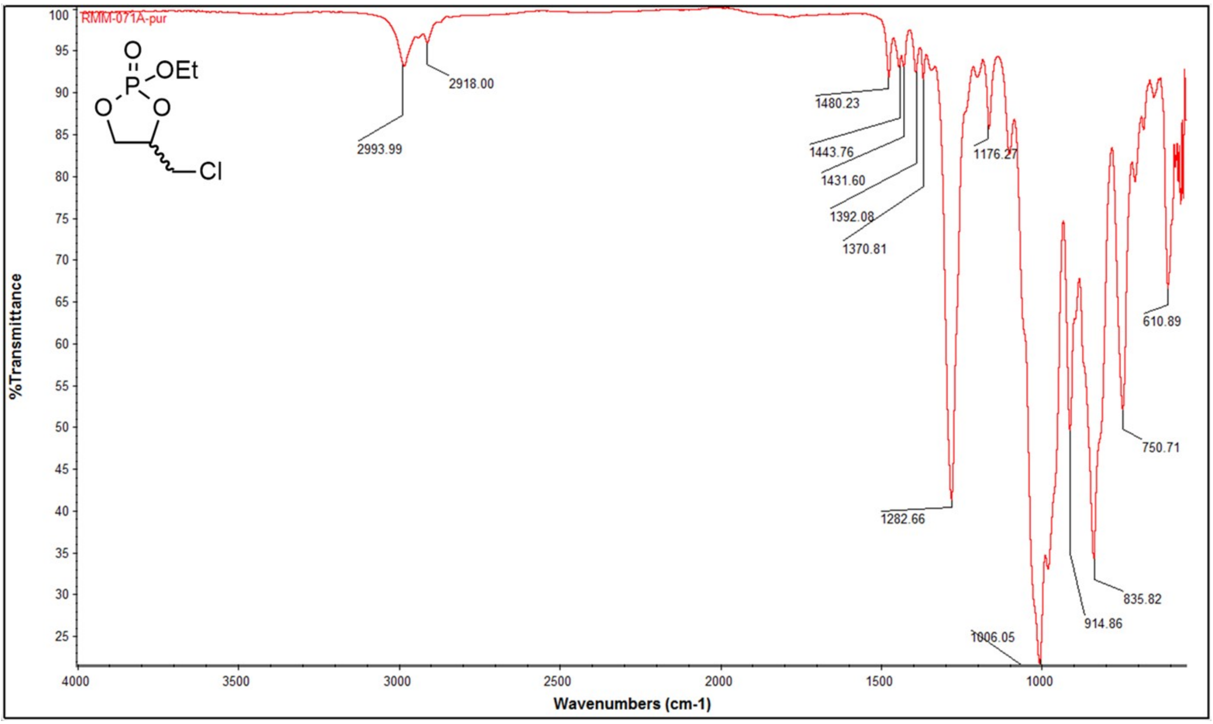

Figure S87. Infrared (ATR) spectrum of 4-(chloromethyl)-2-ethoxy-1,3,2-dioxaphospholane 2-oxide (mixture of stereoisomers).

2.8 **Characterization of polymeric materials**

2.8.1 **TEG-PEEP** (batch process)

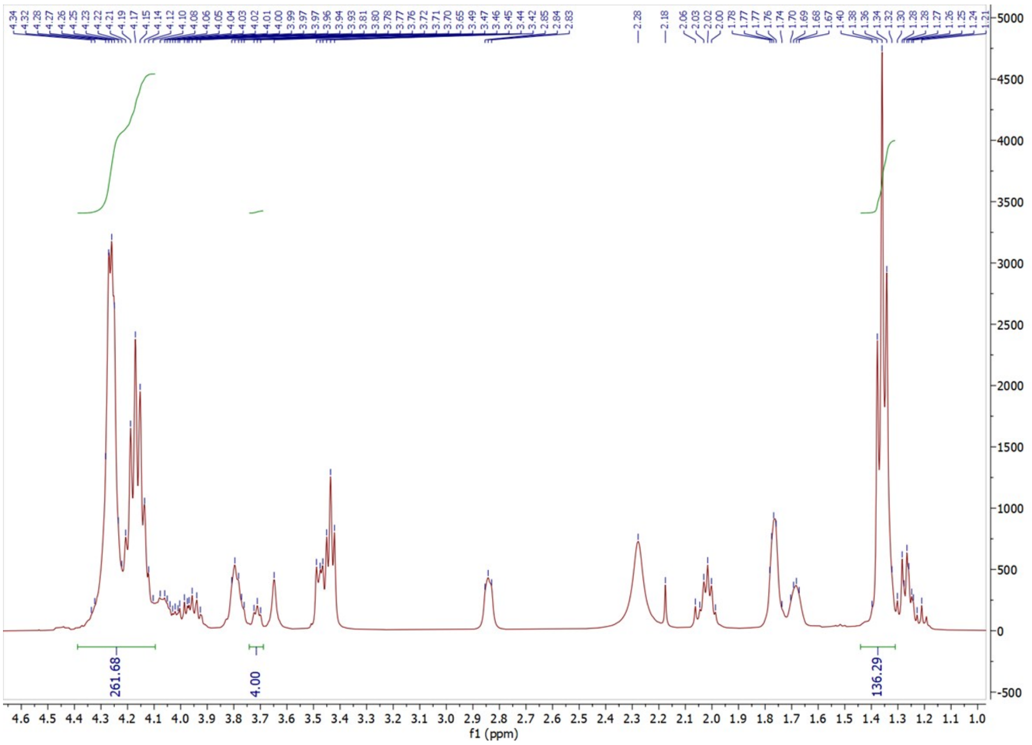

Figure S88. ^{1}H NMR spectrum (400 MHz) of **TEG-PEEP** (obtained by a batch procedure).

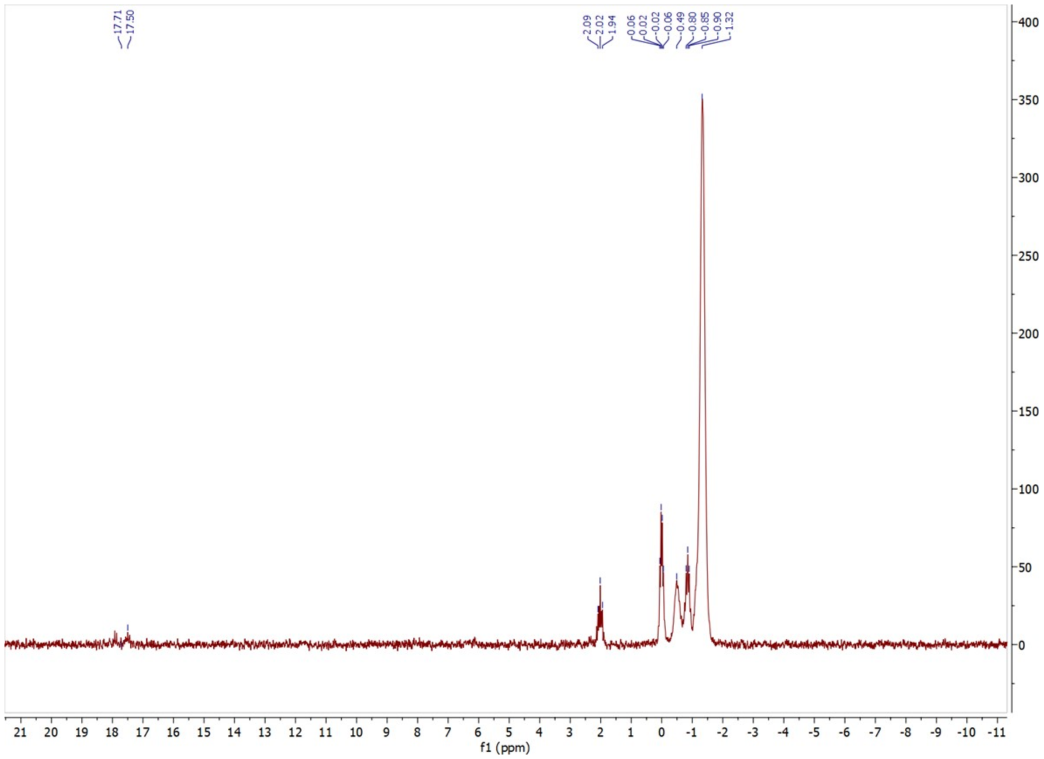

Figure S89. ^{31}P NMR spectrum (162 MHz) of **TEG-PEEP** (obtained by a batch procedure).

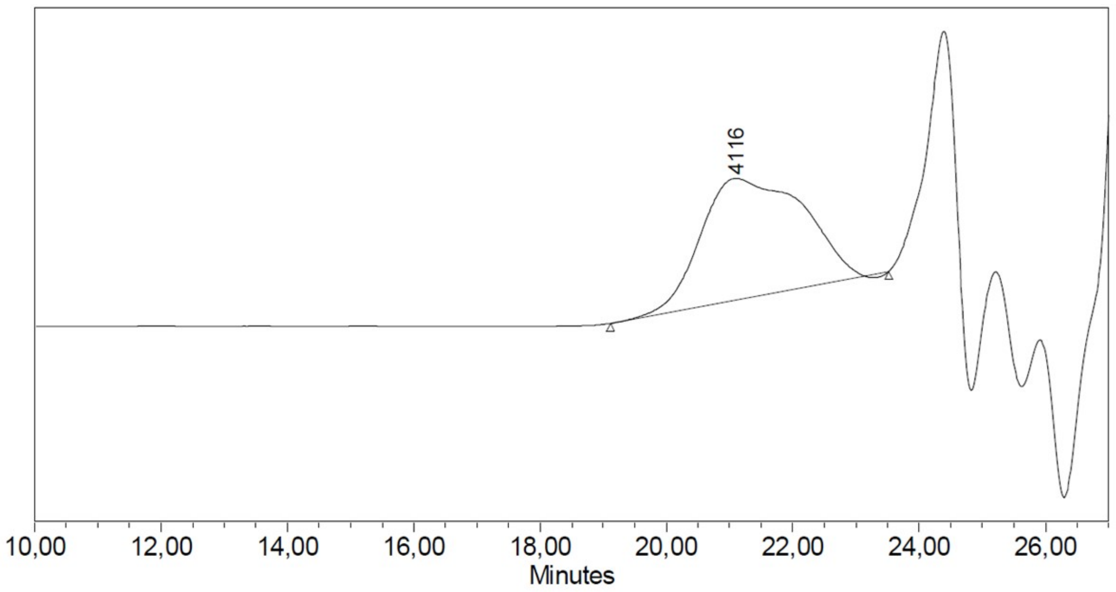

Figure S90. SEC chromatogram of **TEG-PEEP** (obtained by a batch procedure).

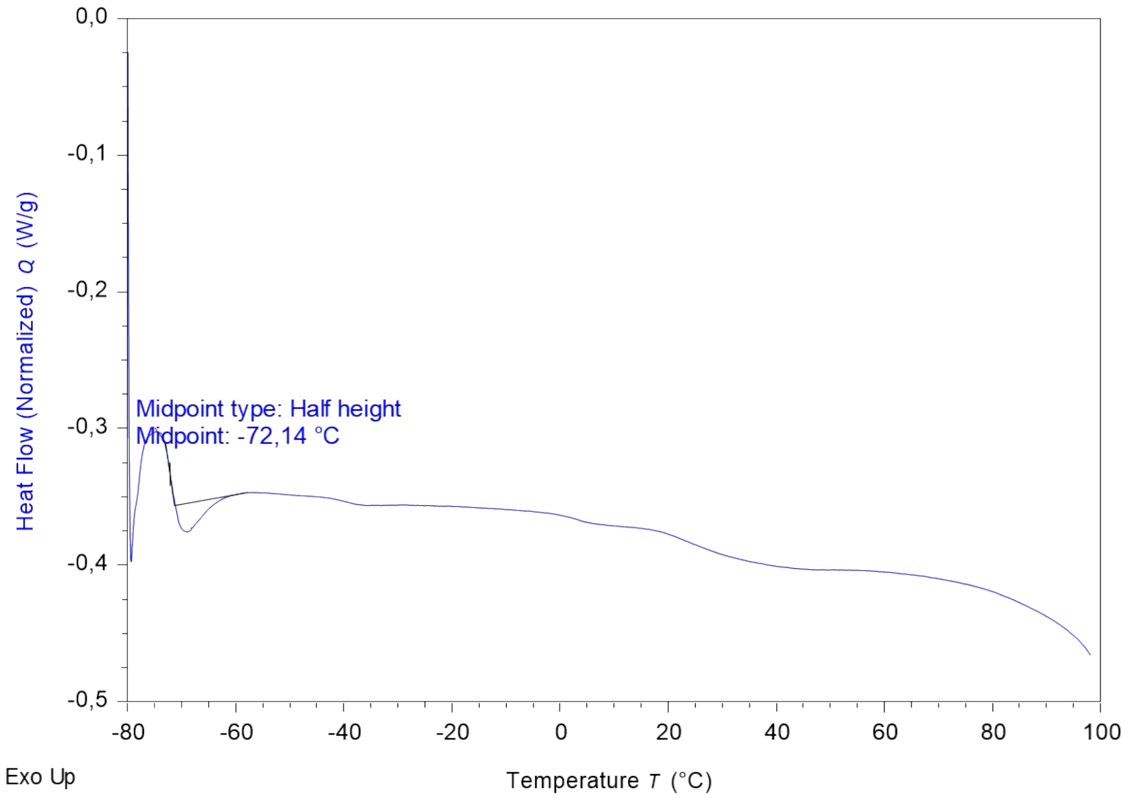

Figure S91. DSC thermogram of **TEG-PEEP** (obtained by a batch procedure).

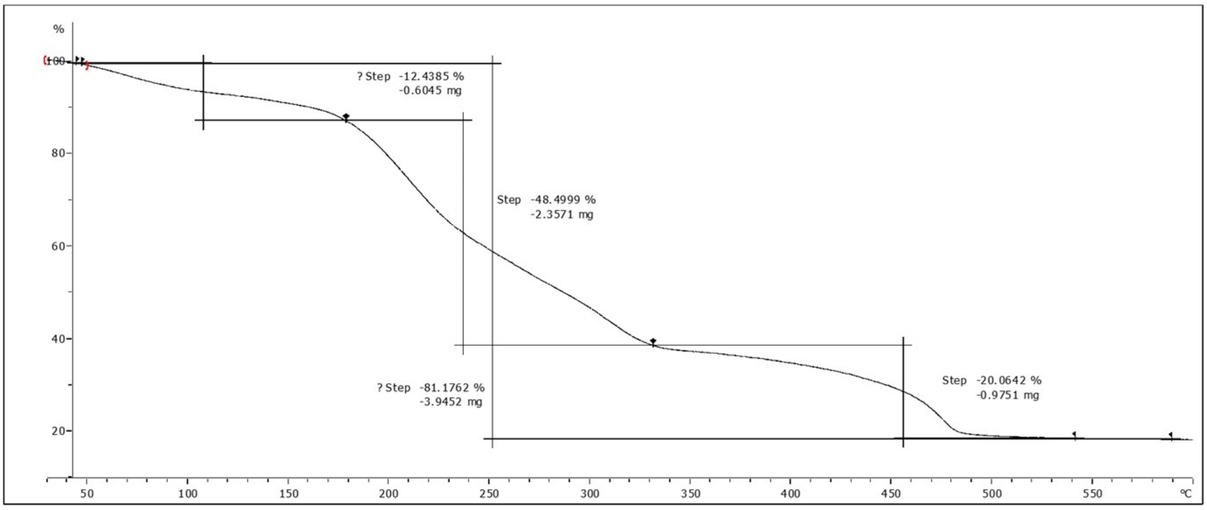

Figure S92. TGA thermogram of **TEG-PEEP** (obtained by a batch procedure).

2.8.2 **TEG-PEEP** (flow process, 10 min of residence time)

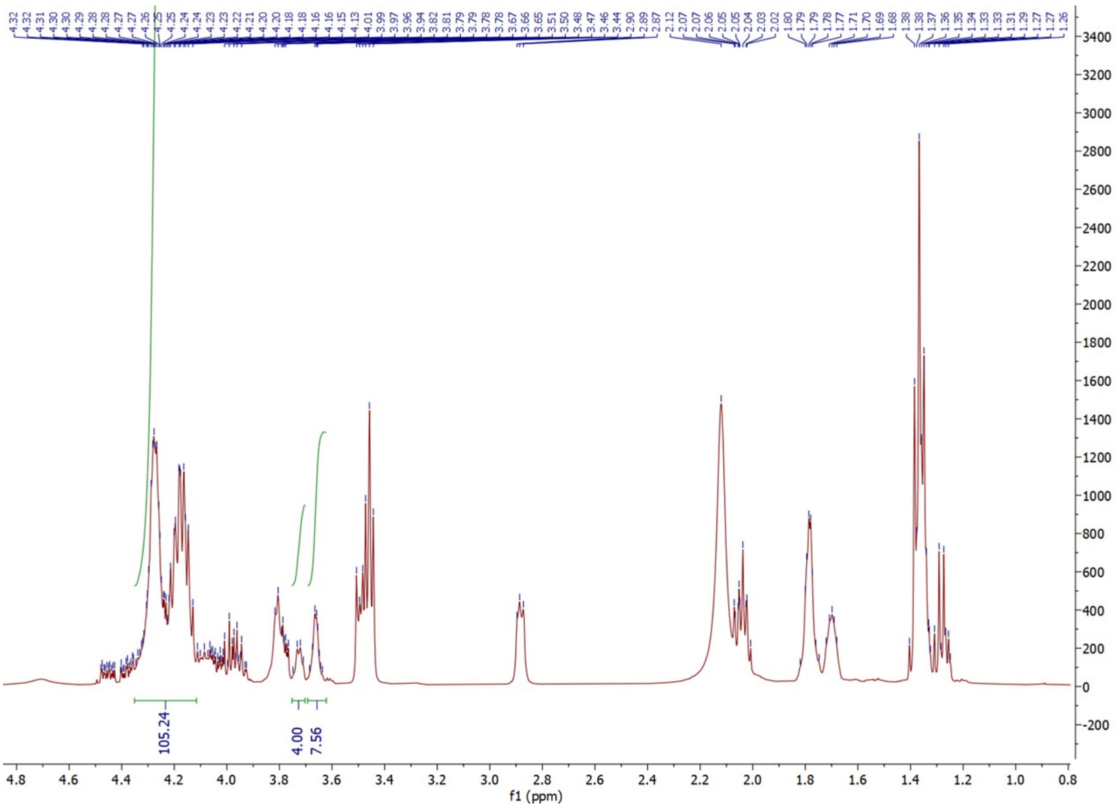

Figure S93. ^{1}H NMR spectrum (400 MHz) of **TEG-PEEP** (obtained by a flow procedure, 10 min of residence time).

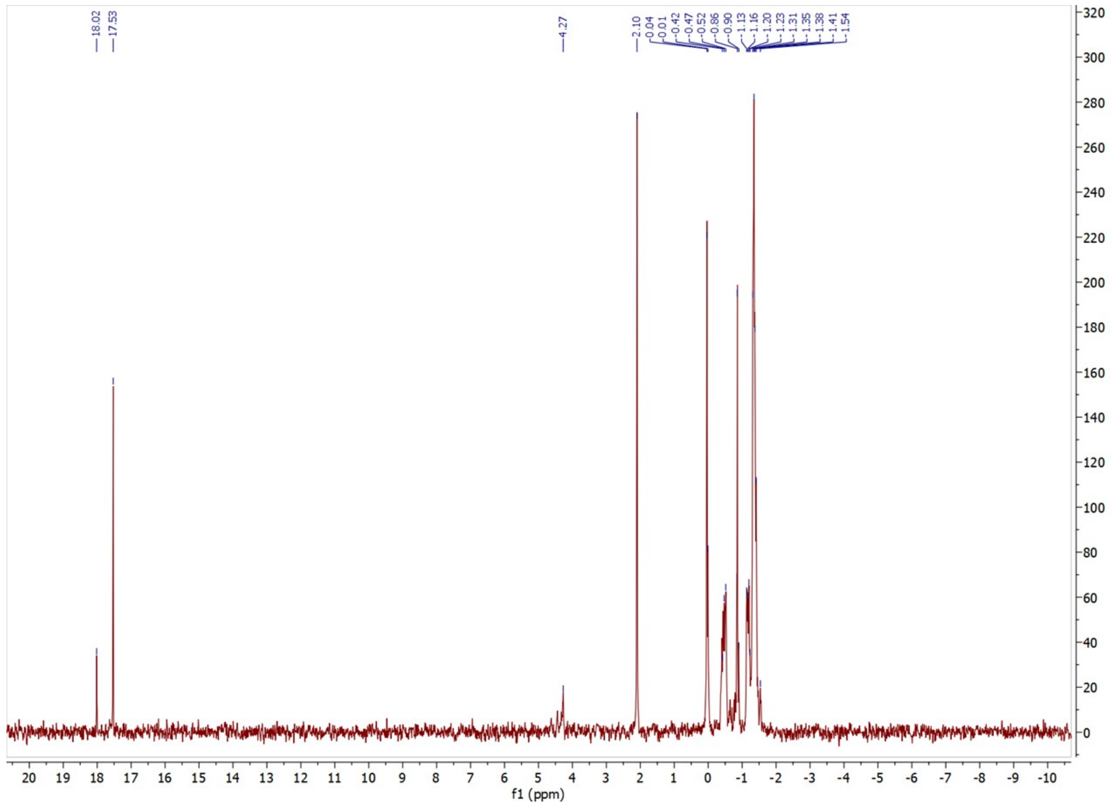

Figure S94. ^{31}P NMR spectrum (162 MHz) of **TEG-PEEP** (obtained by a flow procedure, 10 min of residence time).

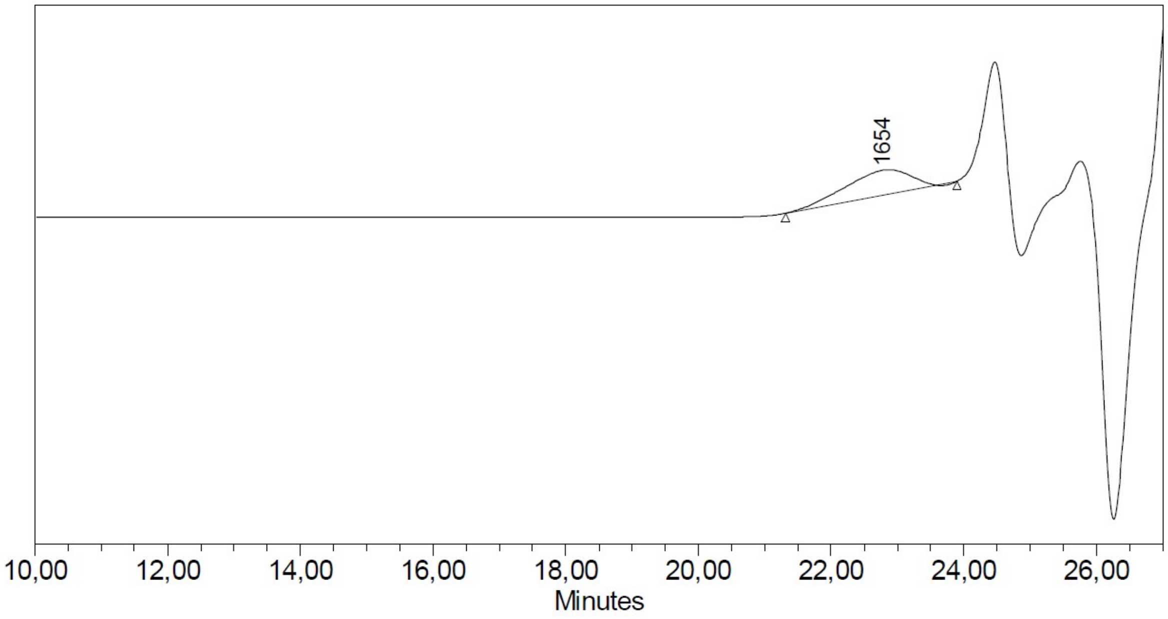

Figure S95. SEC chromatogram of **TEG-PEEP** (obtained by a flow procedure, 10 min of residence time).

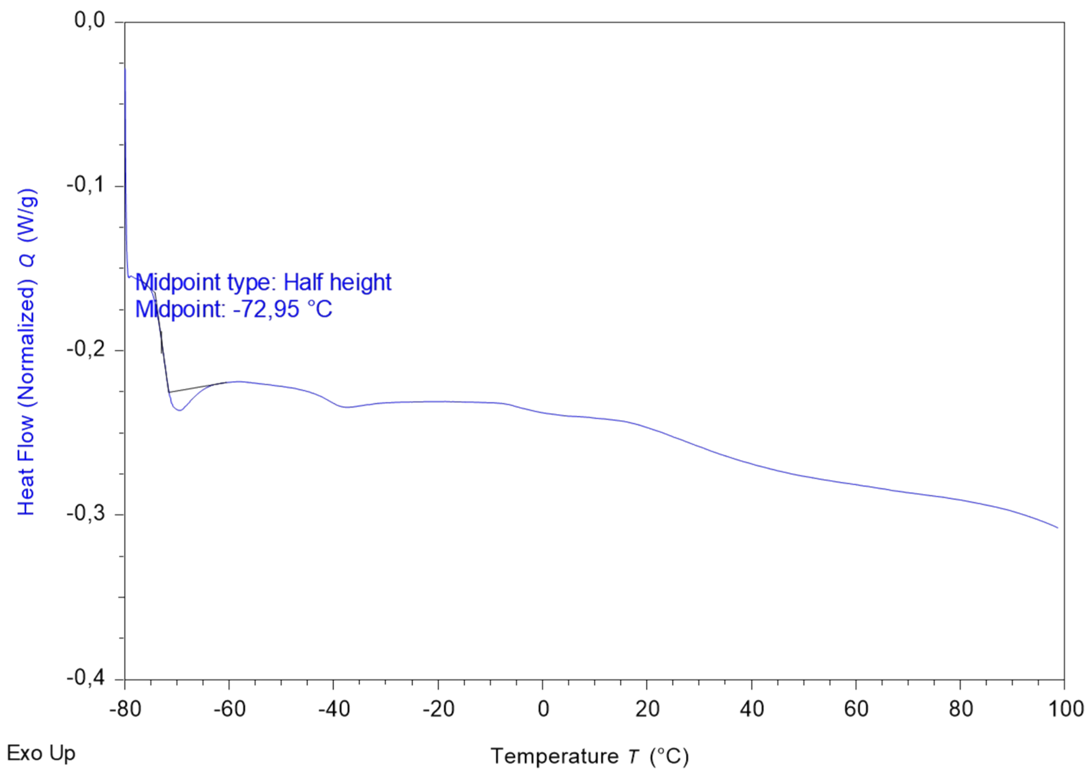

Figure S96. TGA thermogram of **TEG-PEEP** (obtained by a flow procedure, 10 min of residence time).

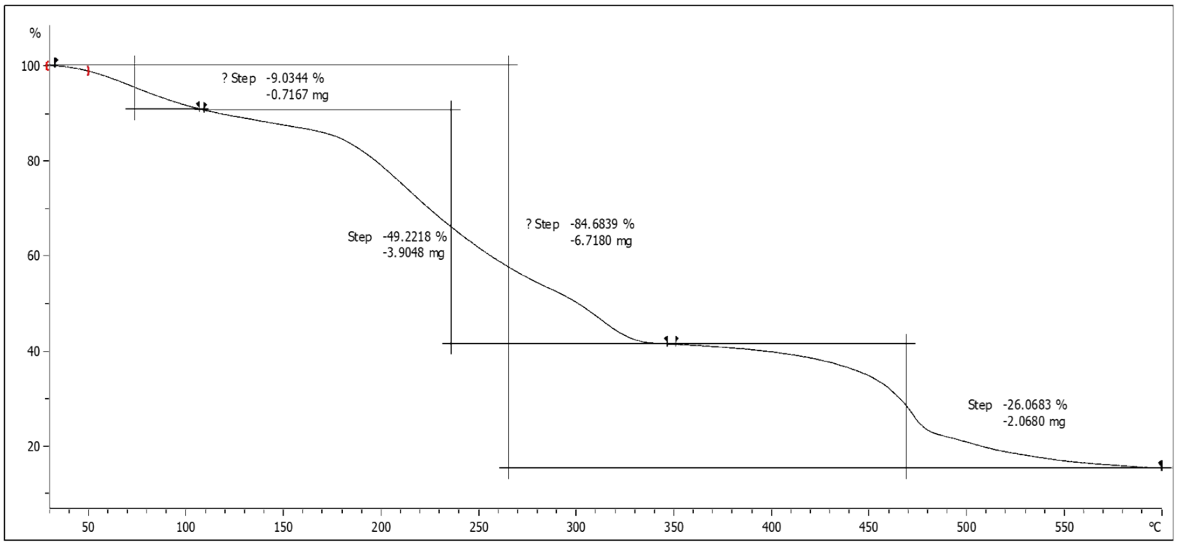

Figure S97. TGA thermogram of **TEG-PEEP** (obtained by a flow procedure, 10 min of residence time).

2.8.3 **TEG-PEEP** (flow process, 20 min of residence time)

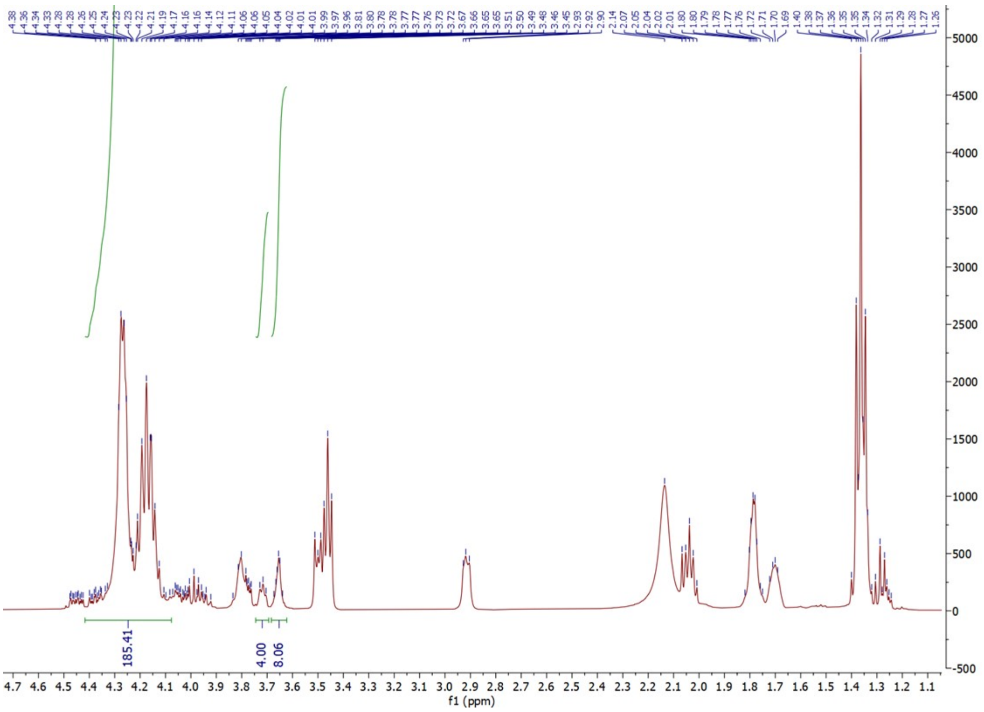

Figure S98. ^{1}H NMR spectrum (400 MHz) of **TEG-PEEP** (obtained by a flow procedure, 20 min of residence time).

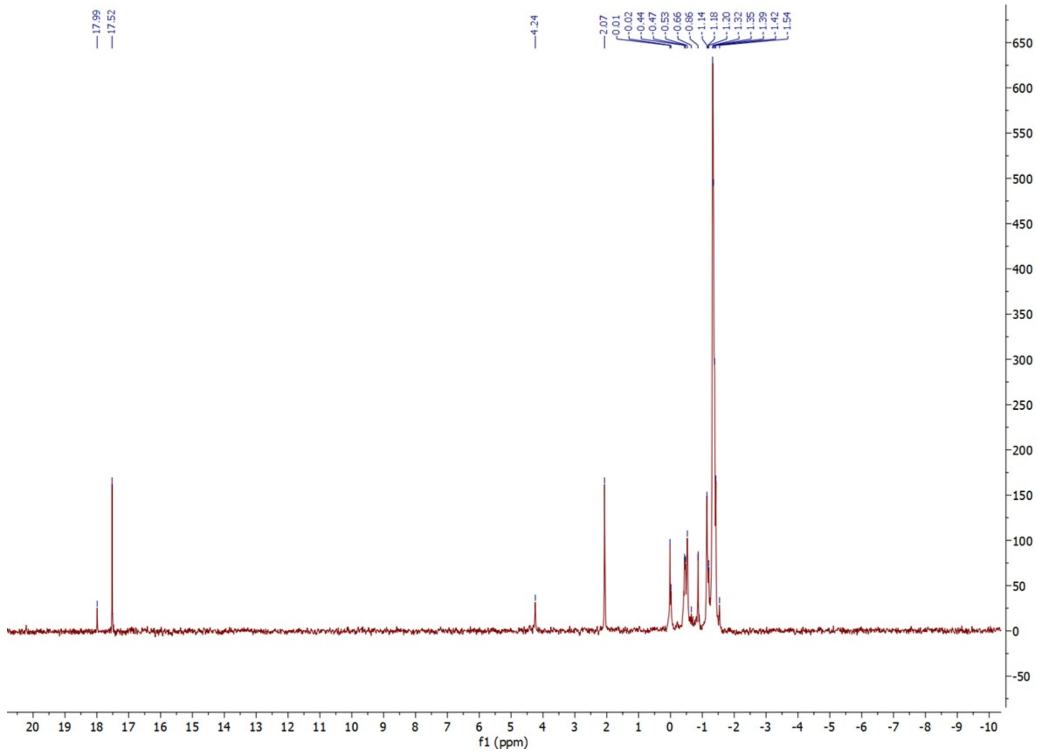

Figure S99. ^{31}P NMR spectrum (162 MHz) of **TEG-PEEP** (obtained by a flow procedure with a TEG initiator, 20 min of residence time).

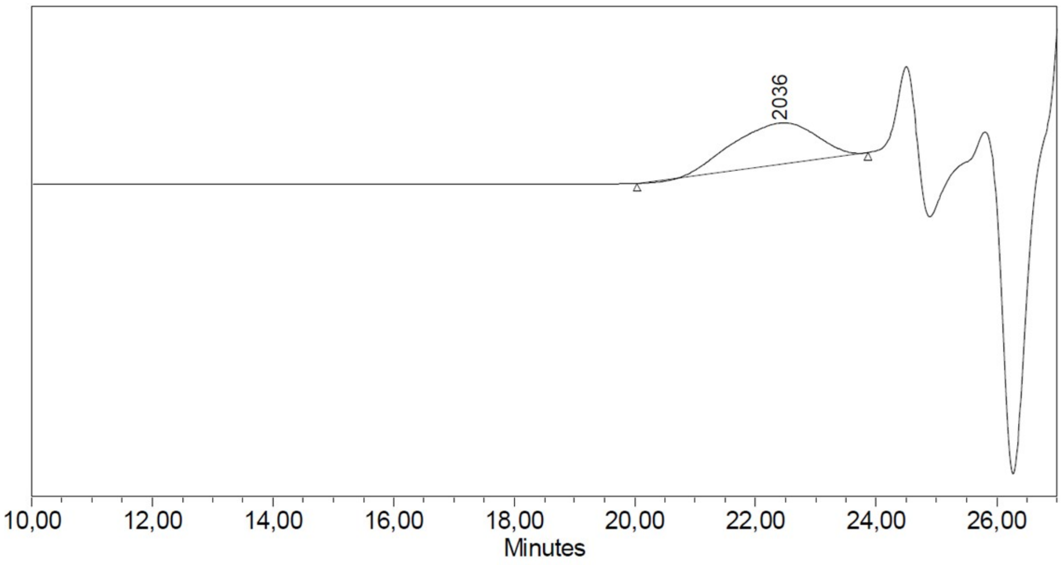

Figure S100. SEC chromatogram of **TEG-PEEP** (obtained by a flow procedure, 20 min of residence time).

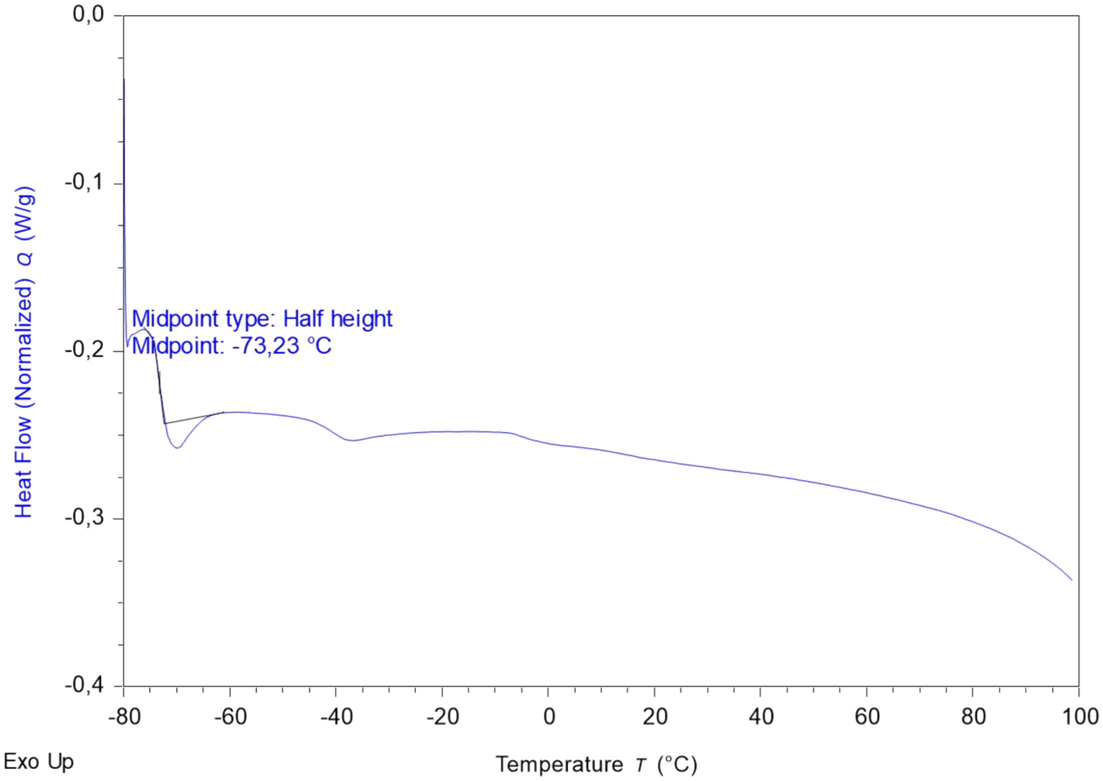

Figure S101. DSC thermogram of **TEG-PEEP** (obtained by a flow procedure, 20 min of residence time).

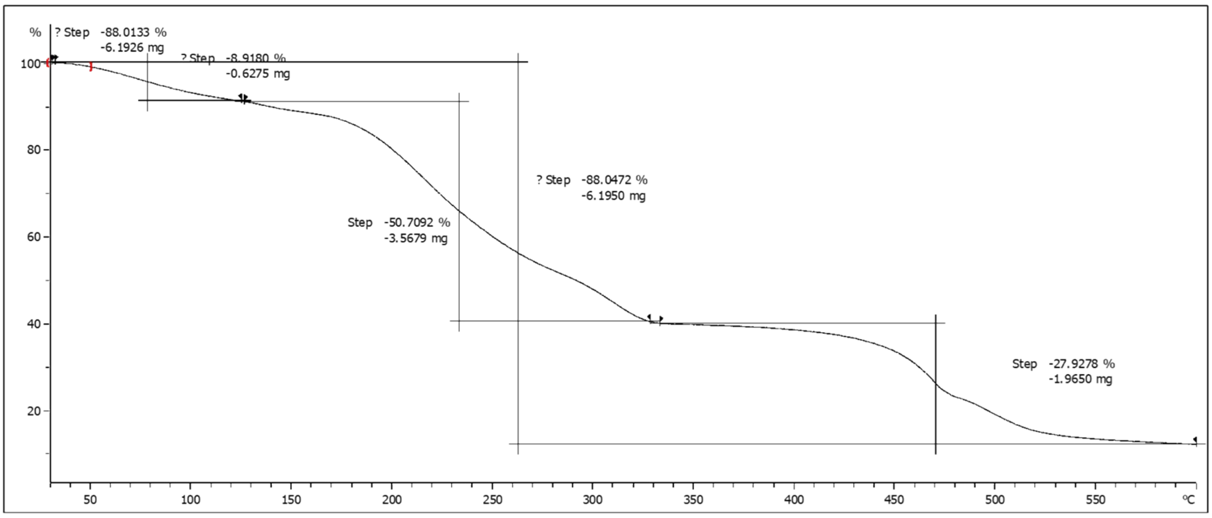

Figure S102. TGA thermogram of **TEG-PEEP** (obtained by a flow procedure, 20 min of residence time).

2.8.4 **MeO-PEO-*b*-PEEP** (flow process)

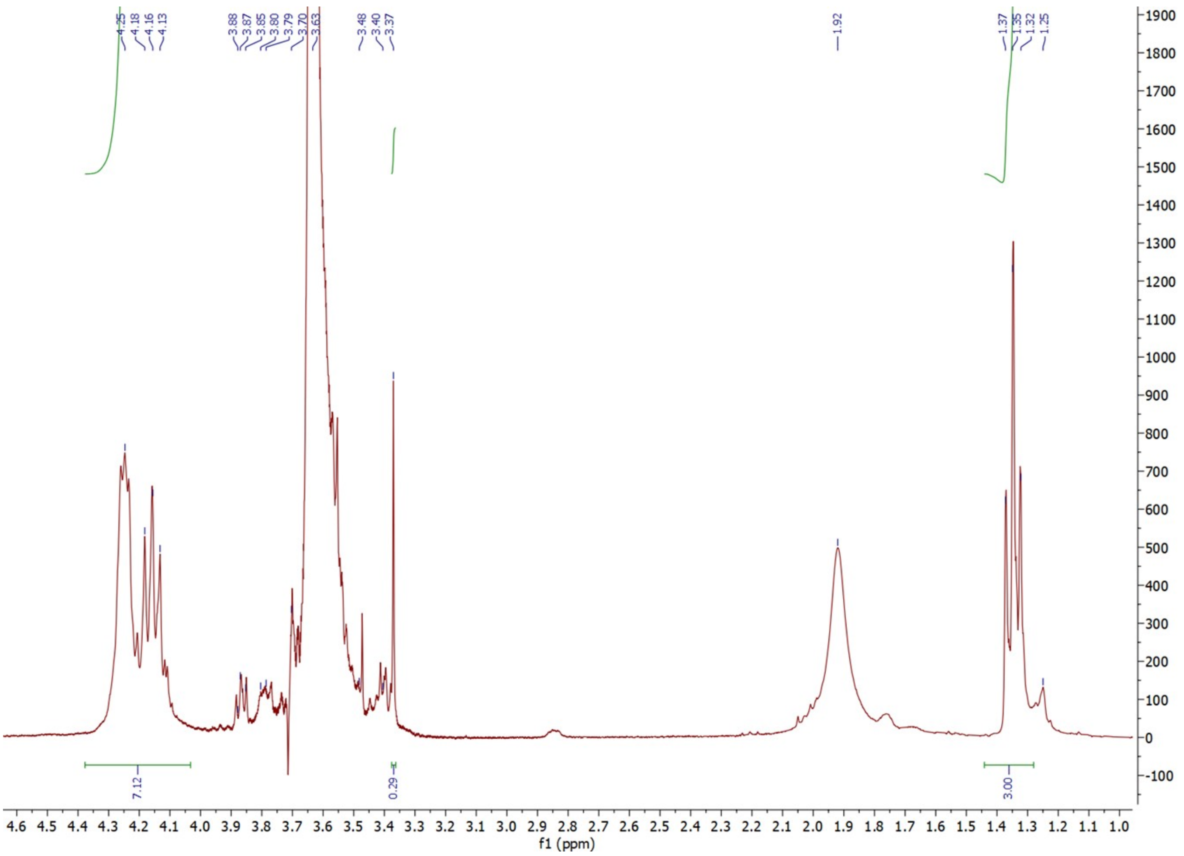

Figure S103. ^{1}H NMR spectrum (400 MHz) of **MeO-PEO-*b*-PEEP** (obtained by a flow procedure after dialysis).

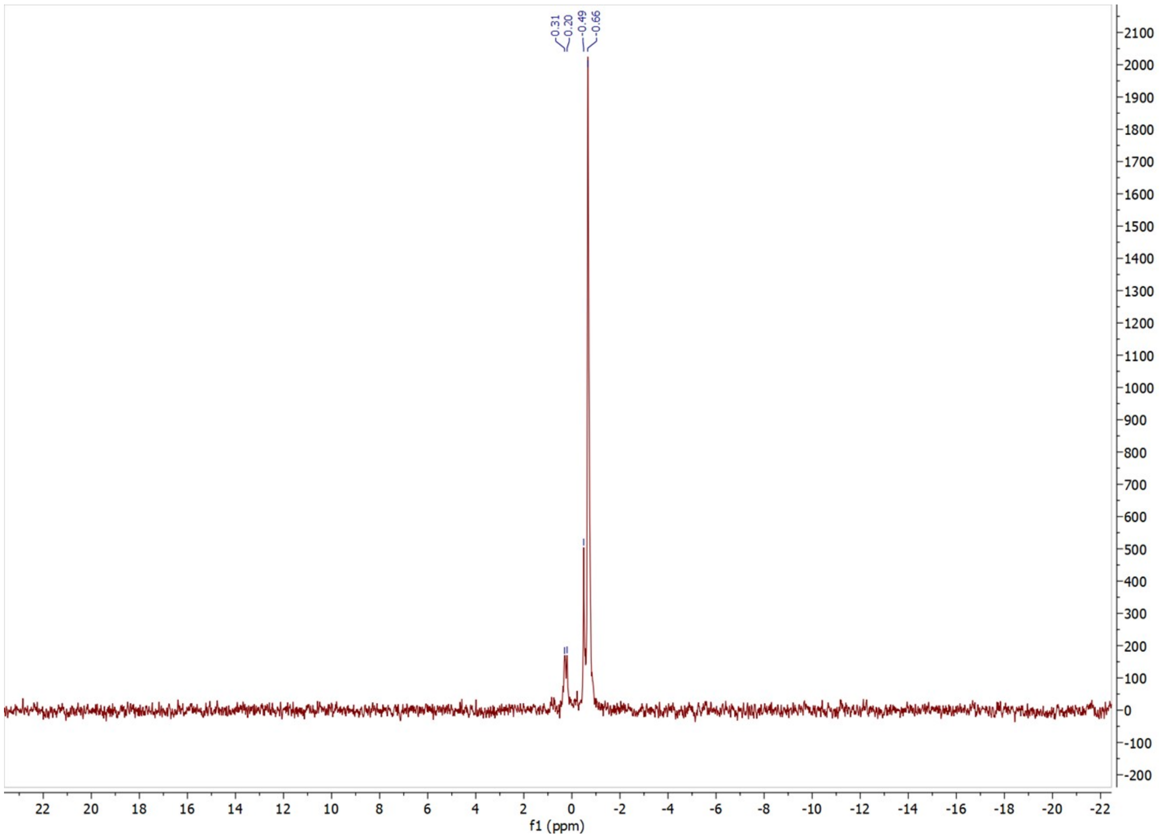

Figure S104. ^{31}P NMR spectrum (162 MHz) of **MeO-PEO-*b*-PEEP** (obtained by a flow procedure after dialysis).

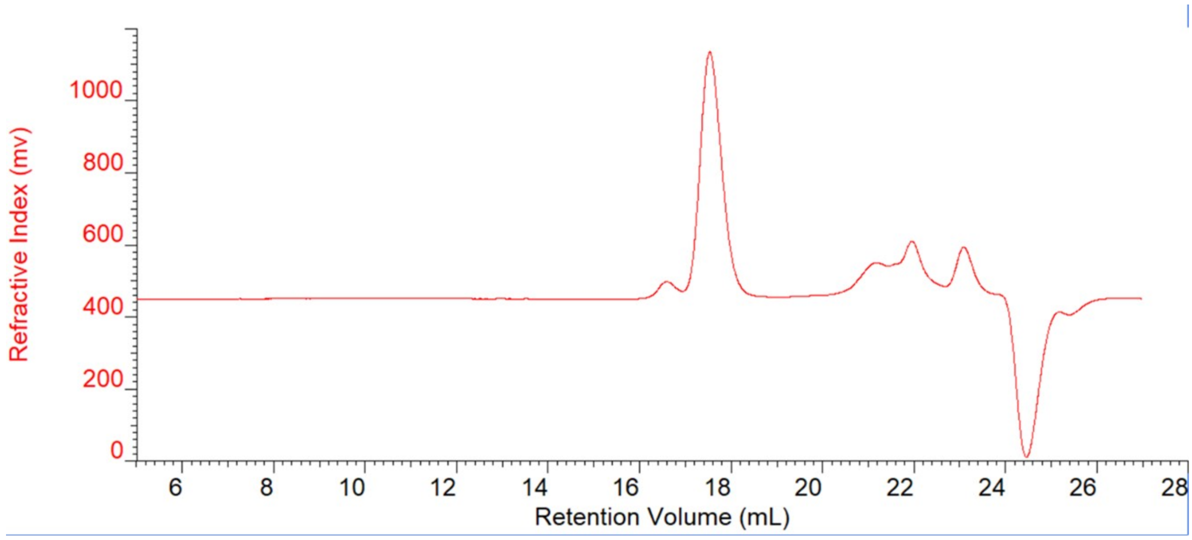

Figure S105. SEC chromatogram of **MeO-PEO-*b*-PEEP** (obtained by a flow procedure after dialysis).

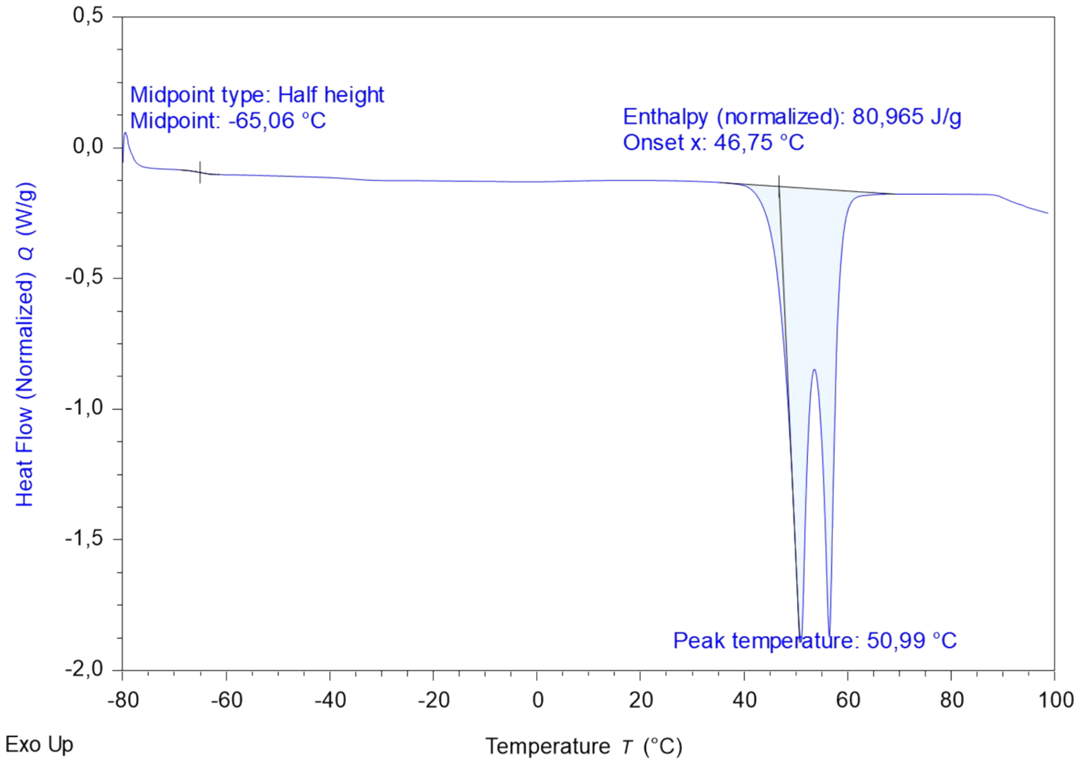

Figure S106. DSC thermogram of **MeO-PEO-*b*-PEEP** (obtained by a flow procedure after dialysis).

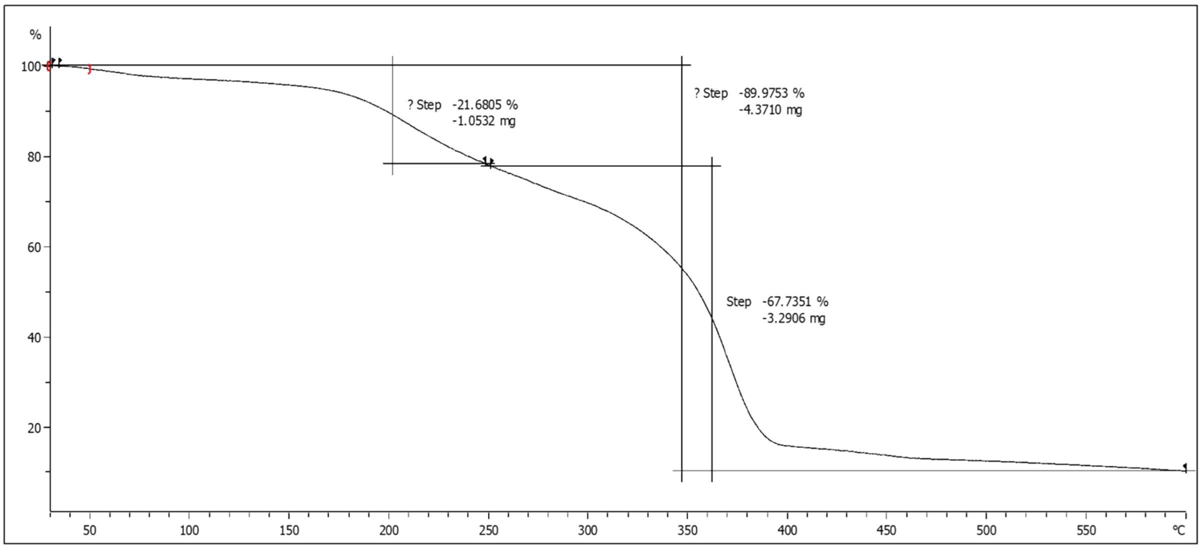

Figure S107. TGA thermogram of **MeO-PEO-*b*-PEEP** (obtained by a flow procedure after dialysis).

2.8.5 **BzO-PEEP** (batch process)

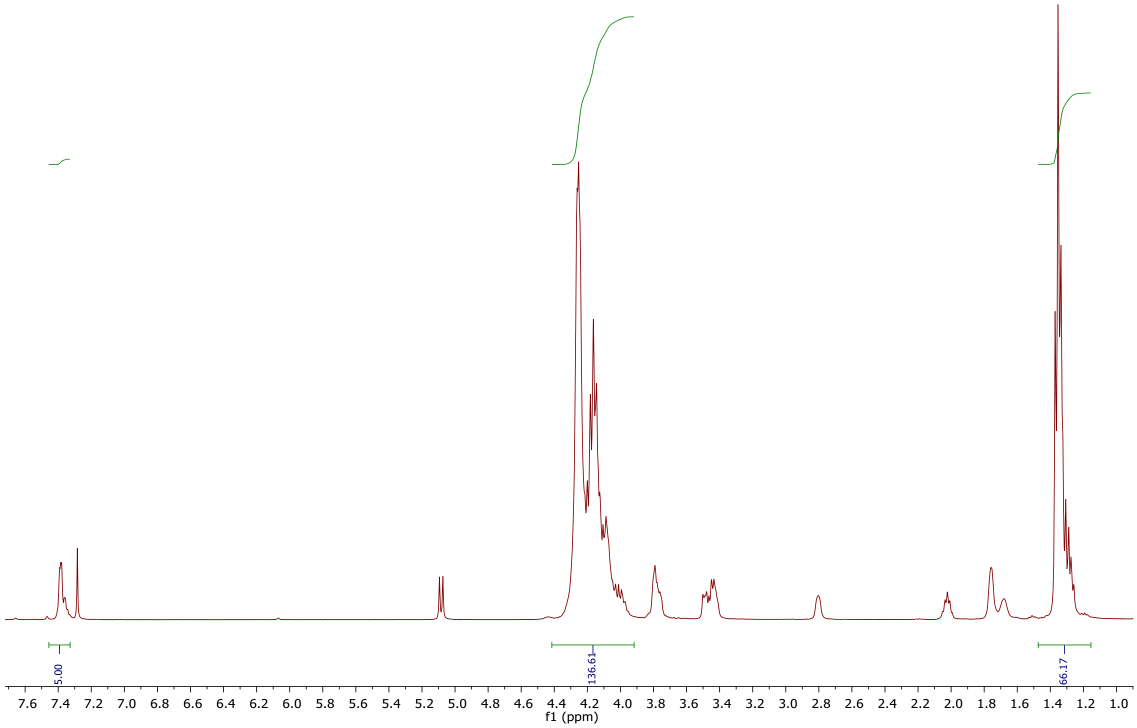

Figure S108. ^{1}H NMR spectrum (400 MHz) of **BzO-PEEP** (batch process, benzyl alcohol initiator).

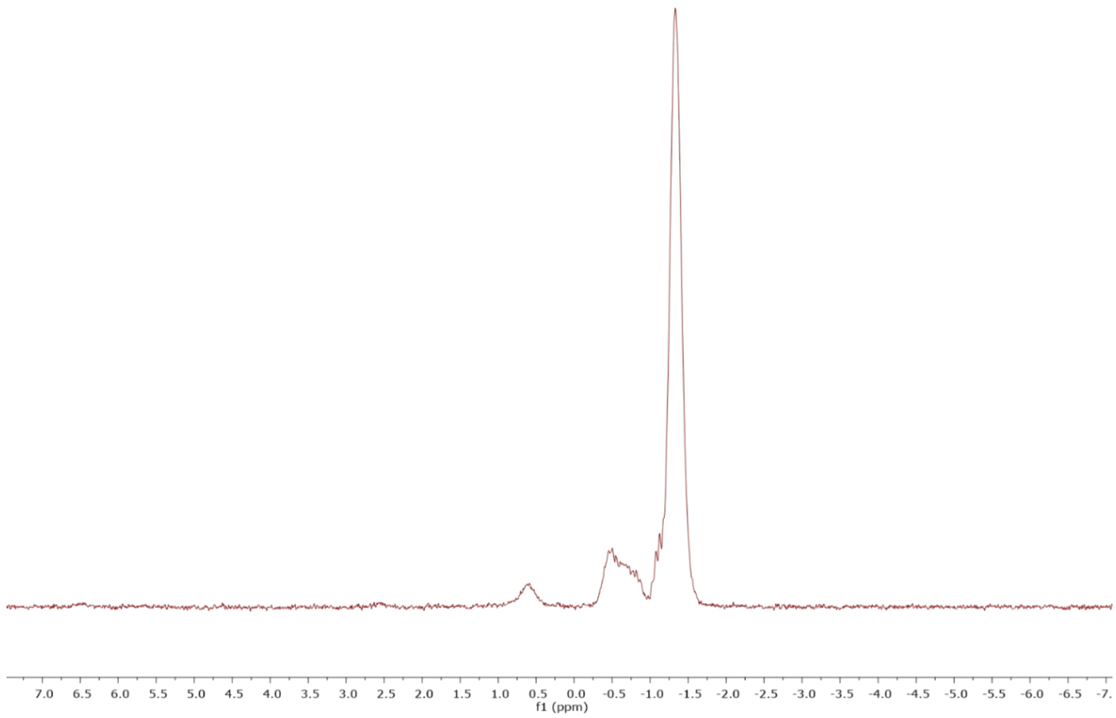

Figure S109. ^{31}P NMR spectrum (162 MHz) of **BzO-PEEP** (batch process).

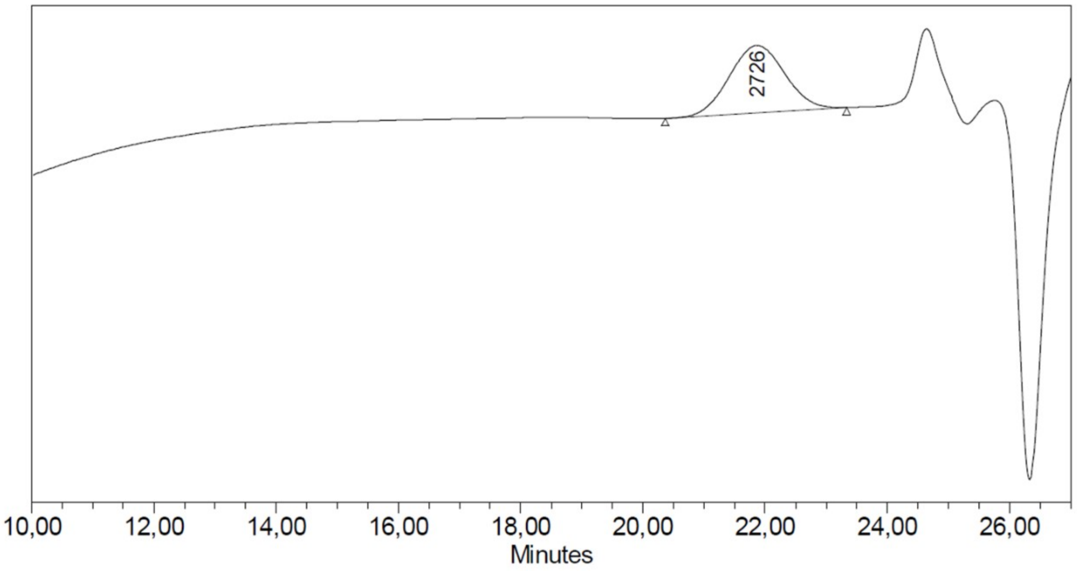

Figure S110. SEC chromatogram of **BzO-PEEP** (batch process).

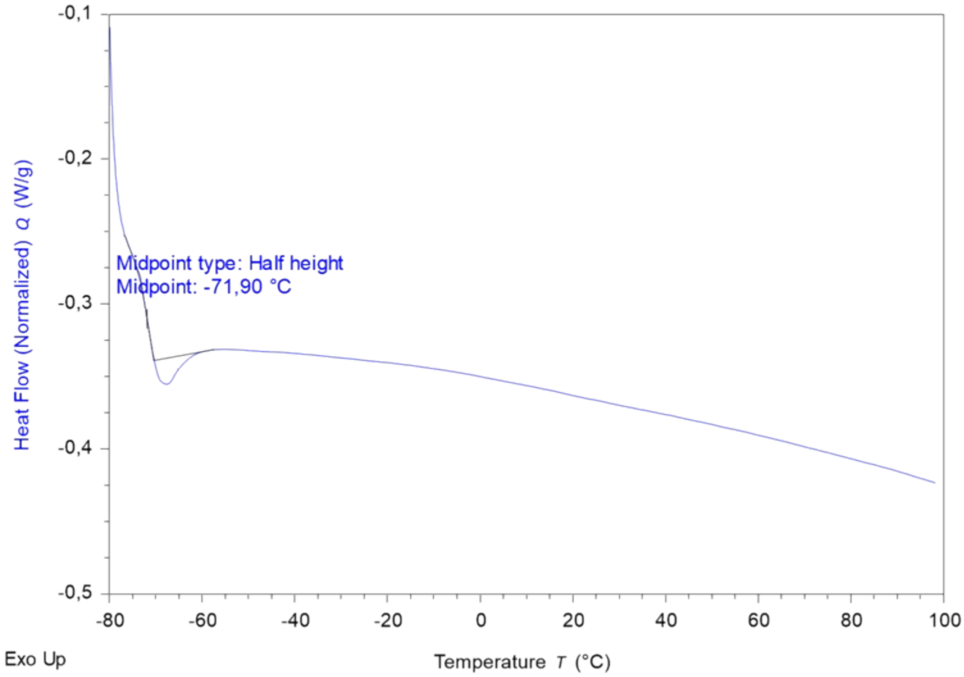

Figure S111. DSC thermogram of **BzO-PEEP** (batch process).

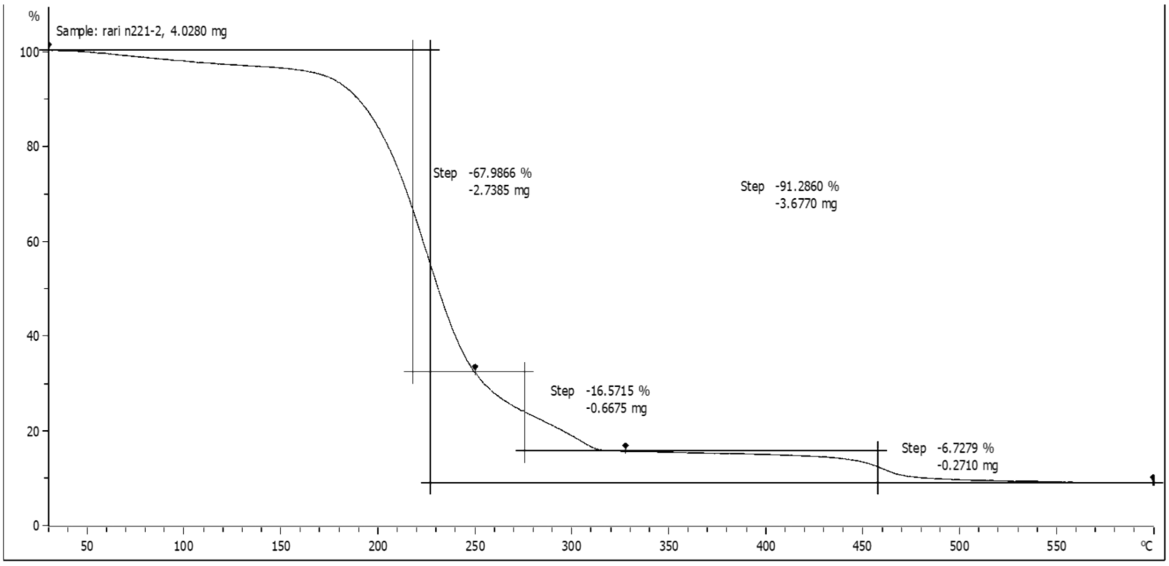

Figure S112. TGA thermogram of **BzO-PEEP** (batch process).

2.8.6 **PEEP-*co*-PEClMEP** (**EEP**/**EClMEP**, 75:25)

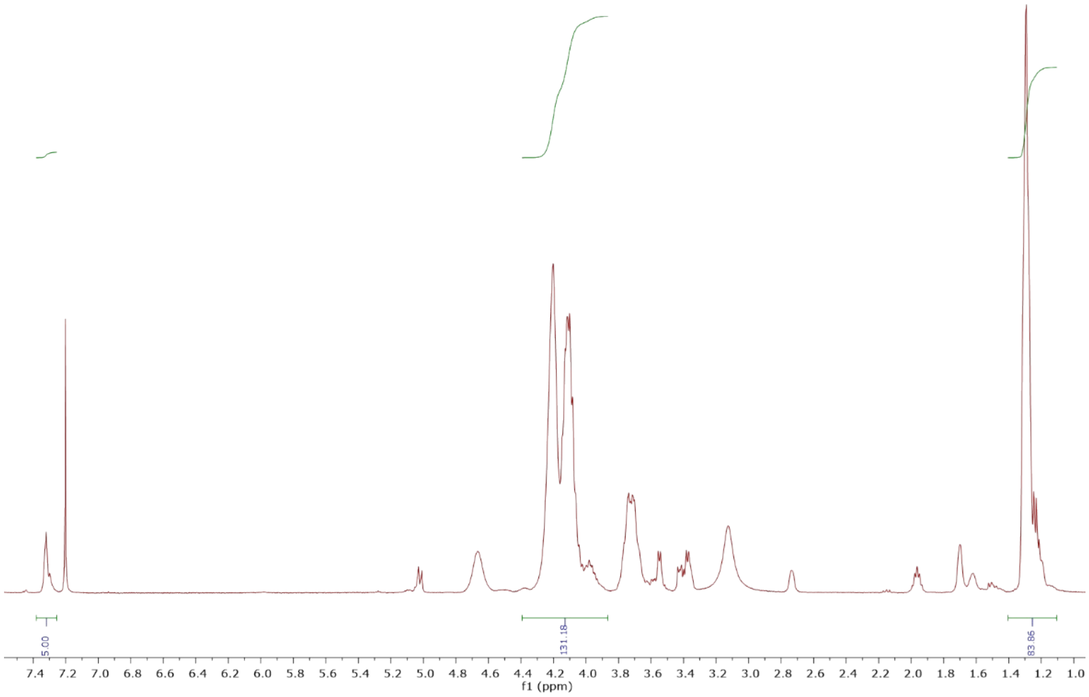

Figure S113. ^{1}H NMR spectrum (400 MHz) of **PEEP-*co*-PEClMEP** (**EEP**/**EClMEP**, 75:25).

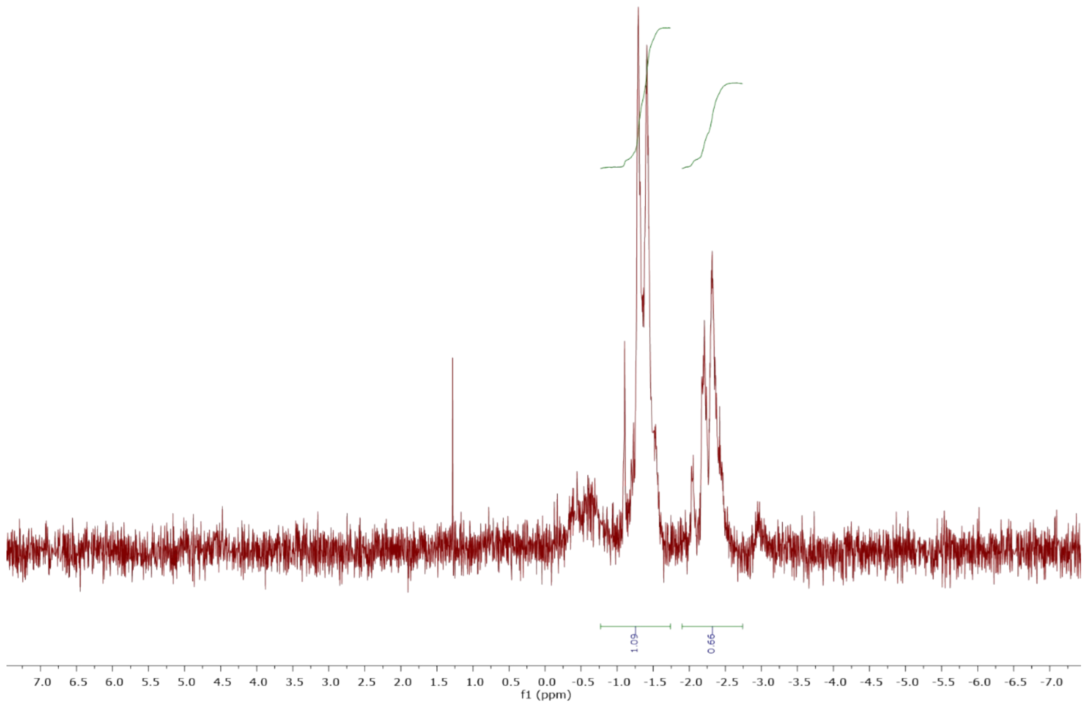

Figure S114. ^{31}P NMR spectrum (162 MHz) of **PEEP-*co*-PEClMEP** (**EEP/EClMEP**, 75:25).

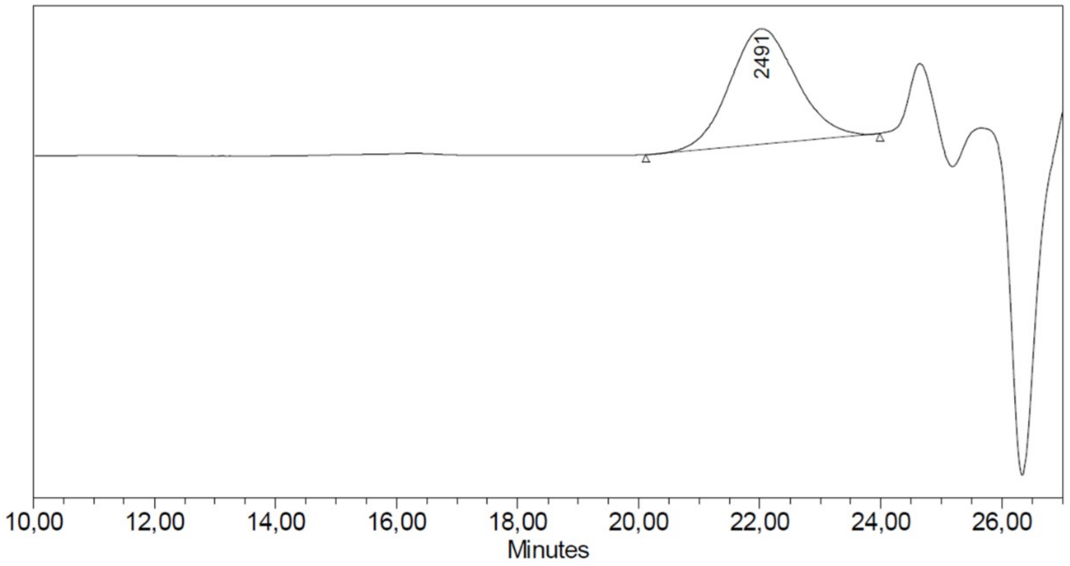

Figure S115. SEC chromatogram of **PEEP-*co*-PEClMEP** (**EEP/EClMEP**, 75:25).

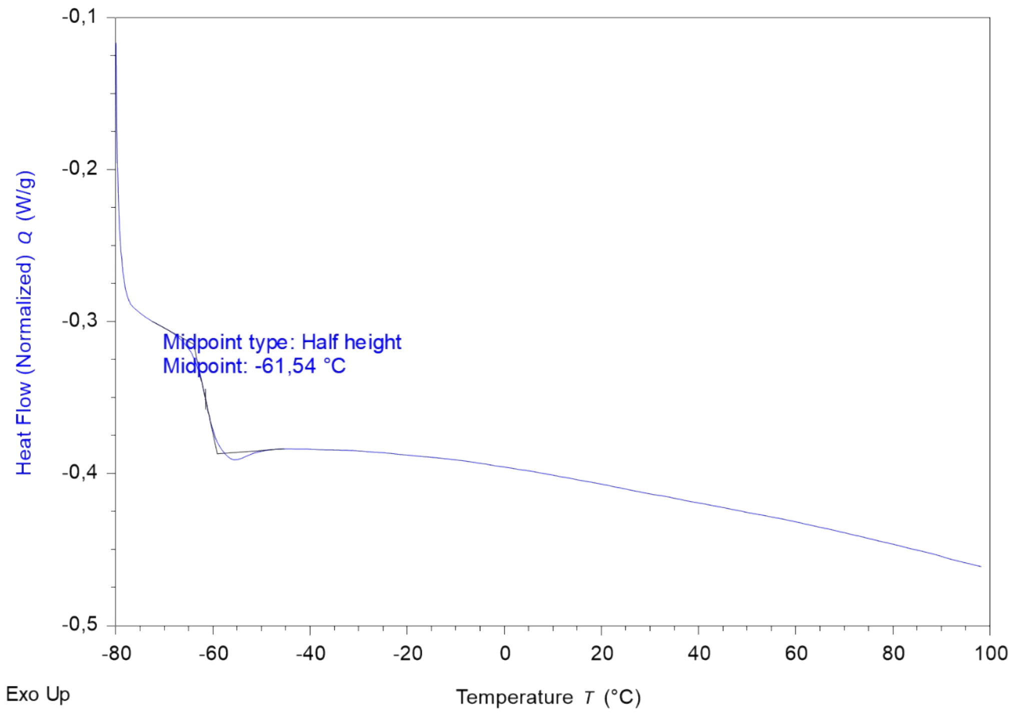

Figure S116. DSC thermogram of **PEEP-*co*-PEClMEP** (**EEP/EClMEP**, 75:25).

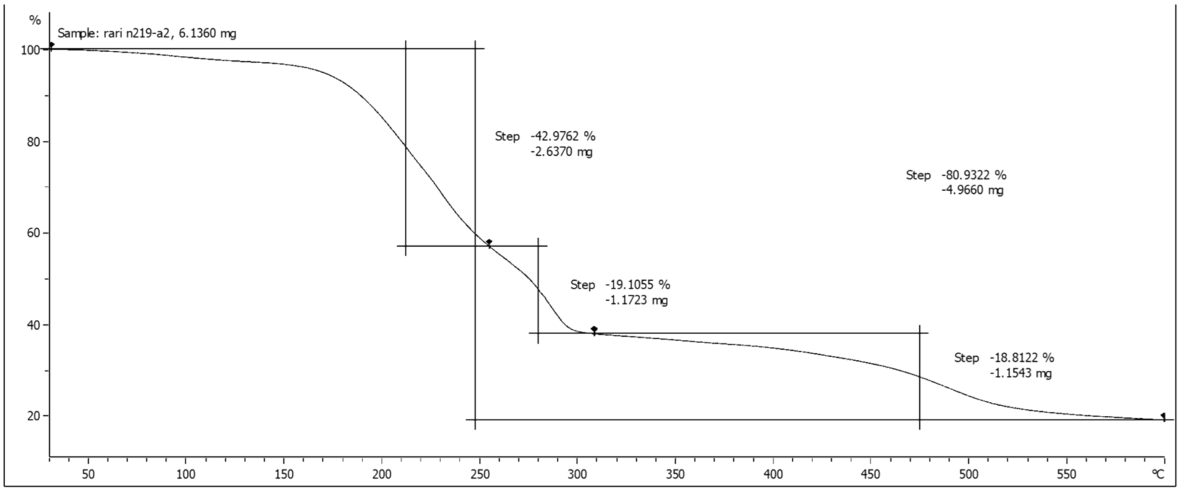

Figure S117. TGA thermogram of **PEEP-*co*-PEClMEP** (**EEP/EClMEP**, 75:25).

2.8.7 **PEEP-*co*-PEClMEP** (**EEP/EClMEP**, 50:50)

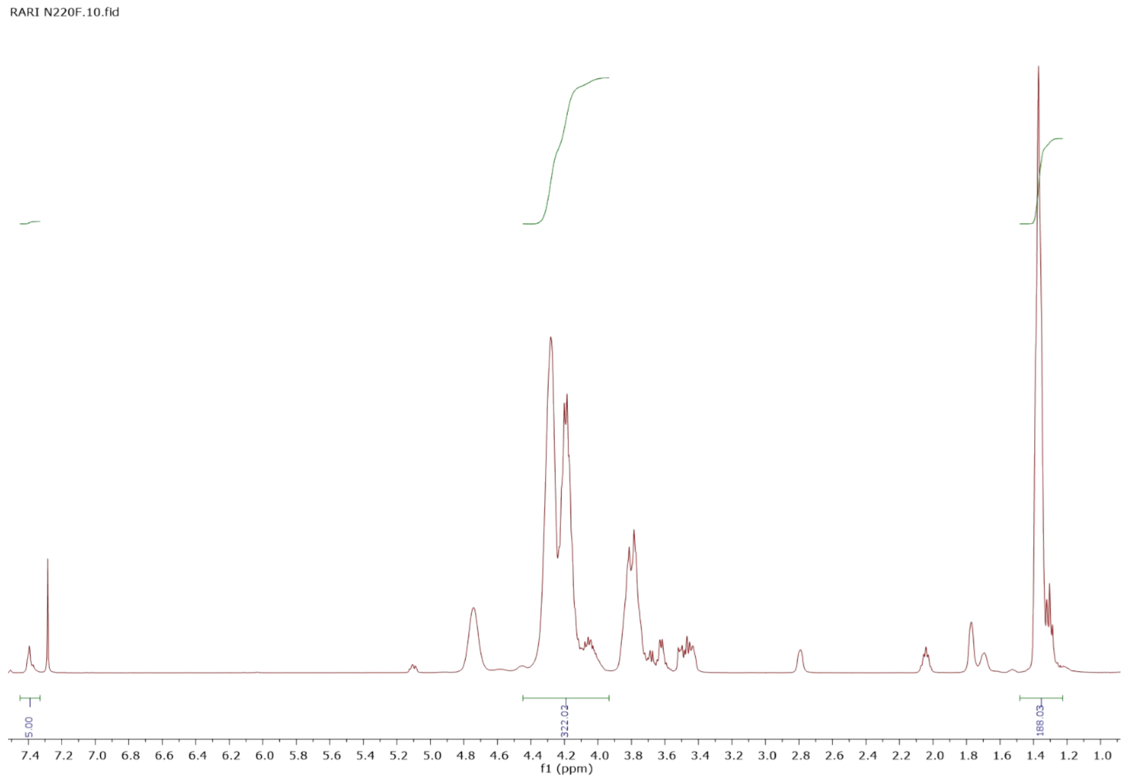

Figure S118. ^{1}H NMR spectrum (400 MHz) of **PEEP-*co*-PEClMEP** (**EEP/EClMEP**, 50:50).

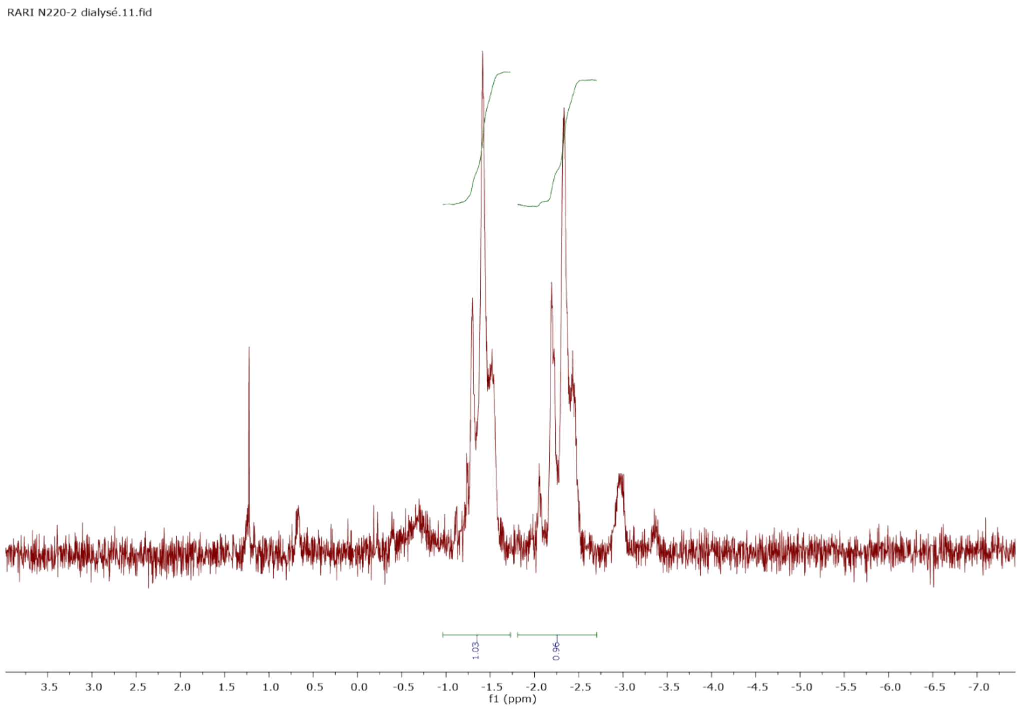

Figure S119. ^{31}P NMR spectrum (162 MHz) of **PEEP-*co*-PEClMEP** (**EEP/EClMEP**, 50:50).

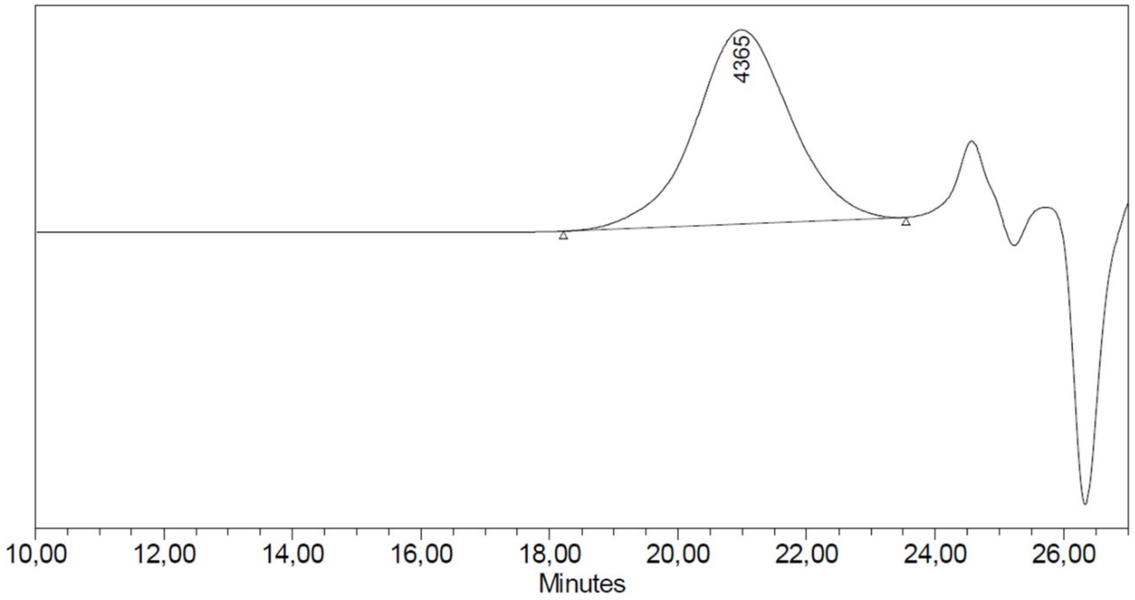

Figure S120. SEC chromatogram of **PEEP-*co*-PEClMEP** (**EEP**/**EClMEP**, 50:50).

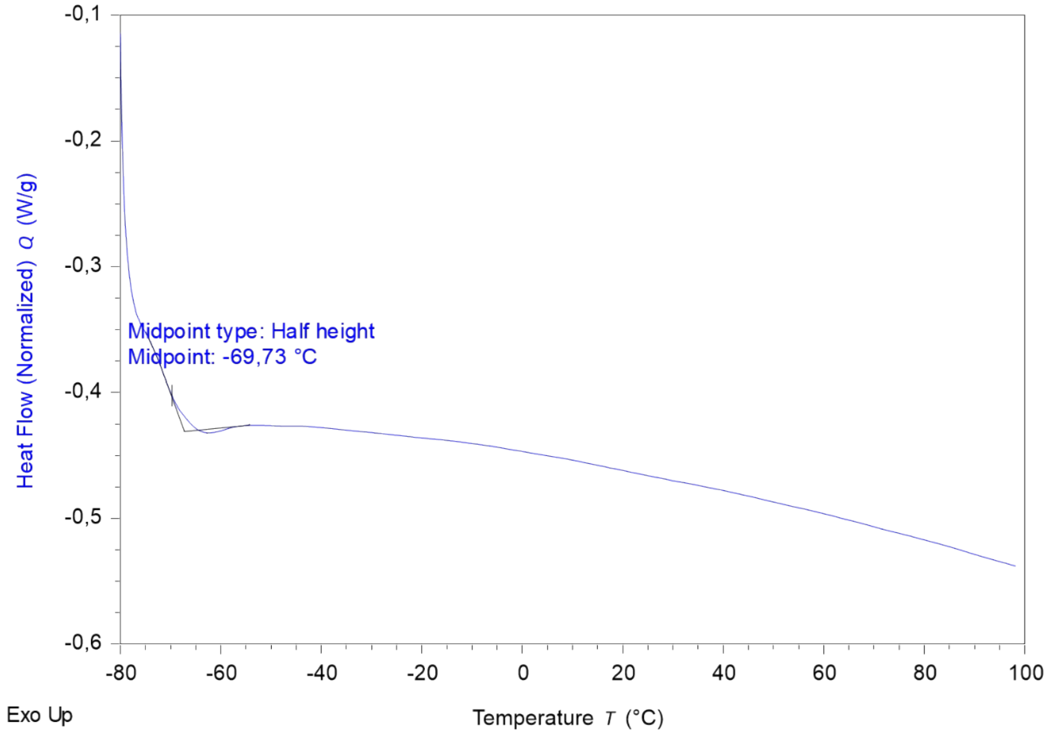

Figure S121. DSC thermogram of **PEEP-*co*-PEClMEP** (**EEP**/**EClMEP**, 50:50).

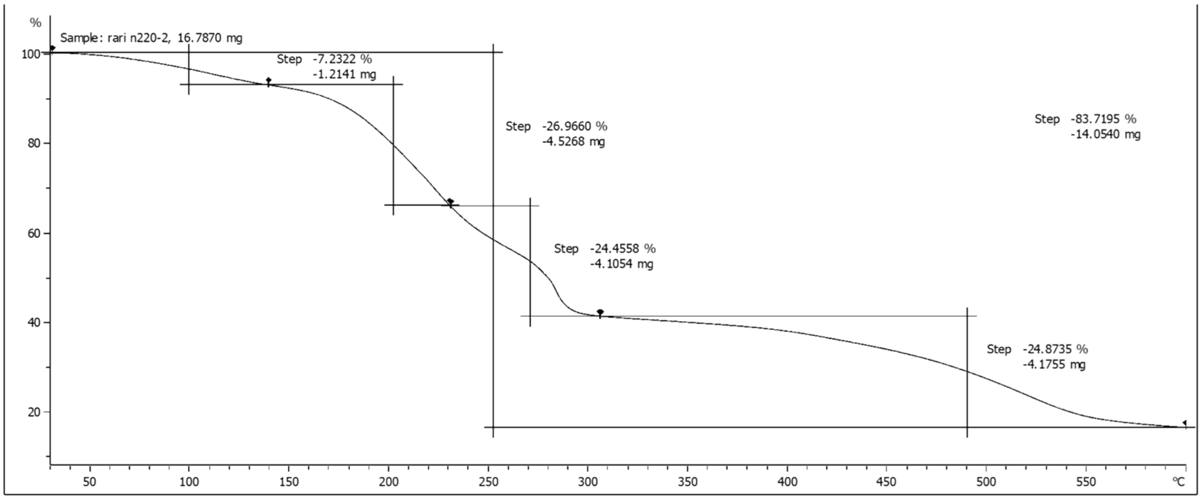

Figure S122. TGA thermogram of **PEEP-*co*-PEClMEP** (**EEP**/**EClMEP**, 50:50).

3 References

(1) Pratt, R. C.; Lohmeijer, B. G. G.; Long, D. A.; Lundberg, P. N. P.; Dove, A. P.; Li, H.; Wade, C. G.; Waymouth, R. M.; Hedrick, J. L. Exploration, Optimization, and Application of Supramolecular Thiourea−Amine Catalysts for the Synthesis of Lactide (Co)Polymers. *Macromolecules* **2006**, *39* (23), 7863–7871.

(2) Biela, T.; Penczek, S.; Slomkowski, S.; Vogl, O. Racemic and Optically Active Poly(4-Methyl-2-Oxo-2-Hydro-1,3,2-Dioxaphospholane). Synthesis and Oxidation to the Polyacids. *Die Makromol. Chemie, Rapid Commun.* **1982**, *3* (10), 667–671.

(3) Nifant'ev, I. E.; Shlyakhtin, A. V; Bagrov, V. V; Komarov, P. D.; Kosarev, M. A.; Tavtorkin, A. N.; Minyaev, M. E.; Roznyatovsky, V. A.; Ivchenko, P. V. Controlled Ring-Opening Polymerisation of Cyclic Phosphates, Phosphonates and Phosphoramidates Catalysed by Heteroleptic BHT-Alkoxy Magnesium Complexes. *Polym. Chem.* **2017**, *8* (44), 6806–6816.

(4) Clément, B.; Grignard, B.; Koole, L.; Jérôme, C.; Lecomte, P. Metal-Free Strategies for the Synthesis of Functional and Well-Defined Polyphosphoesters. *Macromolecules* **2012**, *45* (11), 4476–4486.

(5) Krüger, P.; Weberndörfer, B.; Werner, H. Synthese Und Molekülstruktur Chiraler Bis(1,3,2-Dioxaphospholane). *Zeitschrift für Anorg. und Allg. Chemie* **2000**, *626* (10), 2228–2234.

(6) Hommer, H.; Cuevas, G.; Gordillo, B. Kinetic Studies of the Thermal Cis-to-Trans Isomerization of Dioxaphospholanes. *Phosphorus Sulfur Relat. Elem.* **2008**, *183* (10), 2421–2437.

(7) Denney, D. B.; Denney, D. Z.; Liu, L.-T. PREPARATION AND STRUCTURAL STUDIES OF A NUMBER OF HETEROCYCLIC PHOSPHORANES. *Phosphorus Sulfur Relat. Elem.* **1985**, *22* (1), 71–84.

(8) Granata, A.; Argyropoulos, D. S. 2-Chloro-4,4,5,5-Tetramethyl-1,3,2-Dioxaphospholane, a Reagent for the Accurate Determination of the Uncondensed and Condensed Phenolic Moieties in Lignins. *J. Agric. Food Chem.* **1995**, *43* (6), 1538–1544.

(9) Nifantiev, E. E.; Sorokina, S. F.; Borisenko, A. A.; Zavalishina, A. I.; Vorobjeva, L. A. Cyclic Organic Derivatives of Hypophosphorous Acid. *Tetrahedron* **1981**, *37* (18), 3183–3194.

(10) McGuigan, C.; Swords, B. Synthesis of Phospholipids by Phosphoramidite Methodology. *J. Chem. Soc. Perkin Trans. 1* **1990**, No. 3, 783–787.

(11) Bhanthumnavin, W.; Bentrude, W. G. Effect of Amino Substituents on the Stereochemical Outcome of the Photo-Arbuzov Rearrangements of 1-Arylethyl Phosphorodiamidites. *J. Org. Chem.* **2005**, *70* (12), 4643–4651.

(12) Schöttler, S.; Becker, G.; Winzen, S.; Steinbach, T.; Mohr, K.; Landfester, K.; Mailänder, V.; Wurm, F. R. Protein Adsorption Is Required for Stealth Effect of Poly(Ethylene Glycol)- and Poly(Phosphoester)-Coated Nanocarriers. *Nat. Nanotechnol.* **2016**, *11* (4), 372–377.

(13) Wang, W.; Jiang, S.; Li, S.; Yan, X.; Liu, S.; Mao, X.; Yu, X. Functional Choline Phosphate Lipids for Enhanced Drug Delivery in Cancer Therapy. *Chem. Mater.* **2021**, *33* (2), 774–781.
